# Pharmacological targets of SGLT2 inhibitors on IgA nephropathy and membranous nephropathy: a mendelian randomization study

**DOI:** 10.3389/fphar.2024.1399881

**Published:** 2024-05-22

**Authors:** Xin Lv, Yan Shang, Yong Ning, Weimin Yu, Jian Wang

**Affiliations:** ^1^ Department of Nephrology, Shanxi Bethune Hospital, Shanxi Academy of Medical Sciences, Tongji Shanxi Hospital, Third Hospital of Shanxi Medical University, Taiyuan, China; ^2^ Tongji Hospital, Tongji Medical College, Huazhong University of Science and Technology, Wuhan, China; ^3^ Department of Nephrology, First Affiliated Hospital of Gannan Medical University, Ganzhou, China

**Keywords:** SGLT2 inhibitors, membranous nephropathy, IgA nephropathy, co-location analysis, mendelian randomization

## Abstract

**Introduction:**

Emerging research suggests that sodium-glucose cotransporter 2 (SGLT2) inhibitors may play a pivotal role in the treatment of primary glomerular diseases. This study was aimed to investigate potential pharmacological targets connecting SGLT2 inhibitors with IgA nephropathy (IgAN) and membranous nephropathy (MN).

**Methods:**

A univariate Mendelian randomization (MR) analysis was conducted using publicly available genome-wide association studies (GWAS) datasets. Co-localization analysis was used to identify potential connections between target genes and IgAN and MN. Then, Comparative Toxicogenomics Database (CTD) was employed to predict diseases associated with these target genes and SGLT2 inhibitors (canagliflozin, dapagliflozin, and empagliflozin). Subsequently, phenotypic scan analyses were applied to explore the causal relationships between the predicted diseases and target genes. Finally, we analyzed the immune signaling pathways involving pharmacological target genes using the Kyoto encyclopedia of genes and genomes (KEGG).

**Results:**

The results of MR analysis revealed that eight drug targets were causally linked to the occurrence of IgAN, while 14 drug targets were linked to MN. In the case of IgAN, LCN2 and AGER emerged as co-localized genes related to the pharmacological agent of dapagliflozin and the occurrence of IgAN. LCN2 was identified as a risk factor, while AGER was exhibited a protective role. KEGG analysis revealed that LCN2 is involved in the interleukin (IL)-17 immune signaling pathway, while AGER is associated with the neutrophil extracellular traps (NETs) signaling immune pathway. No positive co-localization results of the target genes were observed between two other SGLT2 inhibitors (canagliflozin and empagliflozin) and the occurrence of IgAN, nor between the three SGLT2 inhibitors and the occurrence of MN.

**Conclusion:**

Our study provided evidence supporting a causal relationship between specific SGLT2 inhibitors and IgAN. Furthermore, we found that dapagliflozin may act on IgAN through the genes LCN2 and AGER.

## 1 Introduction

IgA nephropathy (IgAN) and membranous nephropathy (MN) are the predominant pathological classifications in primary glomerular disease, and they also represent significant subtypes of chronic kidney disease (CKD) (Ronco et al., 2021; Floege, 2023). According to the recommendations of the Kidney Disease: Improving Global Outcomes (KDIGO) guidelines (KDIGO, 2021), patients with CKD who have low or moderate levels of urinary protein and renal dysfunction typically receive treatment through dietary and lifestyle adjustments, along with non-immunosuppressive drugs. These interventions aim to protect kidney function and slow down the progression of renal impairment (Kalantar-Zadeh et al., 2021). One therapeutic approach recommended for delaying the progression of nephropathy is the inhibition of the renin-angiotensin system using angiotensin-converting enzyme inhibitors or angiotensin receptor blocker analogues. Although this strategy has been proven to be somewhat effective, its effects are ultimately limited (Sanz et al., 2019). Finerenone, a novel selective non-steroidal mineralocorticoid receptor antagonist, has shown a protective effect in large-scale clinical studies involving CKD patients, reducing the occurrence of cardiac and renal complications (Agarwal et al., 2022; Ruilope et al., 2023). However, the long-term impact of this treatment requires further observation and investigation.

Sodium-glucose cotransporter 2 (SGLT2) inhibitors play a pivotal role in the management of type 2 diabetes by enhancing glucose excretion in urine, resulting in a reduction in blood glucose levels. These inhibitors have also been associated with demonstrated renal protective properties (Vallon and Verma, 2021). One notable study, CREDENCE, revealed that canagliflozin can substantially decrease the risk of adverse renal outcomes by an impressive 34% in diabetic patients (Perkovic et al., 2019). Additionally, the results of the EMPA-KIDNEY trial indicated that empagliflozin effectively mitigated the risk of kidney disease progression in patients at risk of CKD progression (Herrington et al., 2023). IgAN and MN are two of the most prevalent CKD subtypes. Two clinical studies have shown a protective effect of SGLT2 inhibitors in IgAN. The investigation of Dapagliflozin and Prevention of Adverse Chronic Kidney Disease Outcomes (DAPA-CKD) study in patients with IgAN showed that dapagliflozin reduced the urinary albumin-creatinine ratio by 26% compared to placebo (Wheeler et al., 2021). Another study also demonstrated a significant reduction in proteinuria, with a notable decrease of 27.1%, following the administration of SGLT2 inhibitors in IgAN patients undergoing full-dose therapy with angiotensin-converting enzyme inhibitors or angiotensin receptor blockers over a period of 6 months (Dong et al., 2023). Currently, there are no high-quality clinical studies confirming the benefits of SGLT2 inhibitors in MN patients.

Although some studies have explored the effect of SGLT2 inhibitors on renal diseases, a comprehensive understanding of their exact mechanisms and roles in primary renal diseases remains a topic for further research. Mendelian randomization (MR), a methodology rooted in Mendelian genetic principles and instrumental variable (IV) estimation, has garnered increasing attention in the medical research community for its capacity to deduce causal relationships in the presence of unobserved confounding factors (Sanderson et al., 2022). The application of MR has undergone rapid evolution, particularly in the areas of substantiating disease causation and screening potential drug targets, attributing to the advent of large-sample genome-wide association studies (GWAS) that encompass extensive data on the interplay between exposure, disease, and genetic variations (Mountjoy et al., 2021). By focusing on three specific SGLT2 inhibitors (canagliflozin, dapagliflozin, and empagliflozin) and leveraging expression Quantitative Trait Loci (eQTL) data of SGLT2 inhibitor target genes as exposure factors, the study used MR to explore potential pharmacological targets for SGLT2 inhibitors on IgAN and MN, aiming to provide a theoretical basis for the clinical application of SGLT2 inhibitors in these two diseases.

## 2 Methods

### 2.1 Study design

**FIGURE 1:**
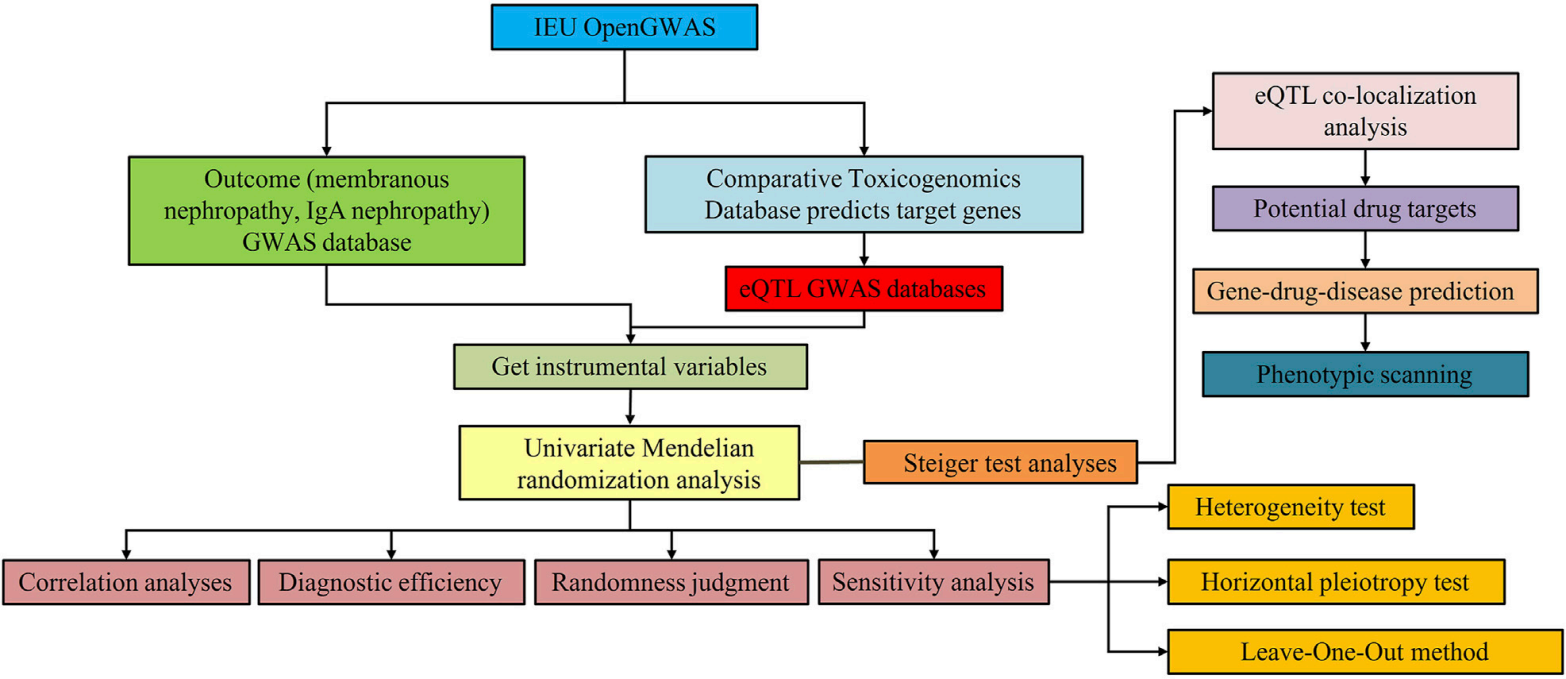
Study design for identification of SGLT2 inhibitors causally associated with IgA nephropathy and membranous nephropathy.

The overall design of this study is shown in Figure 1. There were three critical assumptions in MR studies: (a) a strong correlation existed between IVs and exposure, (b) IVs were independent of confounding factors related to exposure and outcome, and (c) IVs solely influenced the outcome through exposure without involvement in other biological pathways. In this investigation, univariate MR analyses were conducted to explore the bidirectional causal relationship between SGLT2 inhibitor targets and MN, as well as IgAN. The application of publicly available data from established databases eliminated the need for additional ethical approval.

### 2.2 Data source and pre-processing

The target genes associated with three SGLT2 inhibitors, namely, canagliflozin, dapagliflozin, and empagliflozin, were obtained from the Comparative Toxicogenomics Database (CTD). For subsequent analysis, the complete set of drug target genes, excluding SGLT2 (SLC5A2), a previously established target, was considered. The eQTL GWAS datasets corresponding to these target genes were collected from the Integrative Epidemiology Unit Open GWAS database (https://gwas.mrcieu.ac.uk/).

The GWAS datasets related to IgAN (ieu-a-1081) consisted of 278,077 single nucleotide polymorphisms (SNPs) and included a cohort of 595,700 samples. Additionally, the dataset for MN (ebi-a-GCST010005) encompassed 797,900 samples and featured 532,768 SNPs. Subsequently, the “extract instruments” function within the “TwoSampleMR” package (version 0.5.6) (Lu et al., 2023) was utilized to meticulously screen SNPs as IVs. Independent SNPs that demonstrated significant relevance to the exposure factors were carefully chosen, adhering to stringent criteria: *p* < 5 × 10^-8^, *R* ^2^ ≥ 0.001, and linkage disequilibrium distances ≥10 kb.

### 2.3 Process of MR analysis

After the IVs were filtered, MR analyses were conducted by integrating the “mr” function with five distinct methodologies: MR Egger (Bowden et al., 2015), weighted median (Bowden et al., 2016), inverse variance weighted (IVW) (Burgess et al., 2015), simple mode, and weighted mode (Hartwig et al., 2017). The results were mainly referenced to IVW. Subsequently, odds ratios (ORs) were computed, where values exceeding one indicated the presence of a risk factor, while values below one signified a protective factor. The outcomes were visually represented through scatter plots, forest plots, and funnel plots.

To ensure the reliability of the analysis results, we conducted a sensitivity analysis using a heterogeneity test, Horizontal Pleiotropy test, and the Leave-One-Out (LOO) method. In the heterogeneity test, a Quantile *p*-value (Q_pval) exceeding 0.05 suggested the absence of heterogeneity, while a *p*-value greater than 0.05 in the Horizontal Pleiotropy test indicated the absence of horizontal pleiotropy. The LOO method was used to identify potential outliers in the impact of each SNP. Furthermore, we employed the “TwoSampleMR” package to perform the Steiger test of directionality, which helped assess the causal connection between exposure factors and outcomes. Exposure factors identified as TRUE were retained for further in-depth analysis.

### 2.4 Co-localization and path analysis

The “coloc” package was employed to analyze the co-localization of eQTL-GWAS signals, focusing on the key target genes associated with IgAN and MN. A co-localization relationship was considered established when the value of *p*P.H4. abf exceeded 0.6. Visualization of these results was accomplished using the “gwasglue” and “gassocplot” packages. Additionally, the “ClusterProfiler” package was used to conduct KEGG pathway enrichment analyses on the key target genes that co-localized with diseases. This approach was aimed to elucidate the specific pathways in which these genes actively participated.

### 2.5 Potential drug and disease prediction

To understand the relationship between key target genes, drugs, and diseases, disease predictions for the key target genes and target drugs were made using the CTD database (Inference Score > 50). We retained diseases that presented direct evidence of predicted outcomes and featured among the top five in terms of relevance scores for each key target gene prediction, subsequently constructing a network. Causal relationships between these diseases and key target genes were then analyzed through phenotypic scan analyses.

## 3 Results

### 3.1 Causal effects of SGLT2 inhibitors targets on MN

**FIGURE 2:**
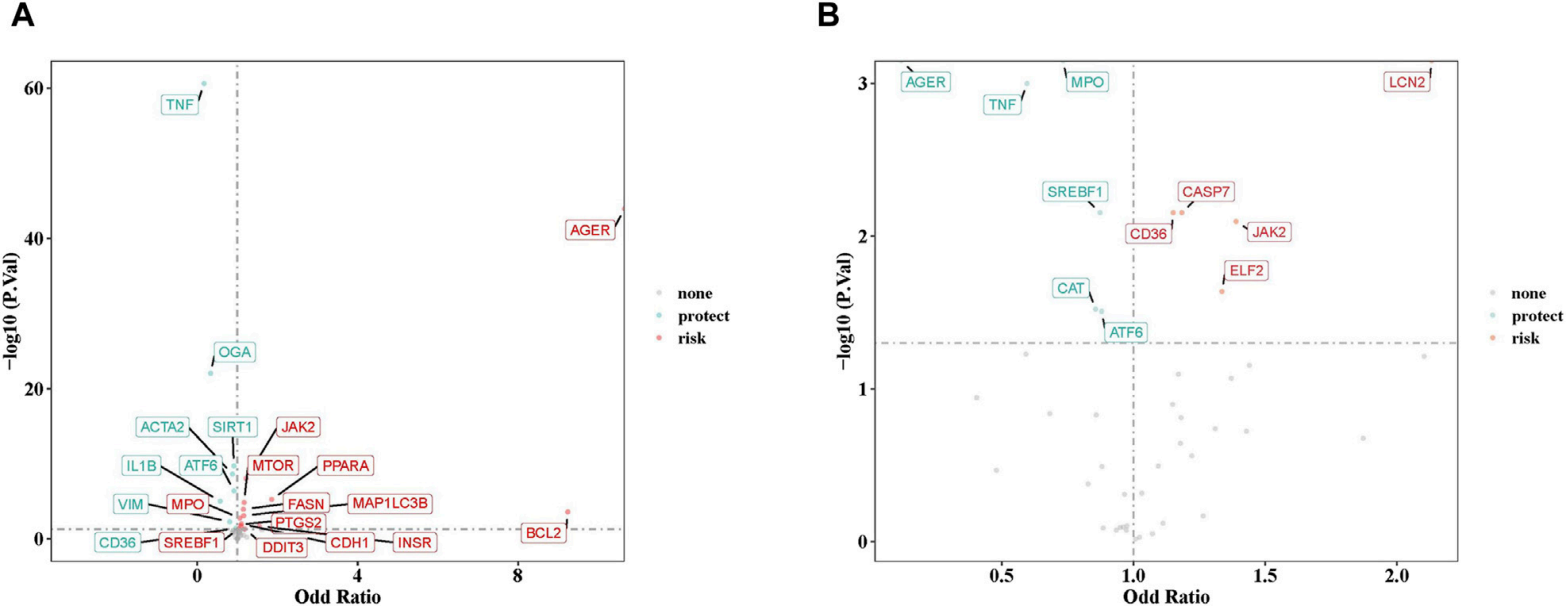
**(A)** The volcano plot of 21 drug targets on membranous nephropathy by odd ratio (OR) value. **(B)** The volcano plot of 11 drug targets on IgA nephropathy by OR value. Red indicates risk factors, green indicates protect factors.

After careful screening, we identified 49 independent SNPs as drug targets, which showed a strong association with MN and therefore served as IVs. Univariate MR analyses were conducted using five different methods, as detailed in Supplementary Table S1. The results from IVW analysis indicated that 21 drug targets had a causal association with the occurrence of MN (Supplementary Table S1). Examination of ORs revealed that TNF, OGA, ACTA2, SIRT1, IL1B, ATF6, VIM, and CD36 acted as protective factors against the occurrence of MN. On the other hand, other drug targets including AGER, MPO, SREBF1, BCL2, CDH1, FASN, INSR, JAK2, MAP1LC3B, MTOR, PPARA, PTGS2, and DDIT3, were identified as risk factors (Figure 2A). The robustness of our causal inference was further confirmed through scatter plots (Supplementary Figure S1) and forest plots (Supplementary Figure S2). Examination of the funnel plot (Supplementary Figure S3) revealed a random distribution of sample exposure factors, which aligned with Mendel’s second law of random grouping.

Heterogeneity tests revealed variation among the 49 drug targets for MN. Thirty-seven of these drug targets showed no significant heterogeneity (Q_pval >0.05), and were analyzed by MR using the fixed-effects IVW method. The remaining 12 drug targets exhibited heterogeneity (Q_pval <0.05), necessitating MR analysis using random effects IVW method (Supplementary Table S2). Subsequent investigation revealed that 11 out of the 49 drug targets exhibited lateral pleiotropy. Notably, five of these 11 genes demonstrating lateral pleiotropy were among the 21 drug targets causally related to MN. Following the exclusion of these five genes, 16 drug targets with a significant causal relationship were retained for further examination (Supplementary Table S3). Furthermore, the robustness of these findings was validated through the LOO sensitivity analysis, where all error lines were positioned to the right of 0 (Supplementary Figure S4). Steiger’s test confirmed that two of the 16 target genes with causal effects were in the incorrect direction (Supplementary Table S4), resulting in the retention of 14 target genes in the correct direction (ACTA2, ATF6, CD36, CDH1, IL1B, INSR, JAK2, MAP1LC3B, MTOR, OGA, PPARA, PTGS2, VIM, and DDIT3).

### 3.2 Lack of co-localization connection between target genes and MN

Following our initial findings, we further investigated through colocalization analyses to examine the potential shared genetic signals between MN and the identified 14 target genes. However, there were no positive co-localization results between MN and 14 genes with a significant causal relationship. This absence of co-localization provided compelling evidence that challenged the idea of an association driven by specific target genes within MN.

### 3.3 Causal effects of SGLT2 inhibitors targets on IgAN

In parallel, a similar MR analysis was conducted for IgAN. After a thorough screening process, 43 independent SNPs, designated as drug targets, showed strong associations with IgAN, thus serving as IVs. The univariate MR analysis, utilizing five different methodologies, is presented in Supplementary Table S5. Results from IVW method indicated that 11 drug targets had a causal association with the occurrence of IgAN. Analysis of ORs revealed that AGER, MPO, TNF, SREBF1, CAT, and ATF6 acted as protective factors against the development of IgAN, while other drug targets (CD36, CASP7, ELF2, JAK2, and LCN2) were identified as risk factors (Figure 2B). The robustness of our causal inference was further confirmed through scatter plots (Supplementary Figure S5) and forest plots (Supplementary Figure S6). Examination of the funnel plot (Supplementary Figure S7) showed a random distribution of sample exposure factors, in accordance with Mendel’s second law of random assortment.

Heterogeneity tests revealed variability among the 43 drug targets for IgAN. Out of the 43 drug targets analyzed, 38 showed no significant heterogeneity (Q_pval >0.05), and were subjected to MR using fixed-effects IVW method. The remaining five drug targets displayed heterogeneity (Q_pval <0.05), thus MR analysis was performed using random effects IVW method (Supplementary Table S6). Upon further investigation, three out of the 43 drug targets were found to demonstrate horizontal pleiotropy (Supplementary Table S7), and these were among the 11 drug targets causally related to IgAN. Consequently, these three genes were excluded, leaving eight drug targets with a significant causal relationship for subsequent examination, namely, AGER, ATF6, CASP7, JAK2, LCN2, MPO, CAT, and ELF2. Furthermore, the robustness of these findings was confirmed in the LOO sensitivity analysis, where all error lines were positioned to the right of 0 (Supplementary Figure S8). The Steiger test verified the correct directionality of the causal effects observed for the eight target genes (Supplementary Table S8).

### 3.4 IgAN and LCN2, AGER expression shared a causal variant

**FIGURE 3:**
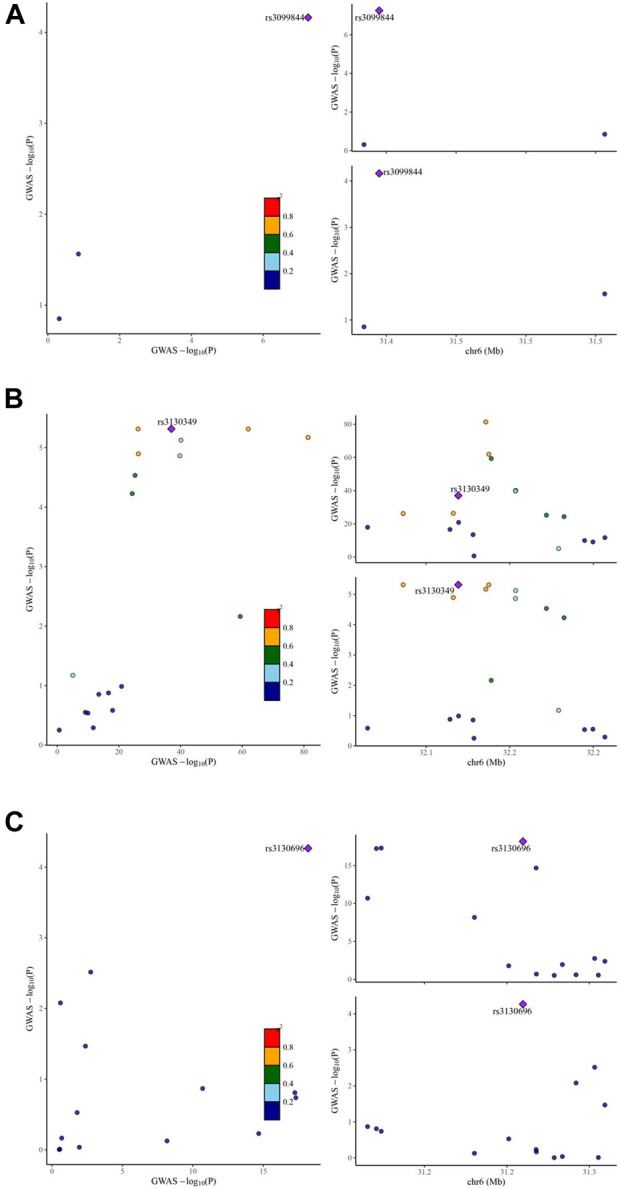
Visualization of the eQTL-GWAS colocalization results. The GWAS signal in the test for LCN2 colocalizes contains one lead variant, rs3099844 **(A)** in IgA nephropathy. The GWAS signal in the test for AGER colocalizes contains two lead variants, rs3130349 **(B)** and rs3130696 **(C)** in IgA nephropathy. Purple dot represents the independent single nucleotide polymorphisms (SNPs) associated with genetic liability to type IgA nephropathy in LCN2 locus and AGER locus.

To determine the presence of shared genetic signals between IgAN and the identified eight target genes, comprehensive colocalization analyses were conducted. Interestingly, LCN2 expression and IgAN were found to share a causal variant, known as rs3099844 (*p*P.H4 = 0.935,308) (Figure 3A). Similarly, AGER expression and IgAN also shared causal variants, specifically rs3130349 (*p*P.H4 = 0.988,317) and rs3130696 (*p*P.H4 = 0.961,451) (Figures 3B,C). These results suggest a positive association between LCN2 expression and IgAN, indicating that LCN2 could be a potential risk factor in the occurrence of IgAN. On the other hand, the analysis supported the idea that AGER might act as a protective factor against IgAN. Interestingly, both LCN2 and AGER were found to correspond to dapagliflozin (Supplementary Table S9). However, no evidence of positive co-localization of target genes was observed between the other two SGLT2 inhibitors (canagliflozin and empagliflozin) and the occurrence of IgAN.

### 3.5 Disease correlation analysis based on LCN2 and AGER

**FIGURE 4:**
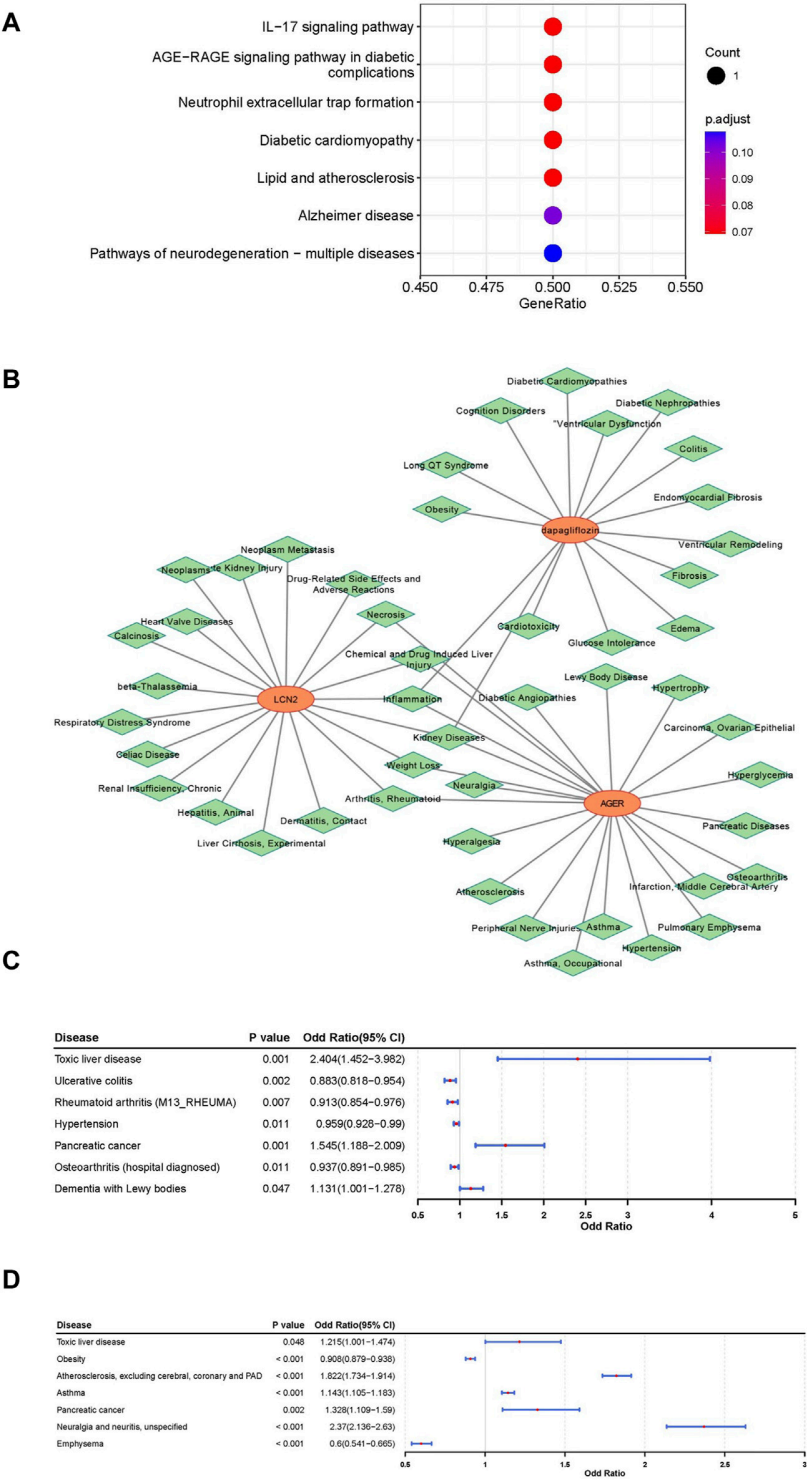
Comprehensive analysis for functional annotation and disease correlation based on LCN2 and AGER. **(A)** Kyoto Encyclopedia of Genes and Genomes (KEGG) enrichment analysis of LCN2 and AGER. **(B)** Construction of gene-drug -diseases network. **(C)** Forest map of correlation between other disease and LCN2. **(D)** Forest map of correlation between other disease and AGER.

An in-depth exploration of the biological pathways associated with LCN2 and AGER was conducted through KEGG pathway enrichment analysis. This analysis uncovered a significant association between LCN2 and the “interleukin (IL)-17 signaling pathway,” while AGER showed potential involvement in the “AGE-RAGE signaling pathway in diabetic complications” and “Neutrophil extracellular trap (NET) formation” (Figure 4A).

To understand the disease mechanisms related to these target genes and the potential side effects of interventions, predictions were made using the CTD Database. Our findings suggested that kidney diseases, arthritis, rheumatoid, inflammation and some other disorders may be simultaneously associated with both AGER and LCN2 **(** Figure 4B). Additionally, kidney diseases and some other diseases were associated with dapagliflozin. By conducting phenotypic scans under different physiological and disease conditions, we gained further insights into the effects of interventions on AGER and LCN2.

LCN2 showed a positive relationship with toxic liver disease, pancreatic cancer, and dementia with lewy bodies, suggesting potential therapeutic benefits of interfering with LCN2 in these conditions. Conversely, interventions targeting LCN2 were associated with an elevated potential risk for four other diseases (Figure 4C; Supplementary Figures S9–S12). Furthermore, AGER exhibited a negative relationship with Obesity and Emphysema, indicating potential therapeutic effects for these two diseases (Figure 4D; Supplementary Figures S13–S16).

## 4 Discussion

In this extensive research endeavor, we conducted a large-scale MR analysis to investigate genes associated with the occurrence of IgAN and MN, and examined the relationship between the target genes of three commonly used SGLT2 inhibitors—canagliflozin, dapagliflozin, and empagliflozin—and the pathogenesis genes of these two renal diseases. Our findings revealed a total of 14 genes associated with MN and eight genes related to IgAN. Furthermore, the study identified instances of co-localization between the target genes of SGLT2 inhibitors and the pathogenesis genes of MN and IgAN. Specifically, LCN2 and AGER emerged as genes co-located with the dapagliflozin target gene and the IgAN pathogenesis gene. Importantly, the study did not find a positive co-localization relationship between the target gene of SGLT2 inhibitors and the pathogenesis gene of MN.

AGER is a highly polymorphic gene, with its polymorphisms potentially influencing disease progression (Serveaux-Dancer et al., 2019). For instance, the incidence of rs1800624 SNP was found to be lower in patients with systemic lupus erythematosus (SLE) and lupus nephritis compared to the control group, whereas the rs3134940 SNP was significantly positively correlated with the severity of lupus nephritis (Martens et al., 2012). Interestingly, the effects of the same SNP can vary across different diseases; for example, the rs1800264 SNP demonstrated protective effects against chronic heart disease, atherosclerosis, and diabetic retinopathy in diabetic patients (Picheth et al., 2007; Prasad et al., 2010). However, it increased susceptibility to invasive aspergillosis in stem cell transplant recipients (Cunha et al., 2011). Our study indicated that the SGLT2 inhibitor dapagliflozin might modulate the rs3130349 and rs3130696 SNPs of the AGER gene for the treatment of IgAN.

The AGER Gene encodes the receptor for advanced glycation end-products (RAGE), serving as a non-specific multiligand pattern recognition receptor. RAGE may have a dual role in various physiological and pathological processes. Firstly, it can bind to different ligands influencing the progression of conditions such as diabetes, neurological disorders, cancer, inflammation, and other diseases (Dong et al., 2022). Specifically within the kidney, RAGE contributes to intrinsic renal cell injury in podocytes, mesangial cells, and tubular epithelial cells, while also facilitating leukocyte recruitment via interaction with different ligands, exacerbating renal inflammation and fibrosis (Curran and Kopp, 2022). Conversely, nuclear RAGE is crucial for the mechanisms of DNA damage repair (Dong et al., 2022). Notably, deficiency in RAGE impairs lung repair mechanisms, leading to pulmonary fibrosis (Englert et al., 2008; Englert et al., 2011), whereas overexpression of nuclear phosphomimetic RAGE promotes DNA repair, consequently mitigating inflammation and fibrosis in experimental diabetic mice (Kumar et al., 2020). Our MR analysis indicated that AGER might serve as a protective factor in IgAN, suggesting the potential benefit of the SGLT2 inhibitor dapagliflozin in IgAN through its interaction with AGER. However, further exploration and verification is required to elucidate the underlying mechanisms.

Polymorphisms also exist in the LCN2 gene. A study conducted among Hong Kong Chinese individuals revealed an association between the rs3814526 SNP of LCN2 and hypertension (Ong et al., 2011). Conversely, research involving breast cancer patients indicated elevated levels of LCN2 protein bothin the plasma and breast cancer cells compared to controls, although no significant correlation was observed with polymorphisms in the LCN2 gene (Linjawi et al., 2018). Our findings suggest a potential therapeutic mechanism whereby the SGLT2 inhibitor dapagliflozin may target the rs3099844 SNP of the LCN2 gene to inhibit LCN2 expression in the treatment of IgAN.

The gene LCN2 encodes the lipocalin-2 protein, commonly referred to as neutrophil gelatinase-associated lipocalin (NGAL). While NGAL is primarily secreted by activated neutrophils, it is also expressed and secreted by various human tissues such as the kidney, heart, and lung (Romejko et al., 2023). Elevated levels of NGAL have been linked to inflammatory conditions, arterial hypertension, obesity, diabetes, metabolic complications, carcinogenesis, and other diseases (Romejko et al., 2023). During the inflammatory response, NGAL isolates the iron carriers, and prevents bacteria from acquiring iron, thereby reducing bacterial growth and proliferation (Parrow et al., 2013).

In renal diseases, NGAL serves as an independent biomarker for acute kidney injury (Wen and Parikh, 2021). Elevated NGAL levels are observed across various CKD types, including IgAN (Rhee et al., 2015), SLE (Lindblom et al., 2022), diabetic nephropathy (Liu et al., 2022a), and autosomal dominant polycystic kidney disease (Meijer et al., 2010). Renal cell damage prompts the secretion of NGAL, thereby enhancing cell proliferation, further exacerbating renal injury and CKD progression by mediating signaling pathways such as epidermal growth factor receptor (EGFR) and hypoxia-inducible factor 1α (HIF-1α) (Romejko et al., 2023). Our MR analysis revealed an association between LCN2 and the occurrence of IgAN, suggesting the potential of the SGLT2 inhibitor dapagliflozin in mitigating IgAN by inhibiting LCN2.

Further investigations delved into the potential immune signaling pathways of LCN2 and AGER, revealing LCN2’s involvement in the IL-17 immune signal pathway and AGER’s association with NETs signaling immune pathway. IL-17 immune signal pathway (Lin et al., 2018; Tang et al., 2021; Ma et al., 2023) havs been pivotal for the onset and progression of IgAN. Additionally, NETs signaling immune pathway is involved in the pathogenesis of immune disorders such as Immunoglobulin A vasculitis (Chen et al., 2022) and SLE (Barnado et al., 2016). However, whether it is related to IgAN remains uncertain and warrants further investigation (Nakazawa et al., 2018). These findings imply that the SGLT2 inhibitor dapagliflozin may hold promise for the treatment of IgAN, potentially through its influence on the LCN2 and AGER genes and their associated immune signaling pathways, which need further validation.

SGLT2 inhibitors exert their renal protective mechanisms through various pathways. Experimental results indicate that SGLT2 inhibitors can enhance renal tubular autophagy (Lee et al., 2019; Tomita et al., 2020) and improve renal oxygenation (Bessho et al., 2019). Recent research has shed light on the impact of SGLT2 inhibitors on the immune system. For example, studies in mouse models of diabetic nephropathy have revealed that dapagliflozin can counteract the imbalance between T helper cell 17 and T regulatory cell by inhibiting SGK17 (Wang et al., 2021). Additionally, it has been demonstrated that dapagliflozin reduces the accumulation of macrophages, CD4^+^ T cells, and B cells in mouse models of aortic aneurysm (Liu et al., 2022b). Furthermore, canagliflozin has exhibited the ability to prevent T cell activation and proliferation, which is a vital mechanism in autoimmune conditions, by antagonizing T cell receptor signaling in patients with rheumatoid arthritis and systemic lupus erythematosus (Jenkins et al., 2023). Empagliflozin has also shown potential to restore the function and number of immature B cells in models of myocardial infarction (Xu et al., 2022). Our previous animal studies have demonstrated that canagliflozin may play a significant role in rats with MN by influencing the polarization of immune T helper cells and the deposition of abnormal IgG secreted by B cells in the kidney (Lv et al., 2022). These studies have provided compelling evidence suggesting that SGLT2 inhibitors can confer renal protection through their modulation of immune signals.

Clinical investigations have unequivocally established that different SGLT2 inhibitors exhibit varying degrees of renal protection. Our study specifically revealed that dapagliflozin stands out with potential therapeutic efficacy in addressing IgAN, suggesting that different SGLT2 inhibitors may indeed operate via distinct molecular mechanisms to achieve their renal protective effects. However, the precise nuances of these differences remain unclear and require further exploration. The disparities in renal protective effects associated with different SGLT2 inhibitors may be attributed to their unique organ distribution, pharmacokinetics, and modulation of immune signals. Notably, pharmacokinetic experiments revealed that canagliflozin possesses the longest half-life of 3.1 h, while empagliflozin demonstrated the highest kidney concentration in normal mice after oral administration of the three SGLT2 inhibitors at a dosage of 3 mg/kg (Tahara et al., 2016). Furthermore, a cell-based study indicated that among the three drugs, dapagliflozin exhibited the lowest IC_50_ concentration, at 1 nM (Wright, 2021). In terms of immunity, different SGLT2 inhibitors have varying effects on T and B cells, as mentioned in our earlier discussion.

This study did not establish a definitive correlation between SGLT2 inhibitors and MN, potentially due to the specific population and off-target effects. Fortunately, the study identified 14 differentially expressed genes related to the pathogenesis of MN, opening the door for future investigations into whether SGLT2 inhibitors can treat MN through these genes. However, this study did have some limitations. The datasets used in this study were primarily sourced from European populations, and further exploration is needed to determine the generalizability of the results to other populations. Additionally, the study predicted the targeting effect of specific drug targets but did not estimate the potential off-target effects. Despite various sensitivity analyses conducted to test the assumptions of MR, the possibility of horizontal pleiotropy could not be entirely ruled out.

## 5 Conclusion

Our study indicated that dapagliflozin might have a therapeutic effect on IgAN by influencing relevant immune signaling pathways through the LCN2 and AGER genes.

## Data Availability

The original contributions presented in the study are included in the article/Supplementary Material, further inquiries can be directed to the corresponding author.
