# Supplementary material for: Pharmacological targets of SGLT2 inhibitors on IgA nephropathy and membranous nephropathy: a mendelian randomization study
Source: Front Pharmacol. 2024 May 22;15:1399881. doi: 10.3389/fphar.2024.1399881 (PMC11155304; doi:10.3389/fphar.2024.1399881)
Supplement: Supplementary file 1 [file DataSheet2.PDF]

Pharmacological targets of SGLT2 inhibitors on IgA nephropathy and membranous nephropathy: a Mendelian randomization study

Supplementary Tables Contents:

Supplementary Table 1 Univariate Mendelian randomization assessment of the causal influence of drug targets on membranous nephropathy ....… P.1-6

Supplementary Table 2 Results of heterogeneity test for the causal influence of drug targets on membranous nephropathy ……………………………………………….. P.7-9

Supplementary Table 3 Results of horizontal pleiotropy for the causal influence of 49 drug targets on membranous nephropathy in MR-Egger intercept test ……….. P.10-11

Supplementary Table 4 Results of directionality analysis of the causal influence of 16 drug targets on membranous nephropathy in Steiger test ………………………… P.12

Supplementary Table 5 Univariate MR assessment of the causal influence of drug targets on IgA nephropathy …………………………………………………… P.13-19

Supplementary Table 6 Results of heterogeneity test for the causal influence of drug targets on IgA nephropathy …………………………………………………… P.20-23

Supplementary Table 7 Results of horizontal pleiotropy analysis for the causal influence of 43 drug targets on IgA nephropathy using MR-Egger intercept test ……………………………………………………………………….. P.24-25

Supplementary Table 8 Results of directionality analysis for the causal influence of 8 drug targets on IgA nephropathy using Steiger test ……………………………… P.26

Supplementary Table 9 Target genes corresponding to canagliflozin, dapagliflozin and empagliflozin ……………………………………………………………...P.27-38

Supplementary Table 1

Outcome	Exposure	Method	SNP Number	Beta	SE	*P* Value	OR
Membranous nephropathy || id:ebi-a-GCST010005	|| id:eqtl-a-ENSG00000026025	MR Egger	25	-0.477241731	0.201707524	0.027	0.620492519
Membranous nephropathy || id:ebi-a-GCST010005	|| id:eqtl-a-ENSG00000026025	Weighted median	25	-0.247280773	0.111217006	0.026	0.780921401
Membranous nephropathy || id:ebi-a-GCST010005	|| id:eqtl-a-ENSG00000026025	Inverse variance weighted (fixed effects)	25	-0.211060545	0.075618788	0.005	0.80972504
Membranous nephropathy || id:ebi-a-GCST010005	|| id:eqtl-a-ENSG00000026025	Simple mode	25	-0.205171666	0.20163577	0.319	0.814507482
Membranous nephropathy || id:ebi-a-GCST010005	|| id:eqtl-a-ENSG00000026025	Weighted mode	25	-0.330374712	0.151106926	0.039	0.718654394
Membranous nephropathy || id:ebi-a-GCST010005	|| id:eqtl-a-ENSG00000039068	MR Egger	49	0.034140784	0.075945882	0.655	1.03473027
Membranous nephropathy || id:ebi-a-GCST010005	|| id:eqtl-a-ENSG00000039068	Weighted median	49	0.057437856	0.046162958	0.213	1.059119451
Membranous nephropathy || id:ebi-a-GCST010005	|| id:eqtl-a-ENSG00000039068	Inverse variance weighted (fixed effects)	49	0.093307483	0.036191124	0.01	1.097799238
Membranous nephropathy || id:ebi-a-GCST010005	|| id:eqtl-a-ENSG00000039068	Simple mode	49	0.088549078	0.067890551	0.198	1.092587874
Membranous nephropathy || id:ebi-a-GCST010005	|| id:eqtl-a-ENSG00000039068	Weighted mode	49	0.060202047	0.047445684	0.211	1.062051109
Membranous nephropathy || id:ebi-a-GCST010005	|| id:eqtl-a-ENSG00000073756	MR Egger	27	0.100107729	0.054266738	0.077	1.105289984
Membranous nephropathy || id:ebi-a-GCST010005	|| id:eqtl-a-ENSG00000073756	Weighted median	27	0.110417258	0.045224954	0.015	1.116743944
Membranous nephropathy || id:ebi-a-GCST010005	|| id:eqtl-a-ENSG00000073756	Inverse variance weighted (fixed effects)	27	0.082782658	0.035245363	0.019	1.086305683
Membranous nephropathy || id:ebi-a-GCST010005	|| id:eqtl-a-ENSG00000073756	Simple mode	27	0.115321259	0.066793871	0.096	1.122233908

Membranous nephropathy || id:ebi-a-GCST010005	|| id:eqtl-a-ENSG00000073756	Weighted mode	27	0.124022328	0.060162435	0.049	1.132041146
Membranous nephropathy || id:ebi-a-GCST010005	|| id:eqtl-a-ENSG00000096968	MR Egger	26	0.309657509	0.094402575	0.003	1.362958233
Membranous nephropathy || id:ebi-a-GCST010005	|| id:eqtl-a-ENSG00000096968	Weighted median	26	0.239843861	0.044498027	0	1.271050674
Membranous nephropathy || id:ebi-a-GCST010005	|| id:eqtl-a-ENSG00000096968	Inverse variance weighted (fixed effects)	26	0.196395769	0.034194103	0	1.217008465
Membranous nephropathy || id:ebi-a-GCST010005	|| id:eqtl-a-ENSG00000096968	Simple mode	26	0.244532801	0.065912999	0.001	1.277024549
Membranous nephropathy || id:ebi-a-GCST010005	|| id:eqtl-a-ENSG00000096968	Weighted mode	26	0.240318441	0.063657155	0.001	1.271654032
Membranous nephropathy || id:ebi-a-GCST010005	|| id:eqtl-a-ENSG00000107796	MR Egger	51	-0.096479363	0.033419415	0.006	0.908028636
Membranous nephropathy || id:ebi-a-GCST010005	|| id:eqtl-a-ENSG00000107796	Weighted median	51	-0.136429186	0.035014252	0	0.872468101
Membranous nephropathy || id:ebi-a-GCST010005	|| id:eqtl-a-ENSG00000107796	Inverse variance weighted (fixed effects)	51	-0.131742811	0.022045237	0	0.876566409
Membranous nephropathy || id:ebi-a-GCST010005	|| id:eqtl-a-ENSG00000107796	Simple mode	51	-0.111205988	0.086545598	0.205	0.894754422
Membranous nephropathy || id:ebi-a-GCST010005	|| id:eqtl-a-ENSG00000107796	Weighted mode	51	-0.081997852	0.027782869	0.005	0.921273937
Membranous nephropathy || id:ebi-a-GCST010005	|| id:eqtl-a-ENSG00000118217	MR Egger	45	-0.078231909	0.027204149	0.006	0.924749944
Membranous nephropathy || id:ebi-a-GCST010005	|| id:eqtl-a-ENSG00000118217	Weighted median	45	-0.045696298	0.023734817	0.054	0.955332054
Membranous nephropathy || id:ebi-a-GCST010005	|| id:eqtl-a-ENSG00000118217	Inverse variance weighted (fixed effects)	45	-0.090200438	0.017795513	0	0.913748017
Membranous nephropathy || id:ebi-a-GCST010005	|| id:eqtl-a-ENSG000001	Simple mode	45	-0.039940309	0.036541121	0.28	0.960846791

	18217						
Membranous nephropathy || id:ebi-a-GCST010005	|| id:eqtl-a-ENSG000001 18217	Weighted mode	45	-0.059226107	0.029073805	0.048	0.942493641
Membranous nephropathy || id:ebi-a-GCST010005	|| id:eqtl-a-ENSG000001 25538	MR Egger	21	0.321761119	0.560793778	0.573	1.379555187
Membranous nephropathy || id:ebi-a-GCST010005	|| id:eqtl-a-ENSG000001 25538	Weighted median	21	-0.520752353	0.154281979	0.001	0.594073427
Membranous nephropathy || id:ebi-a-GCST010005	|| id:eqtl-a-ENSG000001 25538	Inverse variance weighted (multiplicative random effects)	21	-0.559293248	0.126236557	0	0.57161291
Membranous nephropathy || id:ebi-a-GCST010005	|| id:eqtl-a-ENSG000001 25538	Simple mode	21	-1.004185451	0.299901267	0.003	0.366342917
Membranous nephropathy || id:ebi-a-GCST010005	|| id:eqtl-a-ENSG000001 25538	Weighted mode	21	-0.382769586	0.255359207	0.15	0.681970017
Membranous nephropathy || id:ebi-a-GCST010005	|| id:eqtl-a-ENSG000001 35218	MR Egger	37	-0.05572878	0.03566104	0.127	0.94579562
Membranous nephropathy || id:ebi-a-GCST010005	|| id:eqtl-a-ENSG000001 35218	Weighted median	37	-0.031617769	0.025805655	0.22	0.968876846
Membranous nephropathy || id:ebi-a-GCST010005	|| id:eqtl-a-ENSG000001 35218	Inverse variance weighted (fixed effects)	37	-0.039761352	0.019329695	0.04	0.961018757
Membranous nephropathy || id:ebi-a-GCST010005	|| id:eqtl-a-ENSG000001 35218	Simple mode	37	-0.027767324	0.035122204	0.434	0.972614644
Membranous nephropathy || id:ebi-a-GCST010005	|| id:eqtl-a-ENSG000001 35218	Weighted mode	37	-0.030940894	0.025588593	0.234	0.969532876
Membranous nephropathy || id:ebi-a-GCST010005	|| id:eqtl-a-ENSG000001 40941	MR Egger	21	0.140498984	0.071746331	0.065	1.150847911
Membranous nephropathy || id:ebi-a-GCST010005	|| id:eqtl-a-ENSG000001 40941	Weighted median	21	0.193289303	0.059044429	0.001	1.213233734
Membranous nephropathy || id:ebi-a-GCST010005	|| id:eqtl-a-ENSG000001 40941	Inverse variance weighted (fixed effects)	21	0.146124815	0.043643064	0.001	1.157340632
Membranous nephropathy ||	||	Simple mode	21	0.217865804	0.085471688	0.019	1.243420195

id:ebi-a-GCST010005	id:eqtl-a-ENSG00000140941						
Membranous nephropathy || id:ebi-a-GCST010005	|| id:eqtl-a-ENSG00000140941	Weighted mode	21	0.19618034	0.0660362	0.008	1.216746313
Membranous nephropathy || id:ebi-a-GCST010005	|| id:eqtl-a-ENSG00000171105	MR Egger	6	0.518558395	1.020512231	0.638	1.679604577
Membranous nephropathy || id:ebi-a-GCST010005	|| id:eqtl-a-ENSG00000171105	Weighted median	6	0.529580961	0.216181113	0.014	1.698220539
Membranous nephropathy || id:ebi-a-GCST010005	|| id:eqtl-a-ENSG00000171105	Inverse variance weighted (fixed effects)	6	0.436993533	0.187623273	0.02	1.548046065
Membranous nephropathy || id:ebi-a-GCST010005	|| id:eqtl-a-ENSG00000171105	Simple mode	6	0.551733075	0.345183482	0.171	1.736259479
Membranous nephropathy || id:ebi-a-GCST010005	|| id:eqtl-a-ENSG00000171105	Weighted mode	6	0.557607646	0.314645519	0.137	1.746489277
Membranous nephropathy || id:ebi-a-GCST010005	|| id:eqtl-a-ENSG00000171791	MR Egger	27	-7.980832938	5.062987015	0.128	0.000341954
Membranous nephropathy || id:ebi-a-GCST010005	|| id:eqtl-a-ENSG00000171791	Weighted median	27	0.176013492	0.159376275	0.269	1.192454148
Membranous nephropathy || id:ebi-a-GCST010005	|| id:eqtl-a-ENSG00000171791	Inverse variance weighted (multiplicative random effects)	27	2.223296388	0.607503989	0	9.237731879
Membranous nephropathy || id:ebi-a-GCST010005	|| id:eqtl-a-ENSG00000171791	Simple mode	27	0.066018439	0.141953941	0.646	1.068246414
Membranous nephropathy || id:ebi-a-GCST010005	|| id:eqtl-a-ENSG00000171791	Weighted mode	27	0.109763181	0.129101369	0.403	1.116013746
Membranous nephropathy || id:ebi-a-GCST010005	|| id:eqtl-a-ENSG00000175197	MR Egger	18	-0.017491362	0.240163205	0.943	0.982660724
Membranous nephropathy || id:ebi-a-GCST010005	|| id:eqtl-a-ENSG00000175197	Weighted median	18	0.141759015	0.115686214	0.22	1.152298928
Membranous nephropathy || id:ebi-a-GCST010005	|| id:eqtl-a-ENSG00000175197	Inverse variance weighted (fixed effects)	18	0.170266717	0.086472497	0.049	1.185621034

Membranous nephropathy || id:ebi-a-GCST010005	|| id:eqtl-a-ENSG00000175197	Simple mode	18	0.087669956	0.188430721	0.648	1.091627777
Membranous nephropathy || id:ebi-a-GCST010005	|| id:eqtl-a-ENSG00000175197	Weighted mode	18	0.129915766	0.122910255	0.305	1.138732459
Membranous nephropathy || id:ebi-a-GCST010005	|| id:eqtl-a-ENSG00000186951	MR Egger	13	1.189056169	0.747001866	0.14	3.283980221
Membranous nephropathy || id:ebi-a-GCST010005	|| id:eqtl-a-ENSG00000186951	Weighted median	13	0.627986423	0.171965064	0	1.873833669
Membranous nephropathy || id:ebi-a-GCST010005	|| id:eqtl-a-ENSG00000186951	Inverse variance weighted (fixed effects)	13	0.618037992	0.136140074	0	1.855284386
Membranous nephropathy || id:ebi-a-GCST010005	|| id:eqtl-a-ENSG00000186951	Simple mode	13	0.52806453	0.277375019	0.081	1.695647255
Membranous nephropathy || id:ebi-a-GCST010005	|| id:eqtl-a-ENSG00000186951	Weighted mode	13	0.72114309	0.266233637	0.019	2.056782956
Membranous nephropathy || id:ebi-a-GCST010005	|| id:eqtl-a-ENSG00000198408	MR Egger	24	-2.548599691	1.187615036	0.043	0.078191081
Membranous nephropathy || id:ebi-a-GCST010005	|| id:eqtl-a-ENSG00000198408	Weighted median	24	-1.102380669	0.147047064	0	0.33207957
Membranous nephropathy || id:ebi-a-GCST010005	|| id:eqtl-a-ENSG00000198408	Inverse variance weighted (fixed effects)	24	-1.103595329	0.112341897	0	0.331676452
Membranous nephropathy || id:ebi-a-GCST010005	|| id:eqtl-a-ENSG00000198408	Simple mode	24	-1.095861657	0.260041343	0	0.334251473
Membranous nephropathy || id:ebi-a-GCST010005	|| id:eqtl-a-ENSG00000198408	Weighted mode	24	-1.093838428	0.272412443	0.001	0.334928425
Membranous nephropathy || id:ebi-a-GCST010005	|| id:eqtl-a-ENSG00000198793	MR Egger	17	0.104368813	0.387460356	0.791	1.110009765
Membranous nephropathy || id:ebi-a-GCST010005	|| id:eqtl-a-ENSG00000198793	Weighted median	17	0.132514769	0.044109592	0.003	1.141695877
Membranous nephropathy || id:ebi-a-GCST010005	|| id:eqtl-a-ENSG000001	Inverse variance weighted (fixed effects)	17	0.155873016	0.035918755	0	1.16867779

	98793						
Membranous nephropathy || id:ebi-a-GCST010005	|| id:eqtl-a-ENSG00000198793	Simple mode	17	0.122624256	0.069826763	0.098	1.130459577
Membranous nephropathy || id:ebi-a-GCST010005	|| id:eqtl-a-ENSG00000198793	Weighted mode	17	0.123769488	0.073835927	0.113	1.131754958
Membranous nephropathy || id:ebi-a-GCST010005	|| id:eqtl-a-ENSG00000204305	MR Egger	106	2.372785492	1.111550369	0.035	10.72723133
Membranous nephropathy || id:ebi-a-GCST010005	|| id:eqtl-a-ENSG00000204305	Weighted median	106	2.25855658	0.146773329	0	9.56926672
Membranous nephropathy || id:ebi-a-GCST010005	|| id:eqtl-a-ENSG00000204305	Inverse variance weighted (multiplicative random effects)	106	4.087476439	0.291498891	0	59.58932453
Membranous nephropathy || id:ebi-a-GCST010005	|| id:eqtl-a-ENSG00000204305	Simple mode	106	6.699462583	0.467564901	0	811.9693416
Membranous nephropathy || id:ebi-a-GCST010005	|| id:eqtl-a-ENSG00000204305	Weighted mode	106	2.52093357	0.125385813	0	12.44020505

Abbreviation: SE: standard error, OR: odds ratio.

Supplementary Table 2

Outcome	Exposure	Method	Q	Q_df	Q_*P* vale
Membranous nephropathy || id:ebi-a-GCST010005	|| id:eqtl-a-ENSG00000005381	MR Egger	24.31034629	69	1
Membranous nephropathy || id:ebi-a-GCST010005	|| id:eqtl-a-ENSG00000005381	Inverse variance weighted	34.56214897	70	1
Membranous nephropathy || id:ebi-a-GCST010005	|| id:eqtl-a-ENSG00000026025	MR Egger	17.09286889	23	0.805
Membranous nephropathy || id:ebi-a-GCST010005	|| id:eqtl-a-ENSG00000026025	Inverse variance weighted	19.11909236	24	0.746
Membranous nephropathy || id:ebi-a-GCST010005	|| id:eqtl-a-ENSG00000039068	MR Egger	41.76687008	47	0.689
Membranous nephropathy || id:ebi-a-GCST010005	|| id:eqtl-a-ENSG00000039068	Inverse variance weighted	42.55213574	48	0.695
Membranous nephropathy || id:ebi-a-GCST010005	|| id:eqtl-a-ENSG00000044574	MR Egger	799.6630911	15	0
Membranous nephropathy || id:ebi-a-GCST010005	|| id:eqtl-a-ENSG00000044574	Inverse variance weighted	846.9997081	16	0
Membranous nephropathy || id:ebi-a-GCST010005	|| id:eqtl-a-ENSG00000067606	MR Egger	1.608729137	8	0.991
Membranous nephropathy || id:ebi-a-GCST010005	|| id:eqtl-a-ENSG00000067606	Inverse variance weighted	2.607209224	9	0.978
Membranous nephropathy || id:ebi-a-GCST010005	|| id:eqtl-a-ENSG00000072310	MR Egger	36.73385734	63	0.997
Membranous nephropathy || id:ebi-a-GCST010005	|| id:eqtl-a-ENSG00000072310	Inverse variance weighted	43.00465427	64	0.98
Membranous nephropathy || id:ebi-a-GCST010005	|| id:eqtl-a-ENSG00000073756	MR Egger	9.648372518	25	0.997
Membranous nephropathy || id:ebi-a-GCST010005	|| id:eqtl-a-ENSG00000073756	Inverse variance weighted	9.824661987	26	0.998
Membranous nephropathy || id:ebi-a-GCST010005	|| id:eqtl-a-ENSG00000087088	MR Egger	4.185593733	7	0.758
Membranous nephropathy || id:ebi-a-GCST010005	|| id:eqtl-a-ENSG00000087088	Inverse variance weighted	10.00127912	8	0.265
Membranous nephropathy || id:ebi-a-GCST010005	|| id:eqtl-a-ENSG00000096717	MR Egger	50.91939883	91	1
Membranous nephropathy || id:ebi-a-GCST010005	|| id:eqtl-a-ENSG00000096717	Inverse variance weighted	85.16541503	92	0.68
Membranous nephropathy || id:ebi-a-GCST010005	|| id:eqtl-a-ENSG00000096968	MR Egger	17.65818738	24	0.819
Membranous nephropathy || id:ebi-a-GCST010005	|| id:eqtl-a-ENSG00000096968	Inverse variance weighted	19.31502177	25	0.782
Membranous nephropathy || id:ebi-a-GCST010005	|| id:eqtl-a-ENSG00000100292	MR Egger	38.77812959	21	0.01
Membranous nephropathy || id:ebi-a-GCST010005	|| id:eqtl-a-ENSG00000100292	Inverse variance weighted	38.91143483	22	0.014
Membranous nephropathy || id:ebi-a-GCST010005	|| id:eqtl-a-ENSG00000107796	MR Egger	42.36313886	49	0.737
Membranous nephropathy || id:ebi-a-GCST010005	|| id:eqtl-a-ENSG00000107796	Inverse variance weighted	44.33425956	50	0.699
Membranous nephropathy || id:ebi-a-GCST010005	|| id:eqtl-a-ENSG00000109381	MR Egger	3.229119951	19	1
Membranous nephropathy || id:ebi-a-GCST010005	|| id:eqtl-a-ENSG00000109381	Inverse variance weighted	3.274442808	20	1
Membranous nephropathy || id:ebi-a-GCST010005	|| id:eqtl-a-ENSG00000109819	MR Egger	15.89803261	18	0.6
Membranous nephropathy || id:ebi-a-GCST010005	|| id:eqtl-a-ENSG00000109819	Inverse variance weighted	16.30579669	19	0.637
Membranous nephropathy || id:ebi-a-GCST010005	|| id:eqtl-a-ENSG00000112299	MR Egger	64.83463585	61	0.344
Membranous nephropathy || id:ebi-a-GCST010005	|| id:eqtl-a-ENSG00000112299	Inverse variance weighted	67.67168919	62	0.29
Membranous nephropathy || id:ebi-a-GCST010005	|| id:eqtl-a-ENSG00000113249	MR Egger	14.39238911	16	0.57
Membranous nephropathy || id:ebi-a-GCST010005	|| id:eqtl-a-ENSG00000113249	Inverse variance weighted	15.28451853	17	0.575
Membranous nephropathy || id:ebi-a-GCST010005	|| id:eqtl-a-ENSG00000116044	MR Egger	2.574846167	4	0.631
Membranous nephropathy || id:ebi-a-GCST010005	|| id:eqtl-a-ENSG00000116044	Inverse variance weighted	13.75702355	5	0.017
Membranous nephropathy || id:ebi-a-GCST010005	|| id:eqtl-a-ENSG00000118217	MR Egger	30.03420784	43	0.933
Membranous nephropathy || id:ebi-a-GCST010005	|| id:eqtl-a-ENSG00000118217	Inverse variance weighted	30.3725418	44	0.941
Membranous nephropathy || id:ebi-a-GCST010005	|| id:eqtl-a-ENSG00000121691	MR Egger	36.86777739	31	0.216
Membranous nephropathy || id:ebi-a-GCST010005	|| id:eqtl-a-ENSG00000121691	Inverse variance weighted	37.83453259	32	0.22
Membranous nephropathy || id:ebi-a-GCST010005	|| id:eqtl-a-ENSG00000125538	MR Egger	29.48869062	19	0.059
Membranous nephropathy || id:ebi-a-GCST010005	|| id:eqtl-a-ENSG00000125538	Inverse variance weighted	33.50827104	20	0.03
Membranous nephropathy || id:ebi-a-GCST010005	|| id:eqtl-a-ENSG00000126581	MR Egger	7.465965335	30	1
Membranous nephropathy || id:ebi-a-GCST010005	|| id:eqtl-a-ENSG00000126581	Inverse variance weighted	7.577228854	31	1

Membranous nephropathy || id:ebi-a-GCST010005	|| id:eqtl-a-ENSG00000135218	MR Egger	16.80487114	35	0.996
Membranous nephropathy || id:ebi-a-GCST010005	|| id:eqtl-a-ENSG00000135218	Inverse variance weighted	17.08876663	36	0.997
Membranous nephropathy || id:ebi-a-GCST010005	|| id:eqtl-a-ENSG00000136634	MR Egger	3.071146036	9	0.961
Membranous nephropathy || id:ebi-a-GCST010005	|| id:eqtl-a-ENSG00000136634	Inverse variance weighted	3.48260124	10	0.968
Membranous nephropathy || id:ebi-a-GCST010005	|| id:eqtl-a-ENSG00000137752	MR Egger	20.67330768	39	0.993
Membranous nephropathy || id:ebi-a-GCST010005	|| id:eqtl-a-ENSG00000137752	Inverse variance weighted	22.38489973	40	0.989
Membranous nephropathy || id:ebi-a-GCST010005	|| id:eqtl-a-ENSG00000140941	MR Egger	9.174769571	19	0.97
Membranous nephropathy || id:ebi-a-GCST010005	|| id:eqtl-a-ENSG00000140941	Inverse variance weighted	9.184529588	20	0.981
Membranous nephropathy || id:ebi-a-GCST010005	|| id:eqtl-a-ENSG00000141510	MR Egger	0.158240103	1	0.691
Membranous nephropathy || id:ebi-a-GCST010005	|| id:eqtl-a-ENSG00000141510	Inverse variance weighted	0.168716697	2	0.919
Membranous nephropathy || id:ebi-a-GCST010005	|| id:eqtl-a-ENSG00000142208	MR Egger	38.8584286	13	0
Membranous nephropathy || id:ebi-a-GCST010005	|| id:eqtl-a-ENSG00000142208	Inverse variance weighted	41.66249165	14	0
Membranous nephropathy || id:ebi-a-GCST010005	|| id:eqtl-a-ENSG00000145335	MR Egger	47.43974569	40	0.195
Membranous nephropathy || id:ebi-a-GCST010005	|| id:eqtl-a-ENSG00000145335	Inverse variance weighted	67.33833106	41	0.006
Membranous nephropathy || id:ebi-a-GCST010005	|| id:eqtl-a-ENSG00000147872	MR Egger	11.77883765	28	0.997
Membranous nephropathy || id:ebi-a-GCST010005	|| id:eqtl-a-ENSG00000147872	Inverse variance weighted	15.82718868	29	0.977
Membranous nephropathy || id:ebi-a-GCST010005	|| id:eqtl-a-ENSG00000148346	MR Egger	304.5492407	14	0
Membranous nephropathy || id:ebi-a-GCST010005	|| id:eqtl-a-ENSG00000148346	Inverse variance weighted	322.6636814	15	0
Membranous nephropathy || id:ebi-a-GCST010005	|| id:eqtl-a-ENSG00000154229	MR Egger	40.1313914	46	0.716
Membranous nephropathy || id:ebi-a-GCST010005	|| id:eqtl-a-ENSG00000154229	Inverse variance weighted	46.92854911	47	0.475
Membranous nephropathy || id:ebi-a-GCST010005	|| id:eqtl-a-ENSG00000161011	MR Egger	111.4534332	41	0
Membranous nephropathy || id:ebi-a-GCST010005	|| id:eqtl-a-ENSG00000161011	Inverse variance weighted	124.3016694	42	0
Membranous nephropathy || id:ebi-a-GCST010005	|| id:eqtl-a-ENSG00000162711	MR Egger	36.8172717	23	0.034
Membranous nephropathy || id:ebi-a-GCST010005	|| id:eqtl-a-ENSG00000162711	Inverse variance weighted	37.75166953	24	0.037
Membranous nephropathy || id:ebi-a-GCST010005	|| id:eqtl-a-ENSG00000164305	MR Egger	5.146183963	16	0.995
Membranous nephropathy || id:ebi-a-GCST010005	|| id:eqtl-a-ENSG00000164305	Inverse variance weighted	5.148202162	17	0.997
Membranous nephropathy || id:ebi-a-GCST010005	|| id:eqtl-a-ENSG00000165806	MR Egger	20.39169278	34	0.968
Membranous nephropathy || id:ebi-a-GCST010005	|| id:eqtl-a-ENSG00000165806	Inverse variance weighted	20.81060102	35	0.972
Membranous nephropathy || id:ebi-a-GCST010005	|| id:eqtl-a-ENSG00000168610	MR Egger	9.128969662	40	1
Membranous nephropathy || id:ebi-a-GCST010005	|| id:eqtl-a-ENSG00000168610	Inverse variance weighted	9.276306552	41	1
Membranous nephropathy || id:ebi-a-GCST010005	|| id:eqtl-a-ENSG00000169710	MR Egger	2.771099309	14	0.999
Membranous nephropathy || id:ebi-a-GCST010005	|| id:eqtl-a-ENSG00000169710	Inverse variance weighted	7.715197715	15	0.935
Membranous nephropathy || id:ebi-a-GCST010005	|| id:eqtl-a-ENSG00000171105	MR Egger	0.695416511	4	0.952
Membranous nephropathy || id:ebi-a-GCST010005	|| id:eqtl-a-ENSG00000171105	Inverse variance weighted	0.702028064	5	0.983
Membranous nephropathy || id:ebi-a-GCST010005	|| id:eqtl-a-ENSG00000171791	MR Egger	794.8743649	25	0
Membranous nephropathy || id:ebi-a-GCST010005	|| id:eqtl-a-ENSG00000171791	Inverse variance weighted	925.7071947	26	0
Membranous nephropathy || id:ebi-a-GCST010005	|| id:eqtl-a-ENSG00000172071	MR Egger	4.38426984	16	0.998
Membranous nephropathy || id:ebi-a-GCST010005	|| id:eqtl-a-ENSG00000172071	Inverse variance weighted	5.766369464	17	0.995
Membranous nephropathy || id:ebi-a-GCST010005	|| id:eqtl-a-ENSG00000173039	MR Egger	1.66E-05	1	0.997
Membranous nephropathy || id:ebi-a-GCST010005	|| id:eqtl-a-ENSG00000173039	Inverse variance weighted	0.018106776	2	0.991
Membranous nephropathy || id:ebi-a-GCST010005	|| id:eqtl-a-ENSG00000175197	MR Egger	16.47449617	16	0.42
Membranous nephropathy || id:ebi-a-GCST010005	|| id:eqtl-a-ENSG00000175197	Inverse variance weighted	17.20077072	17	0.441
Membranous nephropathy || id:ebi-a-GCST010005	|| id:eqtl-a-ENSG00000185532	MR Egger	5.435361318	10	0.86
Membranous nephropathy || id:ebi-a-GCST010005	|| id:eqtl-a-ENSG00000185532	Inverse variance weighted	10.89527767	11	0.452

Membranous nephropathy || id:ebi-a-GCST010005	|| id:eqtl-a-ENSG00000186951	MR Egger	1.381545457	11	1
Membranous nephropathy || id:ebi-a-GCST010005	|| id:eqtl-a-ENSG00000186951	Inverse variance weighted	1.985948153	12	0.999
Membranous nephropathy || id:ebi-a-GCST010005	|| id:eqtl-a-ENSG00000189403	MR Egger	0.052247478	1	0.819
Membranous nephropathy || id:ebi-a-GCST010005	|| id:eqtl-a-ENSG00000189403	Inverse variance weighted	0.219161744	2	0.896
Membranous nephropathy || id:ebi-a-GCST010005	|| id:eqtl-a-ENSG00000198408	MR Egger	0.612181384	22	1
Membranous nephropathy || id:ebi-a-GCST010005	|| id:eqtl-a-ENSG00000198408	Inverse variance weighted	2.105974847	23	1
Membranous nephropathy || id:ebi-a-GCST010005	|| id:eqtl-a-ENSG00000198793	MR Egger	1.696351616	15	1
Membranous nephropathy || id:ebi-a-GCST010005	|| id:eqtl-a-ENSG00000198793	Inverse variance weighted	1.71417455	16	1
Membranous nephropathy || id:ebi-a-GCST010005	|| id:eqtl-a-ENSG00000204305	MR Egger	6387.578081	104	0
Membranous nephropathy || id:ebi-a-GCST010005	|| id:eqtl-a-ENSG00000204305	Inverse variance weighted	6544.359088	105	0
Membranous nephropathy || id:ebi-a-GCST010005	|| id:eqtl-a-ENSG00000232810	MR Egger	322.9040942	83	0
Membranous nephropathy || id:ebi-a-GCST010005	|| id:eqtl-a-ENSG00000232810	Inverse variance weighted	548.0984285	84	0

Abbreviation: Q:Chi-Square Test, Q_df: Quantile Degrees of Freedom.

Supplementary Table 3

Outcome	Exposure	Egger_intercept	SE	*P* vale	Judge
Membranous nephropathy || id:ebi-a-GCST010005	|| id:eqtl-a-ENSG00000005381	-0.047380721	0.01479795	0.002066036	YES
Membranous nephropathy || id:ebi-a-GCST010005	|| id:eqtl-a-ENSG00000026025	0.035752494	0.025116705	0.168029058	NO
Membranous nephropathy || id:ebi-a-GCST010005	|| id:eqtl-a-ENSG00000039068	0.014112326	0.015925398	0.380047299	NO
Membranous nephropathy || id:ebi-a-GCST010005	|| id:eqtl-a-ENSG00000044574	-0.325140864	0.345049056	0.36096741	NO
Membranous nephropathy || id:ebi-a-GCST010005	|| id:eqtl-a-ENSG00000067606	-0.055016656	0.055058514	0.346939714	NO
Membranous nephropathy || id:ebi-a-GCST010005	|| id:eqtl-a-ENSG00000072310	0.035095227	0.014014793	0.014875886	YES
Membranous nephropathy || id:ebi-a-GCST010005	|| id:eqtl-a-ENSG00000073756	-0.006970388	0.016601364	0.678168123	NO
Membranous nephropathy || id:ebi-a-GCST010005	|| id:eqtl-a-ENSG00000087088	0.081196989	0.033669718	0.046666046	YES
Membranous nephropathy || id:ebi-a-GCST010005	|| id:eqtl-a-ENSG00000096717	0.085415155	0.014595867	7.57E-08	YES
Membranous nephropathy || id:ebi-a-GCST010005	|| id:eqtl-a-ENSG00000096968	-0.031066224	0.02413509	0.210307271	NO
Membranous nephropathy || id:ebi-a-GCST010005	|| id:eqtl-a-ENSG00000100292	-0.00728967	0.027131138	0.790795695	NO
Membranous nephropathy || id:ebi-a-GCST010005	|| id:eqtl-a-ENSG00000107796	-0.019225499	0.013693707	0.166635524	NO
Membranous nephropathy || id:ebi-a-GCST010005	|| id:eqtl-a-ENSG00000109381	0.005712702	0.026833845	0.833678993	NO
Membranous nephropathy || id:ebi-a-GCST010005	|| id:eqtl-a-ENSG00000109819	0.011705316	0.018330684	0.531148277	NO
Membranous nephropathy || id:ebi-a-GCST010005	|| id:eqtl-a-ENSG00000112299	0.020013126	0.012249541	0.107455113	NO
Membranous nephropathy || id:ebi-a-GCST010005	|| id:eqtl-a-ENSG00000113249	-0.051147596	0.054151601	0.358946805	NO
Membranous nephropathy || id:ebi-a-GCST010005	|| id:eqtl-a-ENSG00000116044	-0.256891705	0.076822227	0.028729284	YES
Membranous nephropathy || id:ebi-a-GCST010005	|| id:eqtl-a-ENSG00000118217	-0.008226719	0.014143401	0.563829573	NO
Membranous nephropathy || id:ebi-a-GCST010005	|| id:eqtl-a-ENSG00000121691	-0.016658856	0.018476919	0.374219405	NO
Membranous nephropathy || id:ebi-a-GCST010005	|| id:eqtl-a-ENSG00000125538	-0.088363912	0.054907986	0.124038392	NO
Membranous nephropathy || id:ebi-a-GCST010005	|| id:eqtl-a-ENSG00000126581	0.006419482	0.019245252	0.741030494	NO
Membranous nephropathy || id:ebi-a-GCST010005	|| id:eqtl-a-ENSG00000135218	0.008253463	0.015490198	0.597526297	NO
Membranous nephropathy || id:ebi-a-GCST010005	|| id:eqtl-a-ENSG00000136634	-0.024668721	0.038457882	0.537223102	NO
Membranous nephropathy || id:ebi-a-GCST010005	|| id:eqtl-a-ENSG00000137752	0.033915401	0.02592369	0.198437321	NO
Membranous nephropathy || id:ebi-a-GCST010005	|| id:eqtl-a-ENSG00000140941	0.001755691	0.017771445	0.922337646	NO
Membranous nephropathy || id:ebi-a-GCST010005	|| id:eqtl-a-ENSG00000141510	0.010932059	0.106805084	0.935064771	NO
Membranous nephropathy || id:ebi-a-GCST010005	|| id:eqtl-a-ENSG00000142208	0.04068467	0.042005664	0.350443961	NO
Membranous nephropathy || id:ebi-a-GCST010005	|| id:eqtl-a-ENSG00000145335	0.066811931	0.016311132	0.000198858	YES
Membranous nephropathy || id:ebi-a-GCST010005	|| id:eqtl-a-ENSG00000147872	0.030605832	0.015211257	0.05392543	NO
Membranous nephropathy || id:ebi-a-GCST010005	|| id:eqtl-a-ENSG00000148346	-0.289484489	0.317232429	0.376935736	NO
Membranous nephropathy || id:ebi-a-GCST010005	|| id:eqtl-a-ENSG00000154229	0.041522031	0.015926301	0.012267476	YES
Membranous nephropathy || id:ebi-a-GCST010005	|| id:eqtl-a-ENSG00000161011	0.07011779	0.032252342	0.035529047	YES
Membranous nephropathy || id:ebi-a-GCST010005	|| id:eqtl-a-ENSG00000162711	0.020004222	0.026182893	0.452623282	NO
Membranous nephropathy || id:ebi-a-GCST010005	|| id:eqtl-a-ENSG00000164305	-0.001705708	0.037968425	0.964723505	NO
Membranous nephropathy || id:ebi-a-GCST010005	|| id:eqtl-a-ENSG00000165806	-0.009110118	0.014075523	0.521828573	NO
Membranous nephropathy || id:ebi-a-GCST010005	|| id:eqtl-a-ENSG00000168610	-0.00962078	0.025064241	0.703126264	NO
Membranous nephropathy || id:ebi-a-GCST010005	|| id:eqtl-a-ENSG00000169710	-0.067553434	0.030381127	0.043151419	YES
Membranous nephropathy || id:ebi-a-GCST010005	|| id:eqtl-a-ENSG00000171105	-0.011280833	0.138736088	0.939100263	NO
Membranous nephropathy || id:ebi-a-GCST010005	|| id:eqtl-a-ENSG00000171791	0.860759289	0.424328881	0.05328674	NO
Membranous nephropathy || id:ebi-a-GCST010005	|| id:eqtl-a-ENSG00000172071	-0.074720355	0.063557858	0.256941623	NO
Membranous nephropathy || id:ebi-a-GCST010005	|| id:eqtl-a-ENSG00000173039	0.081633706	0.606943418	0.914885625	NO
Membranous nephropathy || id:ebi-a-GCST010005	|| id:eqtl-a-ENSG00000175197	0.038420973	0.045747148	0.413363734	NO

Membranous nephropathy || id:ebi-a-GCST010005	|| id:eqtl-a-ENSG00000185532	-0.080687692	0.034531409	0.04157657	YES
Membranous nephropathy || id:ebi-a-GCST010005	|| id:eqtl-a-ENSG00000186951	-0.073947374	0.09511731	0.453298911	NO
Membranous nephropathy || id:ebi-a-GCST010005	|| id:eqtl-a-ENSG00000189403	1.21572456	2.975695323	0.753082891	NO
Membranous nephropathy || id:ebi-a-GCST010005	|| id:eqtl-a-ENSG00000198408	0.213235528	0.1744674	0.234562603	NO
Membranous nephropathy || id:ebi-a-GCST010005	|| id:eqtl-a-ENSG00000198793	0.017454883	0.130745677	0.89557057	NO
Membranous nephropathy || id:ebi-a-GCST010005	|| id:eqtl-a-ENSG00000204305	0.271853575	0.170153061	0.113143742	NO
Membranous nephropathy || id:ebi-a-GCST010005	|| id:eqtl-a-ENSG00000232810	-0.330992066	0.043504741	3.90E-11	YES

Abbreviation: SE: standard error.

Supplementary Table 4

Exposure	Outcome	SNP_R2.exposure	SNP_R2.outcome	Correct_Causal_Direction	Steiger_*P* vale
|| id:eqtl-a-ENSG00000026025	Membranous nephropathy || id:ebi-a-GCST010005	0.158280732	0.003371414	TRUE	2.71E-146
|| id:eqtl-a-ENSG00000039068	Membranous nephropathy || id:ebi-a-GCST010005	0.311937088	0.006168138	TRUE	0
|| id:eqtl-a-ENSG00000073756	Membranous nephropathy || id:ebi-a-GCST010005	0.33856899	0.00192267	TRUE	0
|| id:eqtl-a-ENSG00000096968	Membranous nephropathy || id:ebi-a-GCST010005	0.35456119	0.006555958	TRUE	0
|| id:eqtl-a-ENSG00000107796	Membranous nephropathy || id:ebi-a-GCST010005	0.398817088	0.010035715	TRUE	0
|| id:eqtl-a-ENSG00000118217	Membranous nephropathy || id:ebi-a-GCST010005	0.695750438	0.007024926	TRUE	0
|| id:eqtl-a-ENSG00000125538	Membranous nephropathy || id:ebi-a-GCST010005	0.04109612	0.008319733	TRUE	9.21E-20
|| id:eqtl-a-ENSG00000135218	Membranous nephropathy || id:ebi-a-GCST010005	0.512882173	0.002672729	TRUE	0
|| id:eqtl-a-ENSG00000140941	Membranous nephropathy || id:ebi-a-GCST010005	0.435435712	0.002558605	TRUE	0
|| id:eqtl-a-ENSG00000171105	Membranous nephropathy || id:ebi-a-GCST010005	0.011765012	0.000767629	TRUE	1.08E-10
|| id:eqtl-a-ENSG00000171791	Membranous nephropathy || id:ebi-a-GCST010005	0.040363958	0.174395926	FALSE	8.42E-82
|| id:eqtl-a-ENSG00000175197	Membranous nephropathy || id:ebi-a-GCST010005	0.052077193	0.002641668	TRUE	4.94E-47
|| id:eqtl-a-ENSG00000186951	Membranous nephropathy || id:ebi-a-GCST010005	0.020196234	0.002834553	TRUE	1.01E-12
|| id:eqtl-a-ENSG00000198408	Membranous nephropathy || id:ebi-a-GCST010005	0.026195769	0.012355681	TRUE	3.94E-05
|| id:eqtl-a-ENSG00000198793	Membranous nephropathy || id:ebi-a-GCST010005	0.323273383	0.002576538	TRUE	0
|| id:eqtl-a-ENSG00000204305	Membranous nephropathy || id:ebi-a-GCST010005	1.133725611	2.313019656	FALSE	NA

Supplementary Table 5

Outcome	Exposure	Method	SNP Number	Beta	SE	P value	OR
IgA nephropathy || id:ieu-a-1081	|| id:eqtl-a-ENSG00000005381	MR Egger	25	-0.140567376	0.138958754	0.322	0.868865122
IgA nephropathy || id:ieu-a-1081	|| id:eqtl-a-ENSG00000005381	Weighted median	25	-0.242276047	0.088248991	0.006	0.784839495
IgA nephropathy || id:ieu-a-1081	|| id:eqtl-a-ENSG00000005381	Inverse variance weighted (fixed effects)	25	-0.311294053	0.066920036	0	0.732498451
IgA nephropathy || id:ieu-a-1081	|| id:eqtl-a-ENSG00000005381	Simple mode	25	-0.447139088	0.14732663	0.006	0.639454961
IgA nephropathy || id:ieu-a-1081	|| id:eqtl-a-ENSG00000005381	Weighted mode	25	-0.205763592	0.112765964	0.081	0.814025496
IgA nephropathy || id:ieu-a-1081	|| id:eqtl-a-ENSG00000026025	MR Egger	7	0.566801255	0.861477761	0.54	1.762619854
IgA nephropathy || id:ieu-a-1081	|| id:eqtl-a-ENSG00000026025	Weighted median	7	-0.135320221	0.245667778	0.582	0.873436174
IgA nephropathy || id:ieu-a-1081	|| id:eqtl-a-ENSG00000026025	Inverse variance weighted (multiplicative random effects)	7	-0.068045839	0.332376364	0.838	0.934217649
IgA nephropathy || id:ieu-a-1081	|| id:eqtl-a-ENSG00000026025	Simple mode	7	0.079036873	0.303746487	0.803	1.082244227
IgA nephropathy || id:ieu-a-1081	|| id:eqtl-a-ENSG00000026025	Weighted mode	7	-0.01624455	0.238331153	0.948	0.983886681
IgA nephropathy || id:ieu-a-1081	|| id:eqtl-a-ENSG00000039068	MR Egger	14	-0.050589082	0.238039719	0.835	0.950669237
IgA nephropathy || id:ieu-a-1081	|| id:eqtl-a-ENSG00000039068	Weighted median	14	-0.221984048	0.164079522	0.176	0.800928141
IgA nephropathy || id:ieu-a-1081	|| id:eqtl-a-ENSG00000039068	Inverse variance weighted (fixed effects)	14	-0.127203937	0.128315172	0.322	0.880554077
IgA nephropathy || id:ieu-a-1081	|| id:eqtl-a-ENSG00000039068	Simple mode	14	-0.206345605	0.234441142	0.395	0.81355186
IgA nephropathy || id:ieu-a-1081	|| id:eqtl-a-ENSG00000039068	Weighted mode	14	-0.18559493	0.164963423	0.281	0.830609982
IgA nephropathy || id:ieu-a-1081	|| id:eqtl-a-ENSG00000044574	MR Egger	3	-1.087260171	1.366482464	0.572	0.337138932
IgA nephropathy || id:ieu-a-1081	|| id:eqtl-a-ENSG00000044574	Weighted median	3	-0.152831026	0.593936941	0.797	0.858274736
IgA nephropathy || id:ieu-a-1081	|| id:eqtl-a-ENSG00000044574	Inverse variance weighted (fixed effects)	3	-0.122149069	0.515979351	0.813	0.88501643
IgA nephropathy || id:ieu-a-1081	|| id:eqtl-a-ENSG00000044574	Simple mode	3	0.100771741	0.776226147	0.909	1.106024153
IgA nephropathy || id:ieu-a-1081	|| id:eqtl-a-ENSG00000044574	Weighted mode	3	-0.356267042	0.798206905	0.699	0.700285589
IgA nephropathy || id:ieu-a-1081	|| id:eqtl-a-ENSG00000067606	MR Egger	6	0.304091063	0.826917815	0.732	1.355392476
IgA nephropathy || id:ieu-a-1081	|| id:eqtl-a-ENSG00000067606	Weighted median	6	0.026694631	0.266158806	0.92	1.027054125
IgA nephropathy || id:ieu-a-1081	|| id:eqtl-a-ENSG00000067606	Inverse variance weighted (fixed effects)	6	0.269668694	0.20203903	0.182	1.309530524
IgA nephropathy || id:ieu-a-1081	|| id:eqtl-a-ENSG00000067606	Simple mode	6	-0.05561006	0.382387602	0.89	0.945907911
IgA nephropathy || id:ieu-a-1081	|| id:eqtl-a-ENSG00000067606	Weighted mode	6	-0.043010885	0.38437206	0.915	0.957900964
IgA nephropathy || id:ieu-a-1081	|| id:eqtl-a-ENSG00000072310	MR Egger	26	0.096278885	0.11915095	0.427	1.101066092
IgA nephropathy || id:ieu-a-1081	|| id:eqtl-a-ENSG00000072310	Weighted median	26	-0.082979205	0.063673482	0.193	0.920370286
IgA nephropathy || id:ieu-a-1081	|| id:eqtl-a-ENSG00000072310	Inverse variance weighted (fixed effects)	26	-0.135702232	0.050192707	0.007	0.873102576
IgA nephropathy || id:ieu-a-1081	|| id:eqtl-a-ENSG00000072310	Simple mode	26	-0.073437469	0.083531082	0.388	0.929194248
IgA nephropathy || id:ieu-a-1081	|| id:eqtl-a-ENSG00000072310	Weighted mode	26	-0.069273824	0.069174534	0.326	0.933071148
IgA nephropathy || id:ieu-a-1081	|| id:eqtl-a-ENSG00000073756	MR Egger	10	0.329964397	0.350516926	0.374	1.390918607
IgA nephropathy || id:ieu-a-1081	|| id:eqtl-a-ENSG00000073756	Weighted median	10	0.15800086	0.167335484	0.345	1.171167202
IgA nephropathy || id:ieu-a-1081	|| id:eqtl-a-ENSG00000073756	Inverse variance weighted (fixed effects)	10	0.163938608	0.135551784	0.227	1.178141985
IgA nephropathy || id:ieu-a-1081	|| id:eqtl-a-ENSG00000073756	Simple mode	10	0.152213923	0.207751839	0.482	1.164409303

IgA nephropathy || id:ieu-a-1081	|| id:eqtl-a-ENSG00000073756	Weighted mode	10	0.186257729	0.191356225	0.356	1.204732715
IgA nephropathy || id:ieu-a-1081	|| id:eqtl-a-ENSG00000087088	MR Egger	4	-2.72981732	2.630435703	0.408	0.065231205
IgA nephropathy || id:ieu-a-1081	|| id:eqtl-a-ENSG00000087088	Weighted median	4	0.142255702	0.566136551	0.802	1.152871402
IgA nephropathy || id:ieu-a-1081	|| id:eqtl-a-ENSG00000087088	Inverse variance weighted (fixed effects)	4	0.069403807	0.486062771	0.886	1.071868949
IgA nephropathy || id:ieu-a-1081	|| id:eqtl-a-ENSG00000087088	Simple mode	4	0.661566384	0.867683917	0.501	1.937825338
IgA nephropathy || id:ieu-a-1081	|| id:eqtl-a-ENSG00000087088	Weighted mode	4	0.63140088	0.865028788	0.518	1.88024273
IgA nephropathy || id:ieu-a-1081	|| id:eqtl-a-ENSG00000096717	MR Egger	31	0.160400005	0.1004667	0.121	1.173980375
IgA nephropathy || id:ieu-a-1081	|| id:eqtl-a-ENSG00000096717	Weighted median	31	0.01097378	0.05744403	0.848	1.011034213
IgA nephropathy || id:ieu-a-1081	|| id:eqtl-a-ENSG00000096717	Inverse variance weighted (fixed effects)	31	0.030870568	0.043702664	0.48	1.031352005
IgA nephropathy || id:ieu-a-1081	|| id:eqtl-a-ENSG00000096717	Simple mode	31	0.048239814	0.070979457	0.502	1.049422291
IgA nephropathy || id:ieu-a-1081	|| id:eqtl-a-ENSG00000096717	Weighted mode	31	0.029241148	0.055552419	0.603	1.029672868
IgA nephropathy || id:ieu-a-1081	|| id:eqtl-a-ENSG00000096968	MR Egger	11	0.509412022	0.240439623	0.063	1.664312328
IgA nephropathy || id:ieu-a-1081	|| id:eqtl-a-ENSG00000096968	Weighted median	11	0.43357276	0.162744456	0.008	1.542759598
IgA nephropathy || id:ieu-a-1081	|| id:eqtl-a-ENSG00000096968	Inverse variance weighted (fixed effects)	11	0.328562225	0.123976327	0.008	1.388969666
IgA nephropathy || id:ieu-a-1081	|| id:eqtl-a-ENSG00000096968	Simple mode	11	0.414803943	0.256060747	0.136	1.514073866
IgA nephropathy || id:ieu-a-1081	|| id:eqtl-a-ENSG00000096968	Weighted mode	11	0.450622445	0.174225186	0.027	1.569288677
IgA nephropathy || id:ieu-a-1081	|| id:eqtl-a-ENSG00000100292	MR Egger	9	0.048058686	0.43732219	0.916	1.049232229
IgA nephropathy || id:ieu-a-1081	|| id:eqtl-a-ENSG00000100292	Weighted median	9	0.095990403	0.228038551	0.674	1.1007485
IgA nephropathy || id:ieu-a-1081	|| id:eqtl-a-ENSG00000100292	Inverse variance weighted (fixed effects)	9	0.199334086	0.182315255	0.274	1.220589679
IgA nephropathy || id:ieu-a-1081	|| id:eqtl-a-ENSG00000100292	Simple mode	9	0.162457331	0.395365846	0.692	1.176398121
IgA nephropathy || id:ieu-a-1081	|| id:eqtl-a-ENSG00000100292	Weighted mode	9	0.107666135	0.236784903	0.661	1.113675866
IgA nephropathy || id:ieu-a-1081	|| id:eqtl-a-ENSG00000107796	MR Egger	10	-0.034278752	0.138141898	0.81	0.966302109
IgA nephropathy || id:ieu-a-1081	|| id:eqtl-a-ENSG00000107796	Weighted median	10	-0.011094296	0.10335728	0.915	0.988967019
IgA nephropathy || id:ieu-a-1081	|| id:eqtl-a-ENSG00000107796	Inverse variance weighted (fixed effects)	10	-0.025279256	0.092768053	0.785	0.975037589
IgA nephropathy || id:ieu-a-1081	|| id:eqtl-a-ENSG00000107796	Simple mode	10	-0.138491768	0.221113519	0.547	0.870670419
IgA nephropathy || id:ieu-a-1081	|| id:eqtl-a-ENSG00000107796	Weighted mode	10	-0.006932141	0.099272596	0.946	0.993091831
IgA nephropathy || id:ieu-a-1081	|| id:eqtl-a-ENSG00000109381	MR Egger	13	0.797369733	0.302072184	0.023	2.219694854
IgA nephropathy || id:ieu-a-1081	|| id:eqtl-a-ENSG00000109381	Weighted median	13	0.343681464	0.166600285	0.039	1.410129387
IgA nephropathy || id:ieu-a-1081	|| id:eqtl-a-ENSG00000109381	Inverse variance weighted (fixed effects)	13	0.289492031	0.127228414	0.023	1.335748796
IgA nephropathy || id:ieu-a-1081	|| id:eqtl-a-ENSG00000109381	Simple mode	13	0.356569337	0.239737407	0.163	1.428420569
IgA nephropathy || id:ieu-a-1081	|| id:eqtl-a-ENSG00000109381	Weighted mode	13	0.441032951	0.199637127	0.047	1.554311918
IgA nephropathy || id:ieu-a-1081	|| id:eqtl-a-ENSG00000109819	MR Egger	11	0.077111241	0.16083834	0.643	1.080162228
IgA nephropathy || id:ieu-a-1081	|| id:eqtl-a-ENSG00000109819	Weighted median	11	0.122867747	0.113148496	0.278	1.130734868
IgA nephropathy || id:ieu-a-1081	|| id:eqtl-a-ENSG00000109819	Inverse variance weighted (fixed effects)	11	0.157464403	0.089977064	0.08	1.17053909
IgA nephropathy || id:ieu-a-1081	|| id:eqtl-a-ENSG00000109819	Simple mode	11	0.148495346	0.136217882	0.301	1.160087399
IgA nephropathy || id:ieu-a-1081	|| id:eqtl-a-ENSG00000109819	Weighted mode	11	0.134890461	0.11515659	0.269	1.144411419
IgA nephropathy || id:ieu-a-1081	|| id:eqtl-a-ENSG00000112299	MR Egger	17	-0.029203982	0.082878301	0.729	0.971218333

IgA nephropathy || id:ieu-a-1081	|| id:eqtl-a-ENSG00000112299	Weighted median	17	-0.026171906	0.065158147	0.688	0.97416761
IgA nephropathy || id:ieu-a-1081	|| id:eqtl-a-ENSG00000112299	Inverse variance weighted (fixed effects)	17	-0.034513965	0.049868857	0.489	0.966074848
IgA nephropathy || id:ieu-a-1081	|| id:eqtl-a-ENSG00000112299	Simple mode	17	-0.0533393	0.080350351	0.516	0.948058282
IgA nephropathy || id:ieu-a-1081	|| id:eqtl-a-ENSG00000112299	Weighted mode	17	-0.033339231	0.061059014	0.593	0.967210396
IgA nephropathy || id:ieu-a-1081	|| id:eqtl-a-ENSG00000113249	MR Egger	5	-2.020364877	2.315337056	0.447	0.132607071
IgA nephropathy || id:ieu-a-1081	|| id:eqtl-a-ENSG00000113249	Weighted median	5	0.636131445	0.515230274	0.217	1.889158412
IgA nephropathy || id:ieu-a-1081	|| id:eqtl-a-ENSG00000113249	Inverse variance weighted (fixed effects)	5	0.743317656	0.396292917	0.061	2.102900655
IgA nephropathy || id:ieu-a-1081	|| id:eqtl-a-ENSG00000113249	Simple mode	5	0.044522696	0.769031586	0.957	1.045528706
IgA nephropathy || id:ieu-a-1081	|| id:eqtl-a-ENSG00000113249	Weighted mode	5	0.538870299	0.65932116	0.46	1.714069382
IgA nephropathy || id:ieu-a-1081	|| id:eqtl-a-ENSG00000116044	MR Egger	3	4.321538606	5.362198326	0.568	75.30440304
IgA nephropathy || id:ieu-a-1081	|| id:eqtl-a-ENSG00000116044	Weighted median	3	-1.132514692	0.691814096	0.102	0.322221948
IgA nephropathy || id:ieu-a-1081	|| id:eqtl-a-ENSG00000116044	Inverse variance weighted (fixed effects)	3	-0.904505283	0.572151631	0.114	0.404742068
IgA nephropathy || id:ieu-a-1081	|| id:eqtl-a-ENSG00000116044	Simple mode	3	-1.359182062	0.916497319	0.276	0.256870795
IgA nephropathy || id:ieu-a-1081	|| id:eqtl-a-ENSG00000116044	Weighted mode	3	-1.351182832	0.927377963	0.282	0.258933804
IgA nephropathy || id:ieu-a-1081	|| id:eqtl-a-ENSG00000118217	MR Egger	13	-0.091554355	0.081574209	0.286	0.912511716
IgA nephropathy || id:ieu-a-1081	|| id:eqtl-a-ENSG00000118217	Weighted median	13	-0.144807678	0.075605095	0.055	0.865188672
IgA nephropathy || id:ieu-a-1081	|| id:eqtl-a-ENSG00000118217	Inverse variance weighted (fixed effects)	13	-0.129019761	0.059843694	0.031	0.878956596
IgA nephropathy || id:ieu-a-1081	|| id:eqtl-a-ENSG00000118217	Simple mode	13	-0.122605127	0.082026203	0.161	0.884612904
IgA nephropathy || id:ieu-a-1081	|| id:eqtl-a-ENSG00000118217	Weighted mode	13	-0.129382889	0.078535768	0.125	0.87863748
IgA nephropathy || id:ieu-a-1081	|| id:eqtl-a-ENSG00000121691	MR Egger	9	-0.069571511	0.123788682	0.592	0.932793426
IgA nephropathy || id:ieu-a-1081	|| id:eqtl-a-ENSG00000121691	Weighted median	9	-0.172407696	0.08703013	0.048	0.841635972
IgA nephropathy || id:ieu-a-1081	|| id:eqtl-a-ENSG00000121691	Inverse variance weighted (fixed effects)	9	-0.155689085	0.071752426	0.03	0.855825238
IgA nephropathy || id:ieu-a-1081	|| id:eqtl-a-ENSG00000121691	Simple mode	9	0.003740008	0.126388822	0.977	1.00374701
IgA nephropathy || id:ieu-a-1081	|| id:eqtl-a-ENSG00000121691	Weighted mode	9	-0.173802191	0.079026727	0.059	0.840463132
IgA nephropathy || id:ieu-a-1081	|| id:eqtl-a-ENSG00000125538	MR Egger	16	0.60517008	0.965124064	0.541	1.831563694
IgA nephropathy || id:ieu-a-1081	|| id:eqtl-a-ENSG00000125538	Weighted median	16	-0.039525125	0.273912329	0.885	0.961245802
IgA nephropathy || id:ieu-a-1081	|| id:eqtl-a-ENSG00000125538	Inverse variance weighted (fixed effects)	16	-0.052462458	0.213098561	0.806	0.948889944
IgA nephropathy || id:ieu-a-1081	|| id:eqtl-a-ENSG00000125538	Simple mode	16	0.011195521	0.447622507	0.98	1.011258425
IgA nephropathy || id:ieu-a-1081	|| id:eqtl-a-ENSG00000125538	Weighted mode	16	-0.003010561	0.403020586	0.994	0.996993967
IgA nephropathy || id:ieu-a-1081	|| id:eqtl-a-ENSG00000126581	MR Egger	11	0.187875703	0.245980962	0.465	1.206683519
IgA nephropathy || id:ieu-a-1081	|| id:eqtl-a-ENSG00000126581	Weighted median	11	0.037590998	0.16981285	0.825	1.038306476
IgA nephropathy || id:ieu-a-1081	|| id:eqtl-a-ENSG00000126581	Inverse variance weighted (fixed effects)	11	0.007740147	0.142888465	0.957	1.00777018
IgA nephropathy || id:ieu-a-1081	|| id:eqtl-a-ENSG00000126581	Simple mode	11	0.028149251	0.23030229	0.905	1.028549185
IgA nephropathy || id:ieu-a-1081	|| id:eqtl-a-ENSG00000126581	Weighted mode	11	0.04007214	0.190070297	0.837	1.040885861
IgA nephropathy || id:ieu-a-1081	|| id:eqtl-a-ENSG00000135218	MR Egger	14	-0.124286519	0.095781154	0.219	0.883126772
IgA nephropathy || id:ieu-a-1081	|| id:eqtl-a-ENSG00000135218	Weighted median	14	0.100547225	0.070326884	0.153	1.105775861
IgA nephropathy || id:ieu-a-1081	|| id:eqtl-a-ENSG00000135218	Inverse variance weighted	14	0.140276552	0.052232726	0.007	1.150591953

		(fixed effects)					
IgA nephropathy || id:ieu-a-1081	|| id:eqtl-a-ENSG00000135218	Simple mode	14	0.245885146	0.099516388	0.028	1.278752695
IgA nephropathy || id:ieu-a-1081	|| id:eqtl-a-ENSG00000135218	Weighted mode	14	0.085155677	0.0626545	0.197	1.088886568
IgA nephropathy || id:ieu-a-1081	|| id:eqtl-a-ENSG00000136634	MR Egger	4	-0.160152862	0.638175433	0.825	0.852013538
IgA nephropathy || id:ieu-a-1081	|| id:eqtl-a-ENSG00000136634	Weighted median	4	-0.420219608	0.312959103	0.179	0.656902543
IgA nephropathy || id:ieu-a-1081	|| id:eqtl-a-ENSG00000136634	Inverse variance weighted (fixed effects)	4	-0.524957491	0.278131768	0.059	0.591580511
IgA nephropathy || id:ieu-a-1081	|| id:eqtl-a-ENSG00000136634	Simple mode	4	-0.863276471	0.456507879	0.155	0.421777873
IgA nephropathy || id:ieu-a-1081	|| id:eqtl-a-ENSG00000136634	Weighted mode	4	-0.404204664	0.336087235	0.315	0.667507493
IgA nephropathy || id:ieu-a-1081	|| id:eqtl-a-ENSG00000137752	MR Egger	17	0.157968754	0.198398477	0.438	1.171129601
IgA nephropathy || id:ieu-a-1081	|| id:eqtl-a-ENSG00000137752	Weighted median	17	0.133793869	0.109684993	0.223	1.143157155
IgA nephropathy || id:ieu-a-1081	|| id:eqtl-a-ENSG00000137752	Inverse variance weighted (fixed effects)	17	0.13837272	0.090328653	0.126	1.148403504
IgA nephropathy || id:ieu-a-1081	|| id:eqtl-a-ENSG00000137752	Simple mode	17	0.223509298	0.158503216	0.178	1.250457267
IgA nephropathy || id:ieu-a-1081	|| id:eqtl-a-ENSG00000137752	Weighted mode	17	0.137757617	0.138720449	0.335	1.147697334
IgA nephropathy || id:ieu-a-1081	|| id:eqtl-a-ENSG00000140941	MR Egger	6	-0.816612847	0.592487747	0.24	0.441925993
IgA nephropathy || id:ieu-a-1081	|| id:eqtl-a-ENSG00000140941	Weighted median	6	-0.022002956	0.218540288	0.92	0.978237343
IgA nephropathy || id:ieu-a-1081	|| id:eqtl-a-ENSG00000140941	Inverse variance weighted (multiplicative random effects)	6	0.105997321	0.340199627	0.755	1.111818898
IgA nephropathy || id:ieu-a-1081	|| id:eqtl-a-ENSG00000140941	Simple mode	6	0.425402395	0.437486871	0.376	1.530206043
IgA nephropathy || id:ieu-a-1081	|| id:eqtl-a-ENSG00000140941	Weighted mode	6	-0.145556529	0.207712522	0.515	0.864541017
IgA nephropathy || id:ieu-a-1081	|| id:eqtl-a-ENSG00000142208	MR Egger	7	0.555632281	0.355102298	0.178	1.743042728
IgA nephropathy || id:ieu-a-1081	|| id:eqtl-a-ENSG00000142208	Weighted median	7	0.369556833	0.221982599	0.096	1.447093168
IgA nephropathy || id:ieu-a-1081	|| id:eqtl-a-ENSG00000142208	Inverse variance weighted (fixed effects)	7	0.364340784	0.200765457	0.07	1.439564711
IgA nephropathy || id:ieu-a-1081	|| id:eqtl-a-ENSG00000142208	Simple mode	7	-0.091636794	0.406889283	0.829	0.912436492
IgA nephropathy || id:ieu-a-1081	|| id:eqtl-a-ENSG00000142208	Weighted mode	7	0.450606374	0.238266668	0.107	1.569263457
IgA nephropathy || id:ieu-a-1081	|| id:eqtl-a-ENSG00000145335	MR Egger	16	0.193771704	0.224098998	0.402	1.213819141
IgA nephropathy || id:ieu-a-1081	|| id:eqtl-a-ENSG00000145335	Weighted median	16	0.163799421	0.150409305	0.276	1.177978013
IgA nephropathy || id:ieu-a-1081	|| id:eqtl-a-ENSG00000145335	Inverse variance weighted (fixed effects)	16	0.165958492	0.116496957	0.154	1.1805241
IgA nephropathy || id:ieu-a-1081	|| id:eqtl-a-ENSG00000145335	Simple mode	16	0.340541718	0.205644886	0.118	1.405708883
IgA nephropathy || id:ieu-a-1081	|| id:eqtl-a-ENSG00000145335	Weighted mode	16	0.177588124	0.155566975	0.272	1.194333303
IgA nephropathy || id:ieu-a-1081	|| id:eqtl-a-ENSG00000147872	MR Egger	7	-0.684191007	0.616152799	0.317	0.5044982
IgA nephropathy || id:ieu-a-1081	|| id:eqtl-a-ENSG00000147872	Weighted median	7	-0.416368007	0.308152289	0.177	0.659437548
IgA nephropathy || id:ieu-a-1081	|| id:eqtl-a-ENSG00000147872	Inverse variance weighted (fixed effects)	7	-0.189309914	0.233699745	0.418	0.827530004
IgA nephropathy || id:ieu-a-1081	|| id:eqtl-a-ENSG00000147872	Simple mode	7	-0.458259731	0.452247734	0.35	0.632383206
IgA nephropathy || id:ieu-a-1081	|| id:eqtl-a-ENSG00000147872	Weighted mode	7	-0.472481545	0.306863202	0.175	0.62345322
IgA nephropathy || id:ieu-a-1081	|| id:eqtl-a-ENSG00000148346	MR Egger	11	1.680616591	1.207501752	0.197	5.368865347
IgA nephropathy || id:ieu-a-1081	|| id:eqtl-a-ENSG00000148346	Weighted median	11	0.96179517	0.28479476	0.001	2.616389124
IgA nephropathy || id:ieu-a-1081	|| id:eqtl-a-ENSG00000148346	Inverse variance weighted (fixed effects)	11	0.756734925	0.205351384	0	2.131305974

IgA nephropathy || id:ieu-a-1081	|| id:eqtl-a-ENSG00000148346	Simple mode	11	1.128918267	0.51021384	0.051	3.092309637
IgA nephropathy || id:ieu-a-1081	|| id:eqtl-a-ENSG00000148346	Weighted mode	11	1.146251814	0.537947238	0.059	3.146377573
IgA nephropathy || id:ieu-a-1081	|| id:eqtl-a-ENSG00000154229	MR Egger	10	-0.97161347	0.706927779	0.207	0.378471892
IgA nephropathy || id:ieu-a-1081	|| id:eqtl-a-ENSG00000154229	Weighted median	10	0.549948487	0.396096649	0.165	1.733163735
IgA nephropathy || id:ieu-a-1081	|| id:eqtl-a-ENSG00000154229	Inverse variance weighted (fixed effects)	10	0.356701963	0.271657834	0.189	1.428610028
IgA nephropathy || id:ieu-a-1081	|| id:eqtl-a-ENSG00000154229	Simple mode	10	0.935629733	0.723252545	0.228	2.548818027
IgA nephropathy || id:ieu-a-1081	|| id:eqtl-a-ENSG00000154229	Weighted mode	10	0.783417324	0.631587169	0.246	2.188939816
IgA nephropathy || id:ieu-a-1081	|| id:eqtl-a-ENSG00000161011	MR Egger	12	-0.087862501	0.359762218	0.812	0.915886802
IgA nephropathy || id:ieu-a-1081	|| id:eqtl-a-ENSG00000161011	Weighted median	12	-0.051079369	0.174360817	0.77	0.950203251
IgA nephropathy || id:ieu-a-1081	|| id:eqtl-a-ENSG00000161011	Inverse variance weighted (fixed effects)	12	-0.027582994	0.135856891	0.839	0.972793943
IgA nephropathy || id:ieu-a-1081	|| id:eqtl-a-ENSG00000161011	Simple mode	12	-0.12455088	0.248061275	0.625	0.882893339
IgA nephropathy || id:ieu-a-1081	|| id:eqtl-a-ENSG00000161011	Weighted mode	12	-0.07765409	0.186471415	0.685	0.925284436
IgA nephropathy || id:ieu-a-1081	|| id:eqtl-a-ENSG00000164305	MR Egger	6	0.293924285	0.316366936	0.405	1.341682314
IgA nephropathy || id:ieu-a-1081	|| id:eqtl-a-ENSG00000164305	Weighted median	6	0.132364312	0.107305348	0.217	1.141524114
IgA nephropathy || id:ieu-a-1081	|| id:eqtl-a-ENSG00000164305	Inverse variance weighted (fixed effects)	6	0.090021242	0.090423404	0.319	1.094197526
IgA nephropathy || id:ieu-a-1081	|| id:eqtl-a-ENSG00000164305	Simple mode	6	0.125744572	0.141664891	0.415	1.133992478
IgA nephropathy || id:ieu-a-1081	|| id:eqtl-a-ENSG00000164305	Weighted mode	6	0.137668606	0.132268447	0.346	1.147595181
IgA nephropathy || id:ieu-a-1081	|| id:eqtl-a-ENSG00000165806	MR Egger	17	0.053209622	0.10473328	0.619	1.0546507
IgA nephropathy || id:ieu-a-1081	|| id:eqtl-a-ENSG00000165806	Weighted median	17	0.158242592	0.080594903	0.05	1.171450345
IgA nephropathy || id:ieu-a-1081	|| id:eqtl-a-ENSG00000165806	Inverse variance weighted (fixed effects)	17	0.168444038	0.062912592	0.007	1.183461996
IgA nephropathy || id:ieu-a-1081	|| id:eqtl-a-ENSG00000165806	Simple mode	17	0.176251437	0.128017933	0.188	1.19273792
IgA nephropathy || id:ieu-a-1081	|| id:eqtl-a-ENSG00000165806	Weighted mode	17	0.152132152	0.077333769	0.067	1.164314092
IgA nephropathy || id:ieu-a-1081	|| id:eqtl-a-ENSG00000168610	MR Egger	13	-0.193719537	0.570813048	0.741	0.823888943
IgA nephropathy || id:ieu-a-1081	|| id:eqtl-a-ENSG00000168610	Weighted median	13	0.173714481	0.223928621	0.438	1.189715831
IgA nephropathy || id:ieu-a-1081	|| id:eqtl-a-ENSG00000168610	Inverse variance weighted (fixed effects)	13	0.315461872	0.183210304	0.085	1.370892342
IgA nephropathy || id:ieu-a-1081	|| id:eqtl-a-ENSG00000168610	Simple mode	13	0.319492986	0.334696364	0.359	1.376429718
IgA nephropathy || id:ieu-a-1081	|| id:eqtl-a-ENSG00000168610	Weighted mode	13	0.235678953	0.280873162	0.418	1.265767874
IgA nephropathy || id:ieu-a-1081	|| id:eqtl-a-ENSG00000169710	MR Egger	8	-0.32914834	0.283578652	0.29	0.719536273
IgA nephropathy || id:ieu-a-1081	|| id:eqtl-a-ENSG00000169710	Weighted median	8	-0.172868193	0.128515637	0.179	0.84124849
IgA nephropathy || id:ieu-a-1081	|| id:eqtl-a-ENSG00000169710	Inverse variance weighted (fixed effects)	8	-0.15267842	0.105483458	0.148	0.858405724
IgA nephropathy || id:ieu-a-1081	|| id:eqtl-a-ENSG00000169710	Simple mode	8	-0.164070032	0.142363284	0.287	0.848682585
IgA nephropathy || id:ieu-a-1081	|| id:eqtl-a-ENSG00000169710	Weighted mode	8	-0.167465929	0.121781654	0.211	0.845805434
IgA nephropathy || id:ieu-a-1081	|| id:eqtl-a-ENSG00000171791	MR Egger	9	8.389084313	7.198012785	0.282	4398.787938
IgA nephropathy || id:ieu-a-1081	|| id:eqtl-a-ENSG00000171791	Weighted median	9	-0.265191766	0.666110203	0.691	0.76705884
IgA nephropathy || id:ieu-a-1081	|| id:eqtl-a-ENSG00000171791	Inverse variance weighted (multiplicative random effects)	9	-0.735073219	0.770839791	0.34	0.479470351
IgA nephropathy || id:ieu-a-1081	|| id:eqtl-a-ENSG00000171791	Simple mode	9	-2.577614932	1.654063375	0.158	0.075954946

IgA nephropathy || id:ieu-a-1081	|| id:eqtl-a-ENSG00000171791	Weighted mode	9	1.113661842	1.070915777	0.329	3.045490105
IgA nephropathy || id:ieu-a-1081	|| id:eqtl-a-ENSG00000172071	MR Egger	10	-1.425971051	1.300298652	0.305	0.240275031
IgA nephropathy || id:ieu-a-1081	|| id:eqtl-a-ENSG00000172071	Weighted median	10	-0.743354839	0.33818415	0.028	0.475515957
IgA nephropathy || id:ieu-a-1081	|| id:eqtl-a-ENSG00000172071	Inverse variance weighted (fixed effects)	10	-0.382864754	0.262495912	0.145	0.681905118
IgA nephropathy || id:ieu-a-1081	|| id:eqtl-a-ENSG00000172071	Simple mode	10	-0.831329385	0.60633704	0.204	0.435469994
IgA nephropathy || id:ieu-a-1081	|| id:eqtl-a-ENSG00000172071	Weighted mode	10	-0.877365155	0.515061275	0.123	0.415877241
IgA nephropathy || id:ieu-a-1081	|| id:eqtl-a-ENSG00000175197	MR Egger	4	0.963768041	0.679805338	0.292	2.621556017
IgA nephropathy || id:ieu-a-1081	|| id:eqtl-a-ENSG00000175197	Weighted median	4	0.259342464	0.330494039	0.433	1.296077588
IgA nephropathy || id:ieu-a-1081	|| id:eqtl-a-ENSG00000175197	Inverse variance weighted (fixed effects)	4	0.022499743	0.265656256	0.933	1.022754771
IgA nephropathy || id:ieu-a-1081	|| id:eqtl-a-ENSG00000175197	Simple mode	4	0.209768776	0.430978456	0.66	1.233392837
IgA nephropathy || id:ieu-a-1081	|| id:eqtl-a-ENSG00000175197	Weighted mode	4	0.269051766	0.345127585	0.492	1.308722887
IgA nephropathy || id:ieu-a-1081	|| id:eqtl-a-ENSG00000185532	MR Egger	3	12.83826005	6.503756917	0.299	376344.433
IgA nephropathy || id:ieu-a-1081	|| id:eqtl-a-ENSG00000185532	Weighted median	3	0.84859118	0.72691445	0.243	2.336353032
IgA nephropathy || id:ieu-a-1081	|| id:eqtl-a-ENSG00000185532	Inverse variance weighted (fixed effects)	3	0.234483246	0.562395689	0.677	1.26425529
IgA nephropathy || id:ieu-a-1081	|| id:eqtl-a-ENSG00000185532	Simple mode	3	0.99035578	0.941466039	0.403	2.69219213
IgA nephropathy || id:ieu-a-1081	|| id:eqtl-a-ENSG00000185532	Weighted mode	3	0.961318013	0.869294853	0.384	2.615140992
IgA nephropathy || id:ieu-a-1081	|| id:eqtl-a-ENSG00000186951	MR Egger	4	1.34998099	2.246685858	0.609	3.857352202
IgA nephropathy || id:ieu-a-1081	|| id:eqtl-a-ENSG00000186951	Weighted median	4	0.638451229	0.553719394	0.249	1.893545939
IgA nephropathy || id:ieu-a-1081	|| id:eqtl-a-ENSG00000186951	Inverse variance weighted (fixed effects)	4	0.626833233	0.499505906	0.21	1.871674029
IgA nephropathy || id:ieu-a-1081	|| id:eqtl-a-ENSG00000186951	Simple mode	4	0.378390625	0.757126079	0.652	1.459933118
IgA nephropathy || id:ieu-a-1081	|| id:eqtl-a-ENSG00000186951	Weighted mode	4	0.826254803	0.761460235	0.357	2.284745873
IgA nephropathy || id:ieu-a-1081	|| id:eqtl-a-ENSG00000198793	MR Egger	3	1.439327182	1.933028662	0.593	4.217857014
IgA nephropathy || id:ieu-a-1081	|| id:eqtl-a-ENSG00000198793	Weighted median	3	-0.023959259	0.18121542	0.895	0.976325485
IgA nephropathy || id:ieu-a-1081	|| id:eqtl-a-ENSG00000198793	Inverse variance weighted (fixed effects)	3	-0.040647761	0.159957559	0.799	0.960167279
IgA nephropathy || id:ieu-a-1081	|| id:eqtl-a-ENSG00000198793	Simple mode	3	-0.044226091	0.220612807	0.86	0.956737623
IgA nephropathy || id:ieu-a-1081	|| id:eqtl-a-ENSG00000198793	Weighted mode	3	0.002852917	0.200931804	0.99	1.002856991
IgA nephropathy || id:ieu-a-1081	|| id:eqtl-a-ENSG00000204305	MR Egger	28	-2.023419334	1.077964034	0.072	0.132202646
IgA nephropathy || id:ieu-a-1081	|| id:eqtl-a-ENSG00000204305	Weighted median	28	-2.035313123	0.232937355	0	0.13063957
IgA nephropathy || id:ieu-a-1081	|| id:eqtl-a-ENSG00000204305	Inverse variance weighted (multiplicative random effects)	28	-2.131235823	0.260975168	0	0.118690523
IgA nephropathy || id:ieu-a-1081	|| id:eqtl-a-ENSG00000204305	Simple mode	28	-2.9580337	0.427640929	0	0.051920909
IgA nephropathy || id:ieu-a-1081	|| id:eqtl-a-ENSG00000204305	Weighted mode	28	-1.856149031	0.29512449	0	0.156273277
IgA nephropathy || id:ieu-a-1081	|| id:eqtl-a-ENSG00000232810	MR Egger	38	-1.342651108	0.412019126	0.002	0.261152407
IgA nephropathy || id:ieu-a-1081	|| id:eqtl-a-ENSG00000232810	Weighted median	38	-0.577250164	0.162444921	0	0.561440114
IgA nephropathy || id:ieu-a-1081	|| id:eqtl-a-ENSG00000232810	Inverse variance weighted (multiplicative random effects)	38	-0.517638702	0.149556924	0.001	0.595926047
IgA nephropathy || id:ieu-a-1081	|| id:eqtl-a-ENSG00000232810	Simple mode	38	-0.85373318	0.266566644	0.003	0.42582229

IgA nephropathy || id:ieu-a-1081	|| id:eqtl-a-ENSG00000232810	Weighted mode	38	-0.566917878	0.17507611	0.003	0.567271146

Abbreviation: SE: standard error, OR: odds ratio.

Supplementary Table 6

Outcome	Exposure	Method	Q	Q_df	Q_pval
Membranous nephropathy || id:ebi-a-GCST010005	|| id:eqtl-a-ENSG00000005381	MR Egger	24.31034629	69	1
Membranous nephropathy || id:ebi-a-GCST010005	|| id:eqtl-a-ENSG00000005381	Inverse variance weighted (fixed effects)	34.56214897	70	1
Membranous nephropathy || id:ebi-a-GCST010005	|| id:eqtl-a-ENSG00000026025	MR Egger	17.09286889	23	0.805
Membranous nephropathy || id:ebi-a-GCST010005	|| id:eqtl-a-ENSG00000026025	Inverse variance weighted (fixed effects)	19.11909236	24	0.746
Membranous nephropathy || id:ebi-a-GCST010005	|| id:eqtl-a-ENSG00000039068	MR Egger	41.76687008	47	0.689
Membranous nephropathy || id:ebi-a-GCST010005	|| id:eqtl-a-ENSG00000039068	Inverse variance weighted (fixed effects)	42.55213574	48	0.695
Membranous nephropathy || id:ebi-a-GCST010005	|| id:eqtl-a-ENSG00000044574	MR Egger	799.6630911	15	0
Membranous nephropathy || id:ebi-a-GCST010005	|| id:eqtl-a-ENSG00000044574	Inverse variance weighted (multiplicative random effects)	846.9997081	16	0
Membranous nephropathy || id:ebi-a-GCST010005	|| id:eqtl-a-ENSG00000067606	MR Egger	1.608729137	8	0.991
Membranous nephropathy || id:ebi-a-GCST010005	|| id:eqtl-a-ENSG00000067606	Inverse variance weighted (fixed effects)	2.607209224	9	0.978
Membranous nephropathy || id:ebi-a-GCST010005	|| id:eqtl-a-ENSG00000072310	MR Egger	36.73385734	63	0.997
Membranous nephropathy || id:ebi-a-GCST010005	|| id:eqtl-a-ENSG00000072310	Inverse variance weighted (fixed effects)	43.00465427	64	0.98
Membranous nephropathy || id:ebi-a-GCST010005	|| id:eqtl-a-ENSG00000073756	MR Egger	9.648372518	25	0.997
Membranous nephropathy || id:ebi-a-GCST010005	|| id:eqtl-a-ENSG00000073756	Inverse variance weighted (fixed effects)	9.824661987	26	0.998
Membranous nephropathy || id:ebi-a-GCST010005	|| id:eqtl-a-ENSG00000087088	MR Egger	4.185593733	7	0.758
Membranous nephropathy || id:ebi-a-GCST010005	|| id:eqtl-a-ENSG00000087088	Inverse variance weighted (fixed effects)	10.00127912	8	0.265
Membranous nephropathy || id:ebi-a-GCST010005	|| id:eqtl-a-ENSG00000096717	MR Egger	50.91939883	91	1
Membranous nephropathy || id:ebi-a-GCST010005	|| id:eqtl-a-ENSG00000096717	Inverse variance weighted (fixed effects)	85.16541503	92	0.68
Membranous nephropathy || id:ebi-a-GCST010005	|| id:eqtl-a-ENSG00000096968	MR Egger	17.65818738	24	0.819
Membranous nephropathy || id:ebi-a-GCST010005	|| id:eqtl-a-ENSG00000096968	Inverse variance weighted (fixed effects)	19.31502177	25	0.782
Membranous nephropathy || id:ebi-a-GCST010005	|| id:eqtl-a-ENSG00000100292	MR Egger	38.77812959	21	0.01

Membranous nephropathy || id:ebi-a-GCST010005	|| id:eqtl-a-ENSG00000100292	Inverse variance weighted (multiplicative random effects)	38.91143483	22	0.014
Membranous nephropathy || id:ebi-a-GCST010005	|| id:eqtl-a-ENSG00000107796	MR Egger	42.36313886	49	0.737
Membranous nephropathy || id:ebi-a-GCST010005	|| id:eqtl-a-ENSG00000107796	Inverse variance weighted (fixed effects)	44.33425956	50	0.699
Membranous nephropathy || id:ebi-a-GCST010005	|| id:eqtl-a-ENSG00000109381	MR Egger	3.229119951	19	1
Membranous nephropathy || id:ebi-a-GCST010005	|| id:eqtl-a-ENSG00000109381	Inverse variance weighted (fixed effects)	3.274442808	20	1
Membranous nephropathy || id:ebi-a-GCST010005	|| id:eqtl-a-ENSG00000109819	MR Egger	15.89803261	18	0.6
Membranous nephropathy || id:ebi-a-GCST010005	|| id:eqtl-a-ENSG00000109819	Inverse variance weighted (fixed effects)	16.30579669	19	0.637
Membranous nephropathy || id:ebi-a-GCST010005	|| id:eqtl-a-ENSG00000112299	MR Egger	64.83463585	61	0.344
Membranous nephropathy || id:ebi-a-GCST010005	|| id:eqtl-a-ENSG00000112299	Inverse variance weighted (fixed effects)	67.67168919	62	0.29
Membranous nephropathy || id:ebi-a-GCST010005	|| id:eqtl-a-ENSG00000113249	MR Egger	14.39238911	16	0.57
Membranous nephropathy || id:ebi-a-GCST010005	|| id:eqtl-a-ENSG00000113249	Inverse variance weighted (fixed effects)	15.28451853	17	0.575
Membranous nephropathy || id:ebi-a-GCST010005	|| id:eqtl-a-ENSG00000116044	MR Egger	2.574846167	4	0.631
Membranous nephropathy || id:ebi-a-GCST010005	|| id:eqtl-a-ENSG00000116044	Inverse variance weighted (multiplicative random effects)	13.75702355	5	0.017
Membranous nephropathy || id:ebi-a-GCST010005	|| id:eqtl-a-ENSG00000118217	MR Egger	30.03420784	43	0.933
Membranous nephropathy || id:ebi-a-GCST010005	|| id:eqtl-a-ENSG00000118217	Inverse variance weighted (fixed effects)	30.3725418	44	0.941
Membranous nephropathy || id:ebi-a-GCST010005	|| id:eqtl-a-ENSG00000121691	MR Egger	36.86777739	31	0.216
Membranous nephropathy || id:ebi-a-GCST010005	|| id:eqtl-a-ENSG00000121691	Inverse variance weighted (fixed effects)	37.83453259	32	0.22
Membranous nephropathy || id:ebi-a-GCST010005	|| id:eqtl-a-ENSG00000125538	MR Egger	29.48869062	19	0.059
Membranous nephropathy || id:ebi-a-GCST010005	|| id:eqtl-a-ENSG00000125538	Inverse variance weighted (multiplicative random effects)	33.50827104	20	0.03
Membranous nephropathy || id:ebi-a-GCST010005	|| id:eqtl-a-ENSG00000126581	MR Egger	7.465965335	30	1
Membranous nephropathy || id:ebi-a-GCST010005	|| id:eqtl-a-ENSG00000126581	Inverse variance weighted (fixed effects)	7.577228854	31	1
Membranous nephropathy || id:ebi-a-GCST010005	|| id:eqtl-a-ENSG00000135218	MR Egger	16.80487114	35	0.996

Membranous nephropathy || id:ebi-a-GCST010005	|| id:eqtl-a-ENSG00000135218	Inverse variance weighted (fixed effects)	17.08876663	36	0.997
Membranous nephropathy || id:ebi-a-GCST010005	|| id:eqtl-a-ENSG00000136634	MR Egger	3.071146036	9	0.961
Membranous nephropathy || id:ebi-a-GCST010005	|| id:eqtl-a-ENSG00000136634	Inverse variance weighted (fixed effects)	3.48260124	10	0.968
Membranous nephropathy || id:ebi-a-GCST010005	|| id:eqtl-a-ENSG00000137752	MR Egger	20.67330768	39	0.993
Membranous nephropathy || id:ebi-a-GCST010005	|| id:eqtl-a-ENSG00000137752	Inverse variance weighted (fixed effects)	22.38489973	40	0.989
Membranous nephropathy || id:ebi-a-GCST010005	|| id:eqtl-a-ENSG00000140941	MR Egger	9.174769571	19	0.97
Membranous nephropathy || id:ebi-a-GCST010005	|| id:eqtl-a-ENSG00000140941	Inverse variance weighted (fixed effects)	9.184529588	20	0.981
Membranous nephropathy || id:ebi-a-GCST010005	|| id:eqtl-a-ENSG00000141510	MR Egger	0.158240103	1	0.691
Membranous nephropathy || id:ebi-a-GCST010005	|| id:eqtl-a-ENSG00000141510	Inverse variance weighted (fixed effects)	0.168716697	2	0.919
Membranous nephropathy || id:ebi-a-GCST010005	|| id:eqtl-a-ENSG00000142208	MR Egger	38.8584286	13	0
Membranous nephropathy || id:ebi-a-GCST010005	|| id:eqtl-a-ENSG00000142208	Inverse variance weighted (multiplicative random effects)	41.66249165	14	0
Membranous nephropathy || id:ebi-a-GCST010005	|| id:eqtl-a-ENSG00000145335	MR Egger	47.43974569	40	0.195
Membranous nephropathy || id:ebi-a-GCST010005	|| id:eqtl-a-ENSG00000145335	Inverse variance weighted (multiplicative random effects)	67.33833106	41	0.006
Membranous nephropathy || id:ebi-a-GCST010005	|| id:eqtl-a-ENSG00000147872	MR Egger	11.77883765	28	0.997
Membranous nephropathy || id:ebi-a-GCST010005	|| id:eqtl-a-ENSG00000147872	Inverse variance weighted (fixed effects)	15.82718868	29	0.977
Membranous nephropathy || id:ebi-a-GCST010005	|| id:eqtl-a-ENSG00000148346	MR Egger	304.5492407	14	0
Membranous nephropathy || id:ebi-a-GCST010005	|| id:eqtl-a-ENSG00000148346	Inverse variance weighted (multiplicative random effects)	322.6636814	15	0
Membranous nephropathy || id:ebi-a-GCST010005	|| id:eqtl-a-ENSG00000154229	MR Egger	40.1313914	46	0.716
Membranous nephropathy || id:ebi-a-GCST010005	|| id:eqtl-a-ENSG00000154229	Inverse variance weighted (fixed effects)	46.92854911	47	0.475
Membranous nephropathy || id:ebi-a-GCST010005	|| id:eqtl-a-ENSG00000161011	MR Egger	111.4534332	41	0
Membranous nephropathy || id:ebi-a-GCST010005	|| id:eqtl-a-ENSG00000161011	Inverse variance weighted (multiplicative random effects)	124.3016694	42	0
Membranous nephropathy || id:ebi-a-GCST010005	|| id:eqtl-a-ENSG00000162711	MR Egger	36.8172717	23	0.034

Membranous nephropathy || id:ebi-a-GCST010005	|| id:eqtl-a-ENSG00000162711	Inverse variance weighted (multiplicative random effects)	37.75166953	24	0.037
Membranous nephropathy || id:ebi-a-GCST010005	|| id:eqtl-a-ENSG00000164305	MR Egger	5.146183963	16	0.995
Membranous nephropathy || id:ebi-a-GCST010005	|| id:eqtl-a-ENSG00000164305	Inverse variance weighted (fixed effects)	5.148202162	17	0.997
Membranous nephropathy || id:ebi-a-GCST010005	|| id:eqtl-a-ENSG00000165806	MR Egger	20.39169278	34	0.968
Membranous nephropathy || id:ebi-a-GCST010005	|| id:eqtl-a-ENSG00000165806	Inverse variance weighted (fixed effects)	20.81060102	35	0.972
Membranous nephropathy || id:ebi-a-GCST010005	|| id:eqtl-a-ENSG00000168610	MR Egger	9.128969662	40	1
Membranous nephropathy || id:ebi-a-GCST010005	|| id:eqtl-a-ENSG00000168610	Inverse variance weighted (fixed effects)	9.276306552	41	1
Membranous nephropathy || id:ebi-a-GCST010005	|| id:eqtl-a-ENSG00000169710	MR Egger	2.771099309	14	0.999
Membranous nephropathy || id:ebi-a-GCST010005	|| id:eqtl-a-ENSG00000169710	Inverse variance weighted (fixed effects)	7.715197715	15	0.935
Membranous nephropathy || id:ebi-a-GCST010005	|| id:eqtl-a-ENSG00000171105	MR Egger	0.695416511	4	0.952
Membranous nephropathy || id:ebi-a-GCST010005	|| id:eqtl-a-ENSG00000171105	Inverse variance weighted (fixed effects)	0.702028064	5	0.983
Membranous nephropathy || id:ebi-a-GCST010005	|| id:eqtl-a-ENSG00000171791	MR Egger	794.8743649	25	0
Membranous nephropathy || id:ebi-a-GCST010005	|| id:eqtl-a-ENSG00000171791	Inverse variance weighted (multiplicative random effects)	925.7071947	26	0
Membranous nephropathy || id:ebi-a-GCST010005	|| id:eqtl-a-ENSG00000172071	MR Egger	4.38426984	16	0.998
Membranous nephropathy || id:ebi-a-GCST010005	|| id:eqtl-a-ENSG00000172071	Inverse variance weighted (fixed effects)	5.766369464	17	0.995
Membranous nephropathy || id:ebi-a-GCST010005	|| id:eqtl-a-ENSG00000173039	MR Egger	1.66E-05	1	0.997
Membranous nephropathy || id:ebi-a-GCST010005	|| id:eqtl-a-ENSG00000173039	Inverse variance weighted (fixed effects)	0.018106776	2	0.991
Membranous nephropathy || id:ebi-a-GCST010005	|| id:eqtl-a-ENSG00000175197	MR Egger	16.47449617	16	0.42
Membranous nephropathy || id:ebi-a-GCST010005	|| id:eqtl-a-ENSG00000175197	Inverse variance weighted (fixed effects)	17.20077072	17	0.441
Membranous nephropathy || id:ebi-a-GCST010005	|| id:eqtl-a-ENSG00000185532	MR Egger	5.435361318	10	0.86
Membranous nephropathy || id:ebi-a-GCST010005	|| id:eqtl-a-ENSG00000185532	Inverse variance weighted (fixed effects)	10.89527767	11	0.452

Abbreviation: Q:Chi-Square Test, Q_df:Quantile Degrees of Freedom.

Supplementary Table 7

Outcome	Exposure	Egger_intercept	SE	*P* vale	Judge
IgA nephropathy || id:ieu-a-1081	|| id:eqtl-a-ENSG00000005381	-0.055691784	0.039726338	0.174301332	NO
IgA nephropathy || id:ieu-a-1081	|| id:eqtl-a-ENSG00000026025	-0.110448442	0.137510219	0.45832275	NO
IgA nephropathy || id:ieu-a-1081	|| id:eqtl-a-ENSG00000039068	-0.018455288	0.048295954	0.709046932	NO
IgA nephropathy || id:ieu-a-1081	|| id:eqtl-a-ENSG00000044574	0.126633746	0.166024883	0.585175568	NO
IgA nephropathy || id:ieu-a-1081	|| id:eqtl-a-ENSG00000067606	-0.006978396	0.160492266	0.967401938	NO
IgA nephropathy || id:ieu-a-1081	|| id:eqtl-a-ENSG00000072310	-0.086621787	0.040350792	0.042131573	YES
IgA nephropathy || id:ieu-a-1081	|| id:eqtl-a-ENSG00000073756	-0.038249628	0.074470542	0.621398195	NO
IgA nephropathy || id:ieu-a-1081	|| id:eqtl-a-ENSG00000087088	0.355270265	0.328099274	0.392069654	NO
IgA nephropathy || id:ieu-a-1081	|| id:eqtl-a-ENSG00000096717	-0.059510044	0.041561855	0.162879374	NO
IgA nephropathy || id:ieu-a-1081	|| id:eqtl-a-ENSG00000096968	-0.043758017	0.049846289	0.402846435	NO
IgA nephropathy || id:ieu-a-1081	|| id:eqtl-a-ENSG00000100292	0.032636594	0.075547384	0.678735001	NO
IgA nephropathy || id:ieu-a-1081	|| id:eqtl-a-ENSG00000107796	0.004437869	0.050475477	0.932100243	NO
IgA nephropathy || id:ieu-a-1081	|| id:eqtl-a-ENSG00000109381	-0.112750585	0.060822671	0.090762227	NO
IgA nephropathy || id:ieu-a-1081	|| id:eqtl-a-ENSG00000109819	0.029002825	0.048119258	0.561567462	NO
IgA nephropathy || id:ieu-a-1081	|| id:eqtl-a-ENSG00000112299	-0.002781632	0.034676738	0.937125865	NO
IgA nephropathy || id:ieu-a-1081	|| id:eqtl-a-ENSG00000113249	0.287684285	0.236606928	0.310980168	NO
IgA nephropathy || id:ieu-a-1081	|| id:eqtl-a-ENSG00000116044	-0.44465289	0.453632871	0.506363947	NO
IgA nephropathy || id:ieu-a-1081	|| id:eqtl-a-ENSG00000118217	-0.027240693	0.040306484	0.513100889	NO
IgA nephropathy || id:ieu-a-1081	|| id:eqtl-a-ENSG00000121691	-0.047305207	0.055410137	0.421506398	NO
IgA nephropathy || id:ieu-a-1081	|| id:eqtl-a-ENSG00000125538	-0.064529316	0.092364213	0.496219728	NO
IgA nephropathy || id:ieu-a-1081	|| id:eqtl-a-ENSG00000126581	-0.049919188	0.055486011	0.391730982	NO
IgA nephropathy || id:ieu-a-1081	|| id:eqtl-a-ENSG00000135218	0.137083589	0.041600036	0.006396487	YES
IgA nephropathy || id:ieu-a-1081	|| id:eqtl-a-ENSG00000136634	-0.072993137	0.114926406	0.590314929	NO
IgA nephropathy || id:ieu-a-1081	|| id:eqtl-a-ENSG00000137752	-0.007850687	0.070767765	0.913137925	NO
IgA nephropathy || id:ieu-a-1081	|| id:eqtl-a-ENSG00000140941	0.203630942	0.114710975	0.150530201	NO
IgA nephropathy || id:ieu-a-1081	|| id:eqtl-a-ENSG00000142208	-0.041706618	0.063860138	0.542530826	NO
IgA nephropathy || id:ieu-a-1081	|| id:eqtl-a-ENSG00000145335	-0.006215026	0.042778132	0.886556728	NO
IgA nephropathy || id:ieu-a-1081	|| id:eqtl-a-ENSG00000147872	0.087474888	0.098996021	0.417357554	NO
IgA nephropathy || id:ieu-a-1081	|| id:eqtl-a-ENSG00000148346	-0.122705021	0.157902328	0.457041254	NO
IgA nephropathy || id:ieu-a-1081	|| id:eqtl-a-ENSG00000154229	0.177044716	0.085268822	0.071523973	NO
IgA nephropathy || id:ieu-a-1081	|| id:eqtl-a-ENSG00000161011	0.011914766	0.065844888	0.860020074	NO
IgA nephropathy || id:ieu-a-1081	|| id:eqtl-a-ENSG00000164305	-0.106497549	0.158343842	0.5380742	NO
IgA nephropathy || id:ieu-a-1081	|| id:eqtl-a-ENSG00000165806	0.052560859	0.038191987	0.188950444	NO
IgA nephropathy || id:ieu-a-1081	|| id:eqtl-a-ENSG00000168610	0.068658975	0.072897157	0.366489133	NO
IgA nephropathy || id:ieu-a-1081	|| id:eqtl-a-ENSG00000169710	0.055862524	0.083326939	0.527548138	NO
IgA nephropathy || id:ieu-a-1081	|| id:eqtl-a-ENSG00000171791	-0.765602377	0.600759454	0.243197954	NO
IgA nephropathy || id:ieu-a-1081	|| id:eqtl-a-ENSG00000172071	0.112074005	0.136831055	0.436465651	NO
IgA nephropathy || id:ieu-a-1081	|| id:eqtl-a-ENSG00000175197	-0.197555169	0.131333423	0.271430582	NO
IgA nephropathy || id:ieu-a-1081	|| id:eqtl-a-ENSG00000185532	-1.204840898	0.619388992	0.302299494	NO
IgA nephropathy || id:ieu-a-1081	|| id:eqtl-a-ENSG00000186951	-0.083799111	0.253832143	0.772670623	NO
IgA nephropathy || id:ieu-a-1081	|| id:eqtl-a-ENSG00000198793	-0.502270892	0.653777407	0.582959734	NO
IgA nephropathy || id:ieu-a-1081	|| id:eqtl-a-ENSG00000204305	-0.018157742	0.175934211	0.918590187	NO

IgA nephropathy || id:ieu-a-1081	|| id:eqtl-a-ENSG00000232810	0.143479614	0.067210728	0.03965795	YES

Abbreviation: SE: standard error.

Supplementary Table 8

Exposure	Outcome	SNP_R2.Exposure	SNP_R2.Outcome	Correct_Causal_Direction	Steiger_*P* vale
|| id:eqtl-a-ENSG00000005381	IgA nephropathy || id:ieu-a-1081	0.312021811	0.00507289	TRUE	0
|| id:eqtl-a-ENSG00000096968	IgA nephropathy || id:ieu-a-1081	0.101467338	0.001880401	TRUE	1.43E-91
|| id:eqtl-a-ENSG00000109381	IgA nephropathy || id:ieu-a-1081	0.08930489	0.002405498	TRUE	2.02E-75
|| id:eqtl-a-ENSG00000118217	IgA nephropathy || id:ieu-a-1081	0.244139048	0.002228536	TRUE	3.14E-268
|| id:eqtl-a-ENSG00000121691	IgA nephropathy || id:ieu-a-1081	0.140261102	0.001850369	TRUE	1.19E-135
|| id:eqtl-a-ENSG00000148346	IgA nephropathy || id:ieu-a-1081	0.071804888	0.003967471	TRUE	7.53E-43
|| id:eqtl-a-ENSG00000165806	IgA nephropathy || id:ieu-a-1081	0.193304993	0.00308375	TRUE	3.18E-191
|| id:eqtl-a-ENSG00000204305	IgA nephropathy || id:ieu-a-1081	0.332663108	0.057962252	TRUE	2.15E-132

Supplementary Table 9

Chemical.Name	Chemical.ID	CAS.RN	Gene.Symbol	Gene.ID	Interaction	Interaction.Actions	Reference.Count	Organism.Count
Canagliflozin	D000068896	NA	ACHE	43	Canagliflozin inhibits the reaction [Scopolamine results in increased activity of ACHE protein]	decreases^reaction|increases^activity	1	1
Canagliflozin	D000068896	NA	ACHE	43	Canagliflozin inhibits the reaction [[Streptozocin co-treated with Dietary Fats] results in increased activity of ACHE protein]	affects^cotreatment|decreases^reaction|increases^activity	1	1
Canagliflozin	D000068896	NA	CHRM1	1128	Canagliflozin affects the reaction [Scopolamine results in decreased expression of CHRM1 protein]	affects^reaction|decreases^expression	1	1
Canagliflozin	D000068896	NA	CHRM1	1128	Canagliflozin results in decreased expression of CHRM1 protein	decreases^expression	1	1
Canagliflozin	D000068896	NA	HAVCR1	26762	Canagliflozin results in increased expression of HAVCR1	increases^expression	1	1
Canagliflozin	D000068896	NA	LHB	3972	Canagliflozin results in increased expression of LHB protein	increases^expression	1	1
Canagliflozin	D000068896	NA	PTH	5741	Canagliflozin results in decreased expression of PTH protein	decreases^expression	1	1
Canagliflozin	D000068896	NA	UGT1A10	54575	Canagliflozin results in decreased activity of UGT1A10 protein	decreases^activity	1	1
Canagliflozin	D000068896	NA	UGT1A8	54576	Canagliflozin results in decreased activity of UGT1A8 protein	decreases^activity	1	1
dapagliflozin	C529054	NA	ACTA2	59	[dapagliflozin co-treated with Atorvastatin] inhibits the reaction [[Dietary Fats co-treated with Fructose] results in increased expression of ACTA2 protein]	affects^cotreatment|decreases^reaction|increases^expression	1	1
dapagliflozin	C529054	NA	ACTA2	59	dapagliflozin inhibits the reaction [[Streptozocin co-treated with Fructose] results in increased expression of ACTA2 protein]	affects^cotreatment|decreases^reaction|increases^expression	1	1
dapagliflozin	C529054	NA	ACTA2	59	Metformin promotes the reaction [dapagliflozin inhibits the reaction [[Streptozocin co-treated with Fructose] results in increased expression of ACTA2 protein]]	affects^cotreatment|decreases^reaction|increases^expression|increases^reaction	1	1
dapagliflozin	C529054	NA	AGER	177	dapagliflozin inhibits the reaction [Trinitrobenzenesulfonic Acid results in increased expression of AGER protein]	decreases^reaction|increases^expression	1	1
dapagliflozin	C529054	NA	AKT1	207	[dapagliflozin co-treated with Atorvastatin] inhibits the reaction	affects^cotreatment|decreases^expression|decr	1	1

					[[Fructose co-treated with Dietary Fats] results in decreased expression of AKT1 protein]	eases^reaction		
dapagliflozin	C529054	NA	AKT1	207	[dapagliflozin co-treated with Vildagliptin] inhibits the reaction [Dietary Fats results in decreased phosphorylation of AKT1 protein]	affects^cotreatment|decreases^phosphorylation|decreases^reaction	1	1
dapagliflozin	C529054	NA	AKT1	207	dapagliflozin inhibits the reaction [[Dietary Fats co-treated with Streptozocin] results in decreased phosphorylation of AKT1 protein]	affects^cotreatment|decreases^phosphorylation|decreases^reaction	1	1
dapagliflozin	C529054	NA	AKT1	207	dapagliflozin inhibits the reaction [Dietary Fats results in decreased phosphorylation of AKT1 protein]	decreases^phosphorylation|decreases^reaction	1	1
dapagliflozin	C529054	NA	ATF6	22926	[dapagliflozin co-treated with Atorvastatin] inhibits the reaction [[Dietary Fats co-treated with Fructose] results in increased expression of ATF6 protein]	affects^cotreatment|decreases^reaction|increases^expression	1	1
dapagliflozin	C529054	NA	BAX	581	dapagliflozin inhibits the reaction [Dietary Fats results in increased expression of BAX protein]	decreases^reaction|increases^expression	1	1
dapagliflozin	C529054	NA	BAX	581	dapagliflozin inhibits the reaction [Doxorubicin results in increased expression of BAX protein]	decreases^reaction|increases^expression	1	1
dapagliflozin	C529054	NA	BAX	581	dapagliflozin promotes the reaction [Trinitrobenzenesulfonic Acid results in increased expression of BAX protein]	increases^expression|increases^reaction	1	1
dapagliflozin	C529054	NA	BAX	581	[Vildagliptin co-treated with dapagliflozin] inhibits the reaction [Dietary Fats results in increased expression of BAX protein]	affects^cotreatment|decreases^reaction|increases^expression	1	1
dapagliflozin	C529054	NA	BCL2	596	[Atorvastatin co-treated with dapagliflozin] inhibits the reaction [[Fructose co-treated with Dietary Fats] results in decreased expression of BCL2 protein]	affects^cotreatment|decreases^expression|decreases^reaction	1	1
dapagliflozin	C529054	NA	BCL2	596	[dapagliflozin co-treated with Vildagliptin] inhibits the reaction [Dietary Fats results in decreased expression of BCL2 protein]	affects^cotreatment|decreases^expression|decreases^reaction	1	1
dapagliflozin	C529054	NA	BCL2	596	dapagliflozin inhibits the reaction [Dietary Fats results in decreased	decreases^expression|decreases^reaction	1	1

					expression of BCL2 protein]			
dapagliflozin	C529054	NA	BCL2	596	dapagliflozin inhibits the reaction [Doxorubicin results in decreased expression of BCL2 protein]	decreases^expression|decreases^reaction	1	1
dapagliflozin	C529054	NA	BCL2	596	dapagliflozin inhibits the reaction [[Fructose co-treated with Dietary Fats] results in decreased expression of BCL2 protein]	affects^cotreatment|decreases^expression|decreases^reaction	1	1
dapagliflozin	C529054	NA	BCL2	596	dapagliflozin inhibits the reaction [Trinitrobenzenesulfonic Acid results in decreased expression of BCL2 protein]	decreases^expression|decreases^reaction	1	1
dapagliflozin	C529054	NA	BCL2	596	STAT3 protein promotes the reaction [dapagliflozin inhibits the reaction [Doxorubicin results in decreased expression of BCL2 protein]]	decreases^expression|decreases^reaction|increases^reaction	1	1
dapagliflozin	C529054	NA	BECN1	8678	dapagliflozin promotes the reaction [Trinitrobenzenesulfonic Acid results in increased expression of BECN1 protein]	increases^expression|increases^reaction	1	1
dapagliflozin	C529054	NA	CASP1	834	[dapagliflozin co-treated with Atorvastatin] inhibits the reaction [[Dietary Fats co-treated with Fructose] results in increased expression of CASP1 protein]	affects^cotreatment|decreases^reaction|increases^expression	1	1
dapagliflozin	C529054	NA	CASP1	834	dapagliflozin inhibits the reaction [[Dietary Fats co-treated with Fructose] results in increased expression of CASP1 protein]	affects^cotreatment|decreases^reaction|increases^expression	1	1
dapagliflozin	C529054	NA	CASP3	836	dapagliflozin inhibits the reaction [Doxorubicin results in increased expression of CASP3 protein modified form]	decreases^reaction|increases^expression	1	1
dapagliflozin	C529054	NA	CASP3	836	dapagliflozin inhibits the reaction [Trinitrobenzenesulfonic Acid results in increased activity of and results in increased cleavage of CASP3 protein]	decreases^reaction|increases^activity|increases^cleavage	1	1
dapagliflozin	C529054	NA	CASP7	840	dapagliflozin inhibits the reaction [[Streptozocin co-treated with Fructose] results in increased expression of CASP7 protein]	affects^cotreatment|decreases^reaction|increases^expression	1	1
dapagliflozin	C529054	NA	CASP7	840	dapagliflozin promotes the reaction [Metformin inhibits the reaction [[Streptozocin co-treated with	affects^cotreatment|decreases^reaction|increases^expression|increases	1	1

					Fructose] results in increased expression of CASP7 protein]]	^reaction		
dapagliflozin	C529054	NA	CASP7	840	Metformin promotes the reaction [dapagliflozin inhibits the reaction [[Streptozocin co-treated with Fructose] results in increased expression of CASP7 protein]]	affects^cotreatment|decreases^reaction|increases^expression|increases^reaction	1	1
dapagliflozin	C529054	NA	CCL2	6347	[dapagliflozin co-treated with Atorvastatin] inhibits the reaction [[Dietary Fats co-treated with Fructose] results in increased expression of CCL2 protein]	affects^cotreatment|decreases^reaction|increases^expression	1	1
dapagliflozin	C529054	NA	CCL2	6347	dapagliflozin inhibits the reaction [[Streptozocin co-treated with Fructose] results in increased expression of and results in increased secretion of CCL2 protein]	affects^cotreatment|decreases^reaction|increases^expression|increases^secretion	1	1
dapagliflozin	C529054	NA	CD36	948	[Atorvastatin co-treated with dapagliflozin] inhibits the reaction [[Fructose co-treated with Dietary Fats] results in increased expression of CD36 protein]	affects^cotreatment|decreases^reaction|increases^expression	1	1
dapagliflozin	C529054	NA	CD36	948	dapagliflozin inhibits the reaction [[Fructose co-treated with Dietary Fats] results in increased expression of CD36 protein]	affects^cotreatment|decreases^reaction|increases^expression	1	1
dapagliflozin	C529054	NA	CDH1	999	[dapagliflozin co-treated with Atorvastatin] inhibits the reaction [[Dietary Fats co-treated with Fructose] results in decreased expression of CDH1 protein]	affects^cotreatment|decreases^expression|decreases^reaction	1	1
dapagliflozin	C529054	NA	CDH1	999	dapagliflozin inhibits the reaction [[Dietary Fats co-treated with Fructose] results in decreased expression of CDH1 protein]	affects^cotreatment|decreases^expression|decreases^reaction	1	1
dapagliflozin	C529054	NA	CYCS	54205	[Atorvastatin co-treated with dapagliflozin] inhibits the reaction [[Fructose co-treated with Dietary Fats] results in increased expression of CYCS protein]	affects^cotreatment|decreases^reaction|increases^expression	1	1
dapagliflozin	C529054	NA	EPO	2056	dapagliflozin inhibits the reaction [[Dietary Fats co-treated with Streptozocin] results in decreased expression of EPO protein]	affects^cotreatment|decreases^expression|decreases^reaction	1	1

dapagliflozin	C529054	NA	FASN	2194	[Atorvastatin co-treated with dapagliflozin] inhibits the reaction [[Fructose co-treated with Dietary Fats] results in increased expression of FASN protein]	affects^cotreatment|decreases^reaction|increases^expression	1	1
dapagliflozin	C529054	NA	FASN	2194	dapagliflozin inhibits the reaction [[Fructose co-treated with Dietary Fats] results in increased expression of FASN protein]	affects^cotreatment|decreases^reaction|increases^expression	1	1
dapagliflozin	C529054	NA	GJA1	2697	Atorvastatin promotes the reaction [dapagliflozin inhibits the reaction [[Dietary Fats co-treated with Fructose] results in decreased expression of GJA1 protein]]	affects^cotreatment|decreases^expression|decreases^reaction|increases^reaction	1	1
dapagliflozin	C529054	NA	GJA1	2697	dapagliflozin inhibits the reaction [[Dietary Fats co-treated with Fructose] results in decreased expression of GJA1 protein]	affects^cotreatment|decreases^expression|decreases^reaction	1	1
dapagliflozin	C529054	NA	GPT	2875	[dapagliflozin co-treated with Atorvastatin] inhibits the reaction [[Fructose co-treated with Dietary Fats] results in increased expression of GPT protein]	affects^cotreatment|decreases^reaction|increases^expression	1	1
dapagliflozin	C529054	NA	GPT	2875	dapagliflozin inhibits the reaction [[Fructose co-treated with Dietary Fats] results in increased expression of GPT protein]	affects^cotreatment|decreases^reaction|increases^expression	1	1
dapagliflozin	C529054	NA	HAVCR1	26762	dapagliflozin inhibits the reaction [[Streptozocin co-treated with Fructose] results in increased expression of HAVCR1 protein]	affects^cotreatment|decreases^reaction|increases^expression	1	1
dapagliflozin	C529054	NA	HAVCR1	26762	dapagliflozin promotes the reaction [Metformin inhibits the reaction [[Streptozocin co-treated with Fructose] results in increased expression of HAVCR1 protein]]	affects^cotreatment|decreases^reaction|increases^expression|increases^reaction	1	1
dapagliflozin	C529054	NA	HAVCR1	26762	Metformin promotes the reaction [dapagliflozin inhibits the reaction [[Streptozocin co-treated with Fructose] results in increased expression of HAVCR1 protein]]	affects^cotreatment|decreases^reaction|increases^expression|increases^reaction	1	1
dapagliflozin	C529054	NA	HMGB1	3146	dapagliflozin inhibits the reaction [Trinitrobenzenesulfonic Acid results in increased expression of HMGB1	decreases^reaction|increases^expression	1	1

					protein]			
dapagliflozin	C529054	NA	HMOX1	3162	[Atorvastatin co-treated with dapagliflozin] inhibits the reaction [[Fructose co-treated with Dietary Fats] results in decreased expression of HMOX1 protein]	affects^cotreatment|decreases^expression|decreases^reaction	1	1
dapagliflozin	C529054	NA	HMOX1	3162	dapagliflozin inhibits the reaction [[Fructose co-treated with Dietary Fats] results in decreased expression of HMOX1 protein]	affects^cotreatment|decreases^expression|decreases^reaction	1	1
dapagliflozin	C529054	NA	HMOX1	3162	dapagliflozin inhibits the reaction [Trinitrobenzenesulfonic Acid results in decreased expression of HMOX1 protein]	decreases^expression|decreases^reaction	1	1
dapagliflozin	C529054	NA	IL10	3586	dapagliflozin inhibits the reaction [Trinitrobenzenesulfonic Acid results in decreased expression of IL10 protein]	decreases^expression|decreases^reaction	1	1
dapagliflozin	C529054	NA	IL1B	3553	[dapagliflozin co-treated with Atorvastatin] inhibits the reaction [[Dietary Fats co-treated with Fructose] results in increased expression of IL1B protein]	affects^cotreatment|decreases^reaction|increases^expression	1	1
dapagliflozin	C529054	NA	INS1	16333	[dapagliflozin co-treated with Atorvastatin] inhibits the reaction [[Dietary Fats co-treated with Fructose] results in increased expression of INS1 protein]	affects^cotreatment|decreases^reaction|increases^expression	1	1
dapagliflozin	C529054	NA	INS1	16333	dapagliflozin inhibits the reaction [[Dietary Fats co-treated with Fructose] results in increased expression of INS1 protein]	affects^cotreatment|decreases^reaction|increases^expression	1	1
dapagliflozin	C529054	NA	INS1	16333	dapagliflozin inhibits the reaction [Dietary Fats results in increased expression of INS1 protein]	decreases^reaction|increases^expression	1	1
dapagliflozin	C529054	NA	INS1	16333	dapagliflozin promotes the reaction [Vildagliptin inhibits the reaction [Dietary Fats results in increased expression of INS1 protein]]	decreases^reaction|increases^expression|increases^reaction	1	1
dapagliflozin	C529054	NA	INSR	3643	[dapagliflozin co-treated with Vildagliptin] inhibits the reaction [Dietary Fats results in decreased phosphorylation of INSR protein]	affects^cotreatment|decreases^phosphorylation|decreases^reaction	1	1
dapagliflozin	C529054	NA	INSR	3643	dapagliflozin inhibits the reaction	decreases^phosphorylat	1	1

					[Dietary Fats results in decreased phosphorylation of INSR protein]	ion|decreases^reaction		
dapagliflozin	C529054	NA	JAK2	3717	dapagliflozin inhibits the reaction [[Dietary Fats co-treated with Streptozocin] results in decreased phosphorylation of JAK2 protein]	affects^cotreatment|decreases^phosphorylation |decreases^reaction	1	1
dapagliflozin	C529054	NA	LCN2	3934	[dapagliflozin co-treated with Atorvastatin] inhibits the reaction [[Fructose co-treated with Dietary Fats] results in increased expression of LCN2 protein]	affects^cotreatment|decreases^reaction|increases^expression	1	1
dapagliflozin	C529054	NA	LCN2	3934	dapagliflozin inhibits the reaction [[Fructose co-treated with Dietary Fats] results in increased expression of LCN2 protein]	affects^cotreatment|decreases^reaction|increases^expression	1	1
dapagliflozin	C529054	NA	LCN2	3934	dapagliflozin inhibits the reaction [[Streptozocin co-treated with Fructose] results in increased expression of LCN2 protein]	affects^cotreatment|decreases^reaction|increases^expression	1	1
dapagliflozin	C529054	NA	MAP1LC3B	81631	[dapagliflozin co-treated with Atorvastatin] inhibits the reaction [[Dietary Fats co-treated with Fructose] results in increased lipidation of MAP1LC3B protein]	affects^cotreatment|decreases^reaction|increases^lipidation	1	1
dapagliflozin	C529054	NA	MPO	4353	dapagliflozin inhibits the reaction [Trinitrobenzenesulfonic Acid results in increased activity of MPO protein]	decreases^reaction|increases^activity	1	1
dapagliflozin	C529054	NA	MTOR	2475	[dapagliflozin co-treated with Atorvastatin] inhibits the reaction [[Dietary Fats co-treated with Fructose] results in increased expression of and results in increased phosphorylation of MTOR protein]	affects^cotreatment|decreases^reaction|increases^expression|increases^phosphorylation	1	1
dapagliflozin	C529054	NA	MTOR	2475	dapagliflozin inhibits the reaction [Trinitrobenzenesulfonic Acid results in increased phosphorylation of MTOR protein]	decreases^reaction|increases^phosphorylation	1	1
dapagliflozin	C529054	NA	NFE2L2	4780	[Atorvastatin co-treated with dapagliflozin] inhibits the reaction [[Fructose co-treated with Dietary Fats] results in increased expression of NFE2L2 protein]	affects^cotreatment|decreases^reaction|increases^expression	1	1
dapagliflozin	C529054	NA	NFE2L2	4780	dapagliflozin inhibits the reaction [[Fructose co-treated with Dietary	affects^cotreatment|decreases^reaction|increas	1	1

					Fats] results in increased expression of NFE2L2 protein]	es^expression		
dapagliflozin	C529054	NA	NFE2L2	4780	dapagliflozin inhibits the reaction [Trinitrobenzenesulfonic Acid affects the expression of NFE2L2 protein]	affects^expression|decreases^reaction	1	1
dapagliflozin	C529054	NA	NLRP3	114548	[dapagliflozin co-treated with Atorvastatin] inhibits the reaction [[Dietary Fats co-treated with Fructose] results in increased expression of NLRP3 protein]	affects^cotreatment|decreases^reaction|increases^expression	1	1
dapagliflozin	C529054	NA	NPHS1	4868	dapagliflozin inhibits the reaction [[Streptozocin co-treated with Fructose] results in decreased expression of NPHS1 mRNA]	affects^cotreatment|decreases^expression|decreases^reaction	1	1
dapagliflozin	C529054	NA	OGA	10724	dapagliflozin inhibits the reaction [[Streptozocin co-treated with Fructose] results in increased expression of and results in increased secretion of OGA protein]	affects^cotreatment|decreases^reaction|increases^expression|increases^secretion	1	1
dapagliflozin	C529054	NA	PLIN2	123	[dapagliflozin co-treated with Atorvastatin] inhibits the reaction [[Dietary Fats co-treated with Fructose] results in increased expression of PLIN2 protein]	affects^cotreatment|decreases^reaction|increases^expression	1	1
dapagliflozin	C529054	NA	PPARA	5465	[dapagliflozin co-treated with Atorvastatin] inhibits the reaction [[Fructose co-treated with Dietary Fats] results in decreased expression of PPARA protein]	affects^cotreatment|decreases^expression|decreases^reaction	1	1
dapagliflozin	C529054	NA	PPARGC1A	10891	[Atorvastatin co-treated with dapagliflozin] inhibits the reaction [[Fructose co-treated with Dietary Fats] results in decreased expression of PPARGC1A protein]	affects^cotreatment|decreases^expression|decreases^reaction	1	1
dapagliflozin	C529054	NA	PPARGC1A	10891	dapagliflozin inhibits the reaction [[Fructose co-treated with Dietary Fats] results in decreased expression of PPARGC1A protein]	affects^cotreatment|decreases^expression|decreases^reaction	1	1
dapagliflozin	C529054	NA	PRKCA	5578	[Atorvastatin co-treated with dapagliflozin] inhibits the reaction [[Fructose co-treated with Dietary Fats] results in increased expression of and results in increased phosphorylation of PRKCA protein]	affects^cotreatment|decreases^reaction|increases^expression|increases^phosphorylation	1	1

dapagliflozin	C529054	NA	PRKCZ	5590	[Atorvastatin co-treated with dapagliflozin] inhibits the reaction [[Fructose co-treated with Dietary Fats] results in decreased expression of PRKCZ protein]	affects^cotreatment|decreases^expression|decreases^reaction	1	1
dapagliflozin	C529054	NA	PRKCZ	5590	dapagliflozin inhibits the reaction [[Fructose co-treated with Dietary Fats] results in decreased expression of PRKCZ protein]	affects^cotreatment|decreases^expression|decreases^reaction	1	1
dapagliflozin	C529054	NA	PTGS2	5743	[dapagliflozin co-treated with Atorvastatin] inhibits the reaction [[Dietary Fats co-treated with Fructose] results in increased expression of PTGS2 protein]	affects^cotreatment|decreases^reaction|increases^expression	1	1
dapagliflozin	C529054	NA	RELA	5970	[dapagliflozin co-treated with Atorvastatin] inhibits the reaction [[Dietary Fats co-treated with Fructose] results in increased expression of RELA protein]	affects^cotreatment|decreases^reaction|increases^expression	1	1
dapagliflozin	C529054	NA	RELA	5970	[dapagliflozin co-treated with Vildagliptin] inhibits the reaction [Dietary Fats results in increased phosphorylation of RELA protein]	affects^cotreatment|decreases^reaction|increases^phosphorylation	1	1
dapagliflozin	C529054	NA	RELA	5970	dapagliflozin inhibits the reaction [Dietary Fats results in increased phosphorylation of RELA protein]	decreases^reaction|increases^phosphorylation	1	1
dapagliflozin	C529054	NA	RELA	5970	dapagliflozin inhibits the reaction [Trinitrobenzenesulfonic Acid results in increased phosphorylation of RELA protein]	decreases^reaction|increases^phosphorylation	1	1
dapagliflozin	C529054	NA	SIRT1	23411	[dapagliflozin co-treated with Atorvastatin] inhibits the reaction [[Dietary Fats co-treated with Fructose] results in decreased expression of SIRT1 protein]	affects^cotreatment|decreases^expression|decreases^reaction	1	1
dapagliflozin	C529054	NA	SQSTM1	8878	[dapagliflozin co-treated with Atorvastatin] inhibits the reaction [[Dietary Fats co-treated with Fructose] results in increased expression of SQSTM1 protein]	affects^cotreatment|decreases^reaction|increases^expression	1	1
dapagliflozin	C529054	NA	SQSTM1	8878	dapagliflozin inhibits the reaction [Trinitrobenzenesulfonic Acid results in increased expression of SQSTM1 protein]	decreases^reaction|increases^expression	1	1

dapagliflozin	C529054	NA	SREBF1	6720	[Atorvastatin co-treated with dapagliflozin] inhibits the reaction [[Fructose co-treated with Dietary Fats] results in increased expression of SREBF1 protein]	affects^cotreatment|decreases^reaction|increases^expression	1	1
dapagliflozin	C529054	NA	SREBF1	6720	dapagliflozin inhibits the reaction [[Fructose co-treated with Dietary Fats] results in increased expression of SREBF1 protein]	affects^cotreatment|decreases^reaction|increases^expression	1	1
dapagliflozin	C529054	NA	STAT3	6774	dapagliflozin inhibits the reaction [Doxorubicin results in decreased expression of STAT3 protein]	decreases^expression|decreases^reaction	1	1
dapagliflozin	C529054	NA	STAT3	6774	dapagliflozin results in increased expression of STAT3 protein	increases^expression	1	1
dapagliflozin	C529054	NA	STAT3	6774	STAT3 protein promotes the reaction [dapagliflozin inhibits the reaction [Doxorubicin results in decreased expression of BCL2 protein]]	decreases^expression|decreases^reaction|increases^reaction	1	1
dapagliflozin	C529054	NA	TGFB1	7040	dapagliflozin inhibits the reaction [[Streptozocin co-treated with Fructose] results in increased expression of and results in increased secretion of TGFB1 protein]	affects^cotreatment|decreases^reaction|increases^expression|increases^secretion	1	1
dapagliflozin	C529054	NA	TGFB1	7040	dapagliflozin promotes the reaction [Metformin inhibits the reaction [[Streptozocin co-treated with Fructose] results in increased expression of and results in increased secretion of TGFB1 protein]]	affects^cotreatment|decreases^reaction|increases^expression|increases^reaction|increases^secretion	1	1
dapagliflozin	C529054	NA	TNF	7124	[dapagliflozin co-treated with Atorvastatin] inhibits the reaction [[Dietary Fats co-treated with Fructose] results in increased expression of TNF protein]	affects^cotreatment|decreases^reaction|increases^expression	1	1
dapagliflozin	C529054	NA	TNF	7124	dapagliflozin inhibits the reaction [[Dietary Fats co-treated with Fructose] results in increased expression of TNF protein]	affects^cotreatment|decreases^reaction|increases^expression	1	1
dapagliflozin	C529054	NA	TNF	7124	dapagliflozin inhibits the reaction [[Dietary Fats co-treated with Streptozocin] results in increased expression of TNF protein]	affects^cotreatment|decreases^reaction|increases^expression	1	1
dapagliflozin	C529054	NA	TNF	7124	dapagliflozin inhibits the reaction [[Streptozocin co-treated with	affects^cotreatment|decreases^reaction|increas	1	1

					Fructose] results in increased expression of and results in increased secretion of TNF protein]	es^expression|increases ^secretion		
dapagliflozin	C529054	NA	TNF	7124	dapagliflozin inhibits the reaction [Trinitrobenzenesulfonic Acid results in increased expression of TNF protein]	decreases^reaction|increases^expression	1	1
dapagliflozin	C529054	NA	TNF	7124	dapagliflozin promotes the reaction [Metformin inhibits the reaction [[Streptozocin co-treated with Fructose] results in increased expression of and results in increased secretion of TNF protein]]	affects^cotreatment|decreases^reaction|increases^expression|increases^reaction|increases^secretion	1	1
dapagliflozin	C529054	NA	TP53	7157	dapagliflozin inhibits the reaction [[Dietary Fats co-treated with Streptozocin] results in increased expression of TP53 protein]	affects^cotreatment|decreases^reaction|increases^expression	1	1
dapagliflozin	C529054	NA	VIM	7431	[dapagliflozin co-treated with Atorvastatin] inhibits the reaction [[Dietary Fats co-treated with Fructose] results in increased expression of VIM protein]	affects^cotreatment|decreases^reaction|increases^expression	1	1
dapagliflozin	C529054	NA	VNN1	8876	dapagliflozin inhibits the reaction [[Streptozocin co-treated with Fructose] results in increased expression of VNN1 mRNA]	affects^cotreatment|decreases^reaction|increases^expression	1	1
dapagliflozin	C529054	NA	WT1	7490	[dapagliflozin co-treated with Atorvastatin] inhibits the reaction [[Dietary Fats co-treated with Fructose] results in decreased expression of WT1 protein]	affects^cotreatment|decreases^expression|decreases^reaction	1	1
empagliflozin	C570240	NA	BECN1	8678	empagliflozin inhibits the reaction [Rotenone results in decreased expression of BECN1 protein]	decreases^expression|decreases^reaction	1	1
empagliflozin	C570240	NA	CAT	847	empagliflozin inhibits the reaction [Rotenone results in decreased activity of CAT protein]	decreases^activity|decreases^reaction	1	1
empagliflozin	C570240	NA	DDIT3	1649	empagliflozin inhibits the reaction [Rotenone results in increased expression of DDIT3 mRNA]	decreases^reaction|increases^expression	1	1
empagliflozin	C570240	NA	EIF2AK3	9451	empagliflozin inhibits the reaction [Rotenone results in increased phosphorylation of EIF2AK3 protein]	decreases^reaction|increases^phosphorylation	1	1
empagliflozin	C570240	NA	ELF2	1998	empagliflozin inhibits the reaction	decreases^expression|d	1	1

					[Rotenone results in decreased expression of ELF2 mRNA]	ecreases^reaction		
empagliflozin	C570240	NA	HSPA5	3309	empagliflozin inhibits the reaction [Rotenone results in increased expression of HSPA5 protein]	decreases^reaction|increases^expression	1	1
empagliflozin	C570240	NA	IL1B	3553	empagliflozin inhibits the reaction [Rotenone results in increased expression of IL1B protein]	decreases^reaction|increases^expression	1	1
empagliflozin	C570240	NA	MIR211	406993	empagliflozin inhibits the reaction [Rotenone results in increased expression of MIR211 mRNA]	decreases^reaction|increases^expression	1	1
empagliflozin	C570240	NA	PRKG1	5592	empagliflozin results in increased expression of PRKG1 protein	increases^expression	1	1
empagliflozin	C570240	NA	SNCA	6622	empagliflozin inhibits the reaction [Rotenone results in increased expression of SNCA protein]	decreases^reaction|increases^expression	1	1
