# Supplementary material for: Pharmacological targets of SGLT2 inhibitors on IgA nephropathy and membranous nephropathy: a mendelian randomization study
Source: Front Pharmacol. 2024 May 22;15:1399881. doi: 10.3389/fphar.2024.1399881 (PMC11155304; doi:10.3389/fphar.2024.1399881)
Supplement: Supplementary file 2 [file DataSheet1.PDF]

Pharmacological targets of SGLT2 inhibitors on IgA nephropathy and membranous nephropathy: a Mendelian randomization study

Supplementary Figures Contents:

Supplementary Figure 1 Scatter plots of Mendelian randomization (MR) analysis for the association between 21 drug targets (MPO, VIM, CDH1, SREBF1, PTGS2, SIRT1, JAK2, ACTA2, ATF6, IL1B, CD36, MAP1LC3B, FASN, INSR, BCL2, DDIT3, PPARA, OGA, MTOR, AGER, and TNF) and membranous nephropathy…..….P.1-21

Supplementary Figure 2 Forest plot of MR estimates between 21 drug targets and membranous nephropathy ……………………………………………………….P.22-42

Supplementary Figure 3 Funnel plots of MR estimates for the effect of 21 drug targets on the risk of membranous nephropathy ………………………………………..P.23-63

Supplementary Figure 4 Leave-One-Out plot for sensitivity test for the effect of 21 drug targets on membranous nephropathy. The dot and bar indicate the estimate and 95% confidence interval when a specific single nucleotide polymorphism is removed ……………………………………………………………………….P.64-84

Supplementary Figure 5 Scatter plots of MR analysis for the association between 11 drug targets (MPO, SREBF1, JAK2, ELF2, ATF6, CAT, CD36, LCN2, CASP7, AGER, and TNF) and the risk of IgA nephropathy ……………………………………..P.85-95

Supplementary Figure 6 Forest plot of MR estimates for the relationship between 11 drug targets and IgA nephropathy……………………………………………….96-106

Supplementary Figure 7 Funnel plots of MR estimates for the effect of 11 drug targets on the risk of IgA nephropathy ……………………………………………….P.107-117

Supplementary Figure 8 Leave-One-Out plot for sensitivity test of the effect of 11 drug targets on IgA nephropathy. The dot and bar indicate the estimate and 95% CI when a specific SNP is removed …………………………………………..P.118-128

Supplementary Figure 9 Scatter plots of MR analysis for the association between LCN2 and the risk of 7 other diseases (toxic liver disease, ulcerative colitis, rheumatoid arthritis, hypertension, pancreatic cancer, osteoarthritis, and dementia with Lewy bodies) ……………………………………………….P.129-135

Supplementary Figure 10 Forest plot of MR estimates for the relationship between LCN2 and 7 other diseases …………………………………………………..P.136-142

Supplementary Figure 11 Funnel plots of MR estimates for the effect of LCN2 on the risk of 7 other diseases ……………………………………………………...P.143-149

Supplementary Figure 12 Leave-One-Out plot for sensitivity test of the effect of LCN2 on 7 other diseases. The dot and bar indicate the estimate and 95% CI when a specific SNP is removed…………………………………………………P.150-156

Supplementary Figure 13 Scatter plots of the MR analysis for the association between AGER and the risk of 8 other diseases (toxic liver disease, obesity, celiac disease, atherosclerosis, excluding cerebral, coronary and PAD, Asthma, Pancreatic cancer, Neuralgia and neuritis, unspecified and Emphysema) …. P.157-164

Supplementary Figure 14 Forest plot of MR estimates between AGER and 8 other diseases……………………………………………………………………P.165-172

Supplementary Figure 15 Funnel plot of MR estimates for the effect of AGER on the risk of 8 other diseases ………………………………………………………P.173-180

Supplementary Figure 16 Leave-One-Out plot for sensitivity test for the effect of AGER on 8 other diseases. The dot and bar represent the estimate and 95% CI when a specific SNP is removed …………………………………………….P.181-188

Supplementary Figure 1 : ACTA2

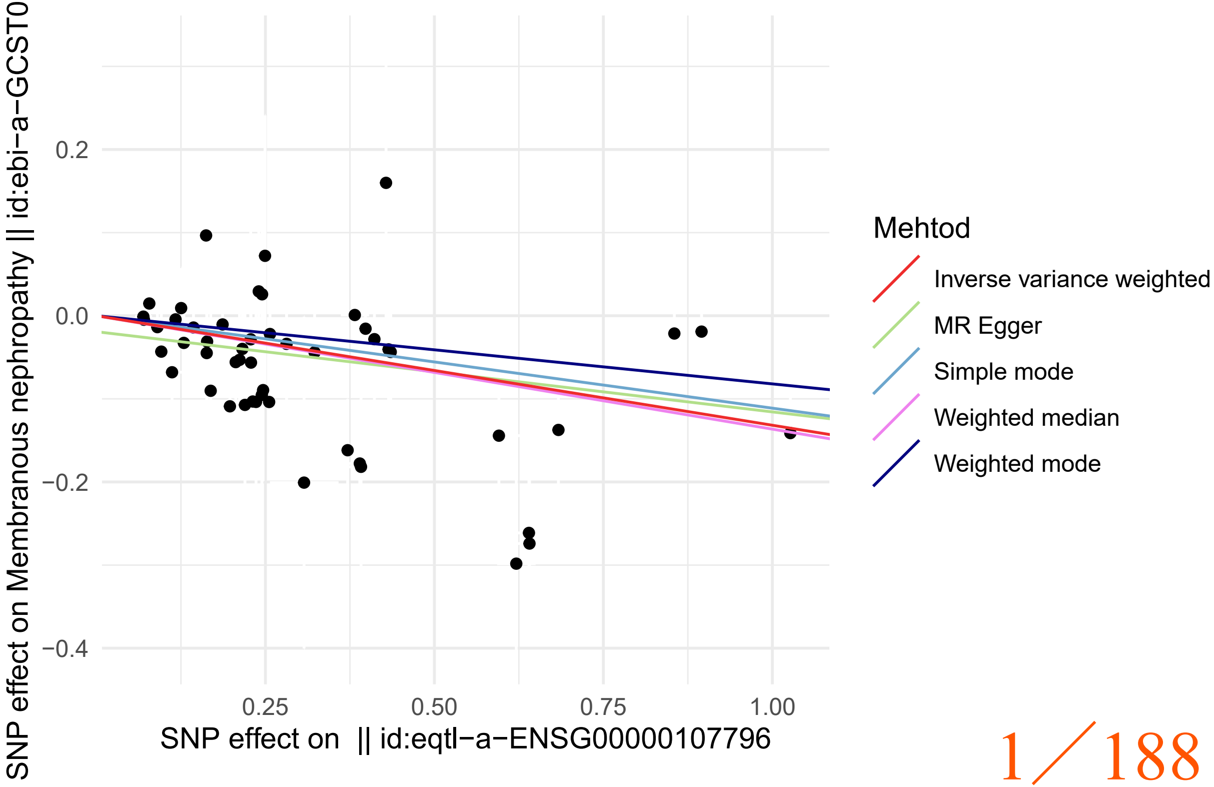

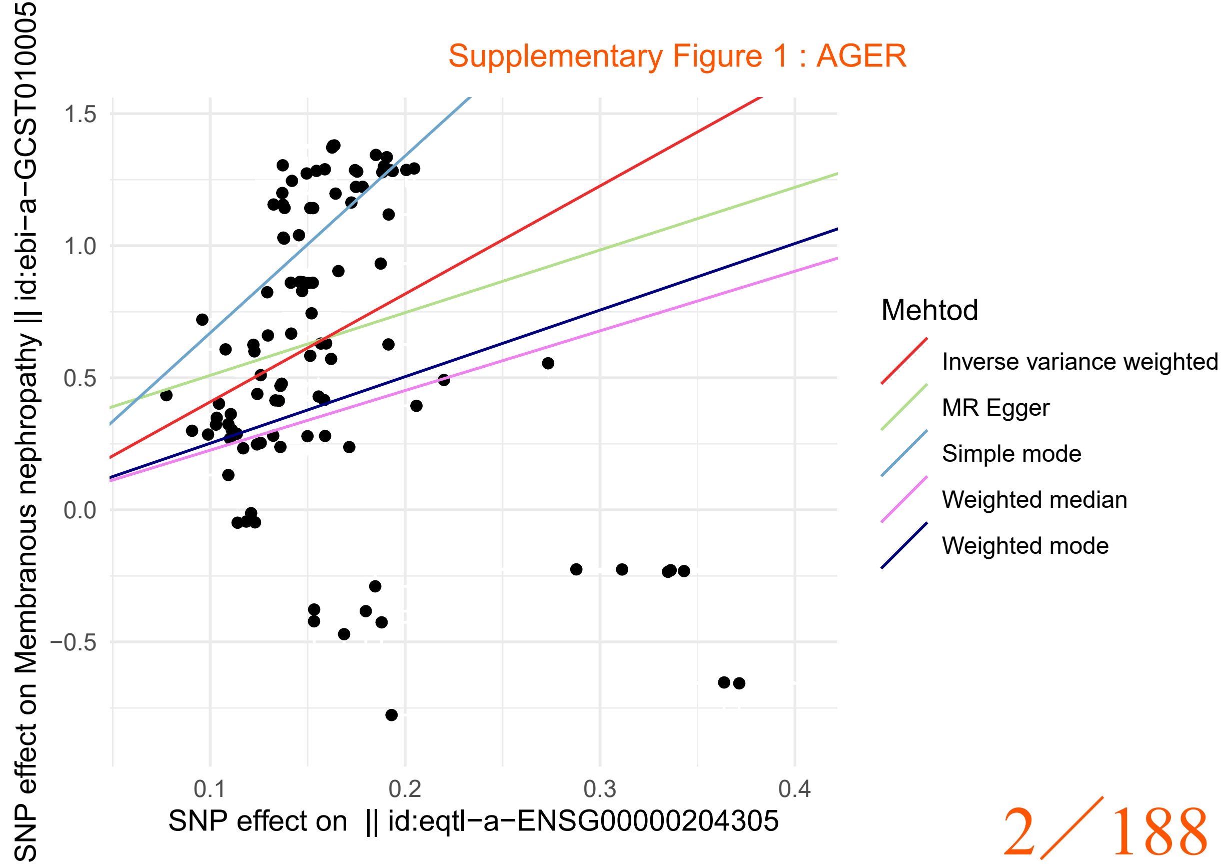
Supplementary Figure 1 : AGER
SNP effect on Membranous nephropathy || id:ebi−a−GCST010005
1.5
1.0
0.5
0.0
−0.5
0.1
0.2
0.3
0.4
SNP effect on || id:eqtl−a−ENSG00000204305
Mehtod
Inverse variance weighted
MR Egger
Simple mode
Weighted median
Weighted mode

Supplementary Figure 1 : ATF6

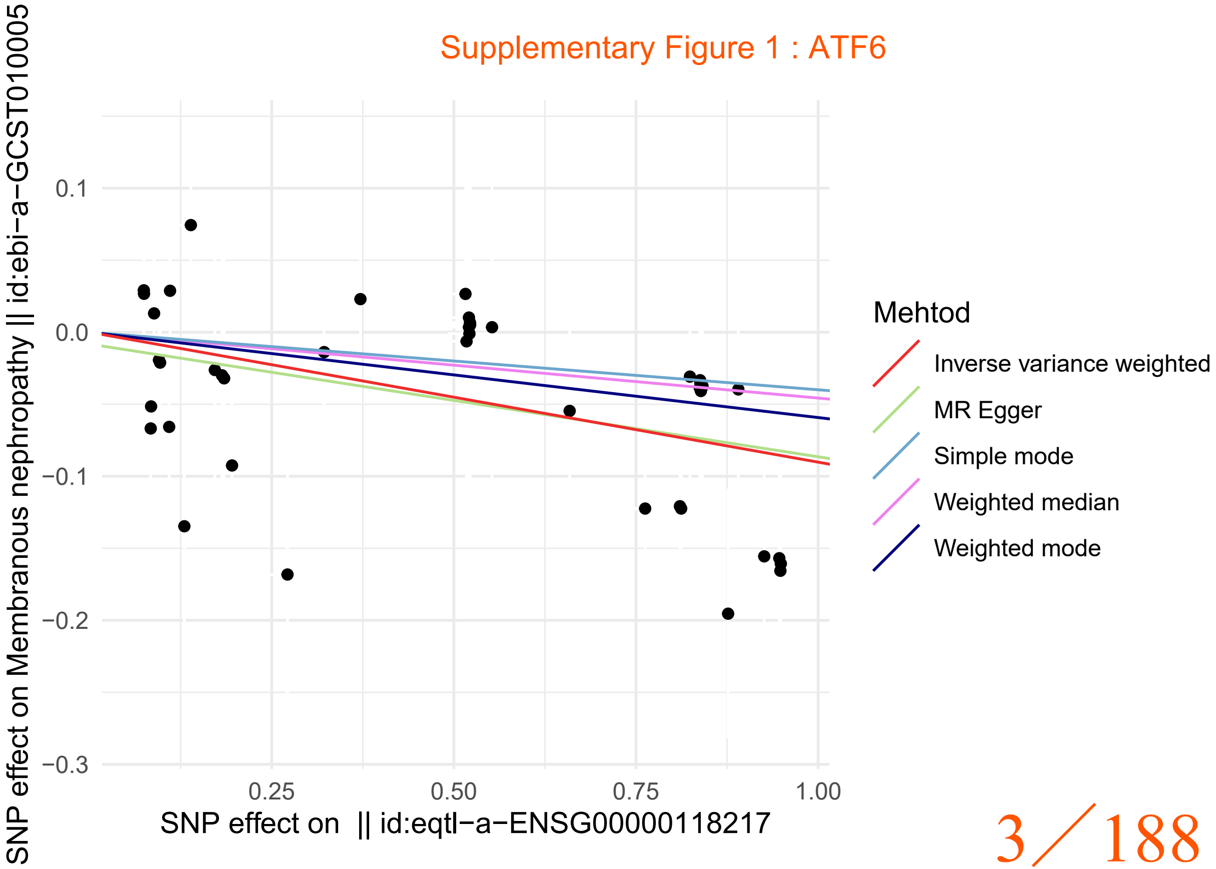

Supplementary Figure 1 : BCL2

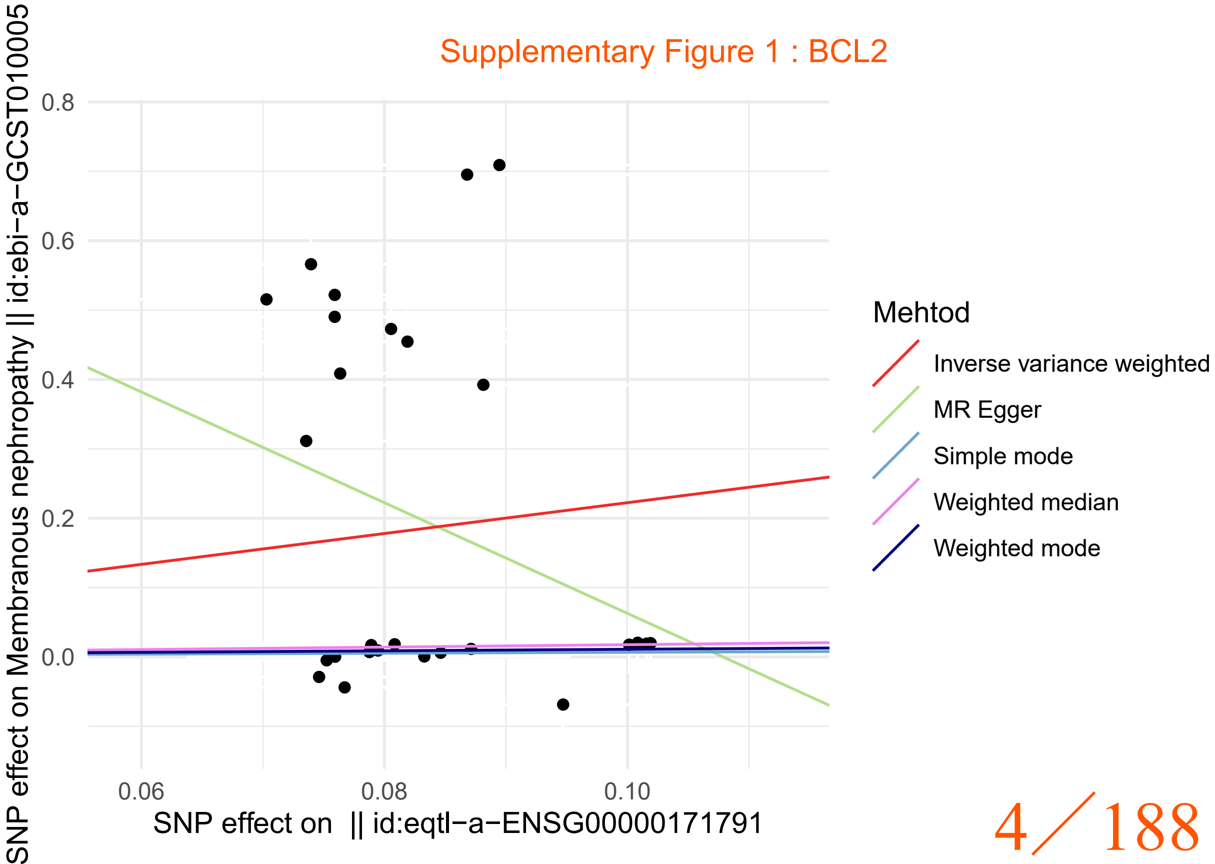

Supplementary Figure 1 : CD36

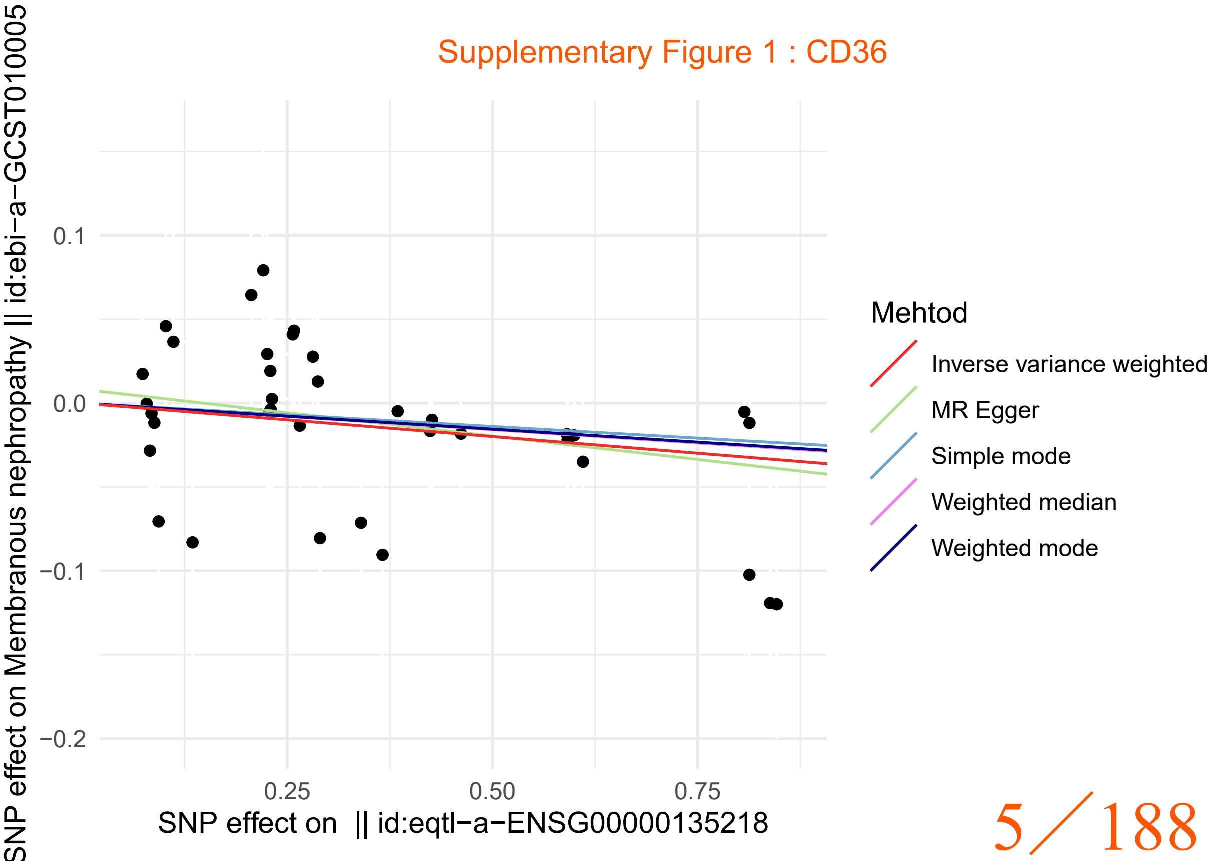

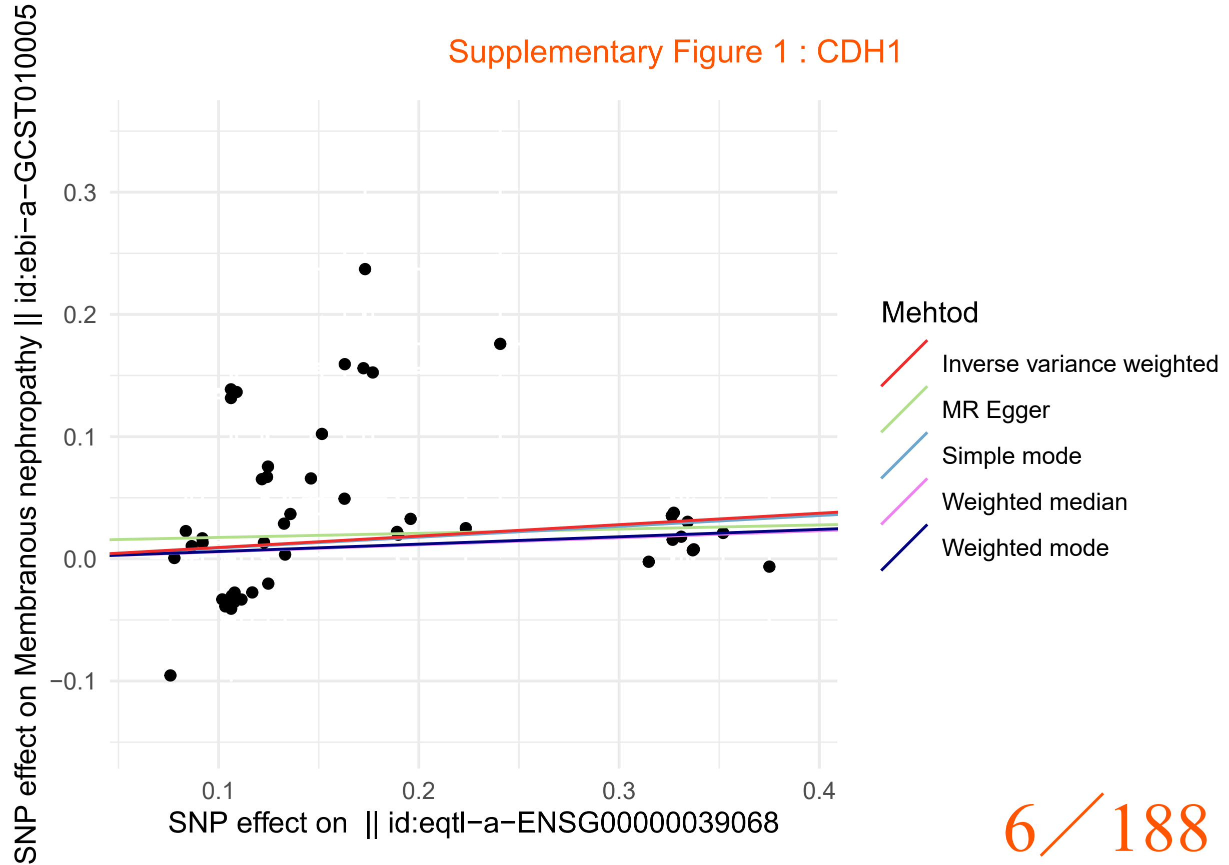
Supplementary Figure 1 : CDH1
SNP effect on Membranous nephropathy || id:ebi−a−GCST010005
0.3
0.2
0.1
0.0
−0.1
0.1
0.2
0.3
0.4
SNP effect on || id:eqtl−a−ENSG00000039068
Mehtod
Inverse variance weighted
MR Egger
Simple mode
Weighted median
Weighted mode

Supplementary Figure 1 : DDIT3

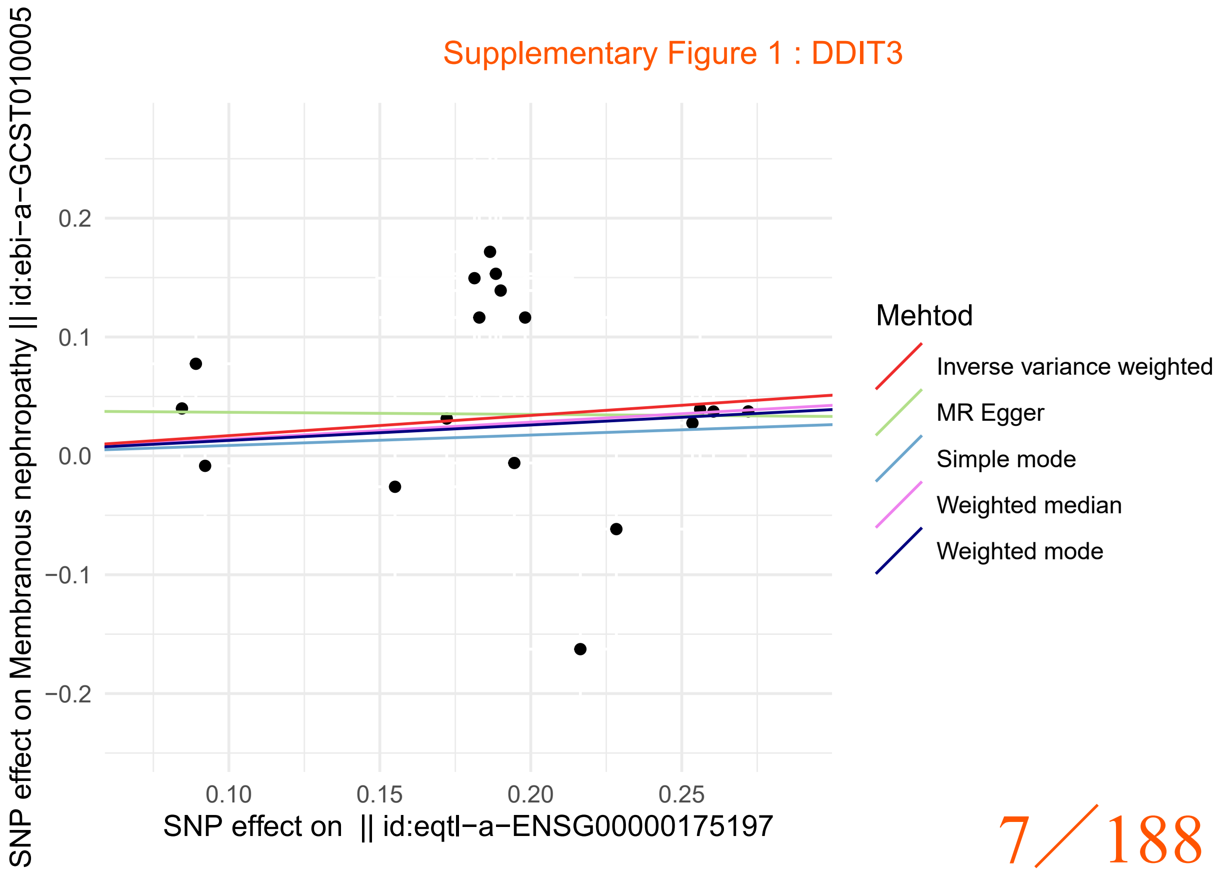

Supplementary Figure 1 : FASN

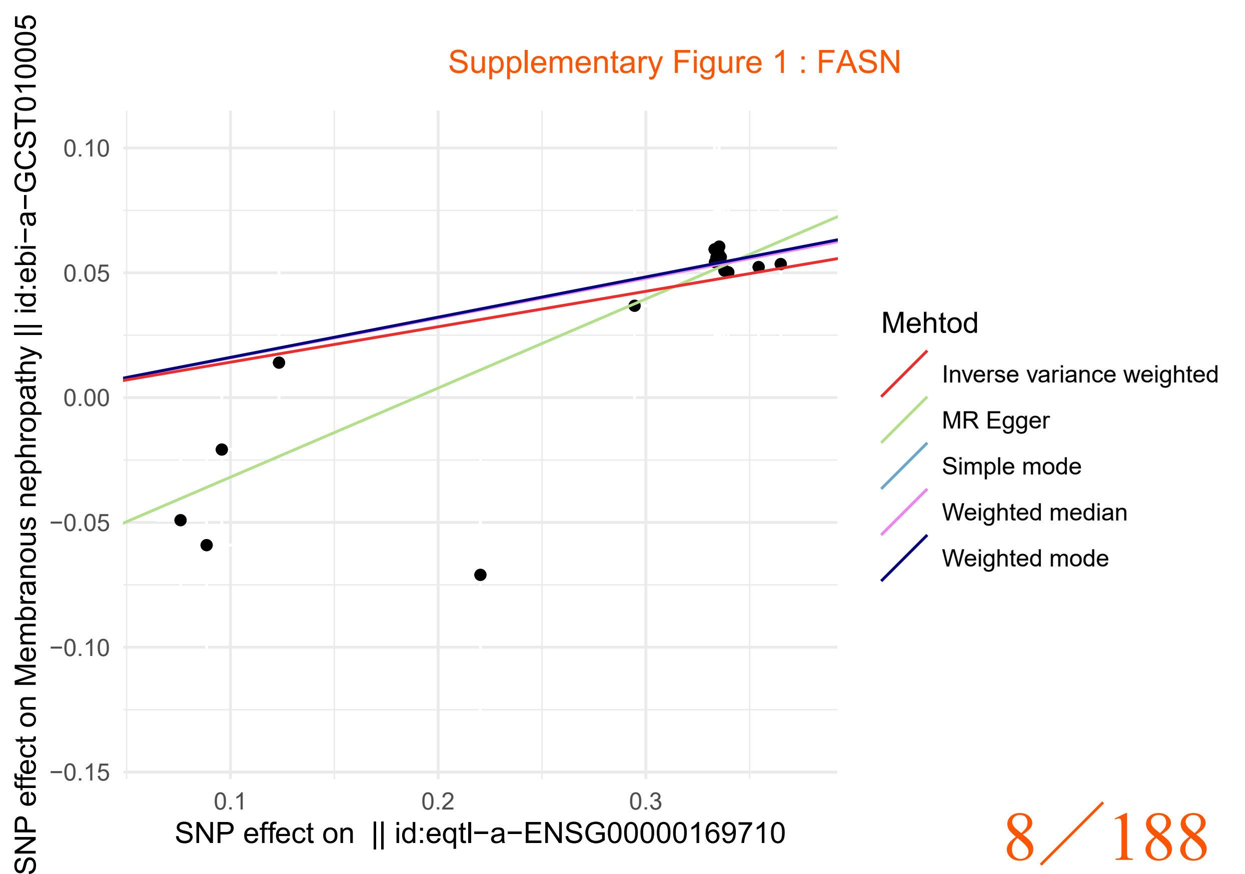

Supplementary Figure 1 : IL1B

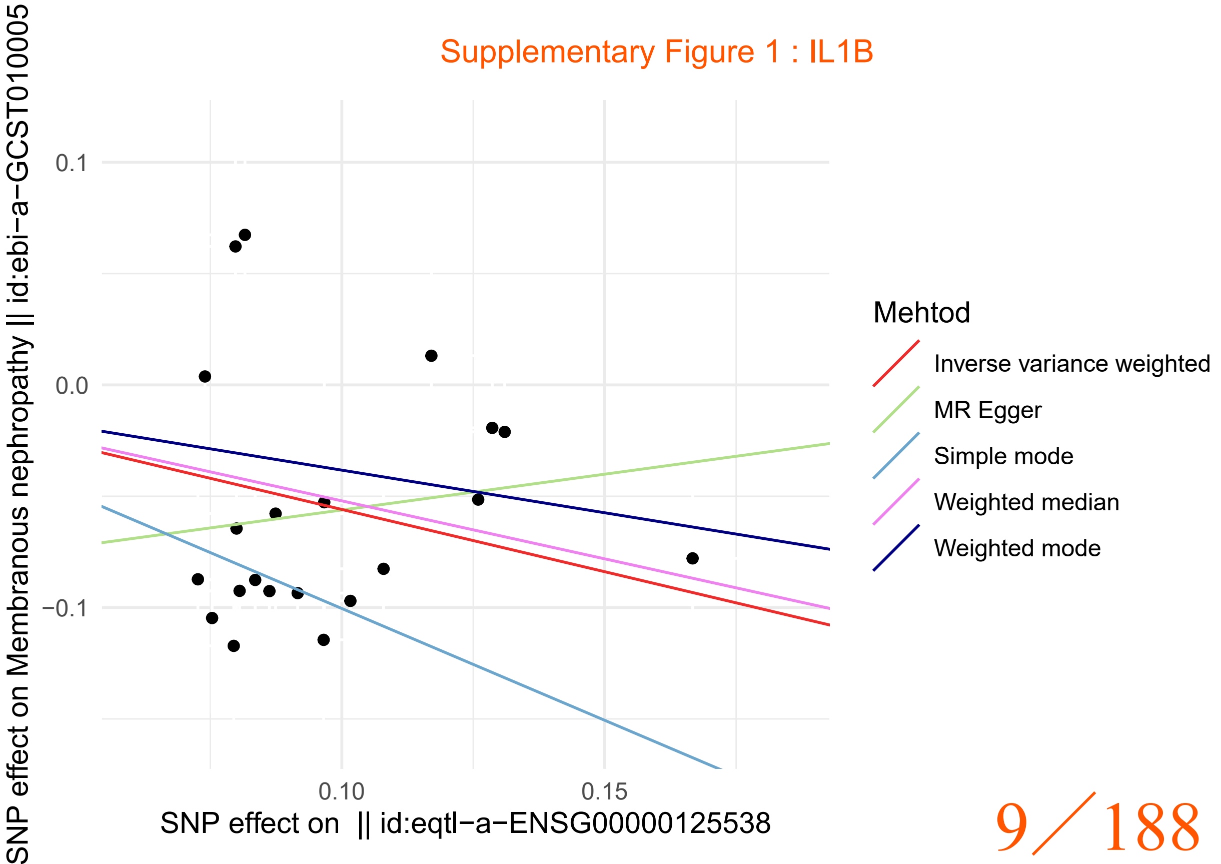

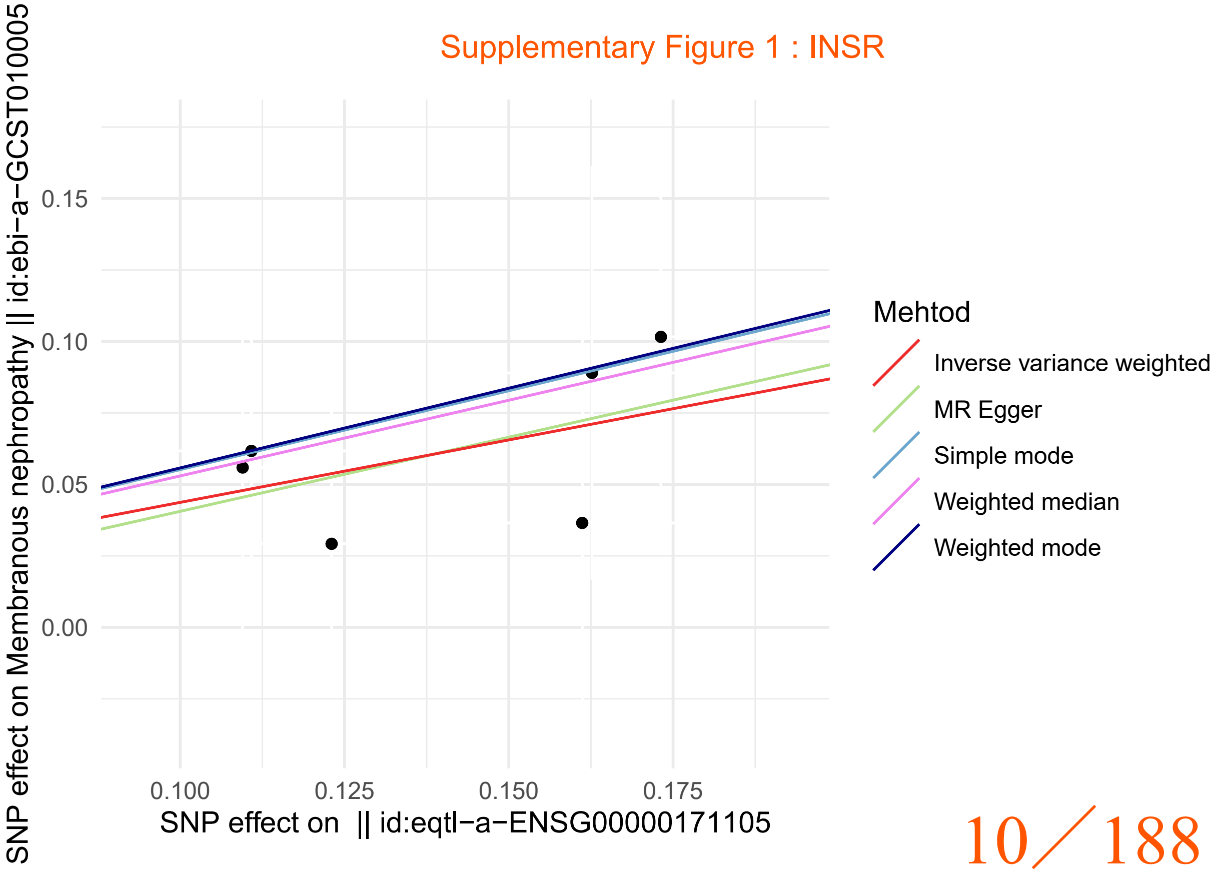
Supplementary Figure 1 : INSR
SNP effect on Membranous nephropathy || id:ebi−a−GCST010005
0.15
0.10
0.05
0.00
0.100
0.125
0.150
0.175
SNP effect on || id:eqtl−a−ENSG00000171105
Mehtod
Inverse variance weighted
MR Egger
Simple mode
Weighted median
Weighted mode

Supplementary Figure 1 : JAK2

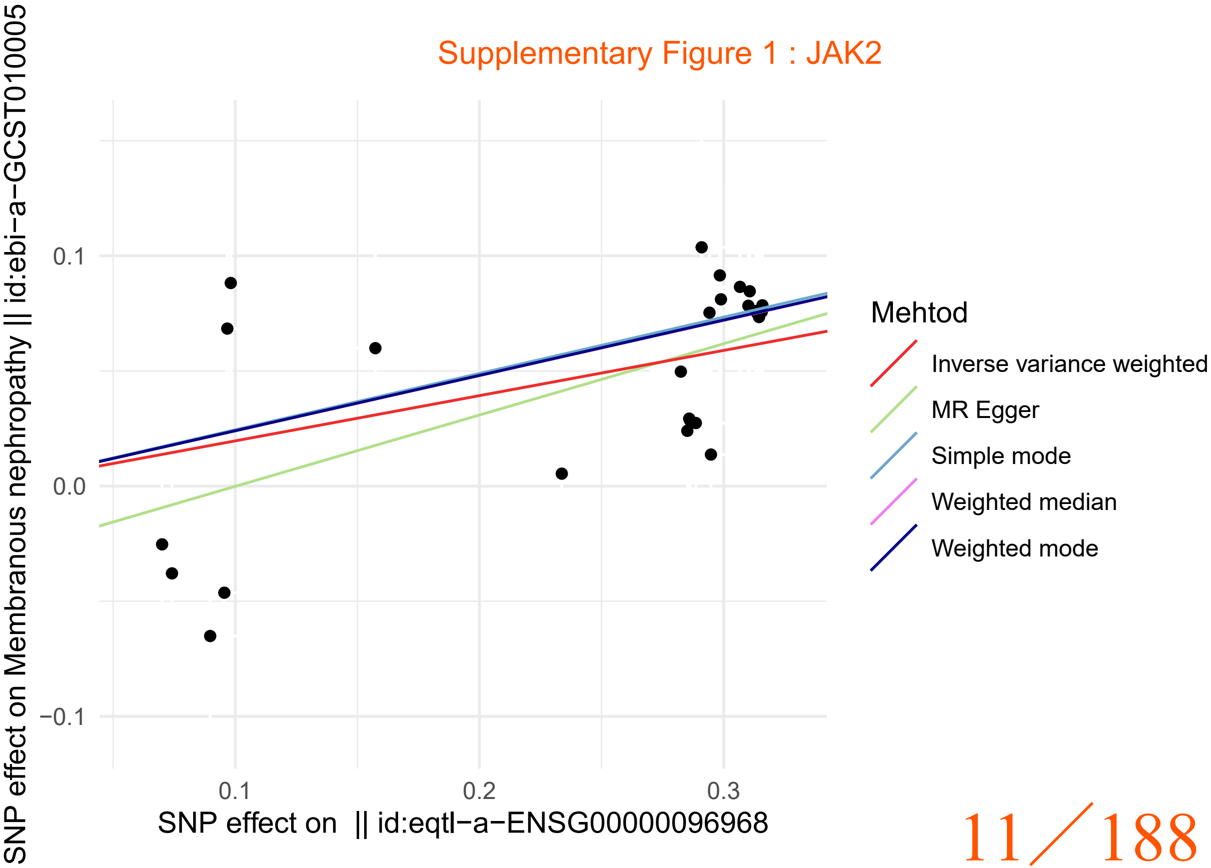

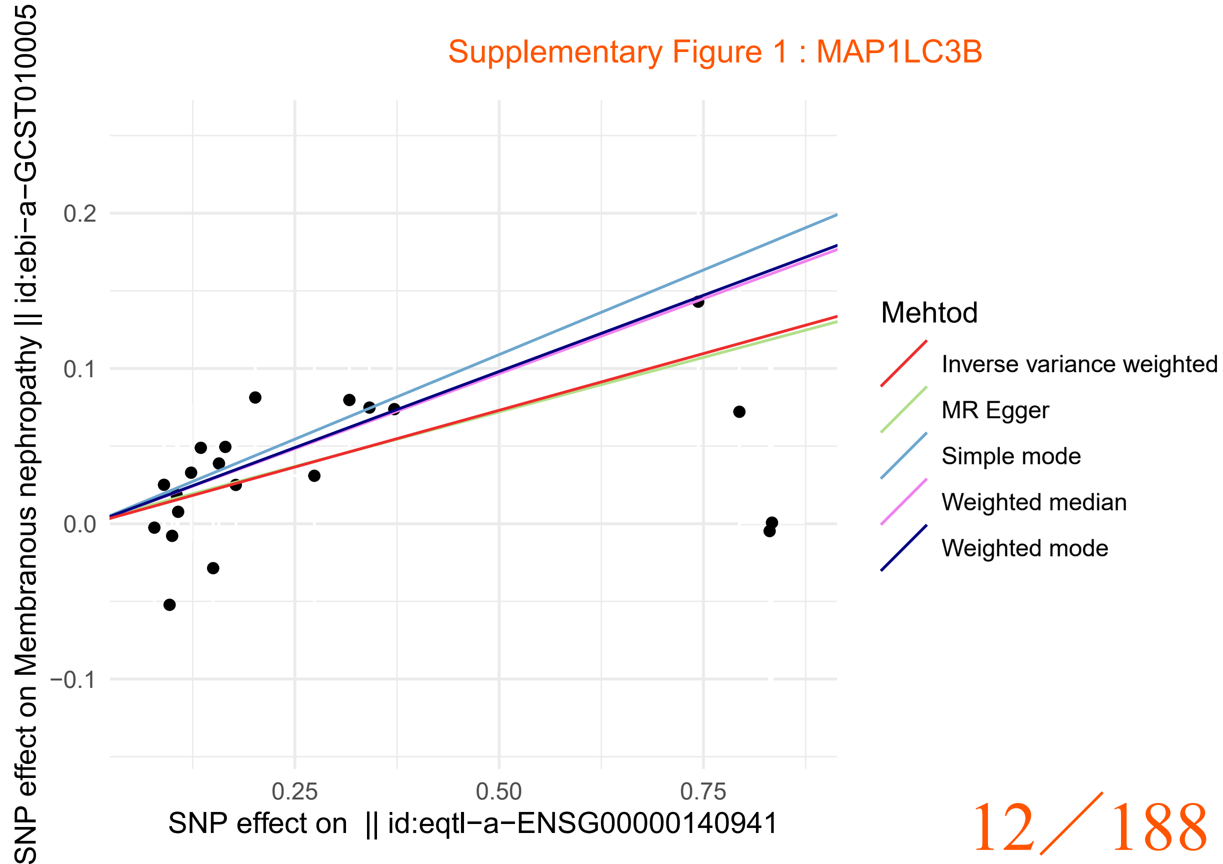
Supplementary Figure 1 : MAP1LC3B
SNP effect on Membranous nephropathy || id:ebi−a−GCST010005
SNP effect on || id:eqtl−a−ENSG00000140941
0.2
0.1
0.0
−0.1
0.25
0.50
0.75
Mehtod
Inverse variance weighted
MR Egger
Simple mode
Weighted median
Weighted mode

Supplementary Figure 1 : MPO

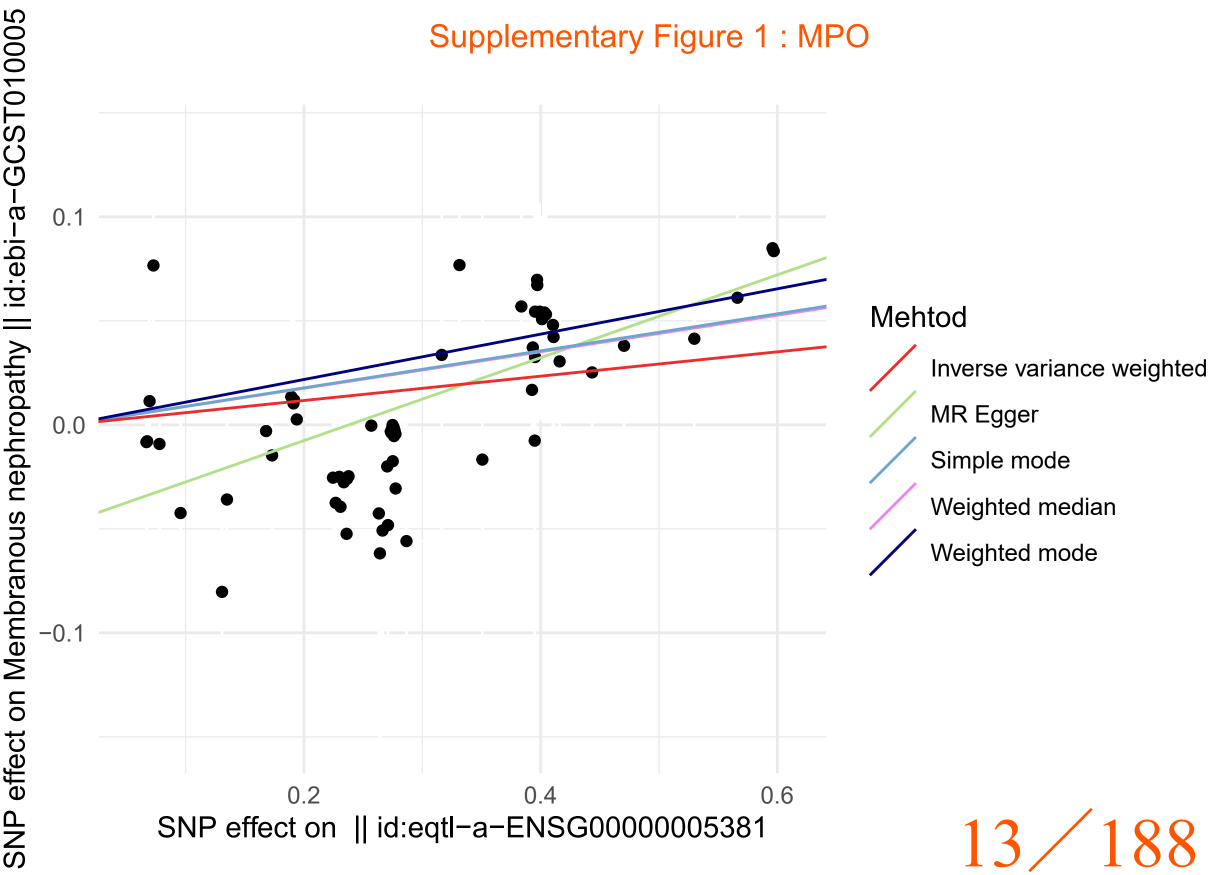

Supplementary Figure 1 : MTOR

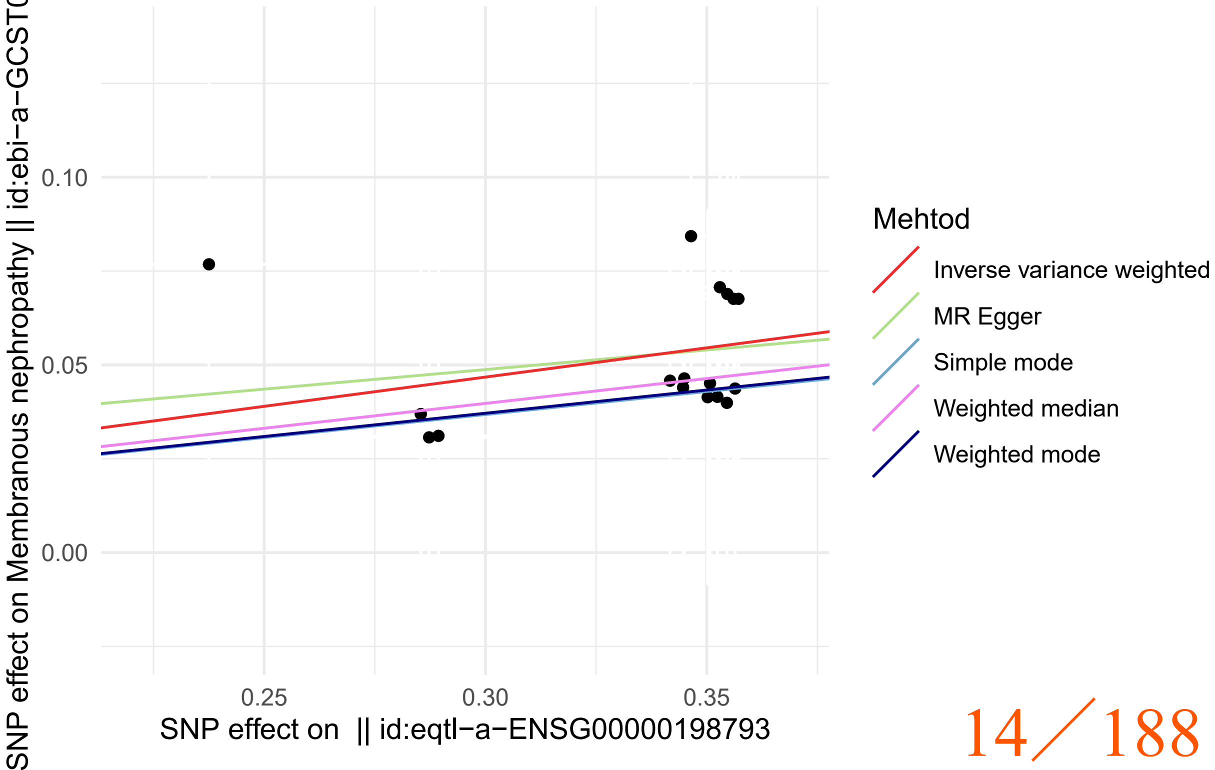

Supplementary Figure 1 : OGA

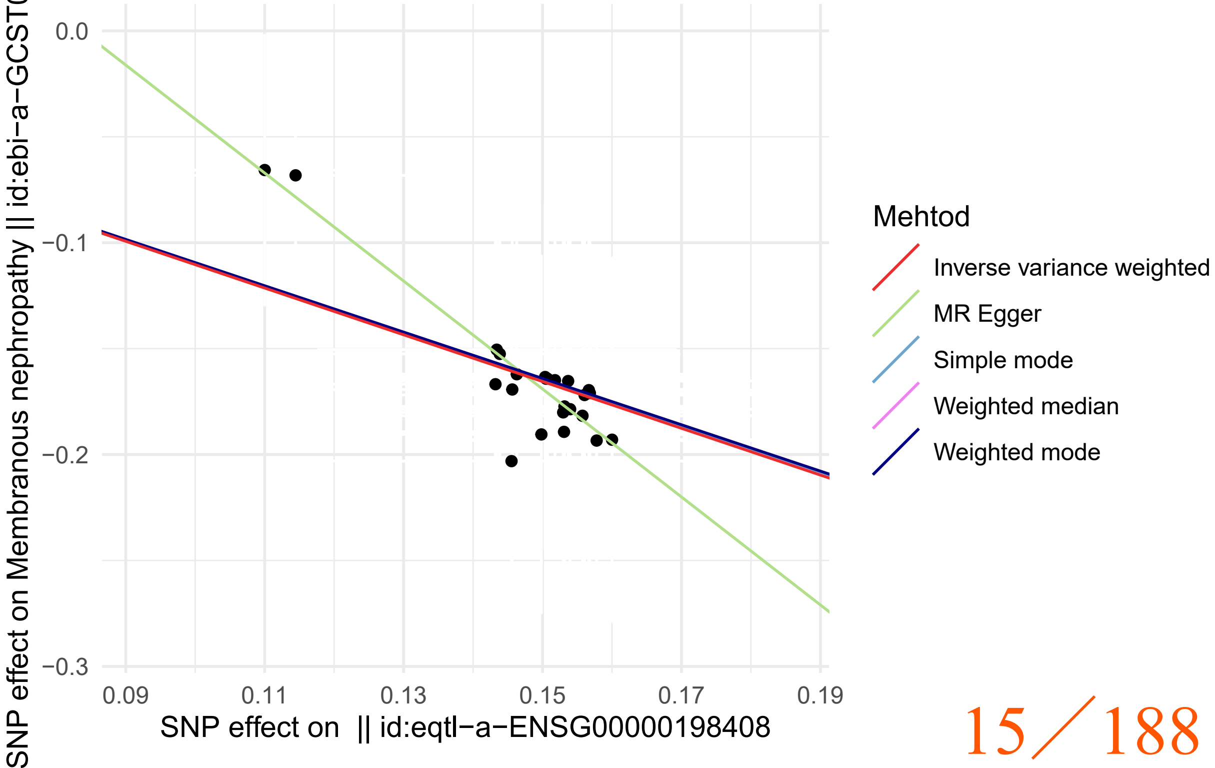

Supplementary Figure 1 : PPARA

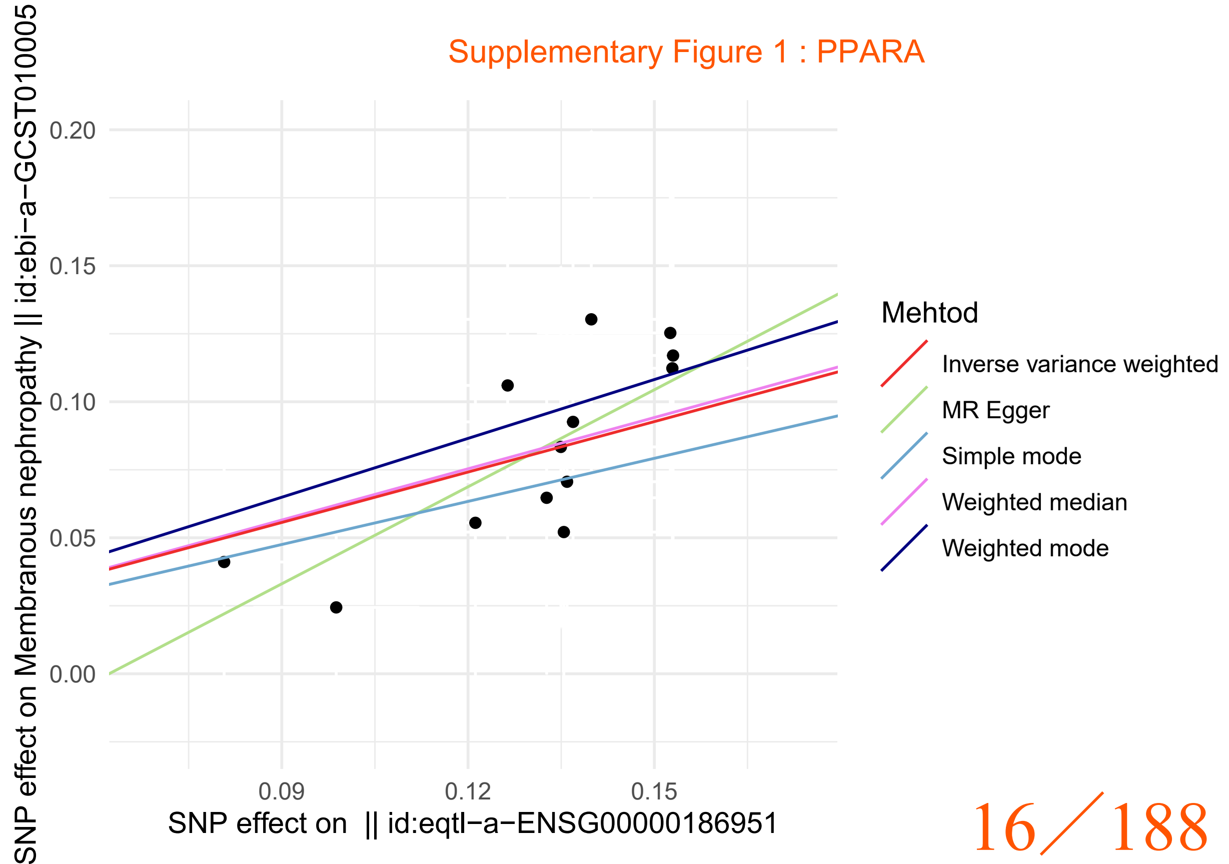

Supplementary Figure 1 : PTGS2

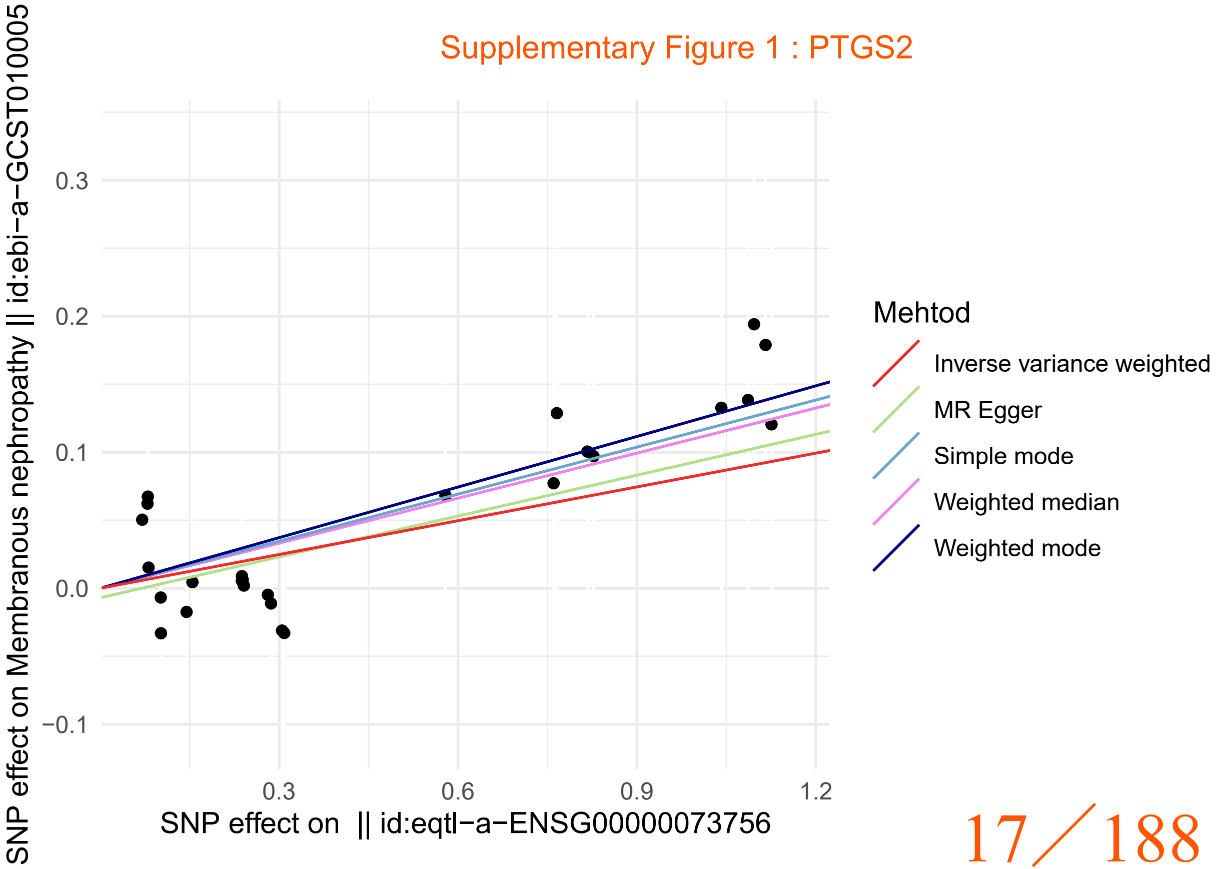

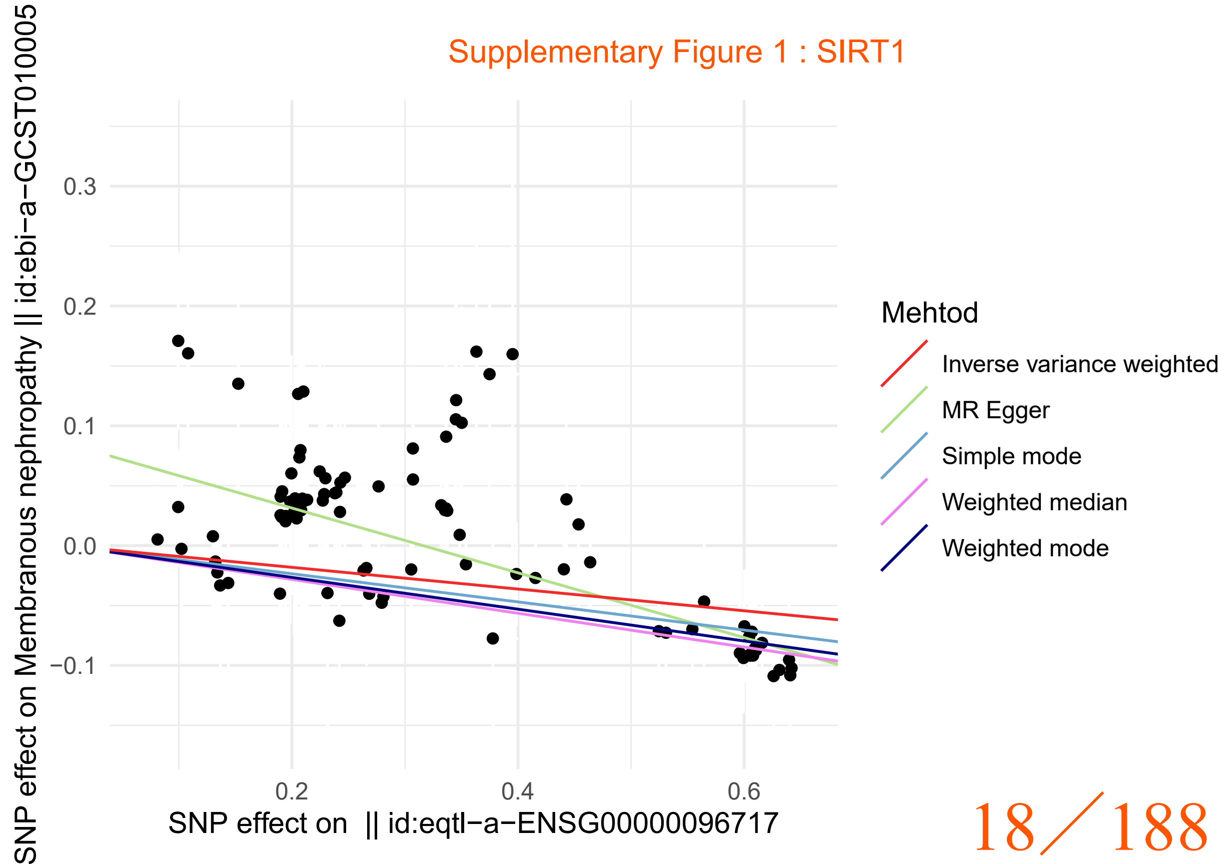

Supplementary Figure 1 : SIRT1
SNP effect on Membranous nephropathy || id:ebi−a−GCST010005
0.3
0.2
0.1
0.0
−0.1
0.2
0.4
0.6
SNP effect on || id:eqtl−a−ENSG00000096717
Mehtod
Inverse variance weighted
MR Egger
Simple mode
Weighted median
Weighted mode

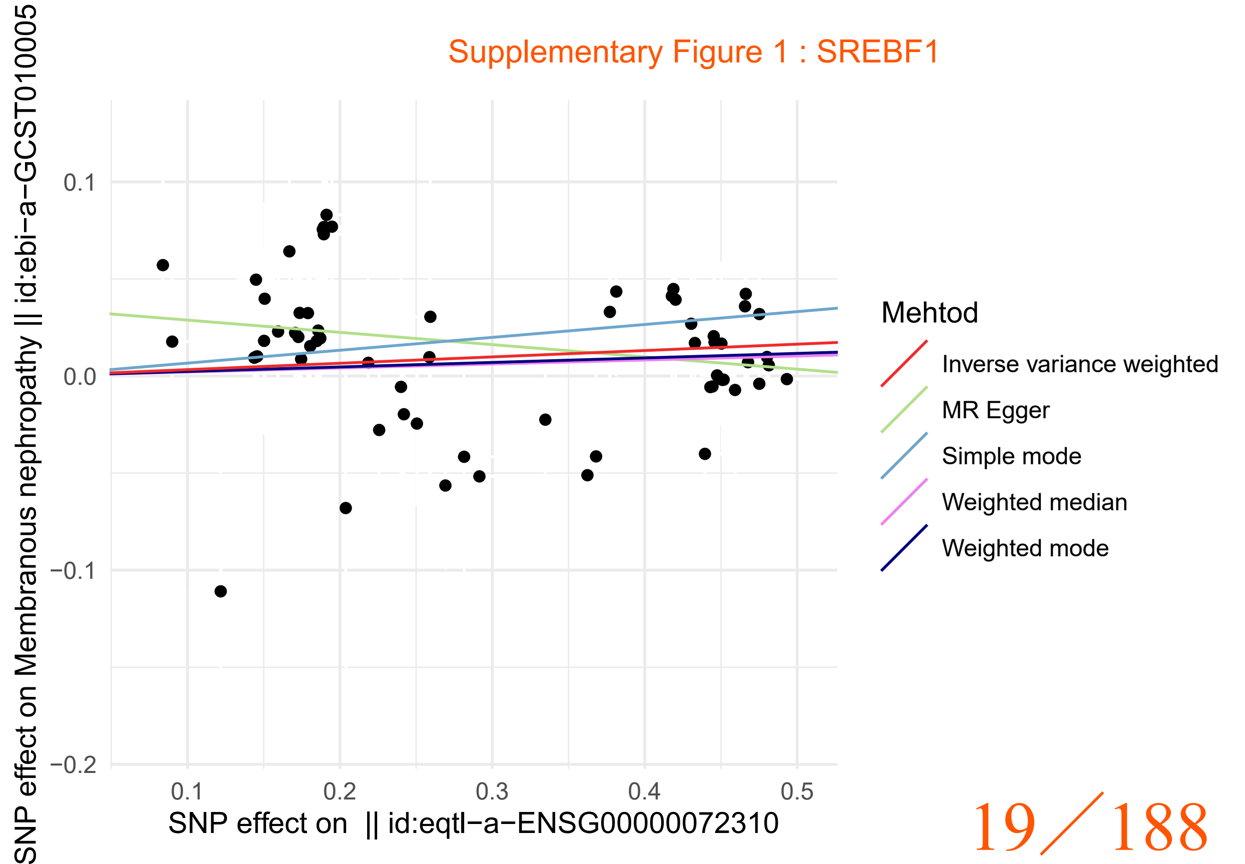
Supplementary Figure 1 : SREBF1
SNP effect on Membranous nephropathy || id:ebi−a−GCST010005
SNP effect on || id:eqtl−a−ENSG00000072310
0.1
0.0
−0.1
−0.2
0.1
0.2
0.3
0.4
0.5
Mehtod
Inverse variance weighted
MR Egger
Simple mode
Weighted median
Weighted mode

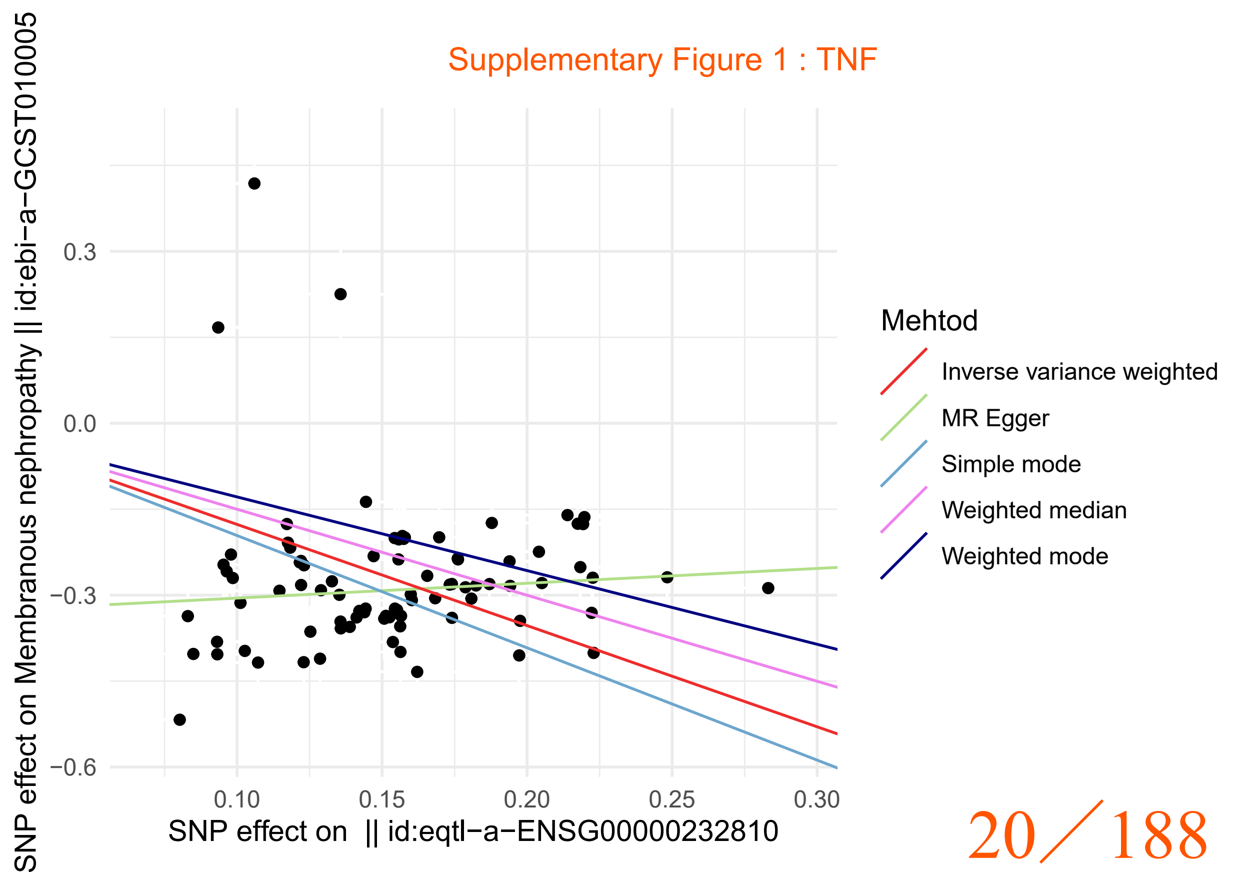
Supplementary Figure 1 : TNF
SNP effect on Membranous nephropathy || id:ebi-a-GCST010005
SNP effect on || id:eqtl-a-ENSG00000232810
0.3
0.0
−0.3
−0.6
0.10
0.15
0.20
0.25
0.30
Mehtod
Inverse variance weighted
MR Egger
Simple mode
Weighted median
Weighted mode

Supplementary Figure 1 : VIM

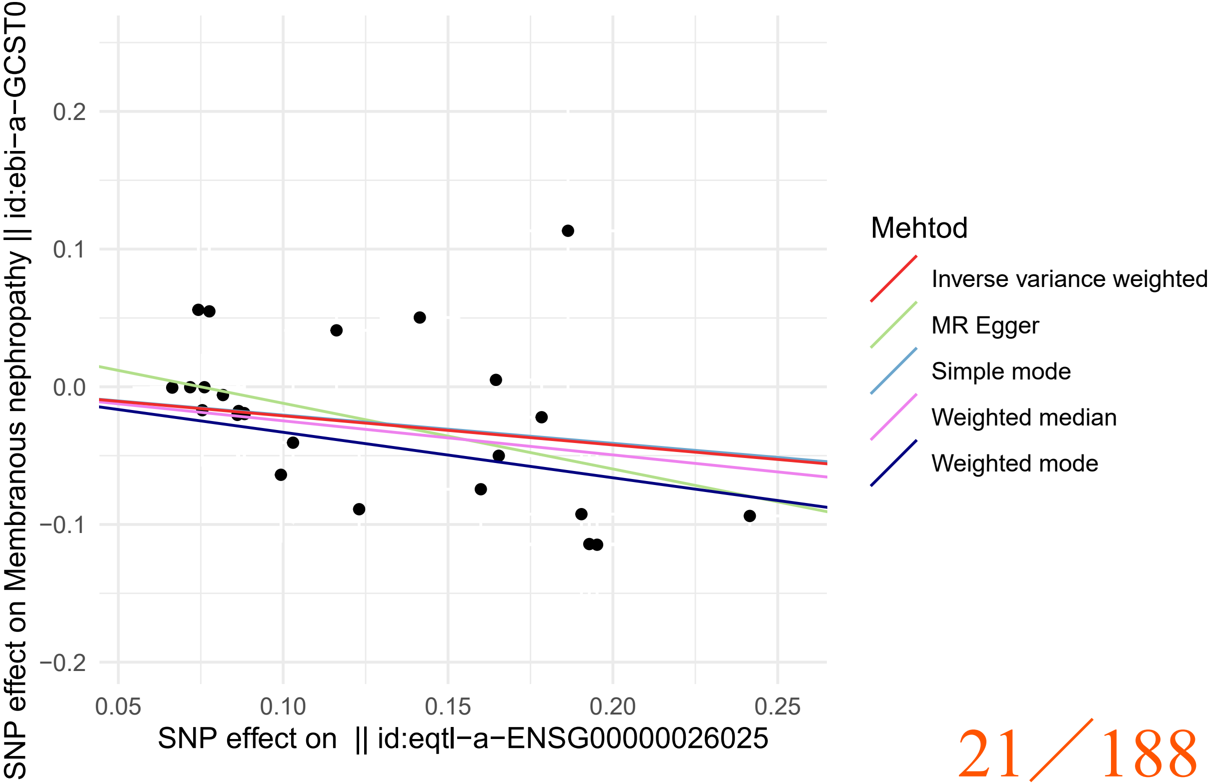

Supplementary Figure 2: ACTA2

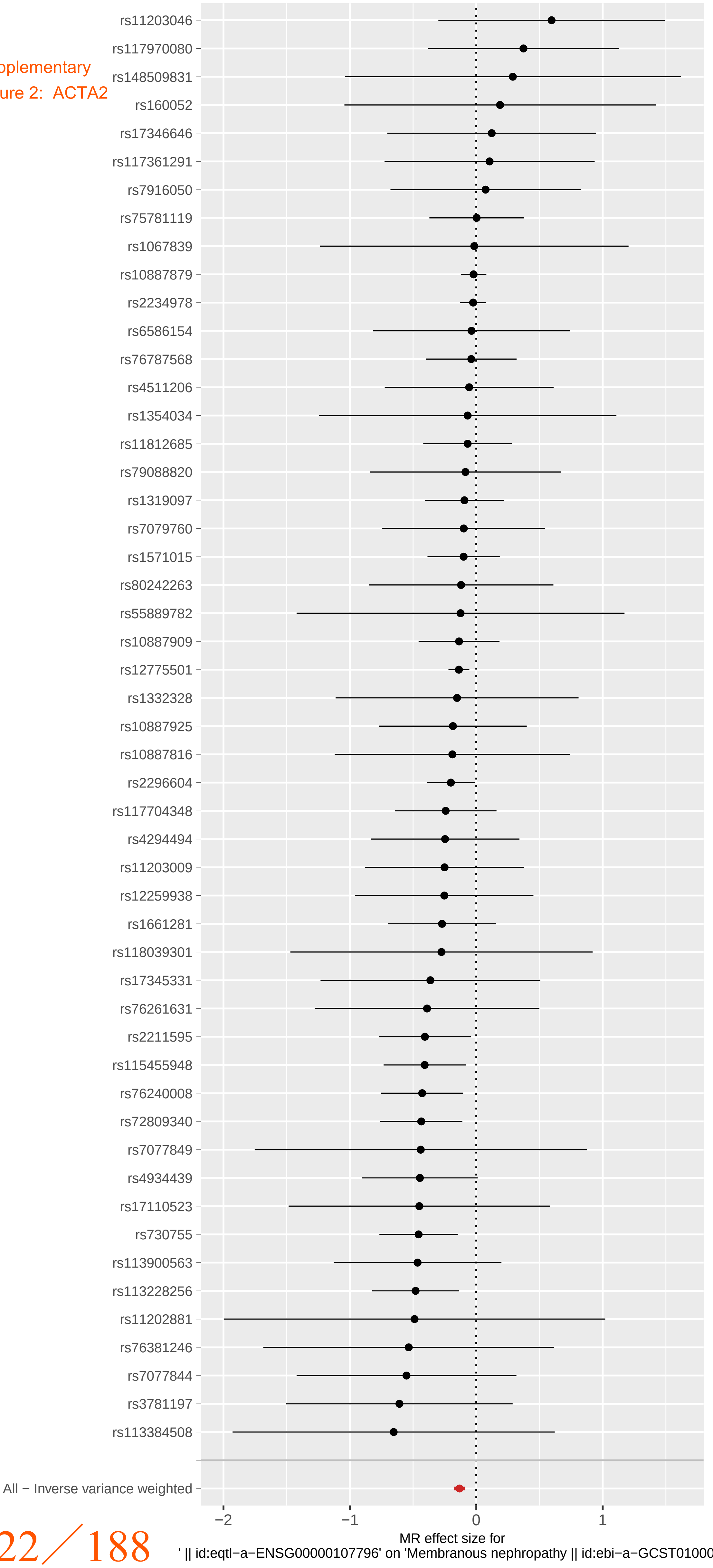

Supplementary Figure 2: AGER

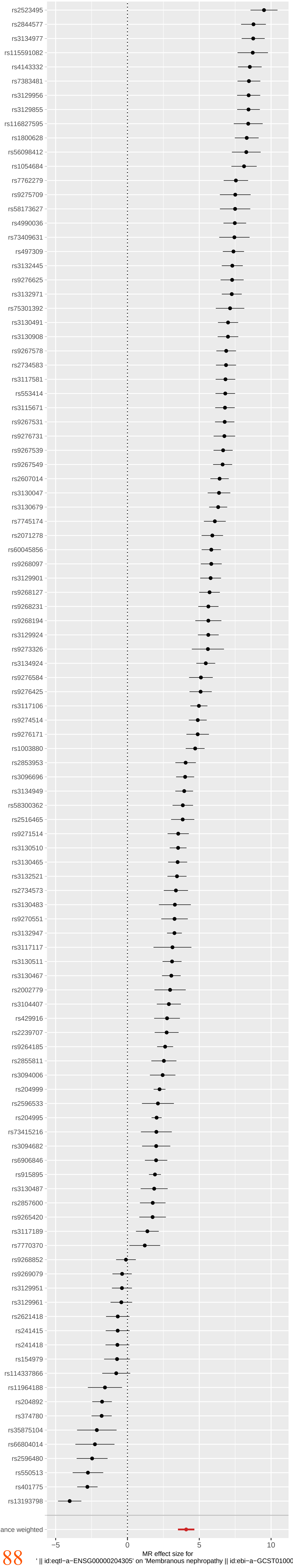

Supplementary Figure 2: ATF6

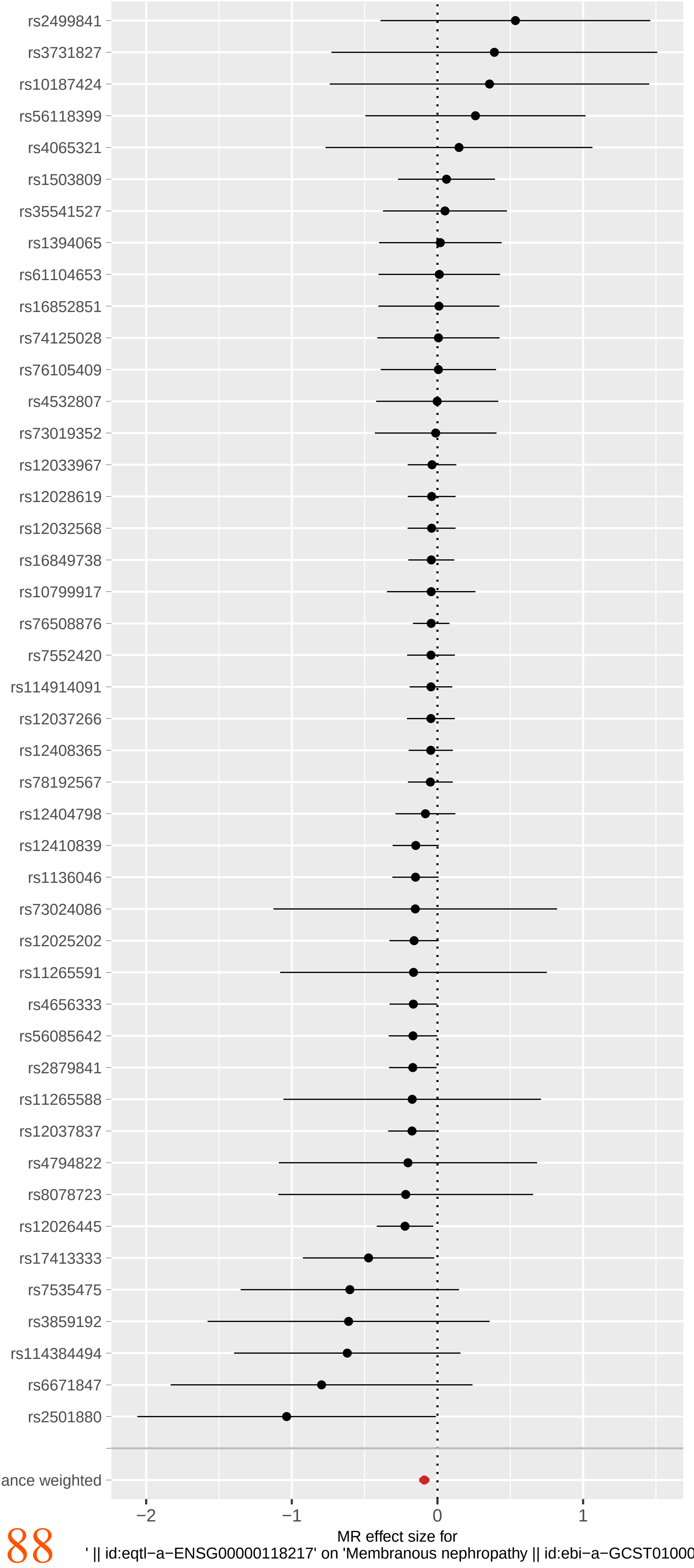

Supplementary Figure 2: BCL2

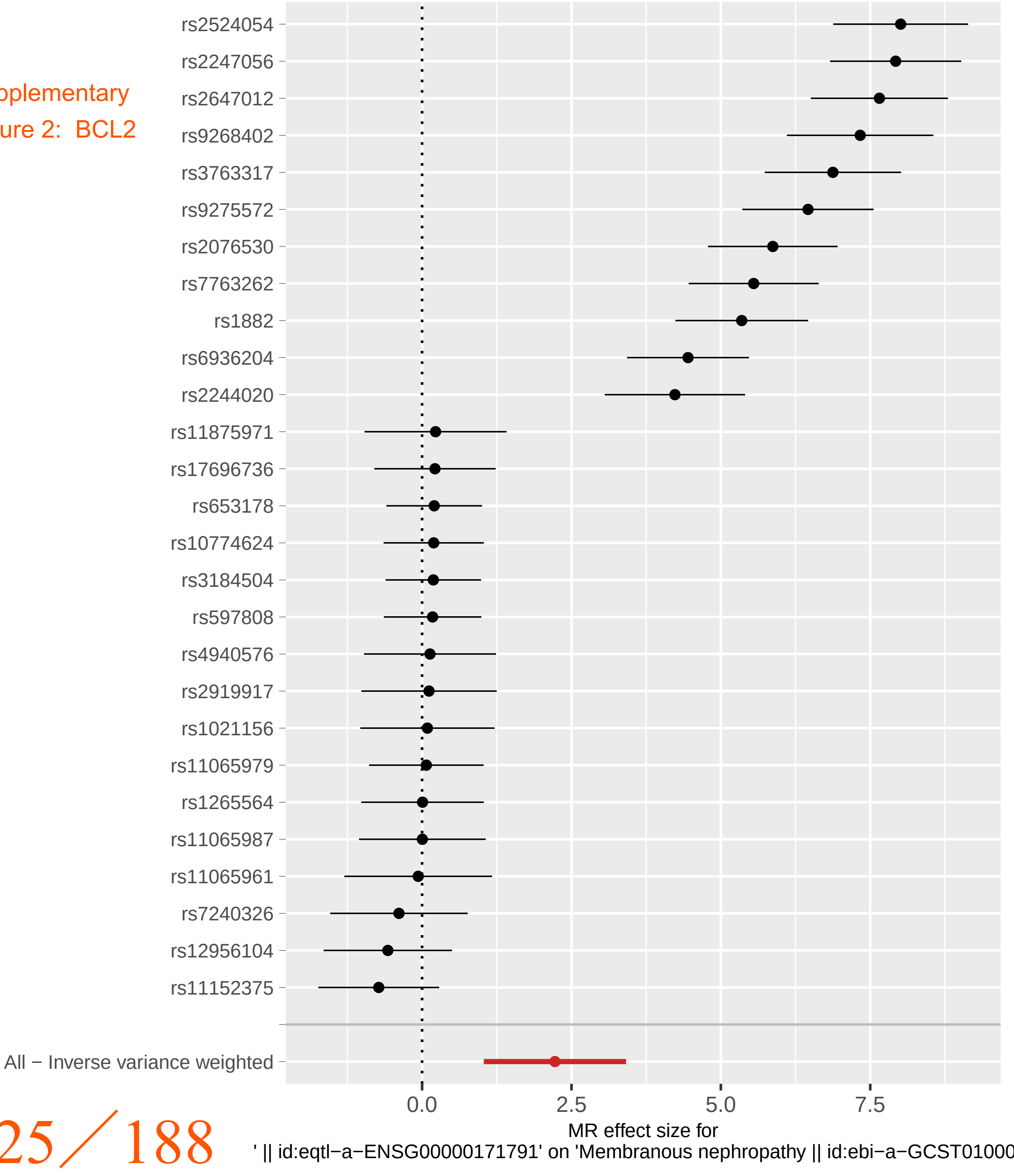

Supplementary Figure 2: CD36

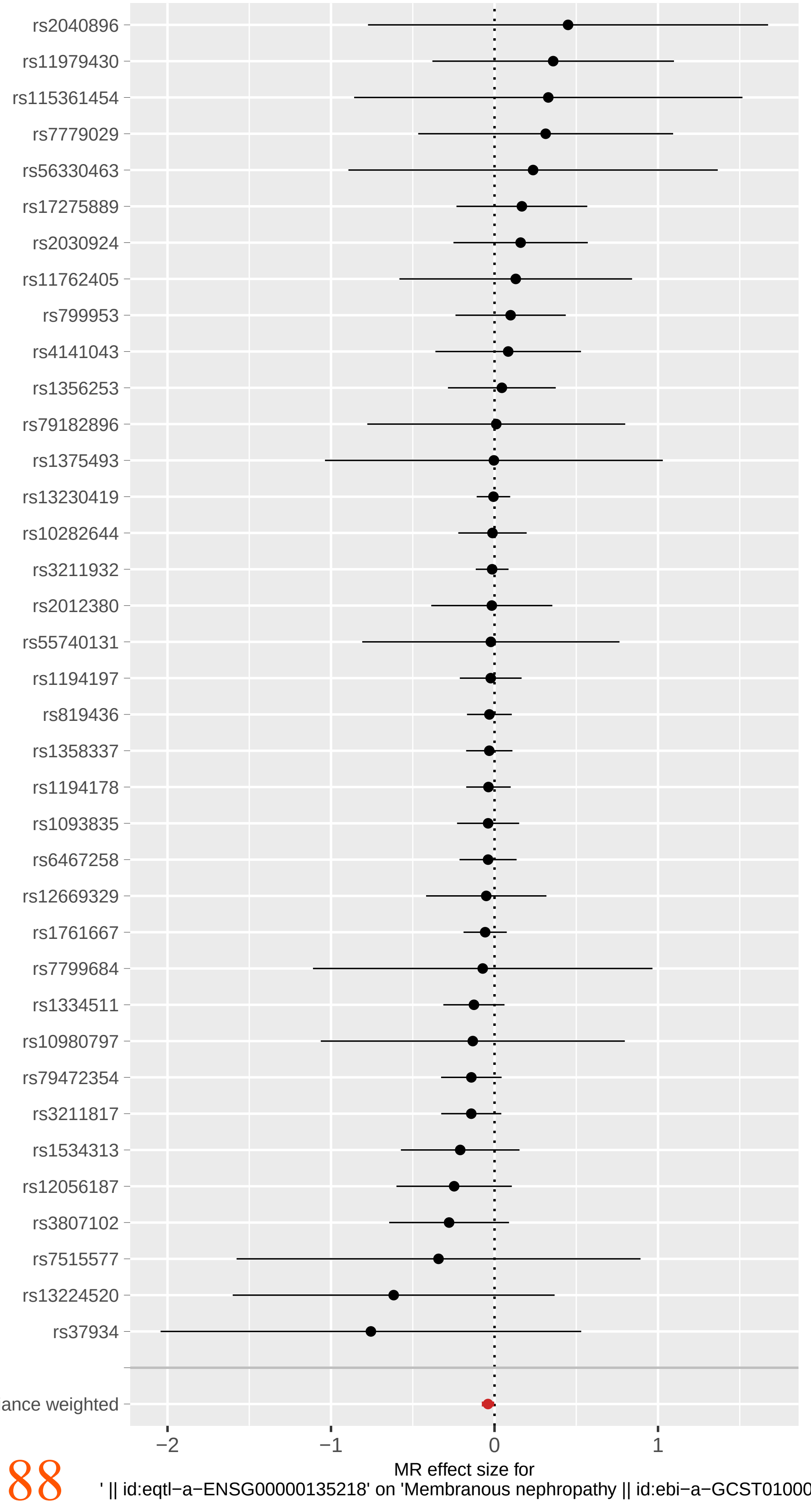

Supplementary Figure 2: CDH1

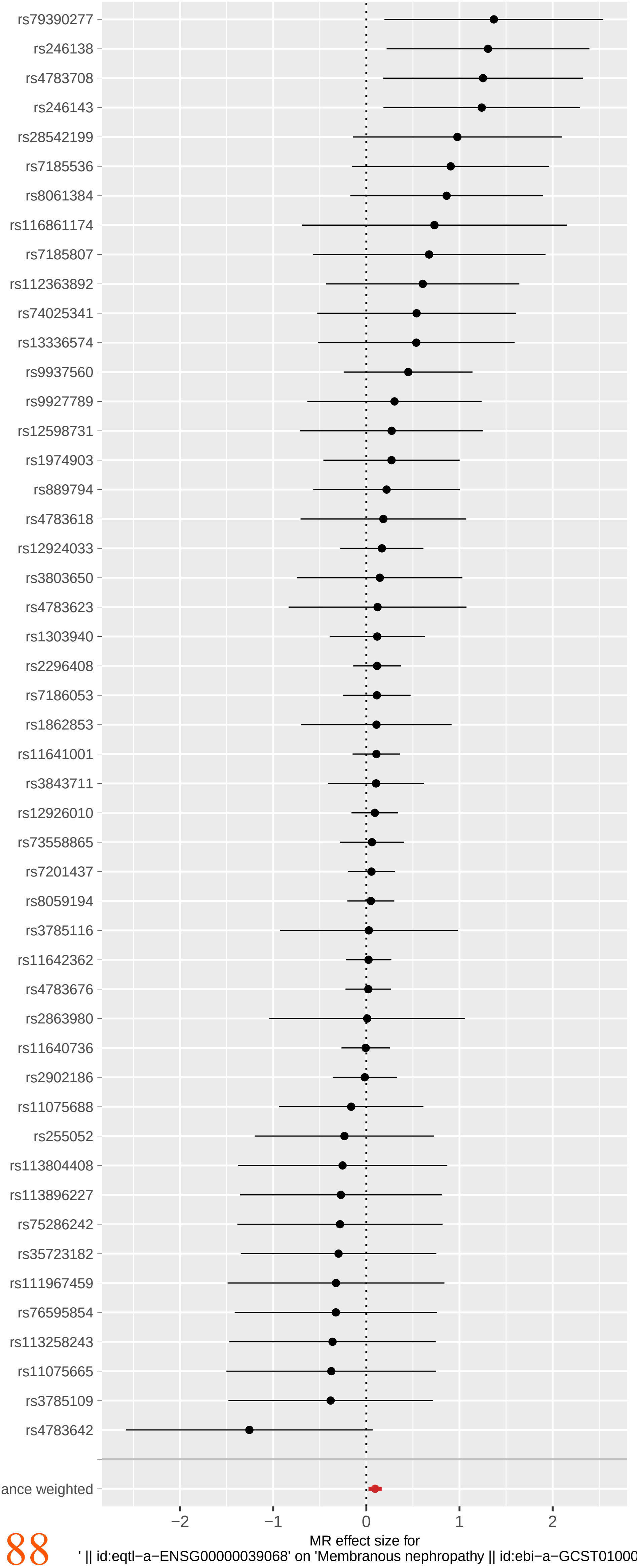

Supplementary Figure 2: DDIT3

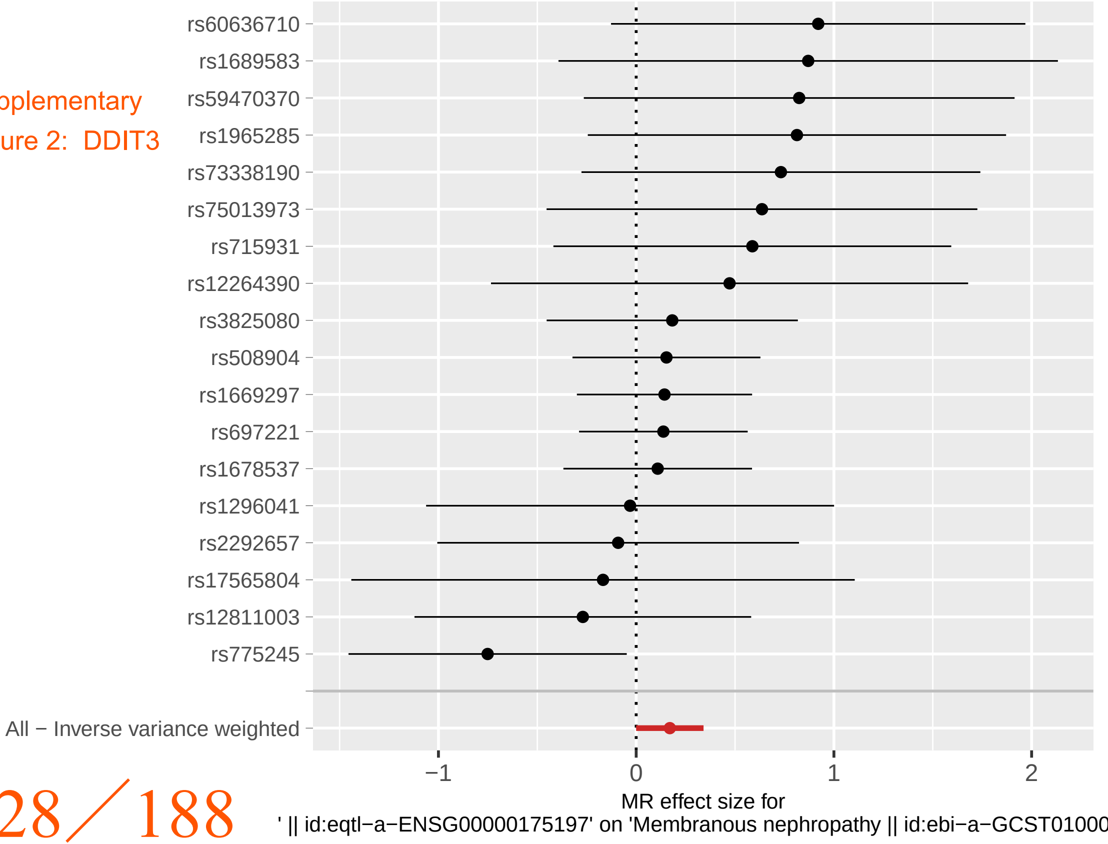

Supplementary Figure 2: FASN

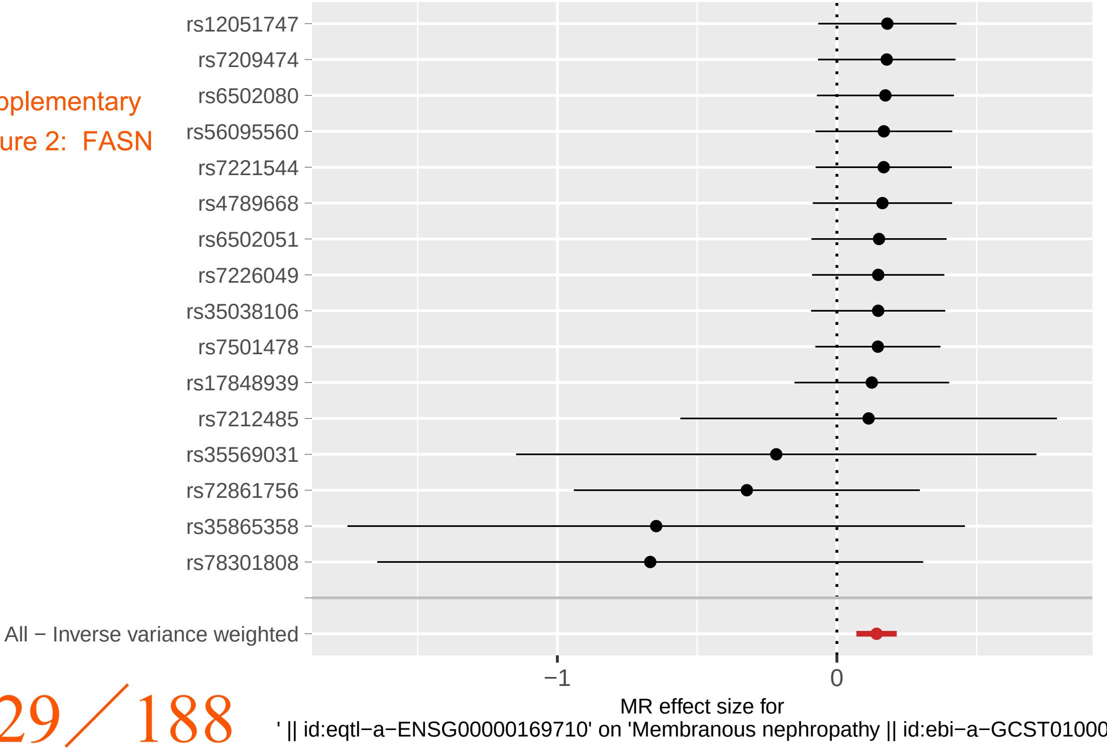

Supplementary Figure 2: IL1B

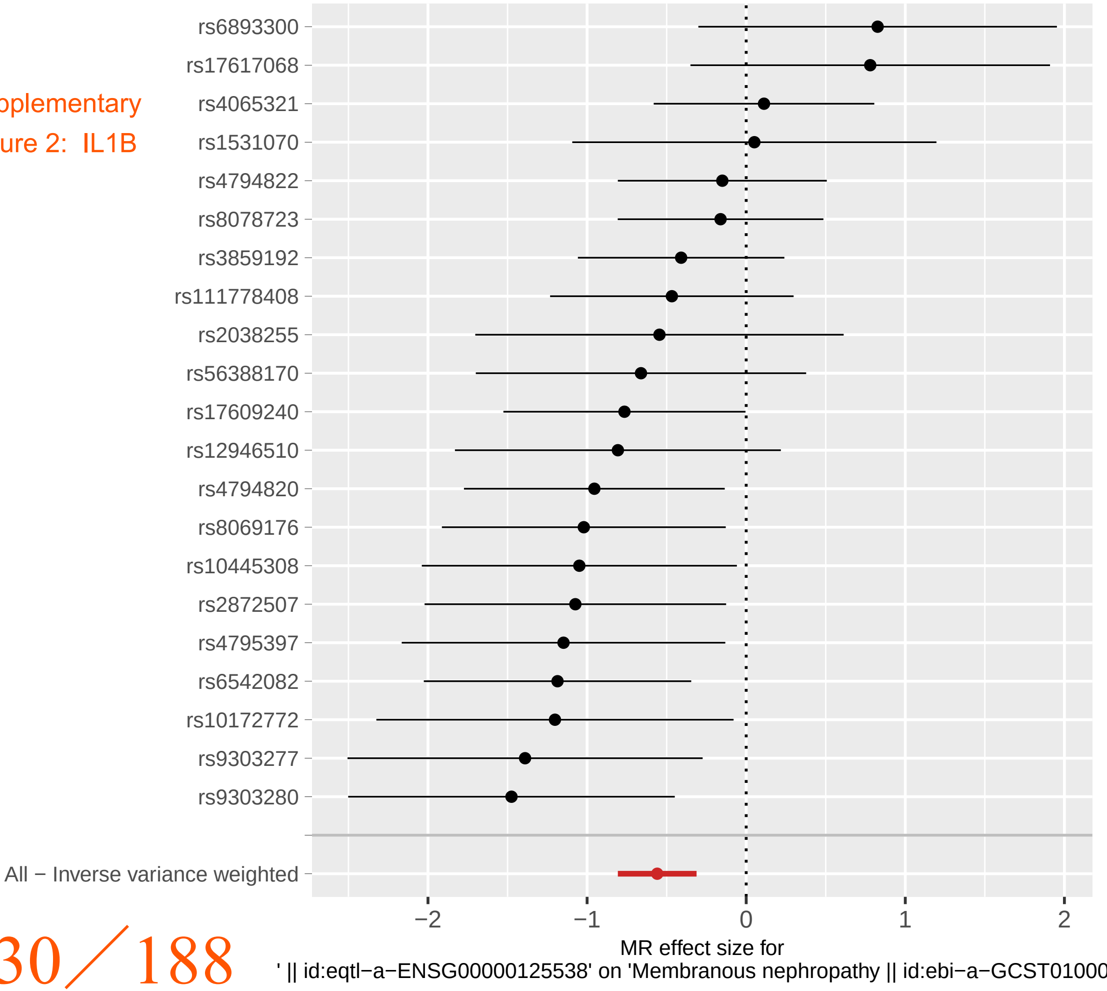

Supplementary Figure 2: INSR

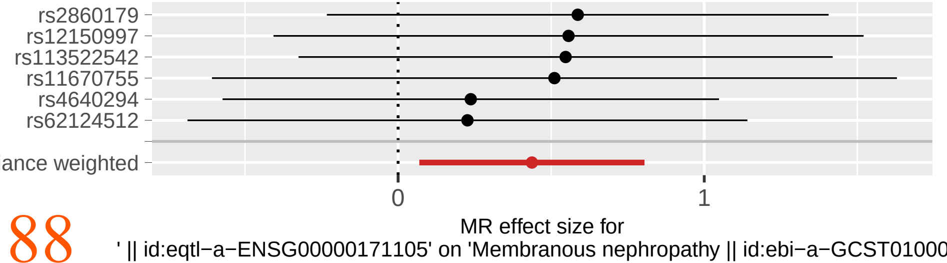

Supplementary Figure 2: JAK2

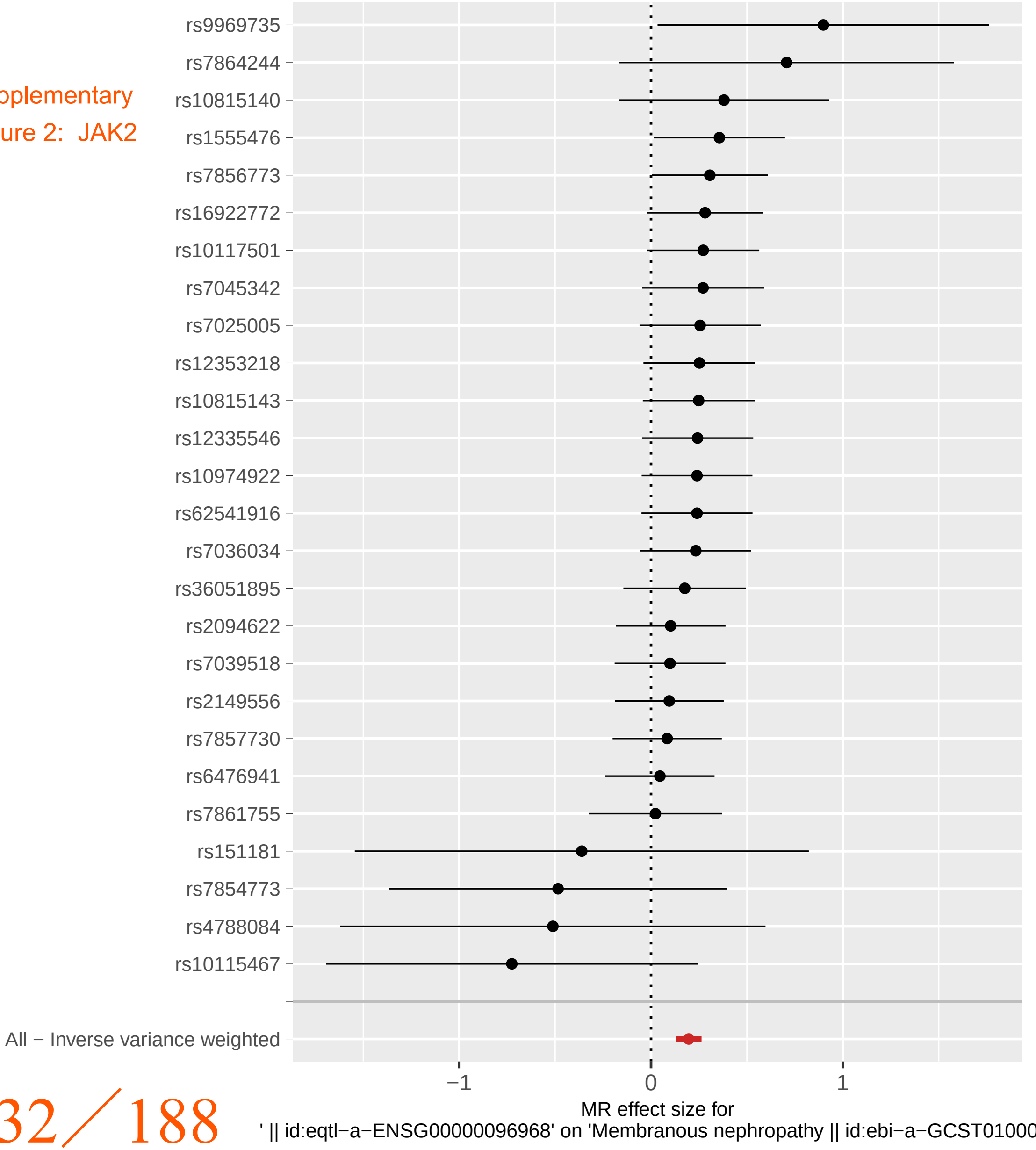

Supplementary Figure 2: MAP1LC3B

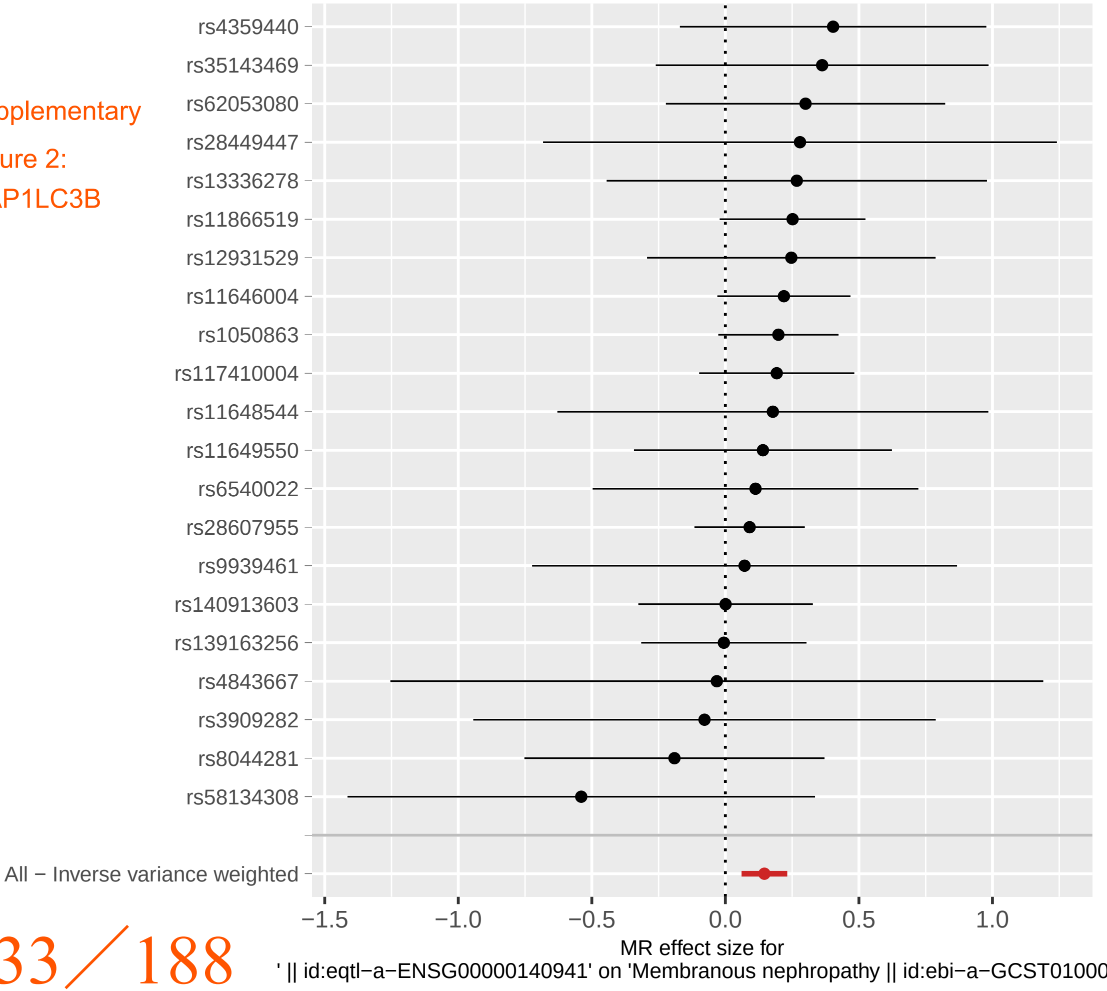

Supplementary Figure 2: MPO

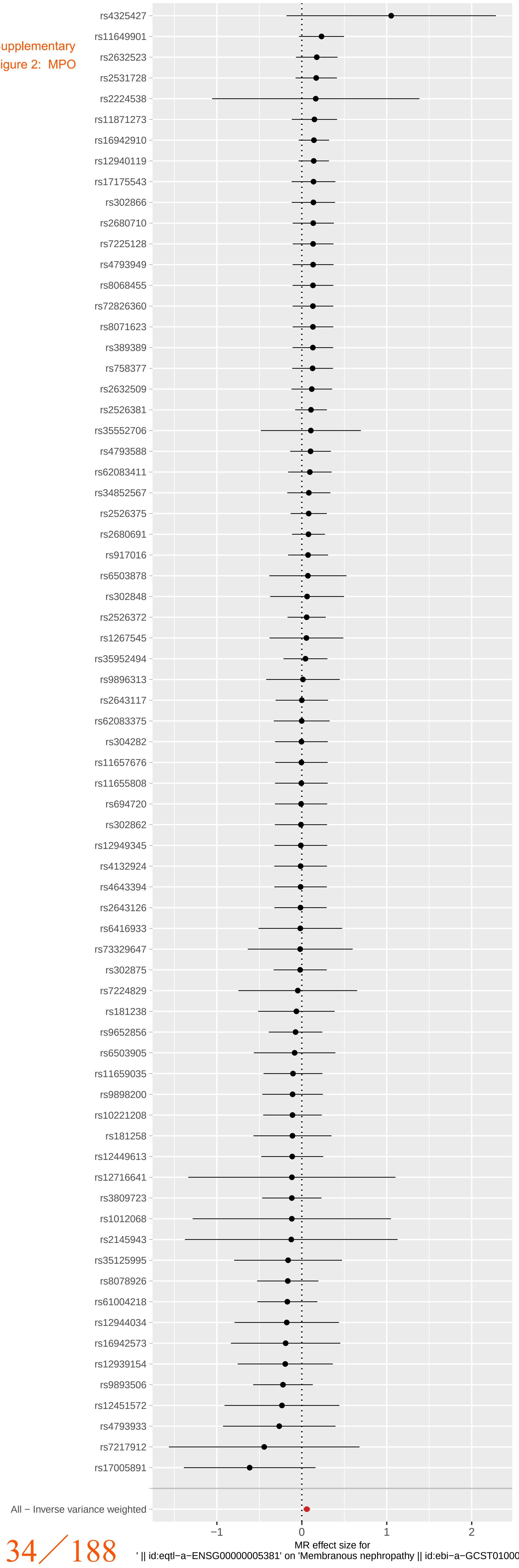

Supplementary Figure 2: MTOR

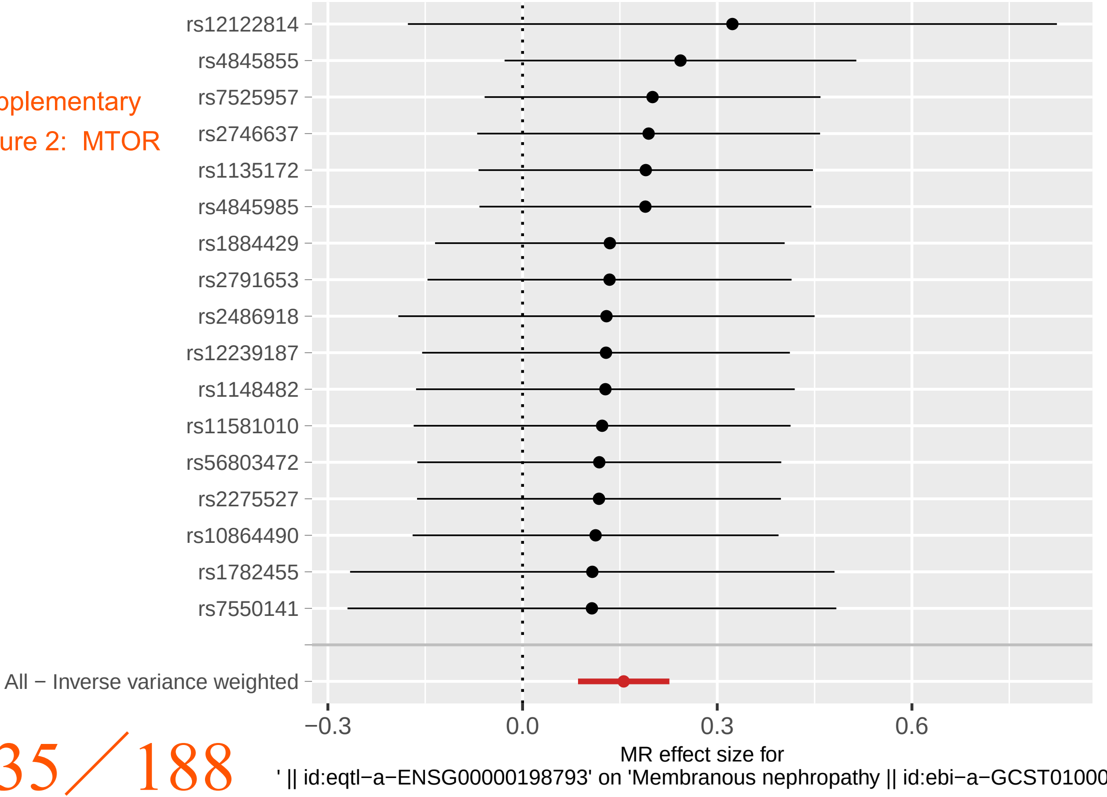

35／188

Supplementary Figure 2: OGA

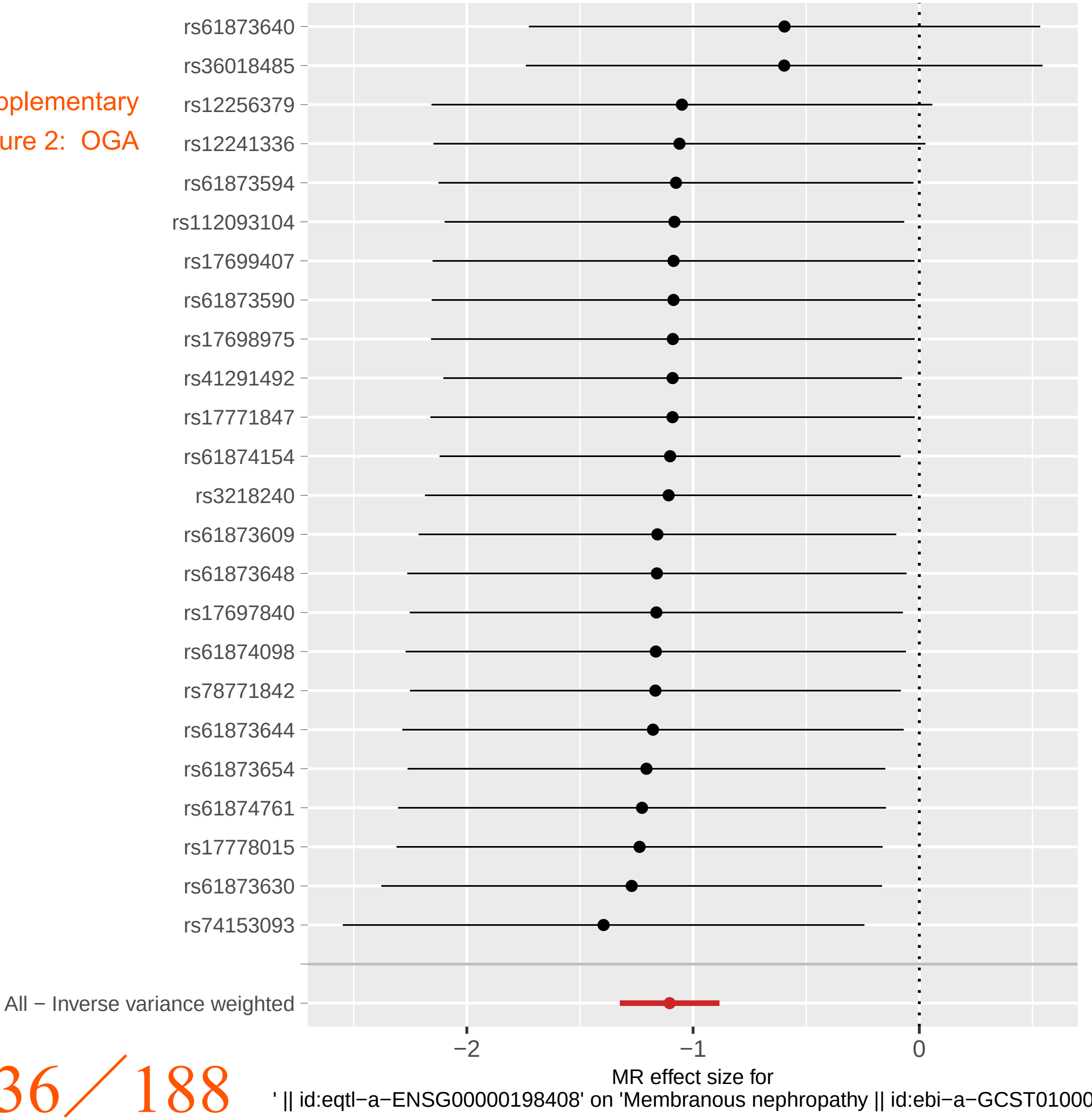

36／188

Supplementary Figure 2: PPARA

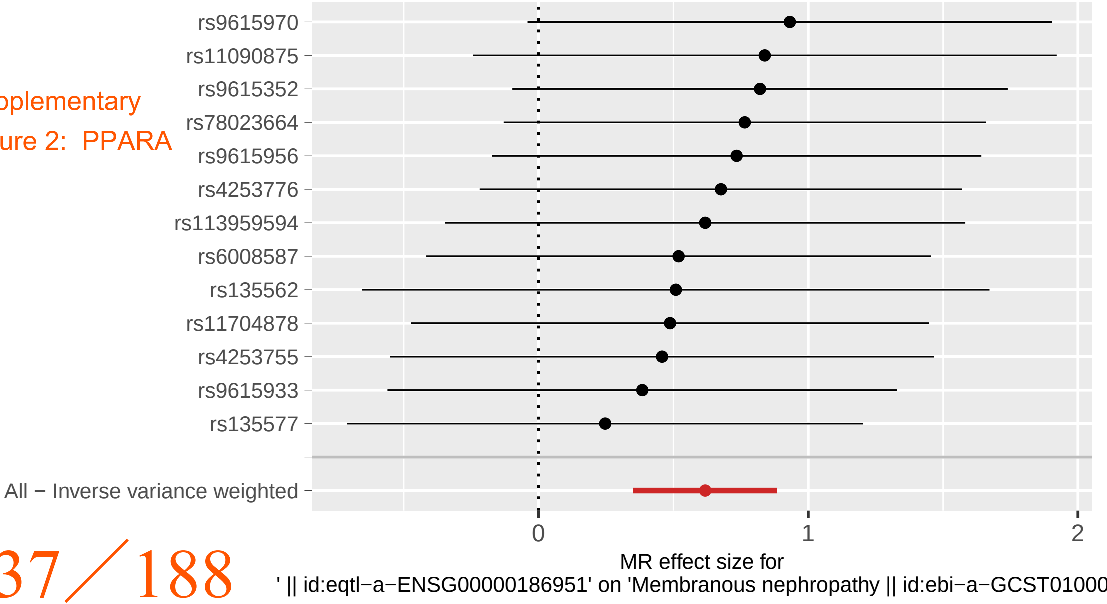

Supplementary Figure 2: PTGS2

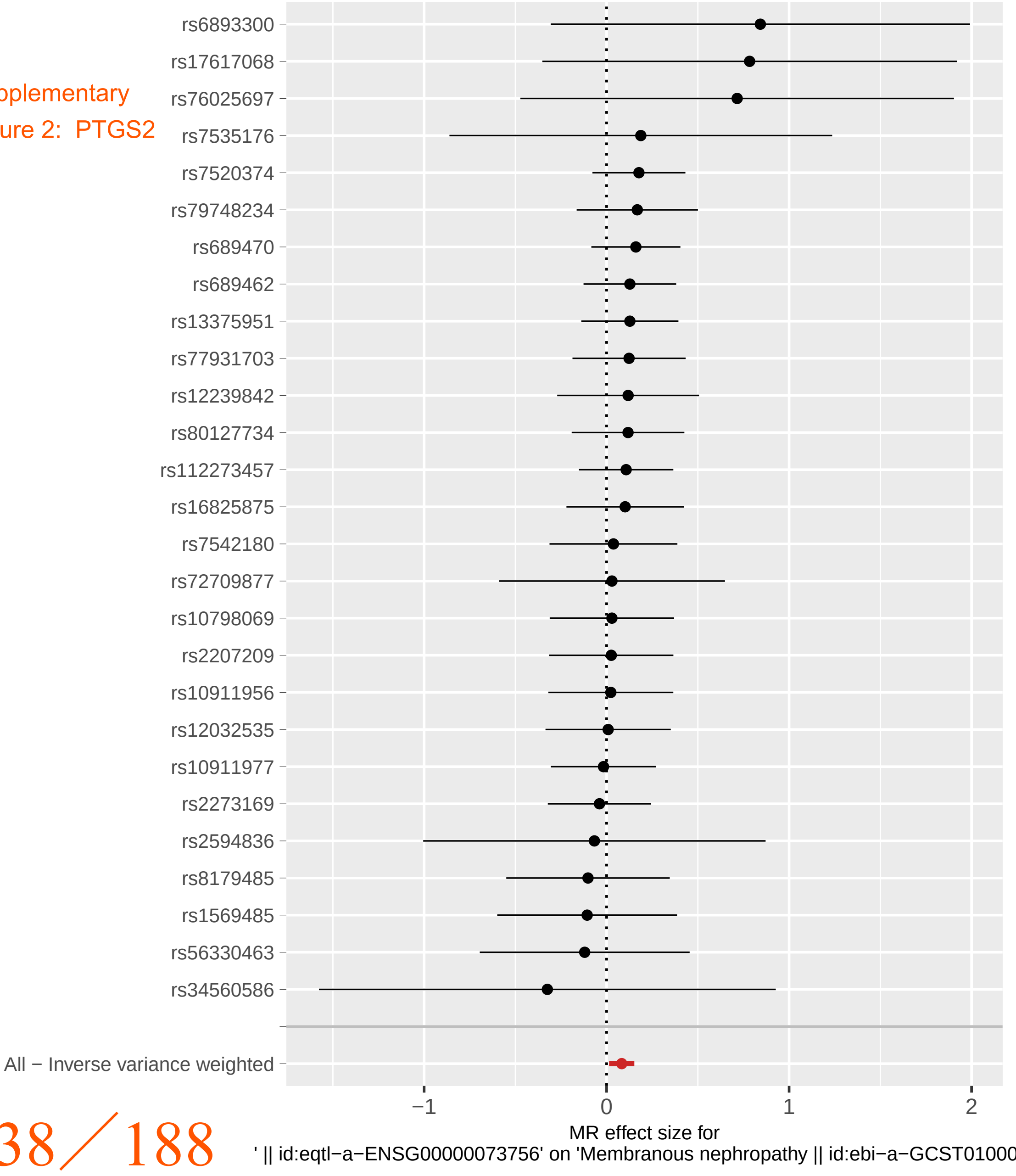

38／188

Supplementary Figure 2: SIRT1

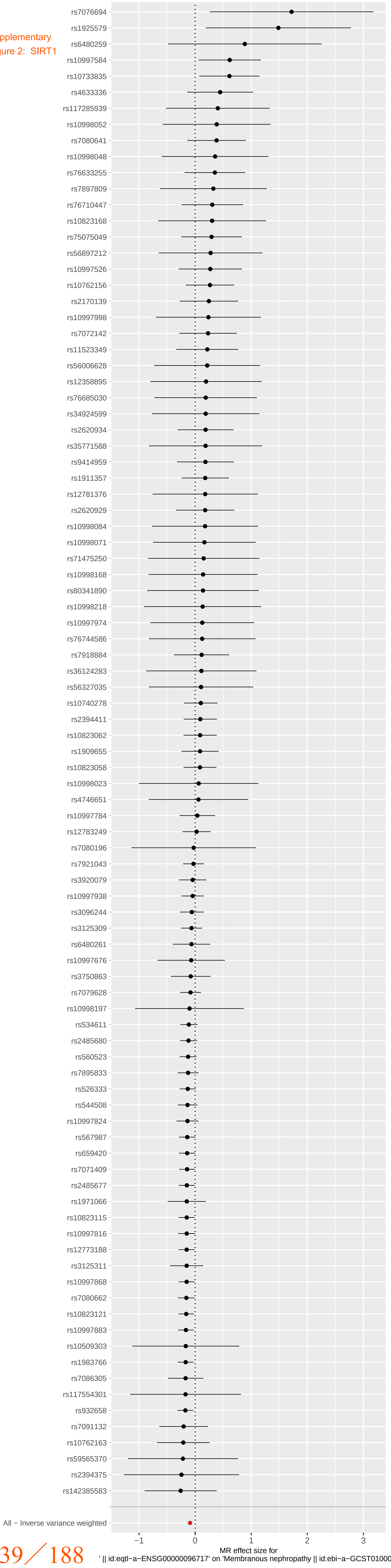

Supplementary Figure 2: SREBF1

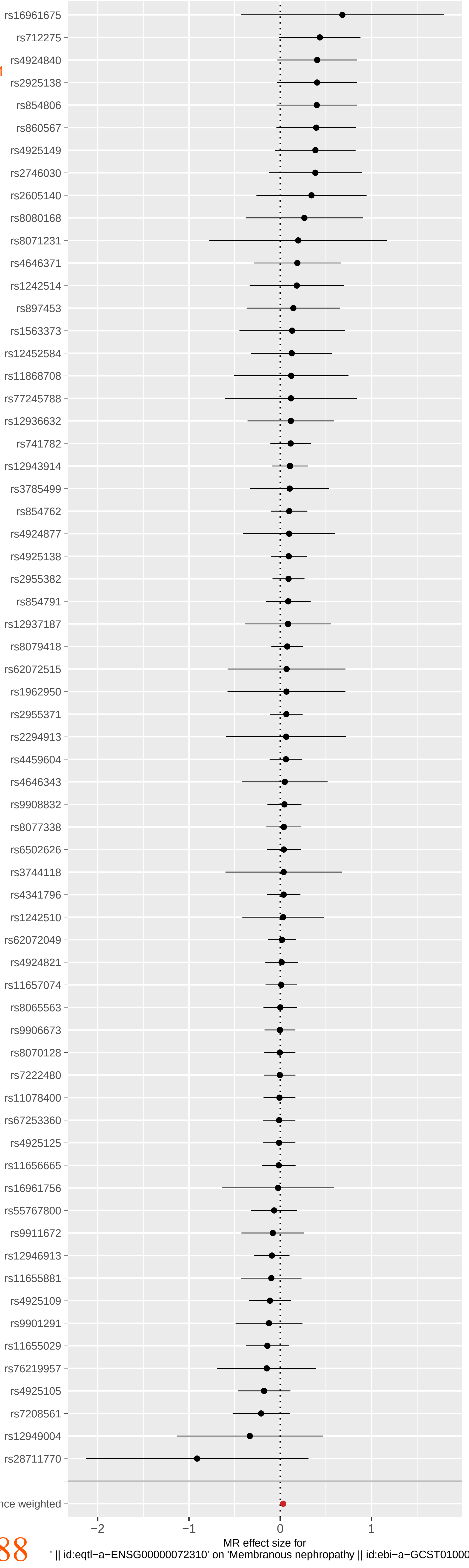

Supplementary Figure 2: TNF

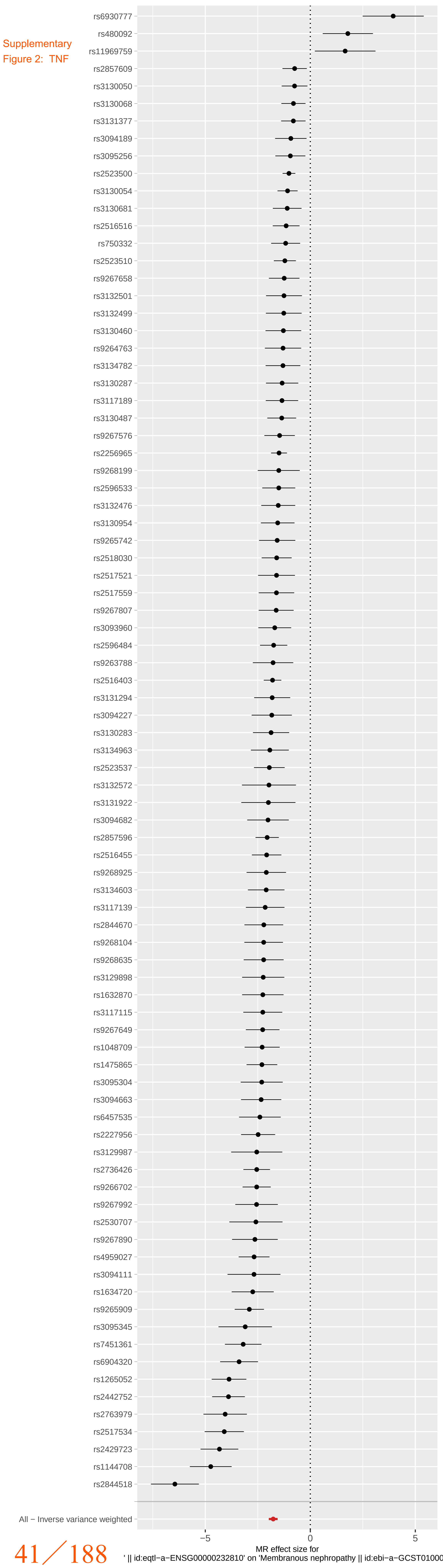

Supplementary Figure 2: VIM

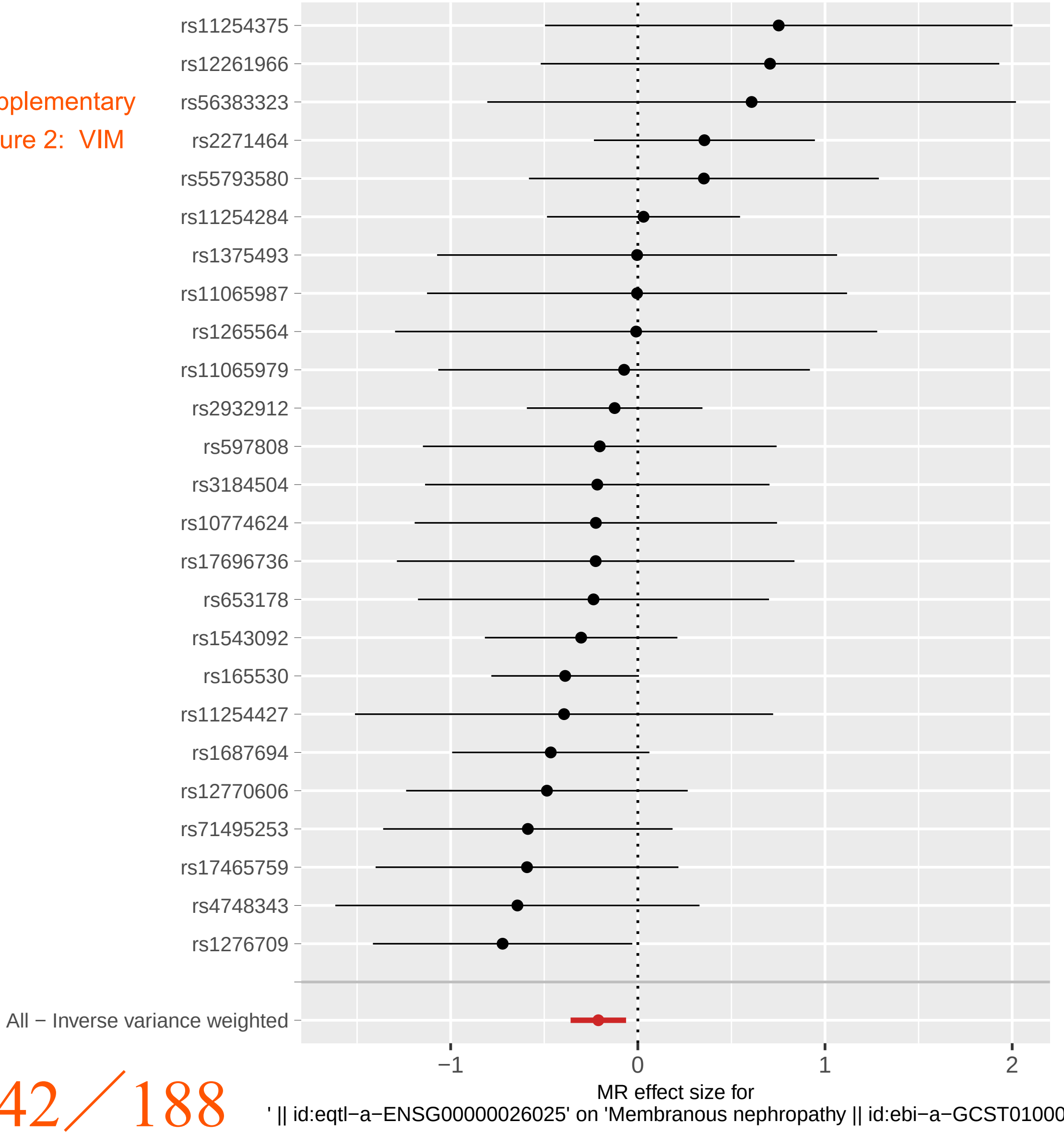

Supplementary Figure 3:
ACTA2

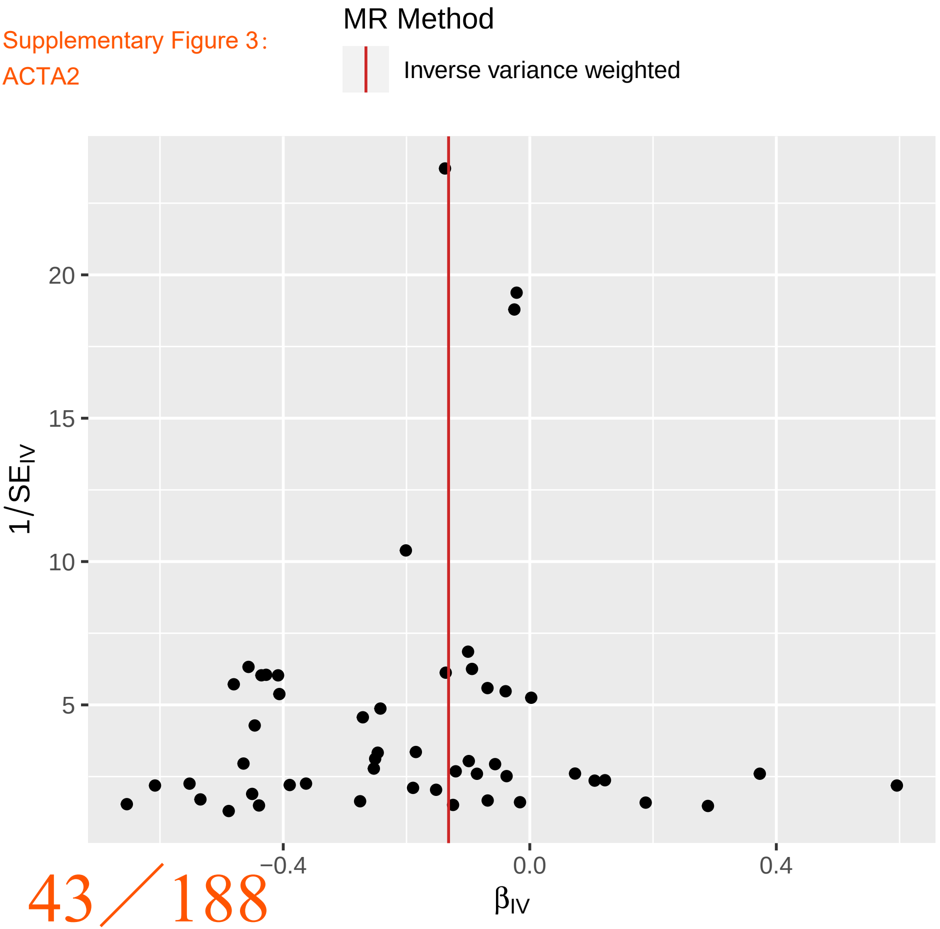

Supplementary Figure 3:
AGER

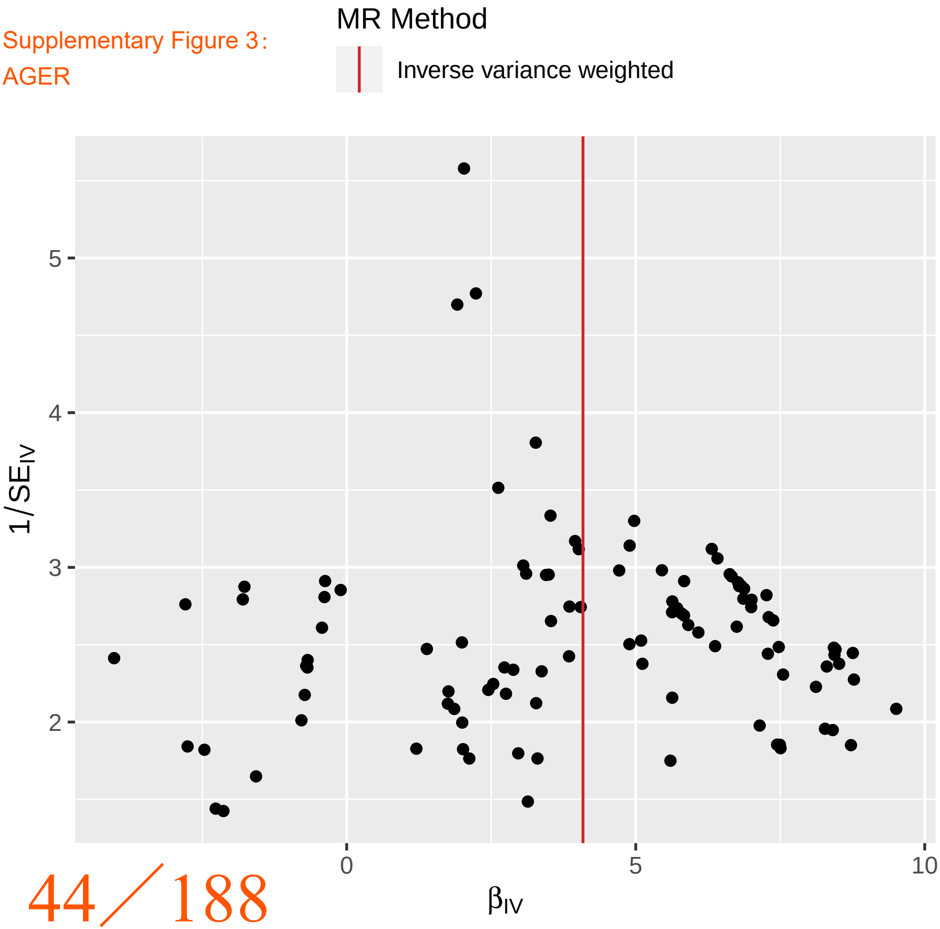

Supplementary Figure 3:
ATF6

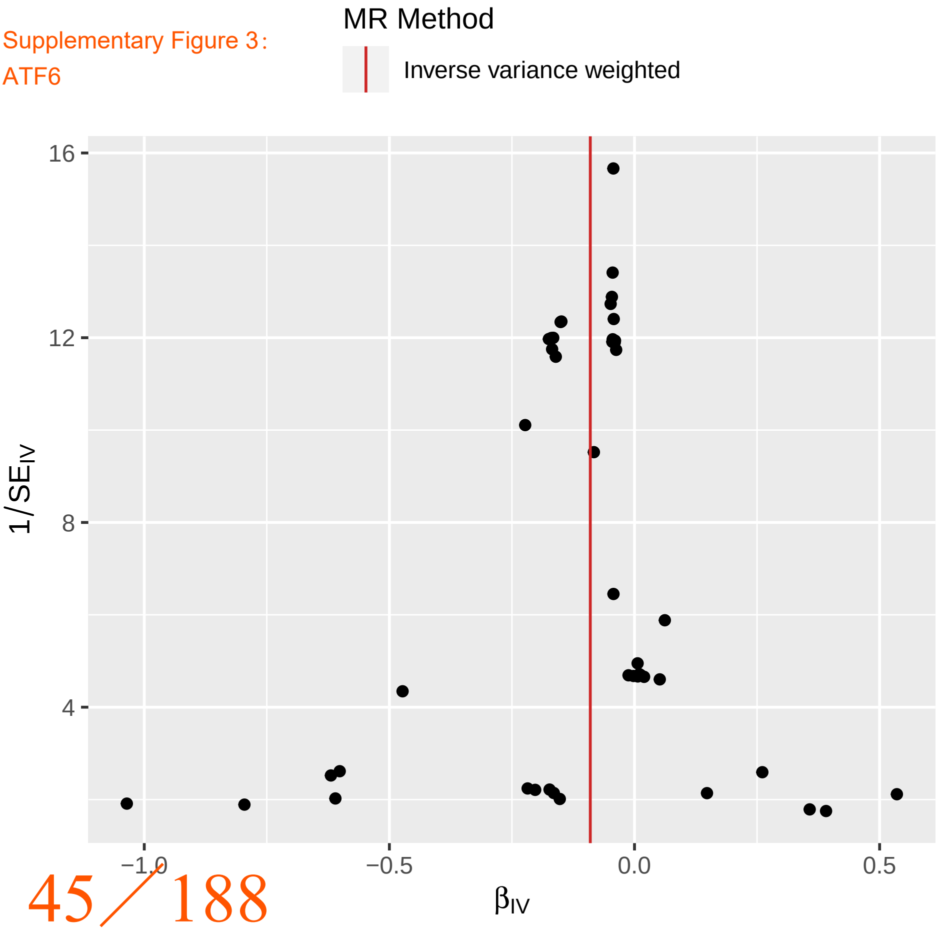

Supplementary Figure 3:
BCL2

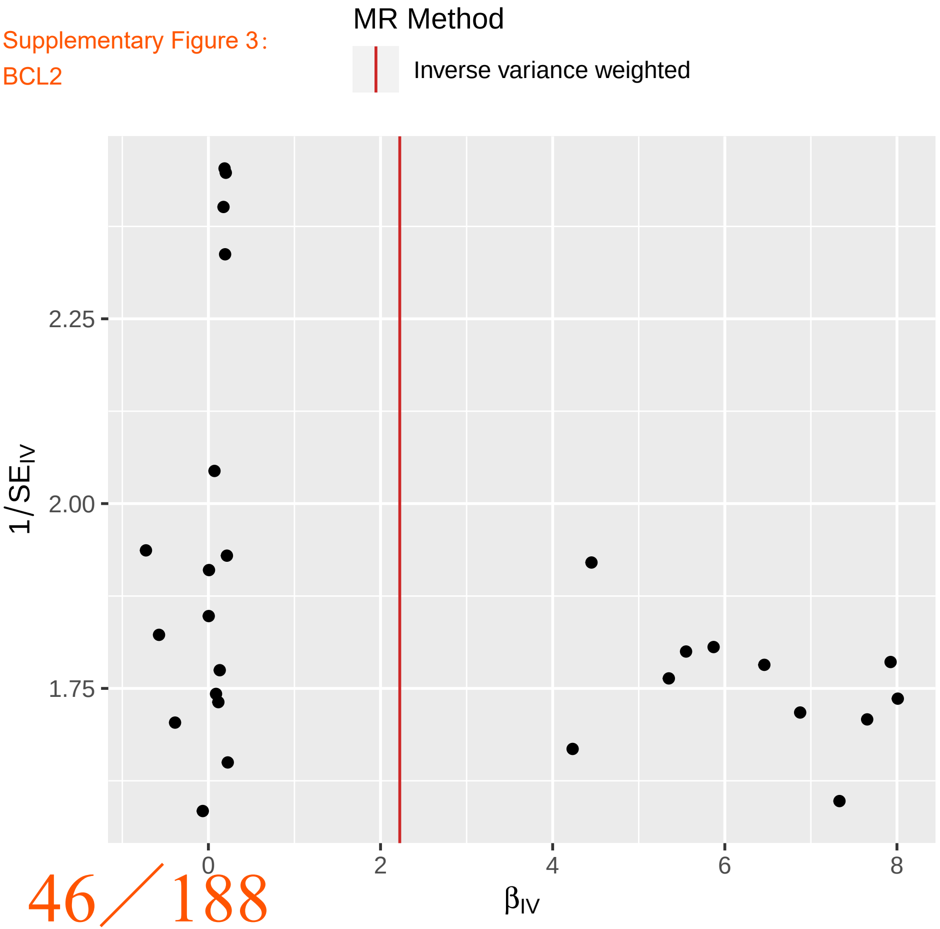

Supplementary Figure 3：
CD36

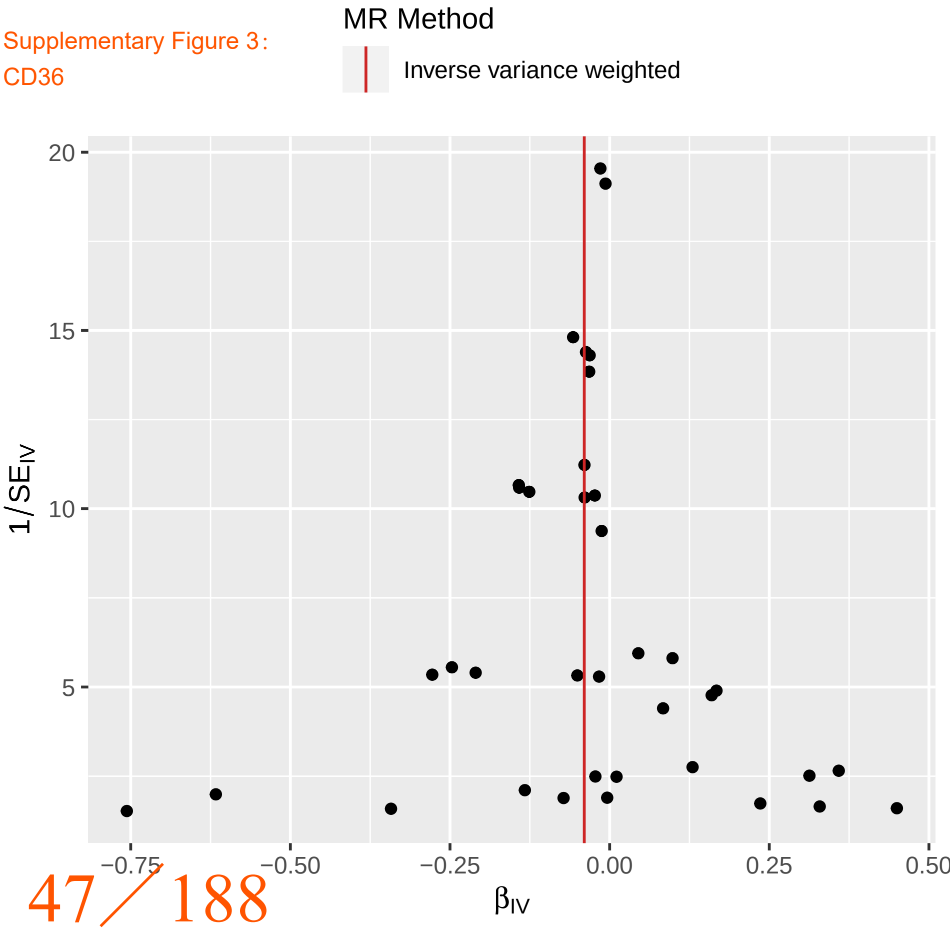

Supplementary Figure 3:
CDH1

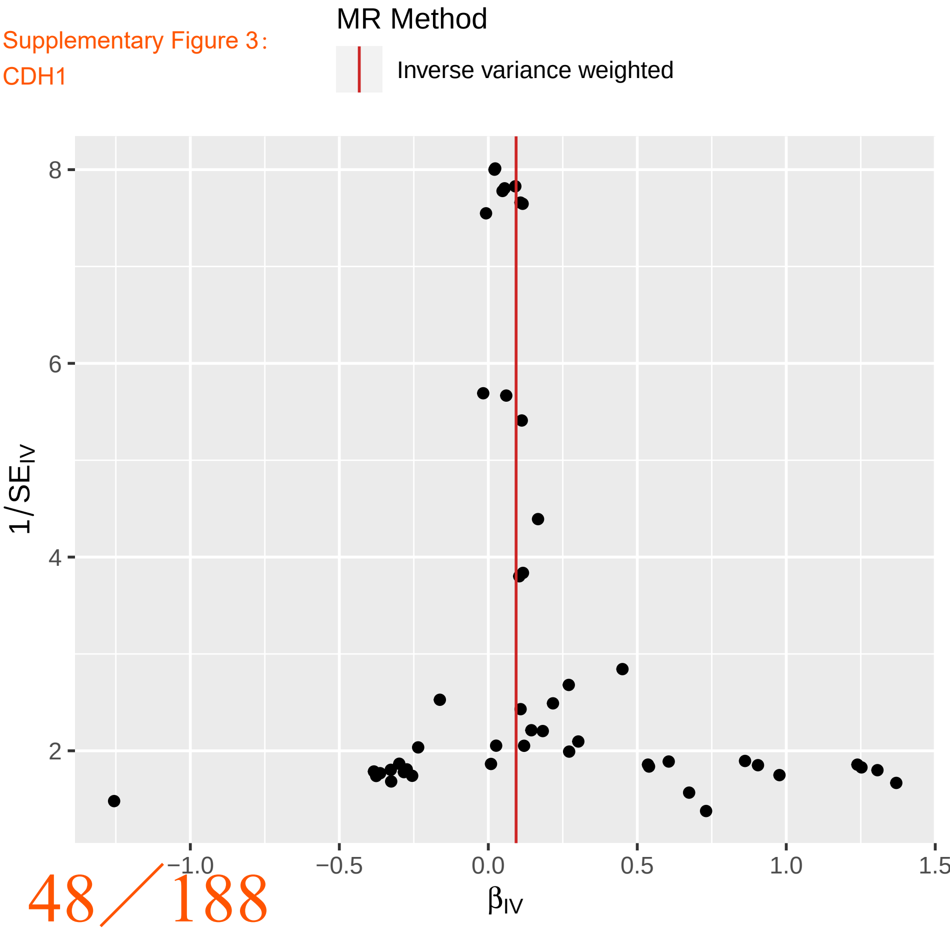

Supplementary Figure 3:
DDIT3

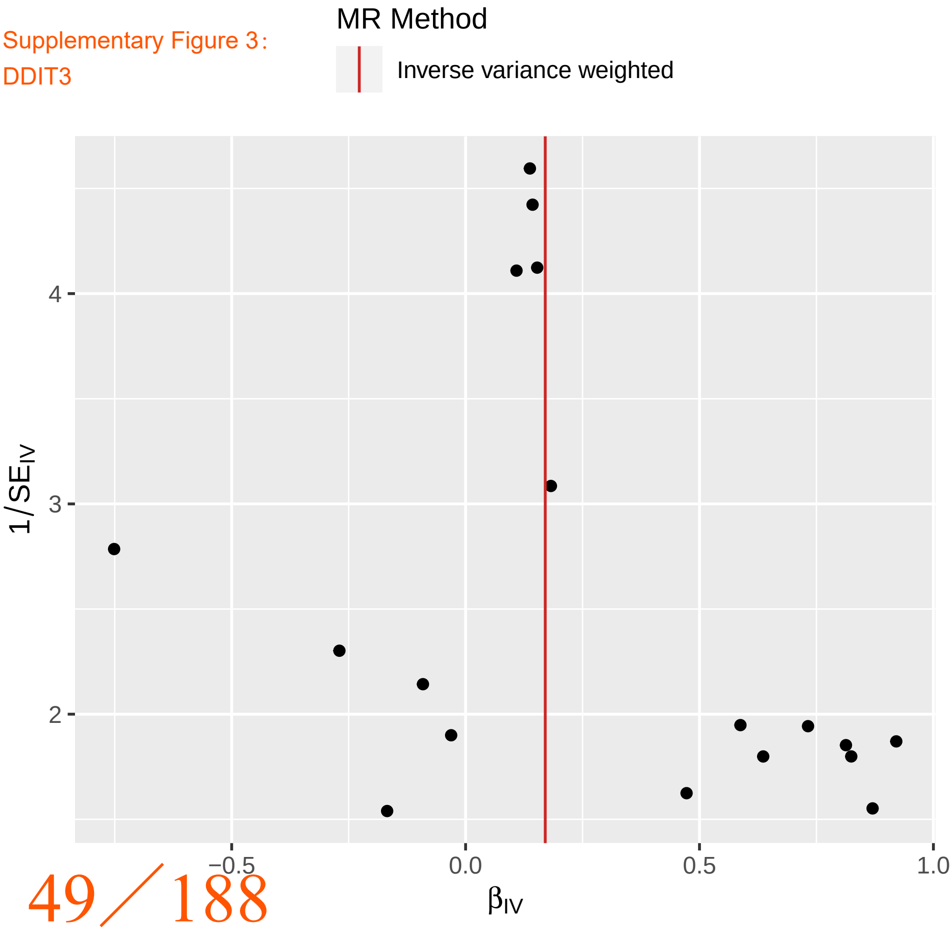

Supplementary Figure 3：FASN

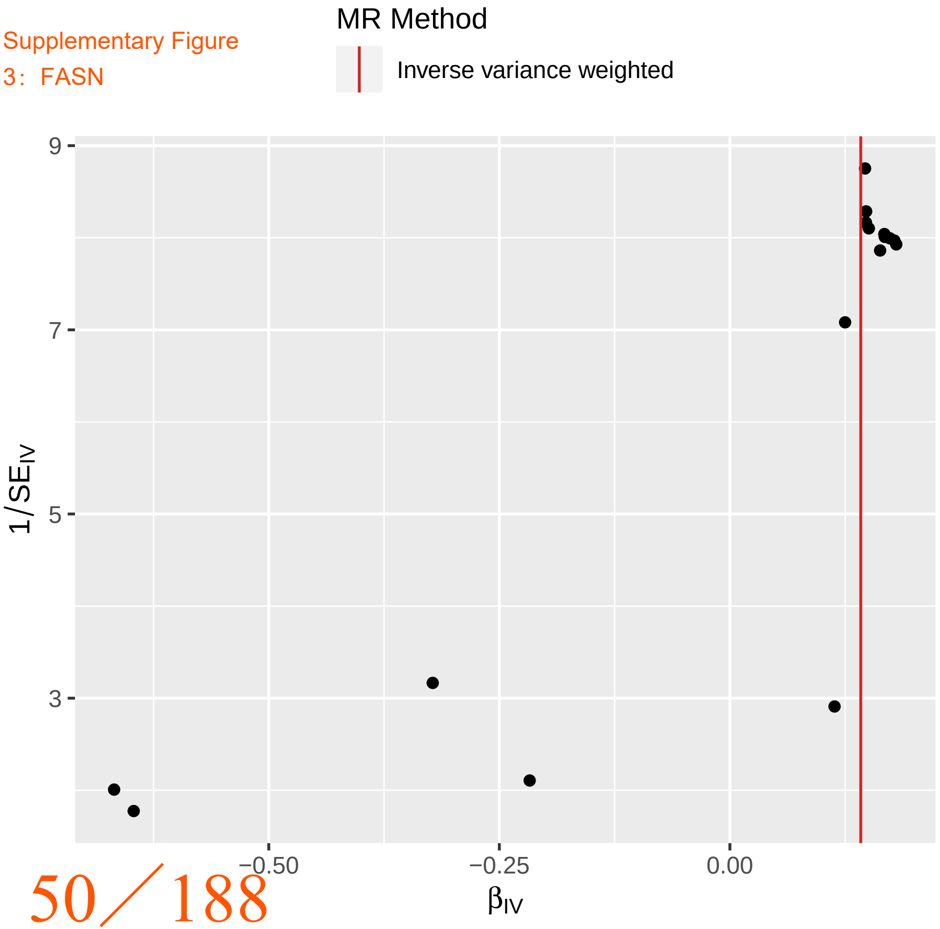

Supplementary Figure 3:
IL1B

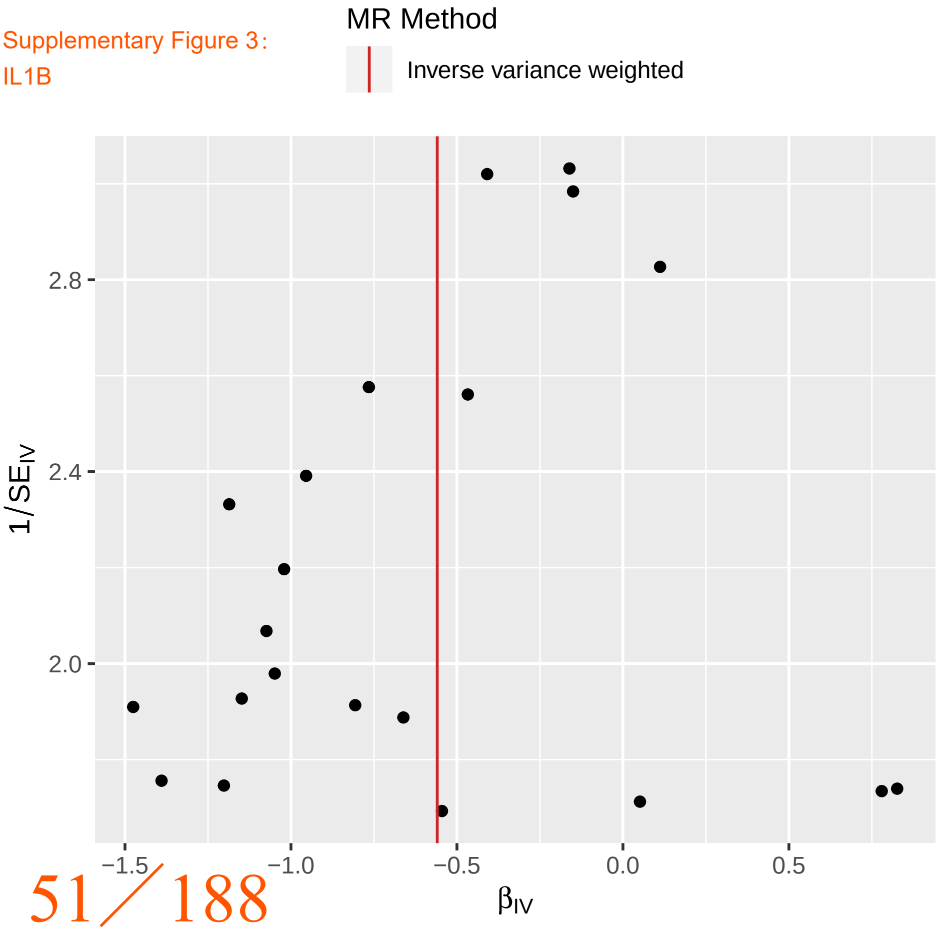

Supplementary Figure 3:
INSR

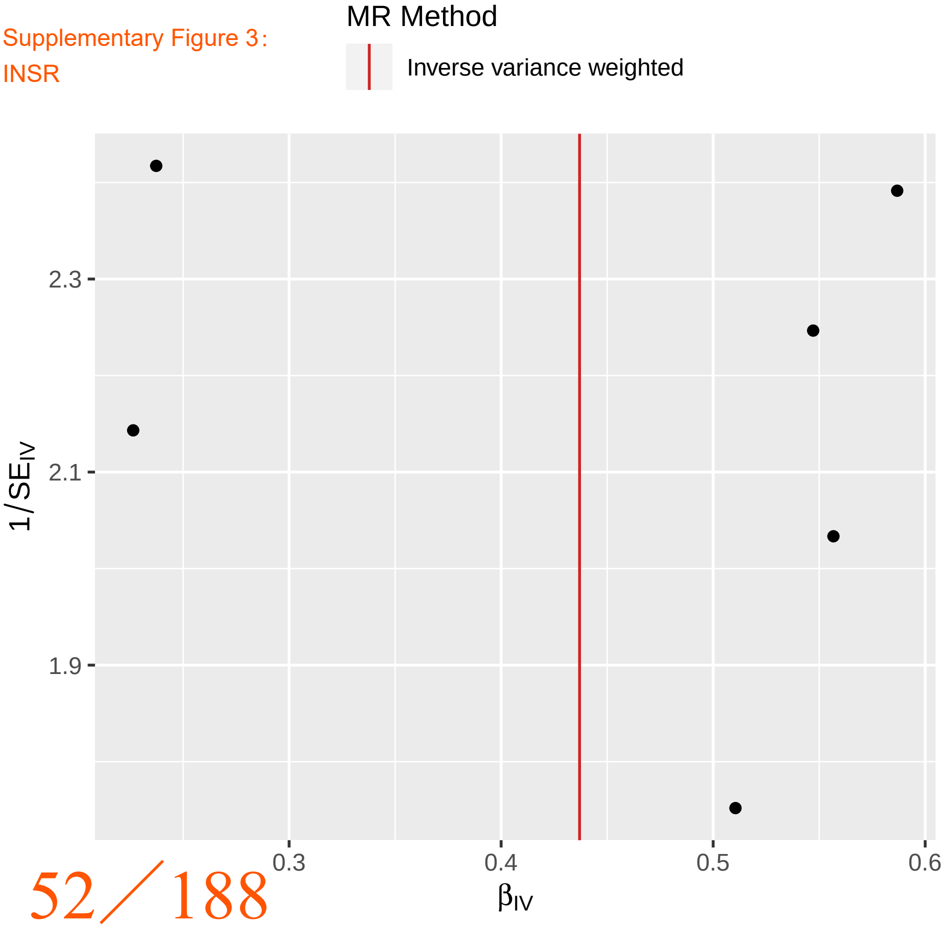

Supplementary Figure 3:
JAK2

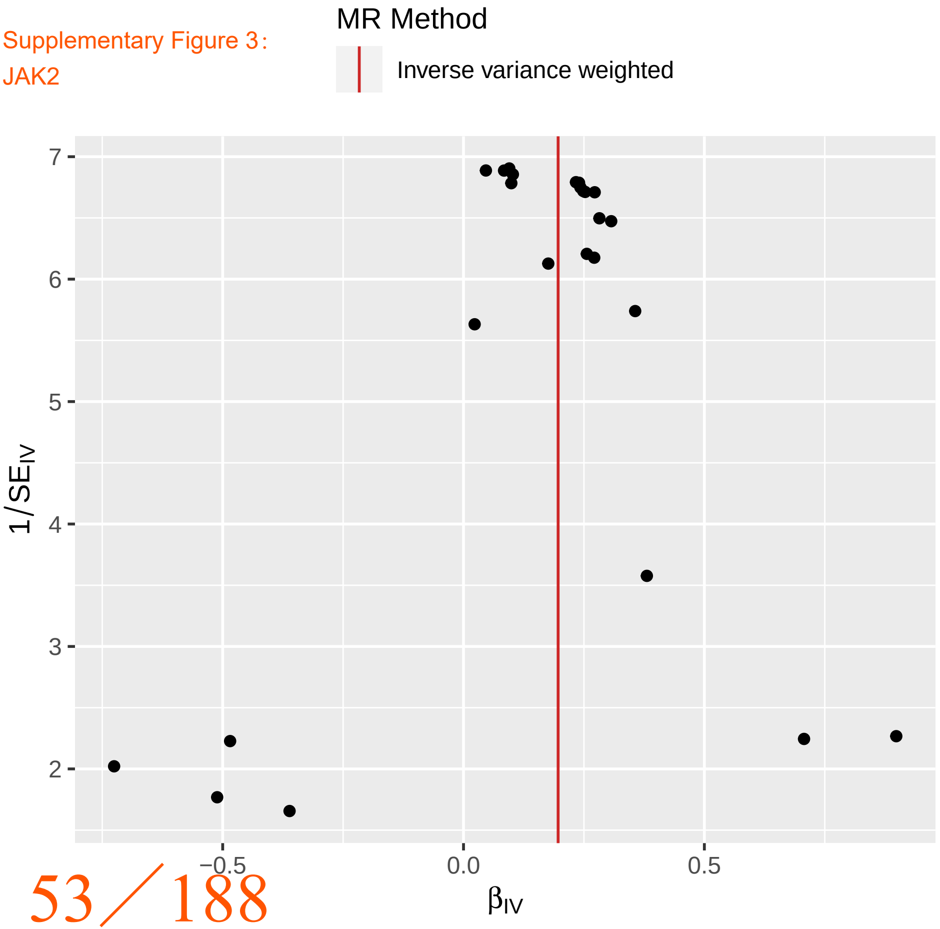

Supplementary Figure 3:
MAP1LC3B

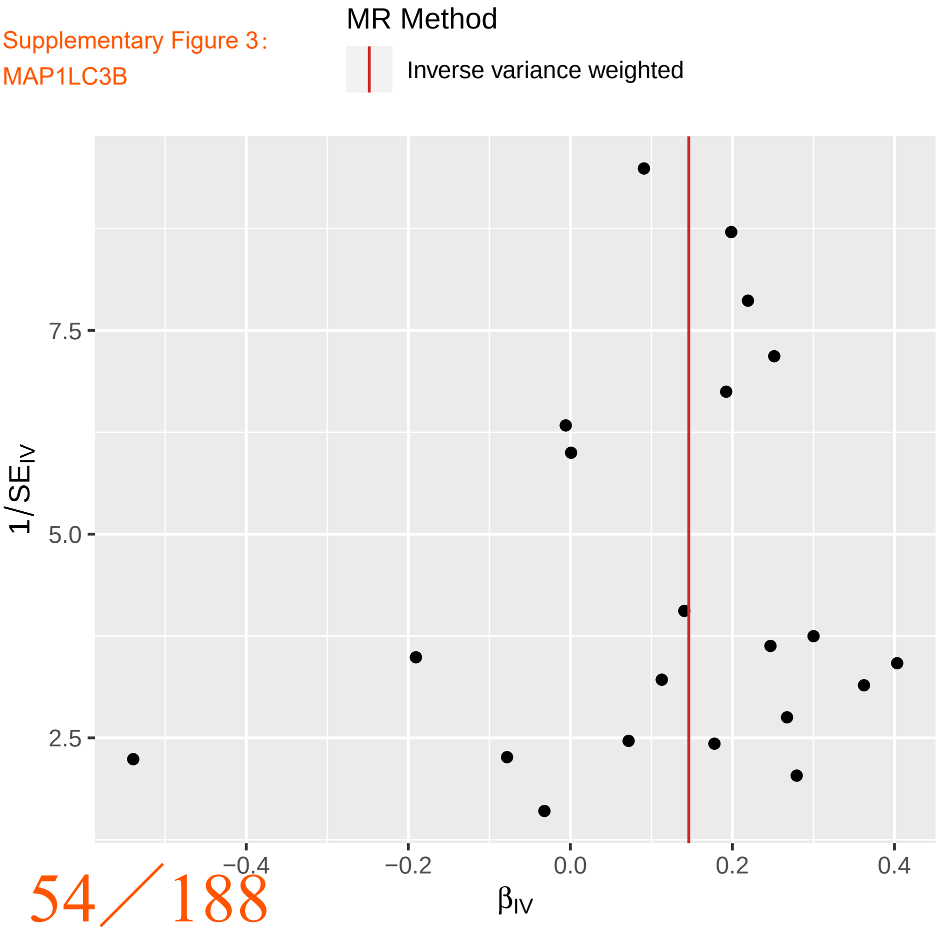

Supplementary Figure 3:
MPO

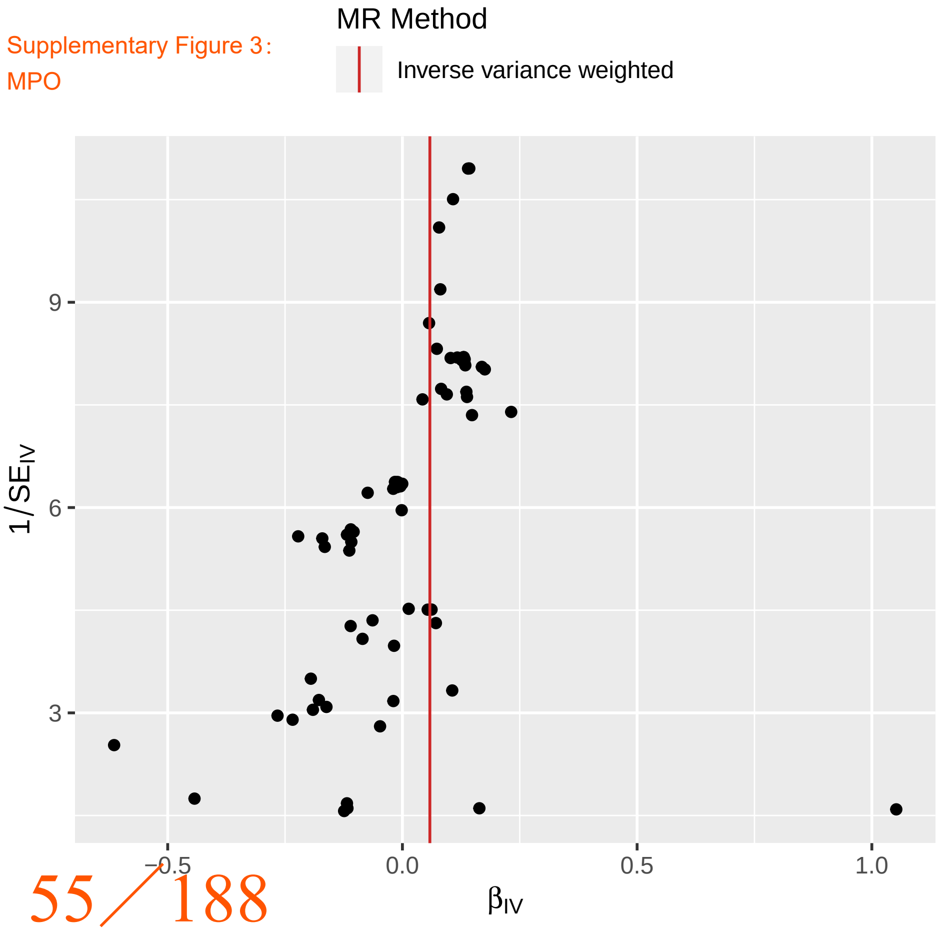

Supplementary Figure 3:
MTOR

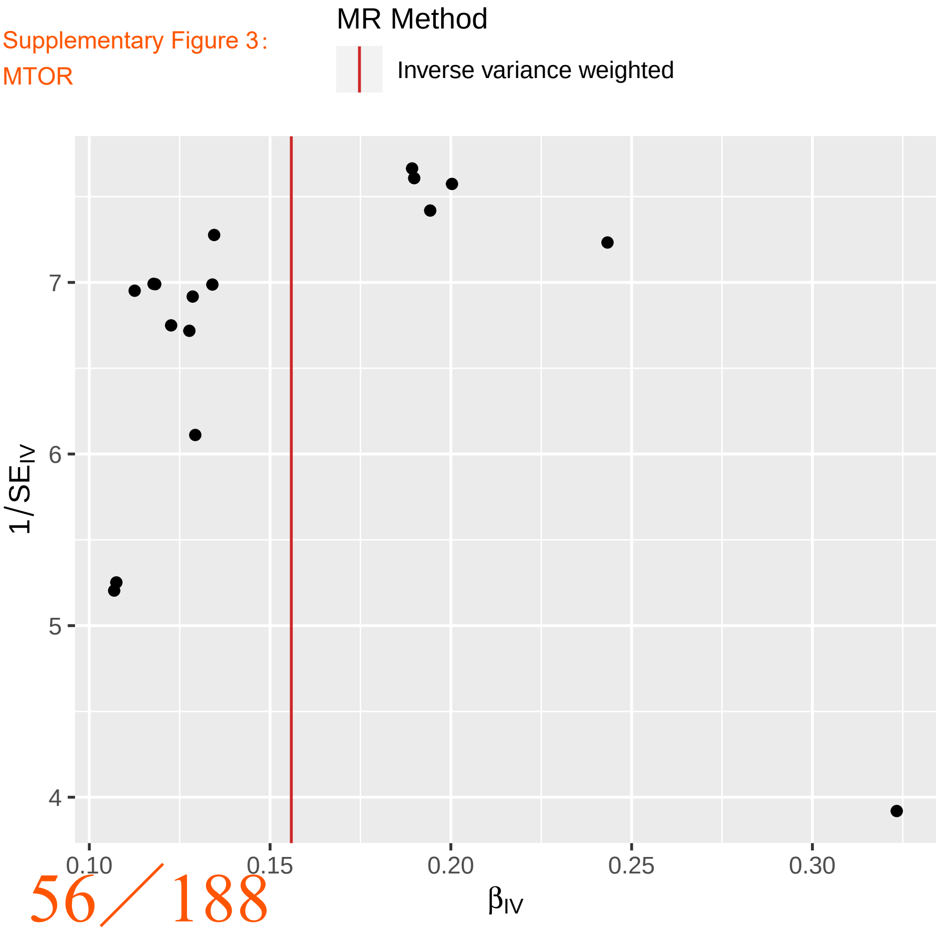

Supplementary Figure 3:
OGA

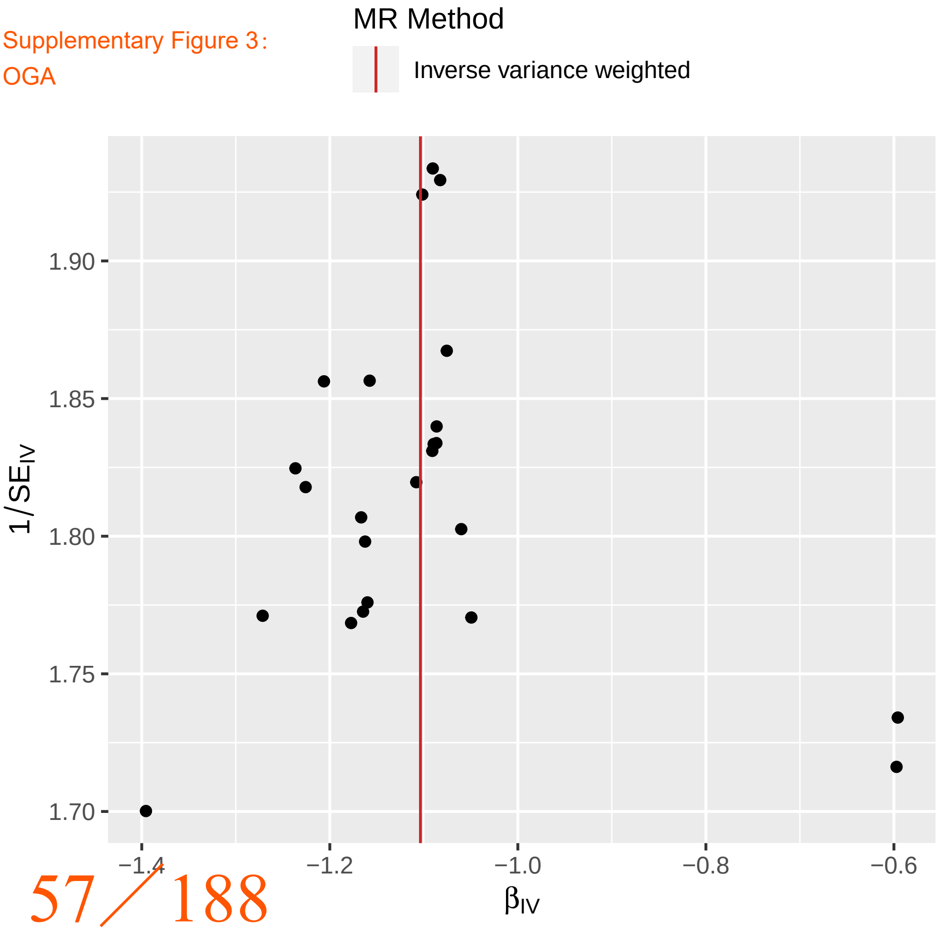

Supplementary Figure 3:
PPARA

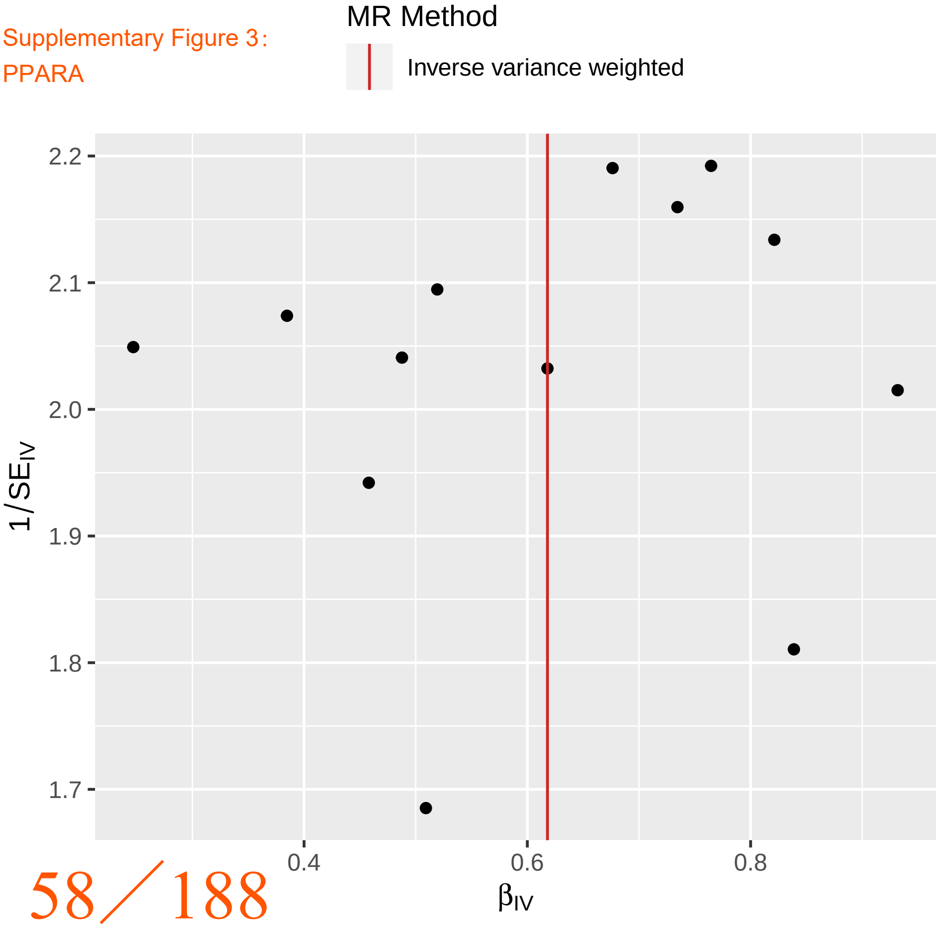

Supplementary Figure 3:
PTGS2

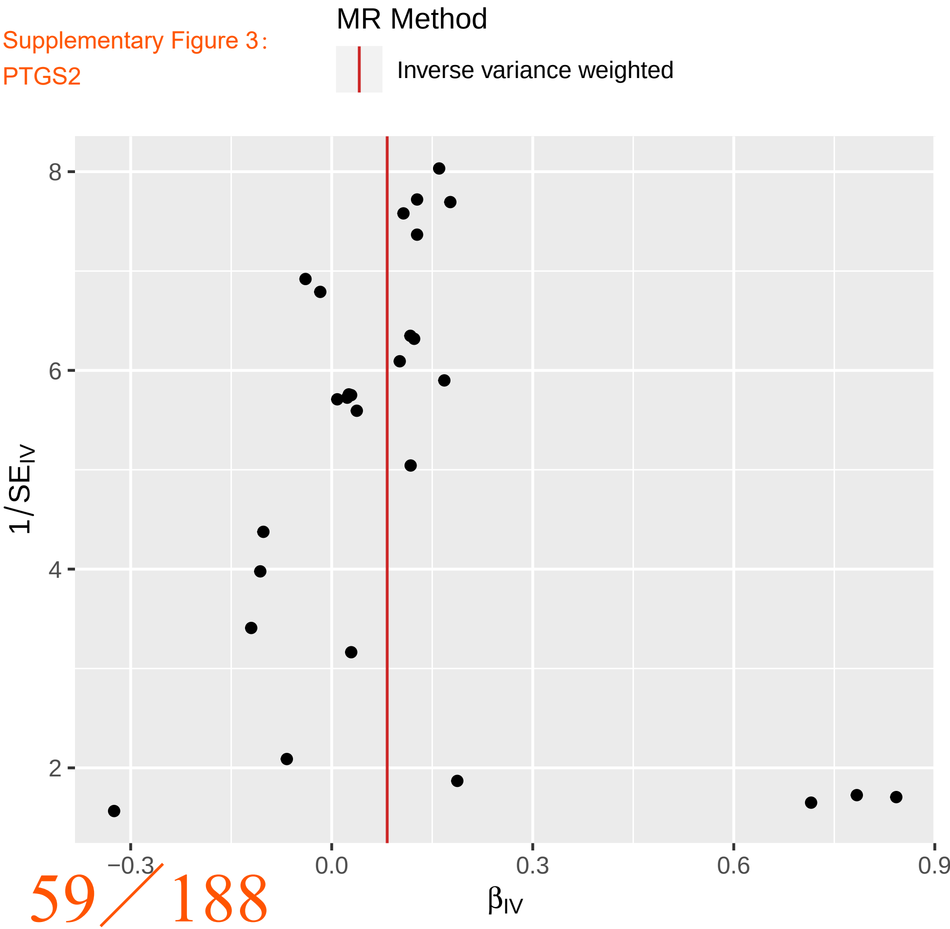

Supplementary Figure 3：
SIRT1

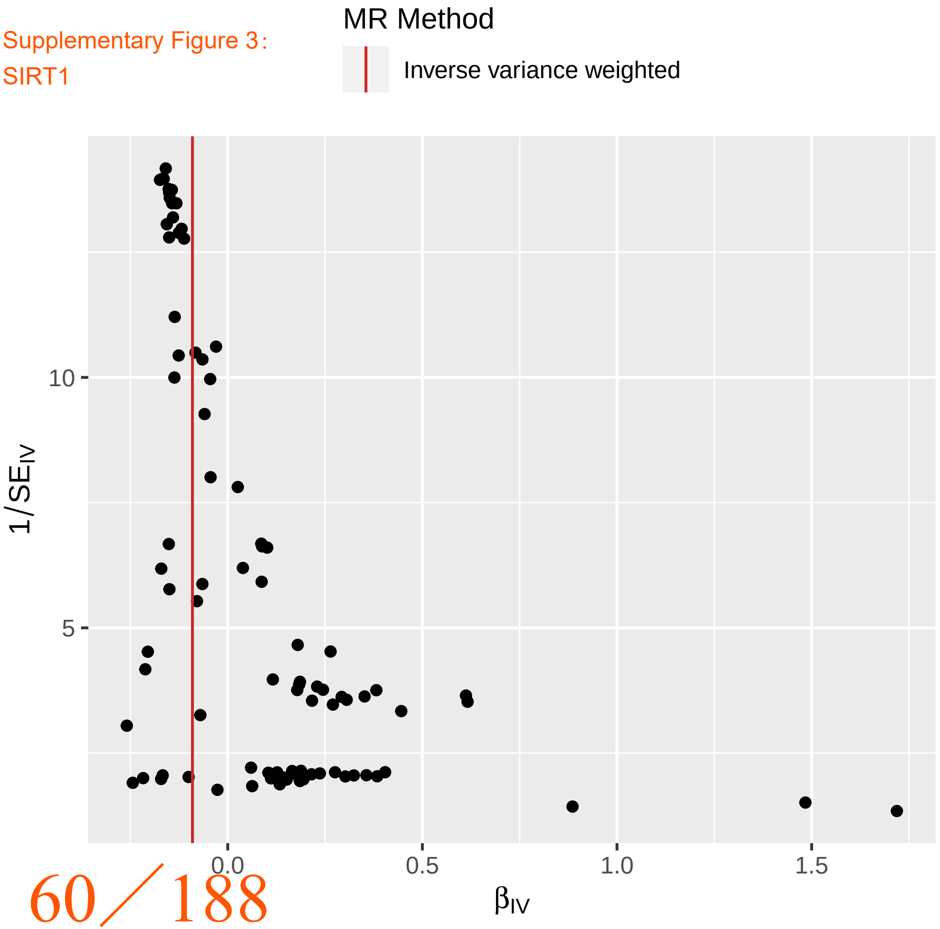

Supplementary Figure 3:
SREBF1

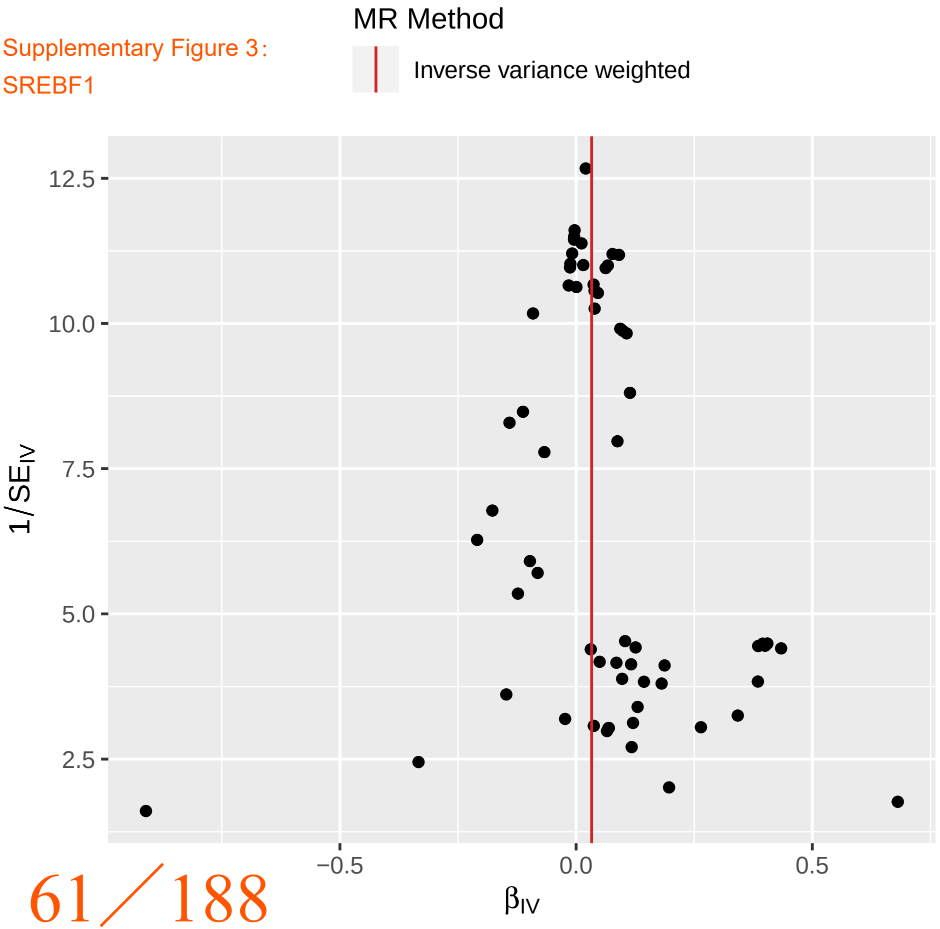

Supplementary Figure 3:

TNF

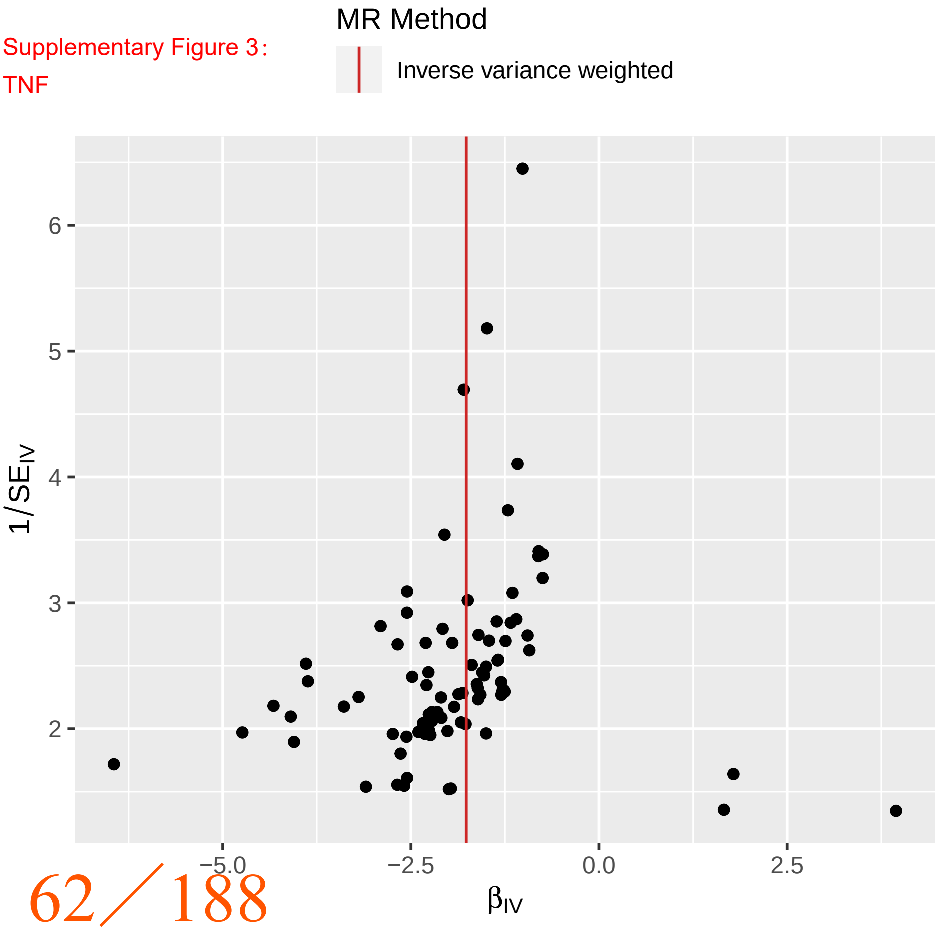

Supplementary Figure 3:
VIM

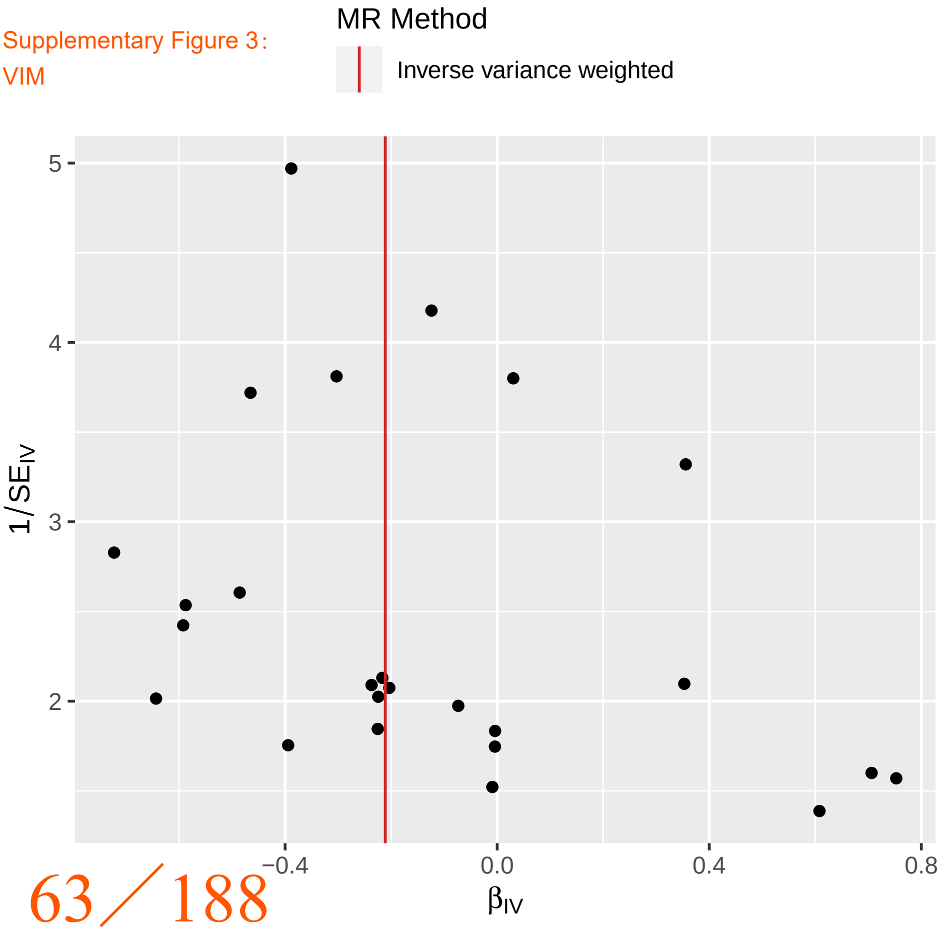

Supplementary Figure 4：ACTA2

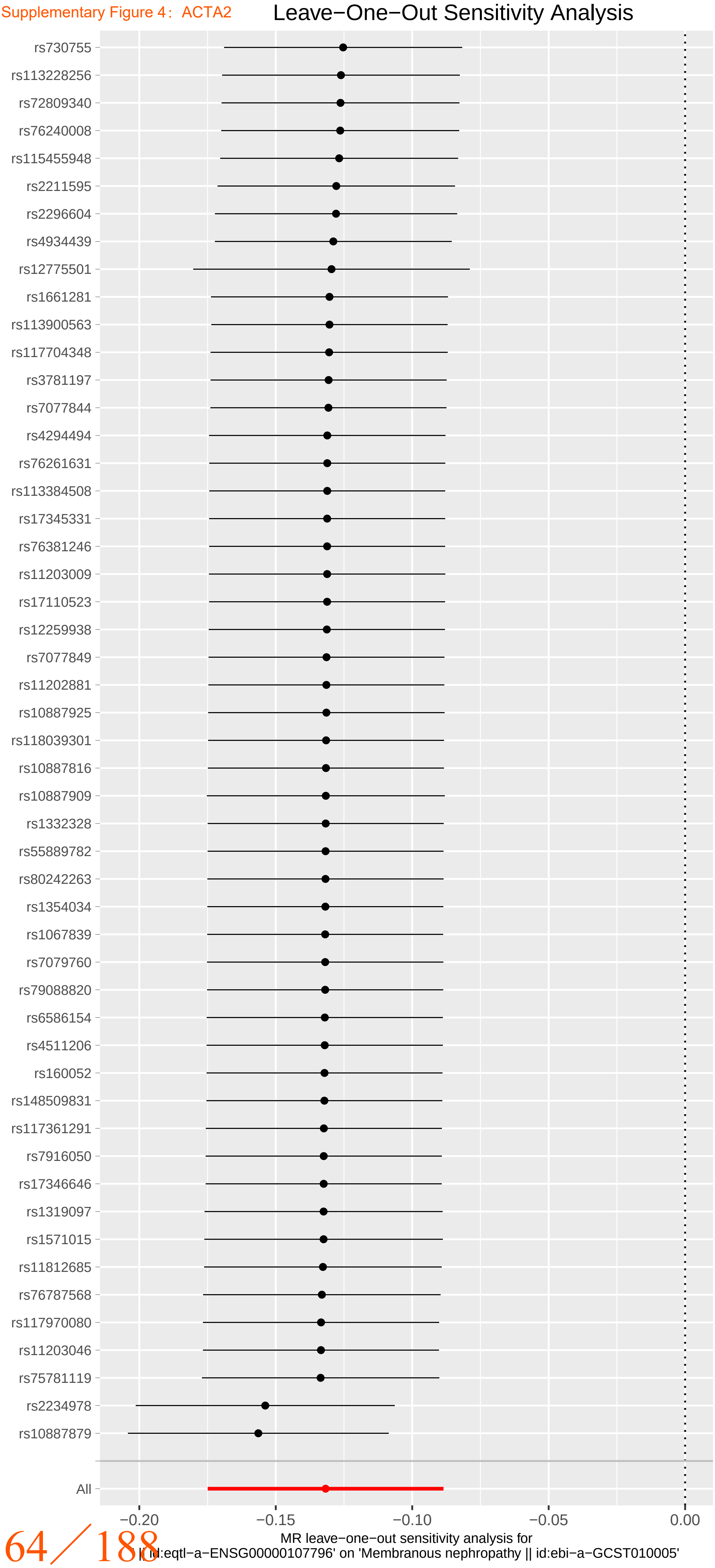

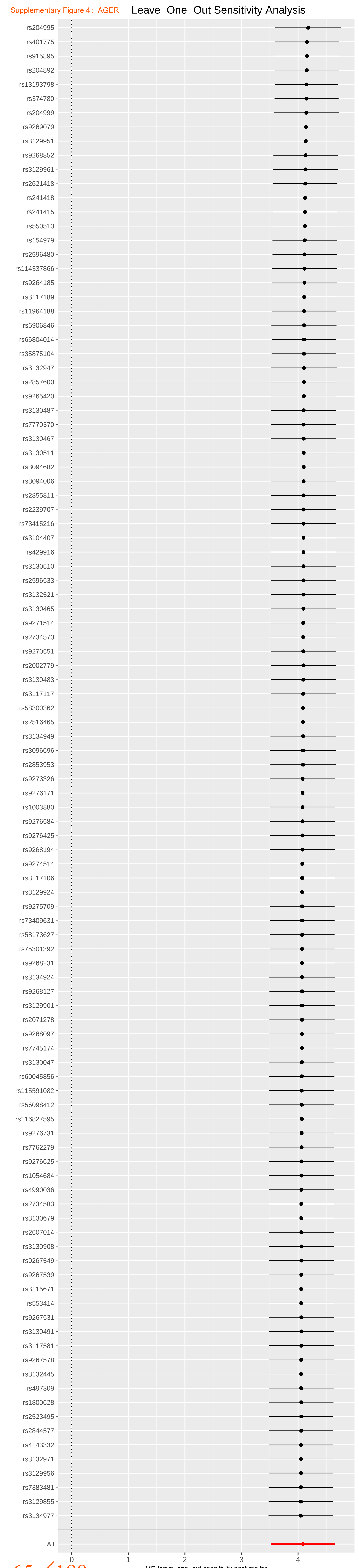

Supplementary Figure 4：AGER
Leave−One−Out Sensitivity Analysis
MR leave−one−out sensitivity analysis for
'eqtl−a−ENSG00000204305' on 'Membranous nephropathy || id:ebi−a−GCST010005'
All
0
1
2
3
4

Supplementary Figure 4：ATF6

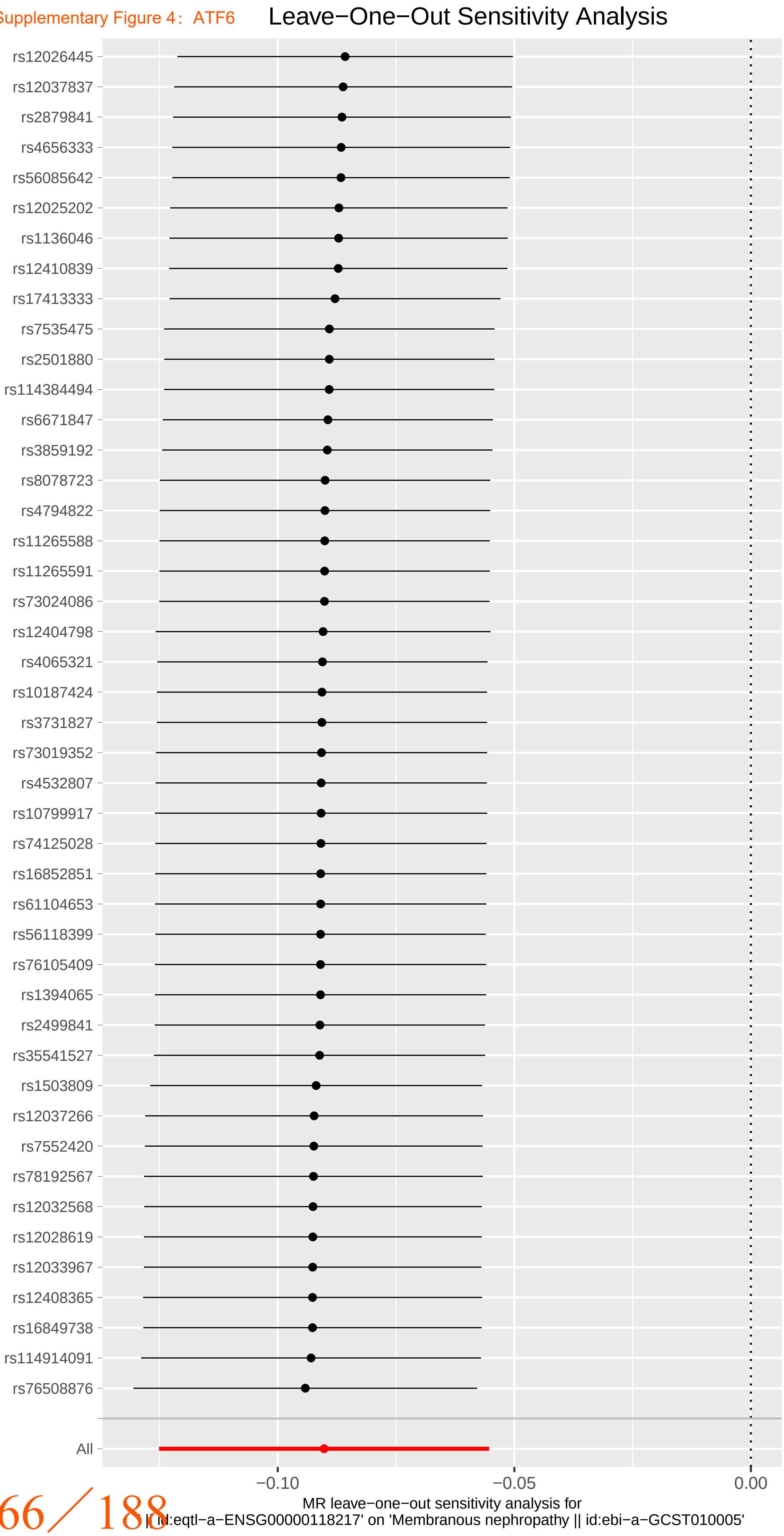

Supplementary Figure 4： BCL2

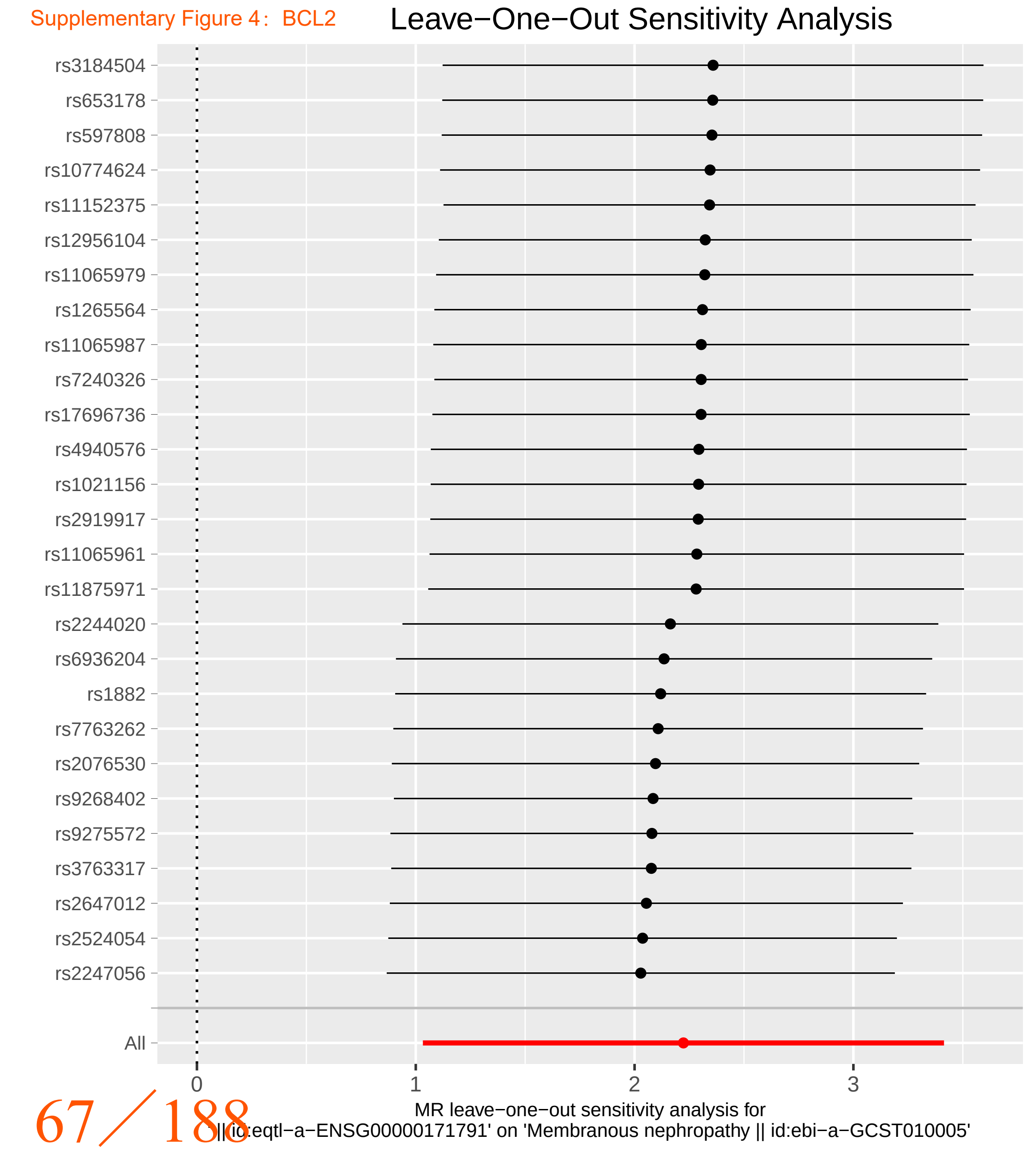

Supplementary Figure 4：CD36

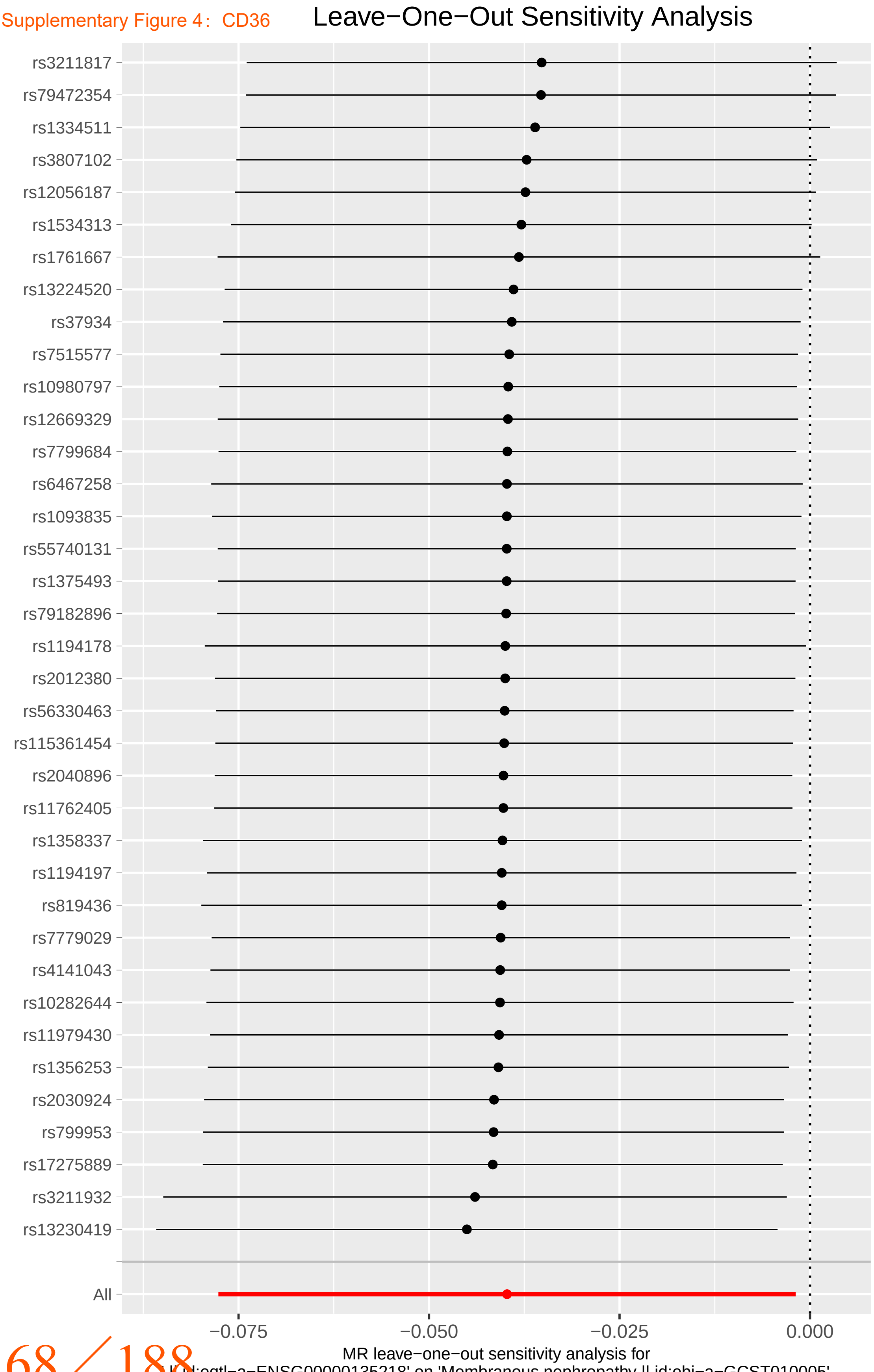

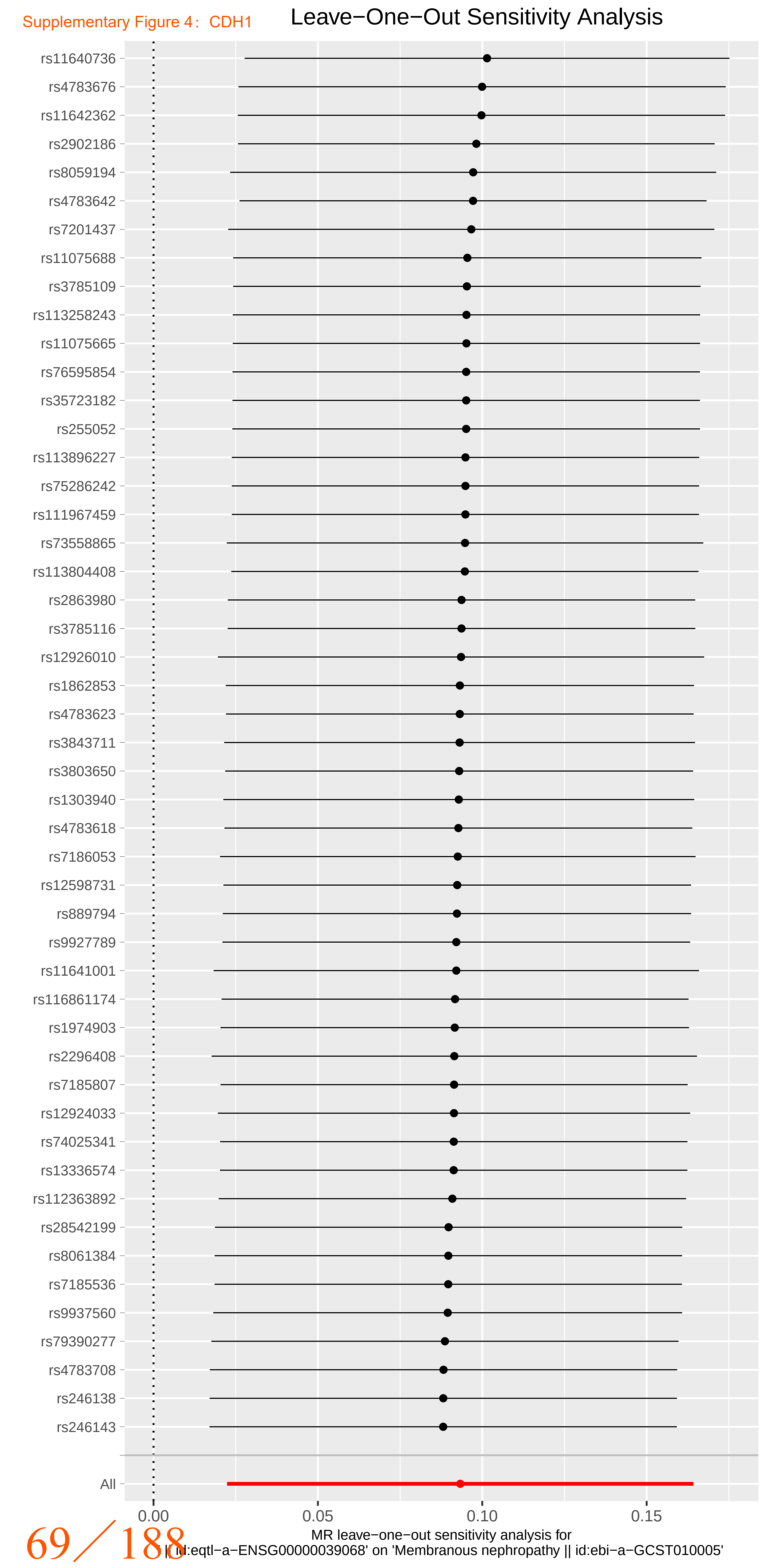
Leave−One−Out Sensitivity Analysis
rs11640736
rs4783676
rs11642362
rs2902186
rs8059194
rs4783642
rs7201437
rs11075688
rs3785109
rs113258243
rs11075665
rs76595854
rs35723182
rs255052
rs113896227
rs75286242
rs111967459
rs73558865
rs113804408
rs2863980
rs3785116
rs12926010
rs1862853
rs4783623
rs3843711
rs3803650
rs1303940
rs4783618
rs7186053
rs12598731
rs889794
rs9927789
rs11641001
rs116861174
rs1974903
rs2296408
rs7185807
rs12924033
rs74025341
rs13336574
rs112363892
rs28542199
rs8061384
rs7185536
rs9937560
rs79390277
rs4783708
rs246138
rs246143
All
0.00
0.05
0.10
0.15
MR leave−one−out sensitivity analysis for
'|| id:eqtl−a−ENSG00000039068' on 'Membranous nephropathy || id:ebi−a−GCST010005'

Supplementary Figure 4： DDIT3

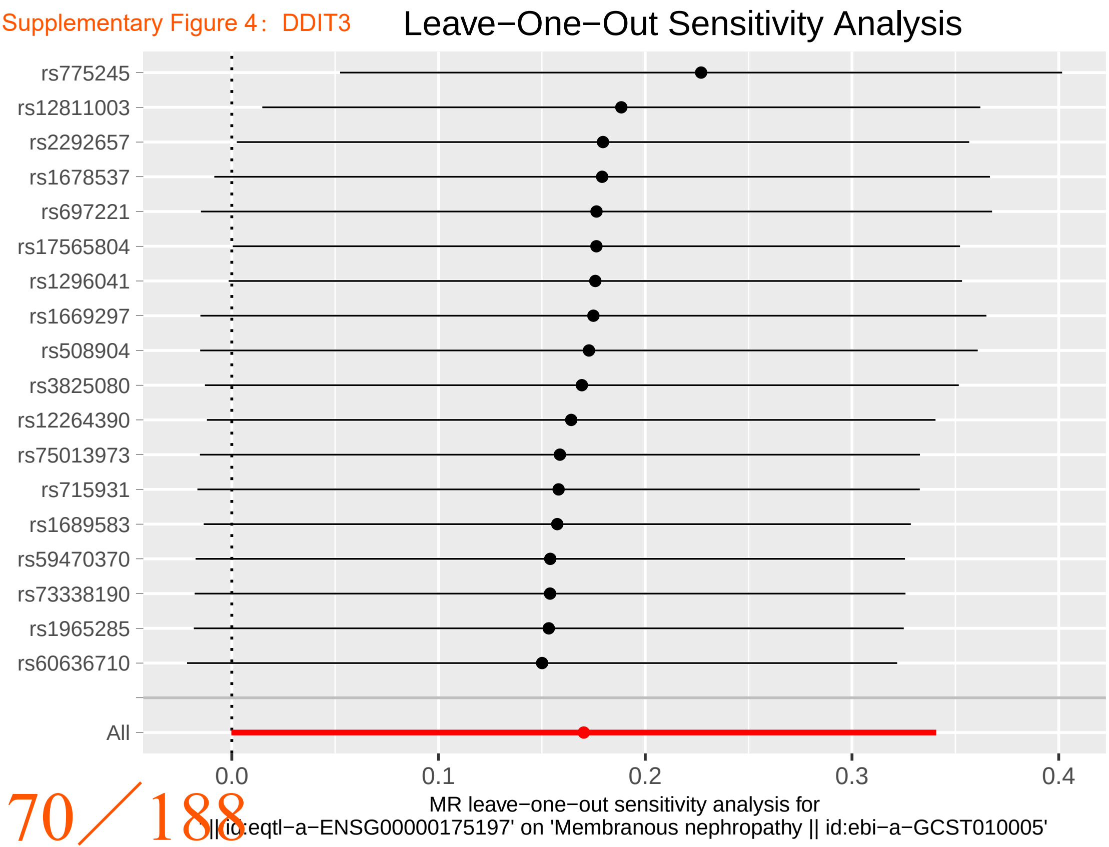

Supplementary Figure 4：FASN

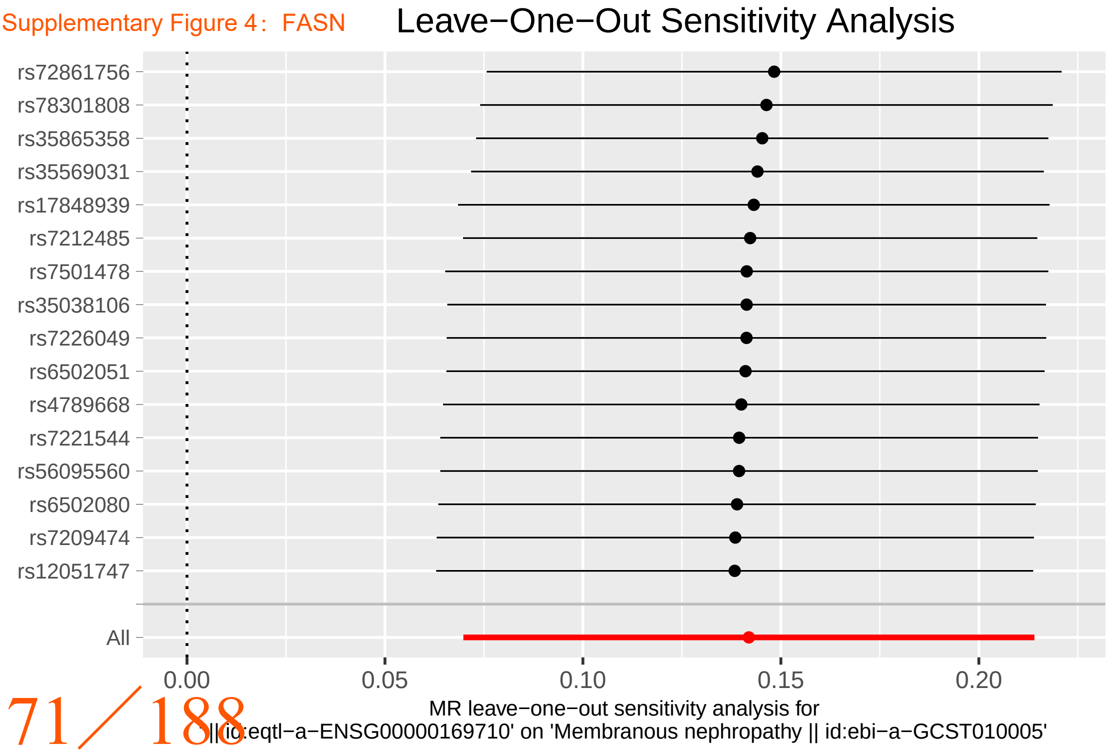

Supplementary Figure 4：IL1B

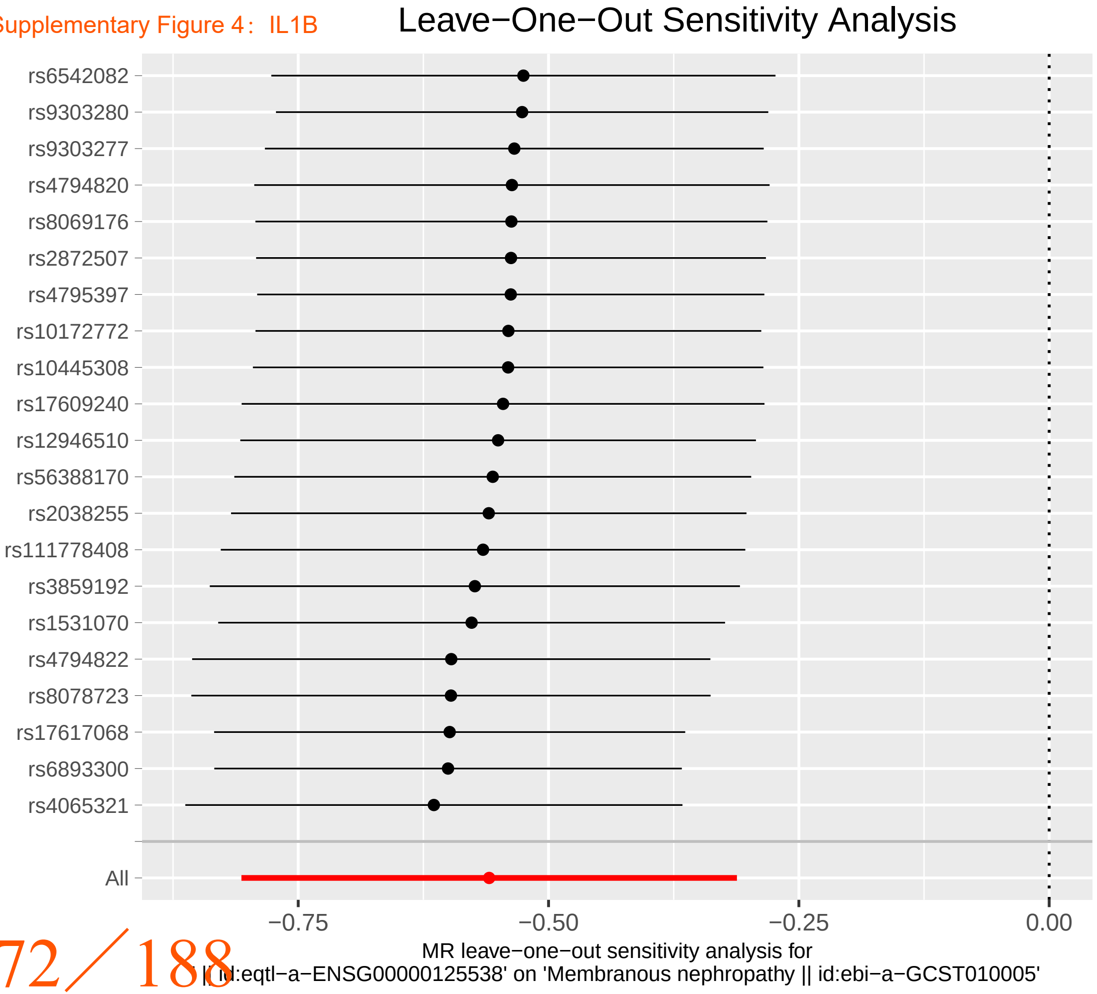

Supplementary Figure 4： INSR

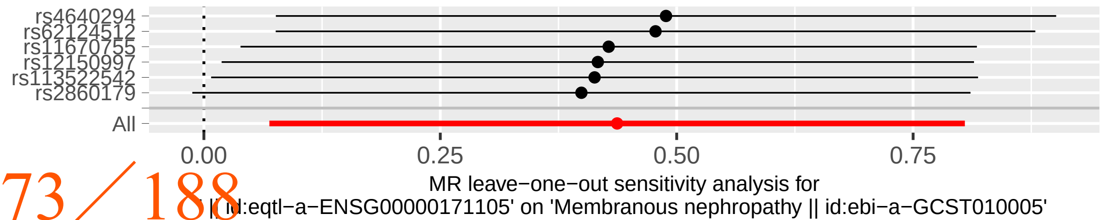

73／188

Supplementary Figure 4： JAK2

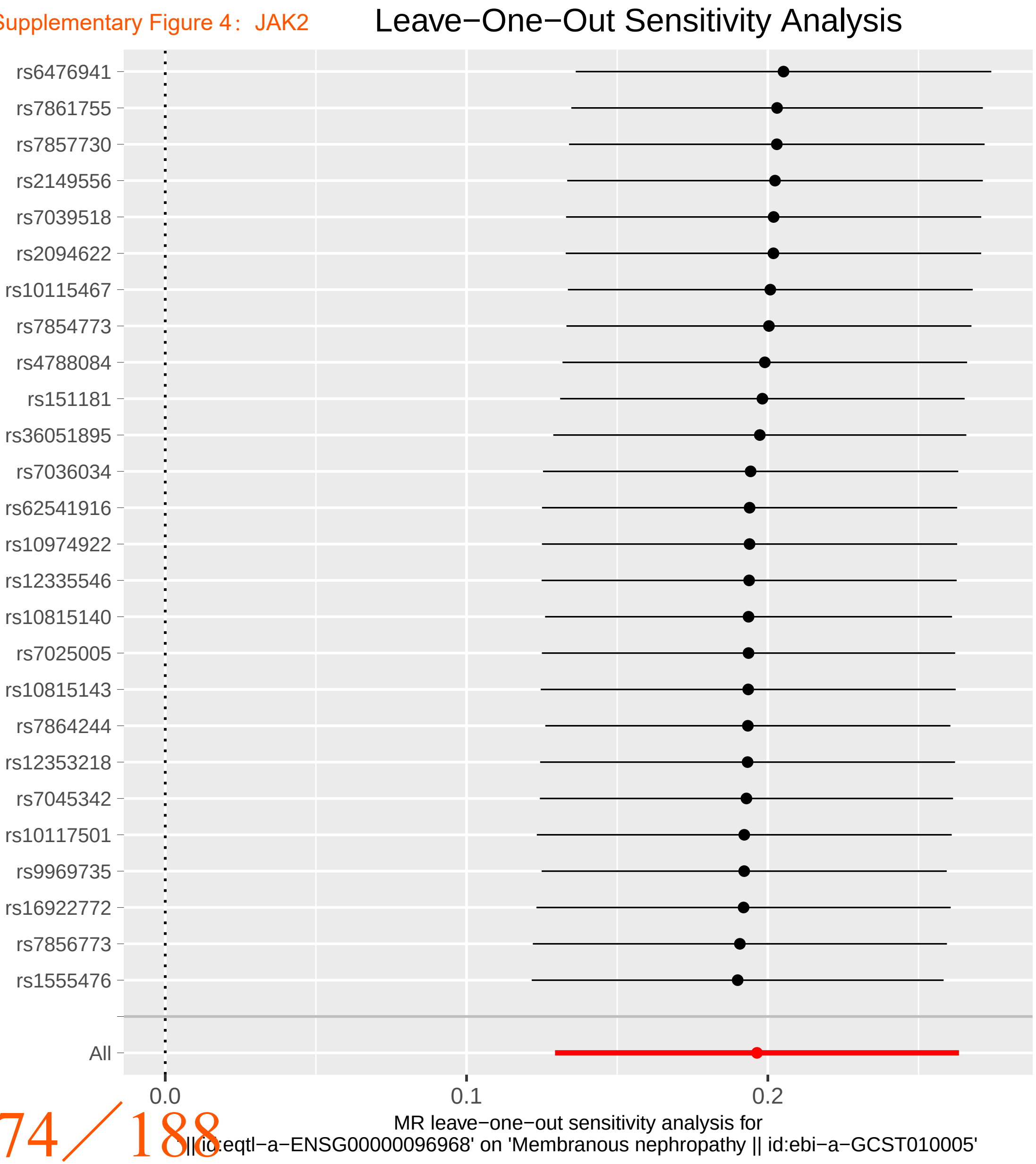

Supplementary Figure 4：MAP1LC3B

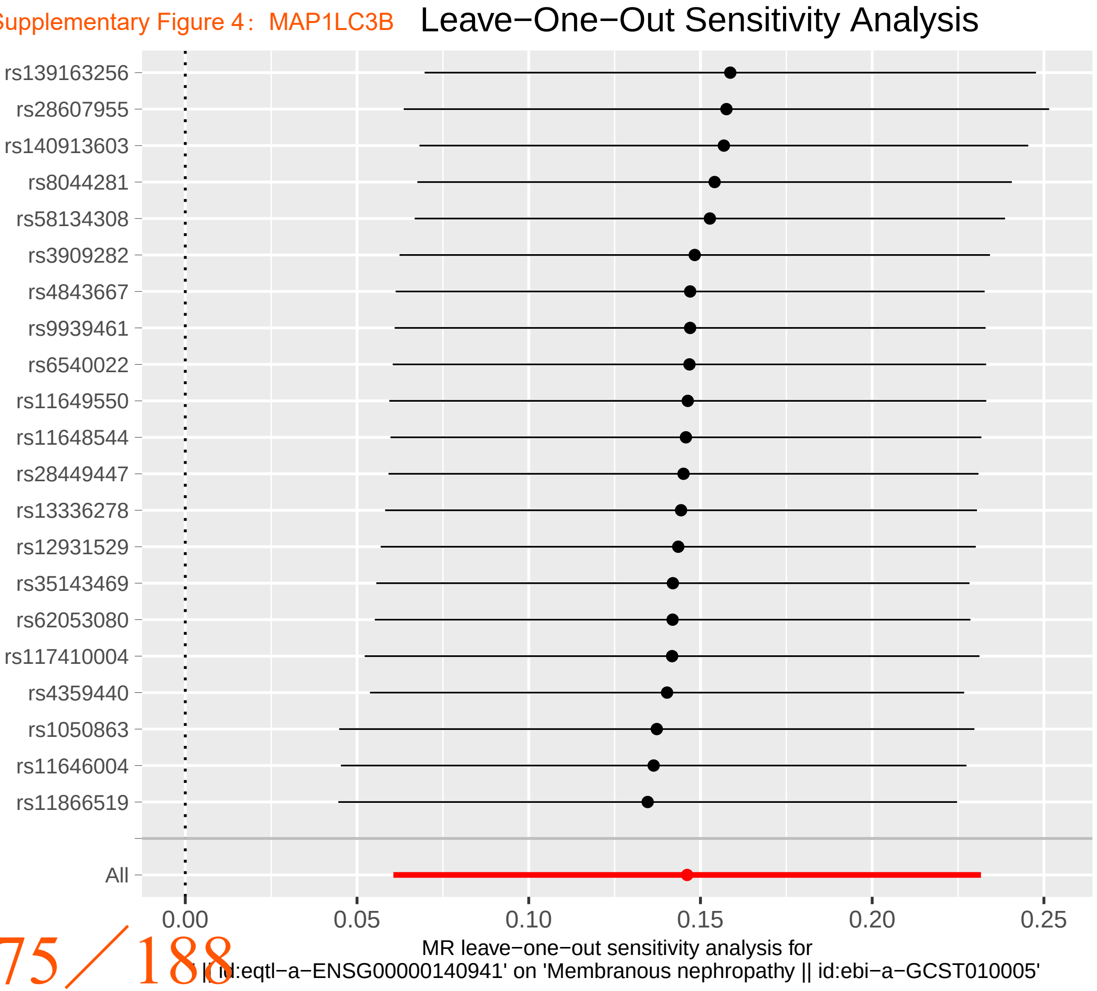

Supplementary Figure 4：MPO

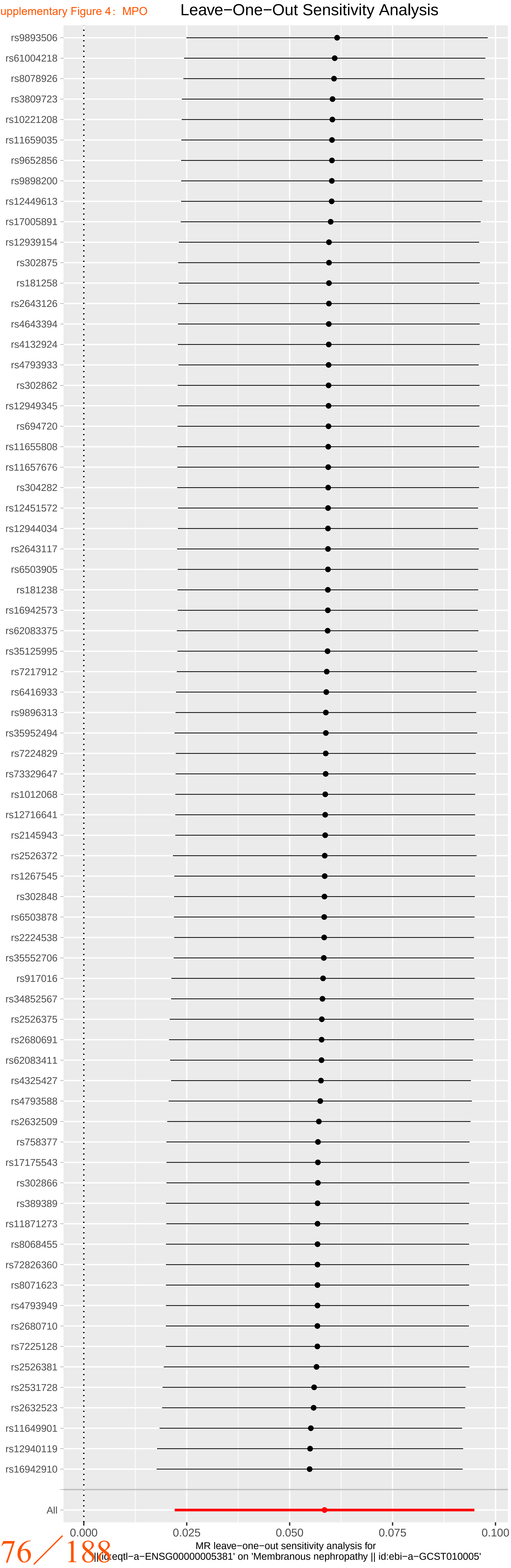

Supplementary Figure 4：MTOR Leave−One−Out Sensitivity Analysis

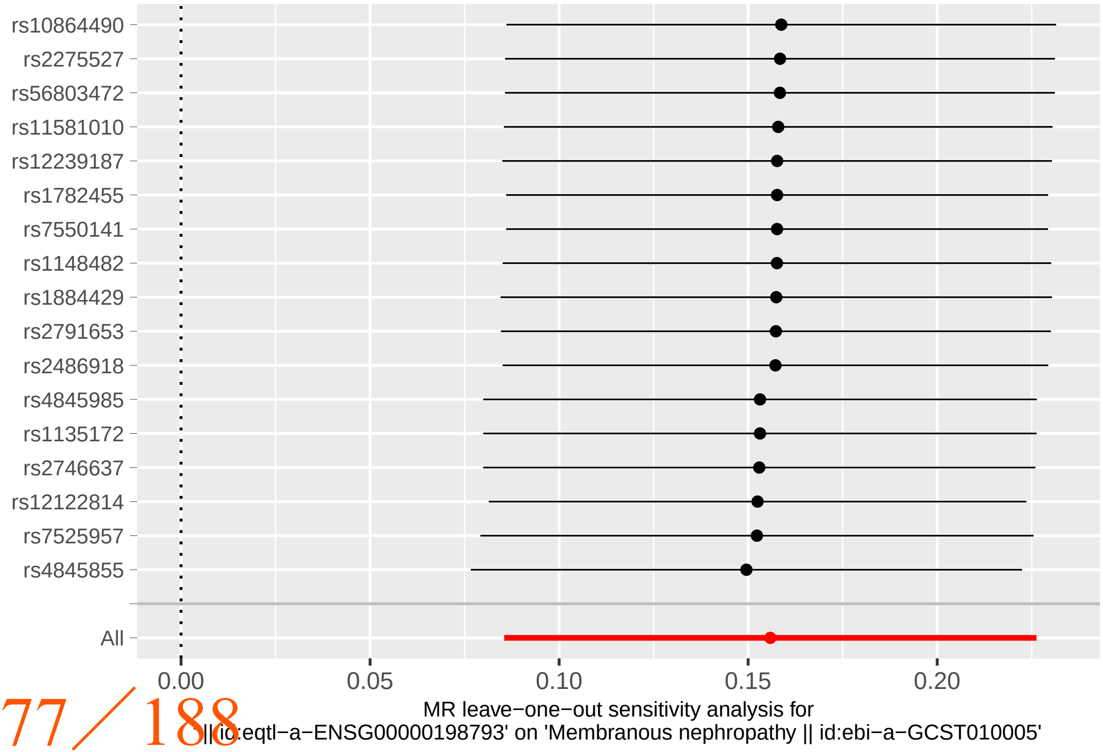

Supplementary Figure 4： OGA

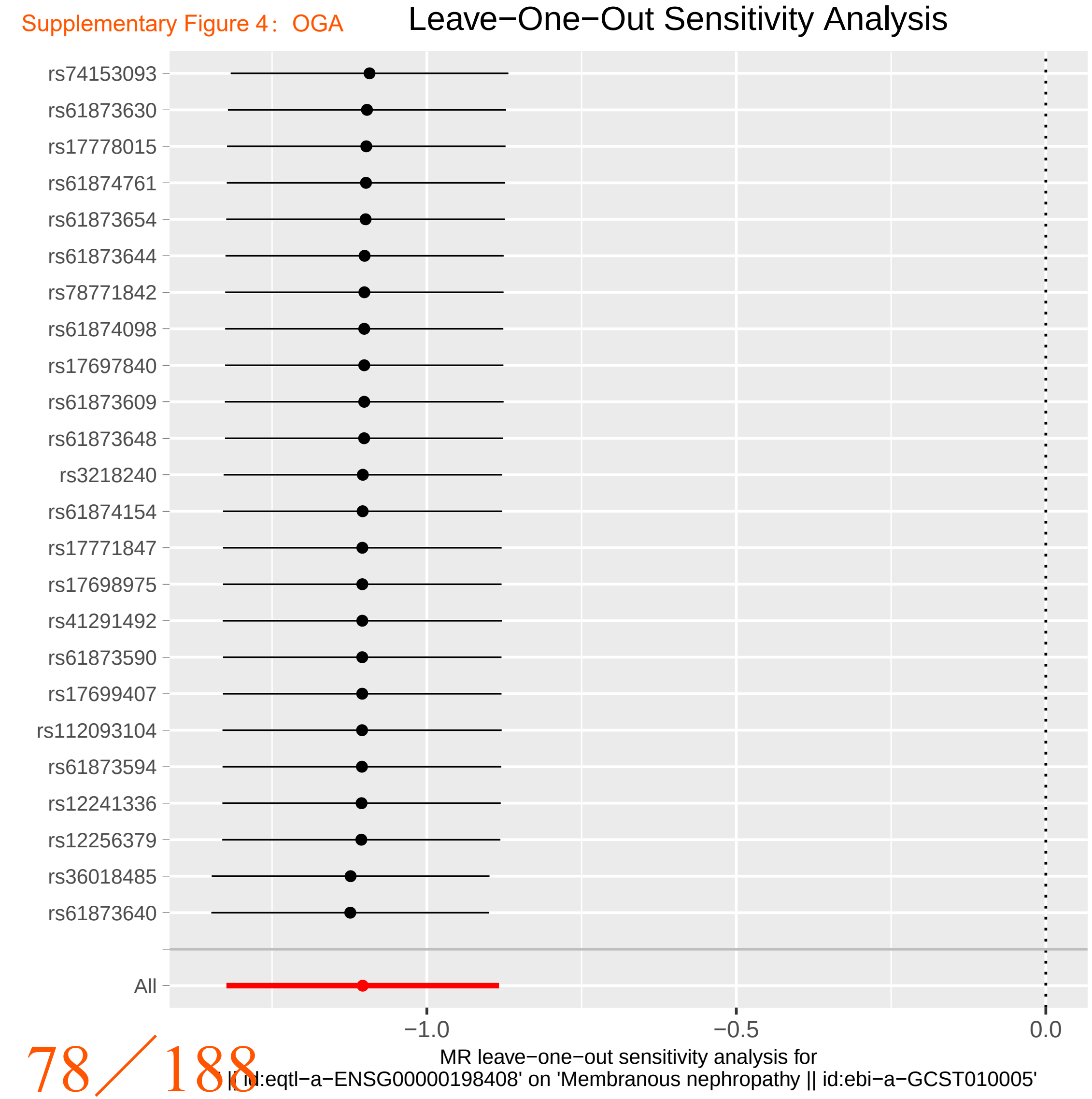

Supplementary Figure 4： PPARA

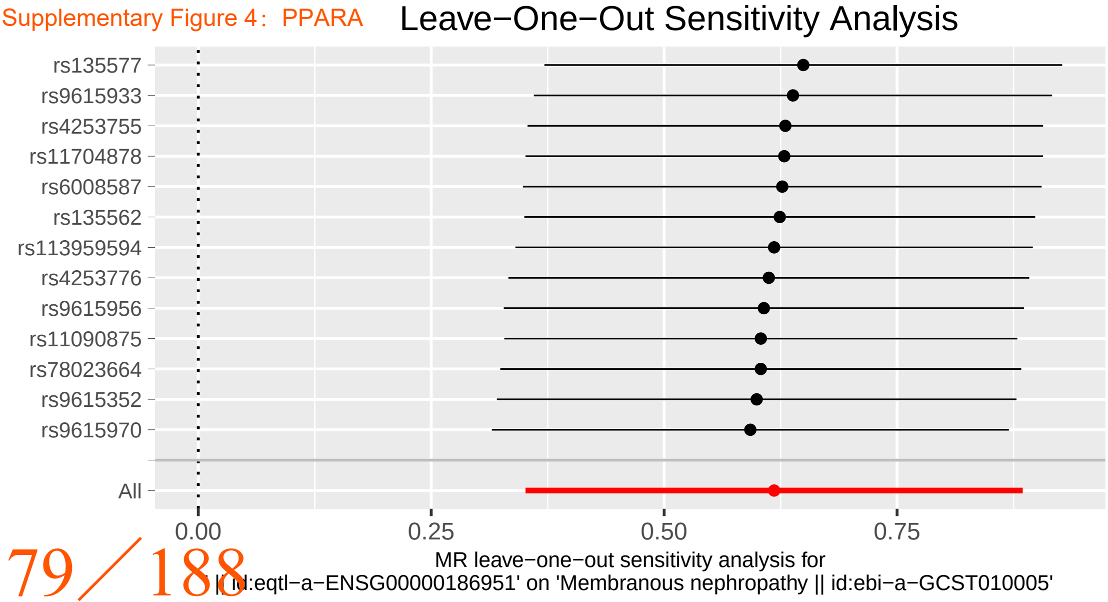

Supplementary Figure 4：PTGS2

Leave−One−Out Sensitivity Analysis

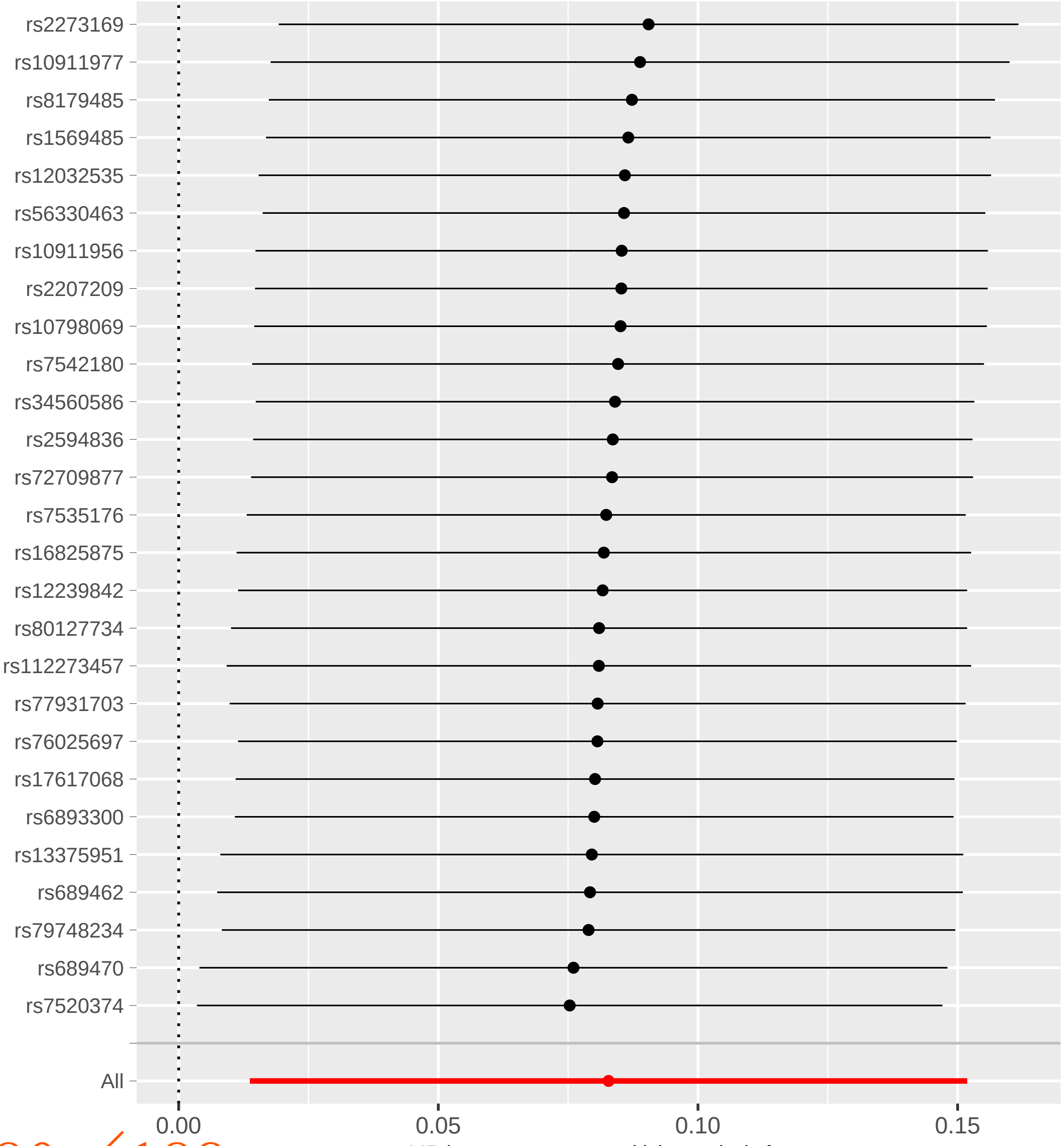

Supplementary Figure 4：SIRT1

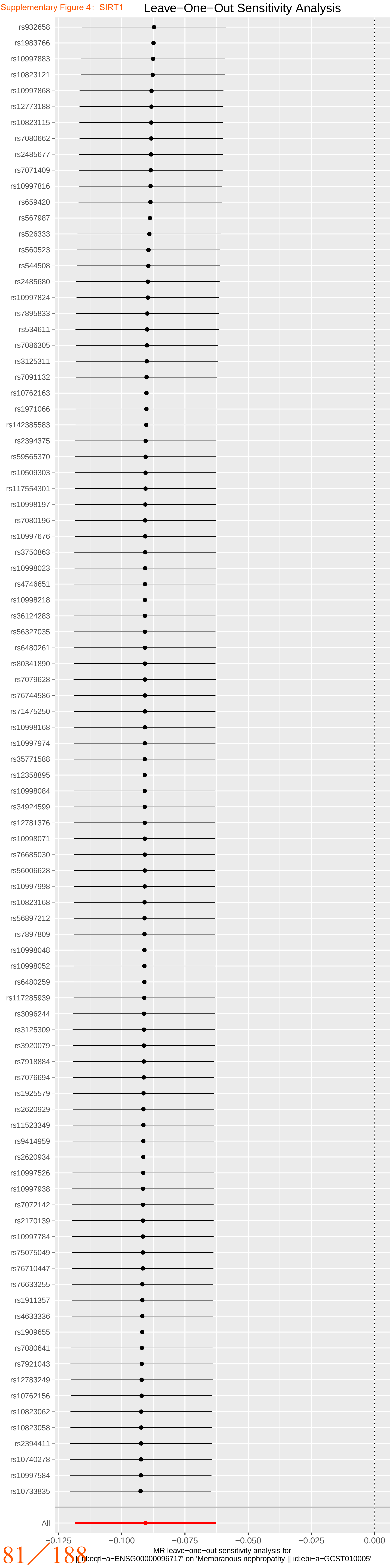

Supplementary Figure 4：SREBF1

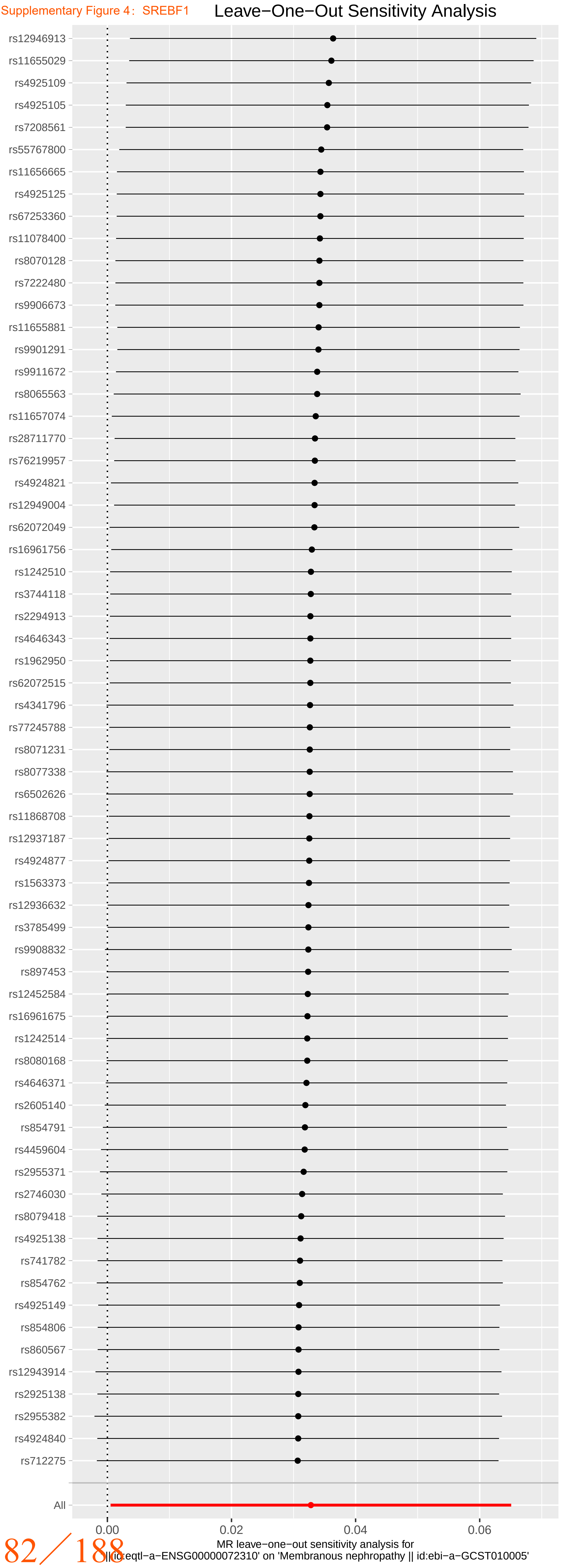

Supplementary Figure 4：TNF Leave−One−Out Sensitivity Analysis

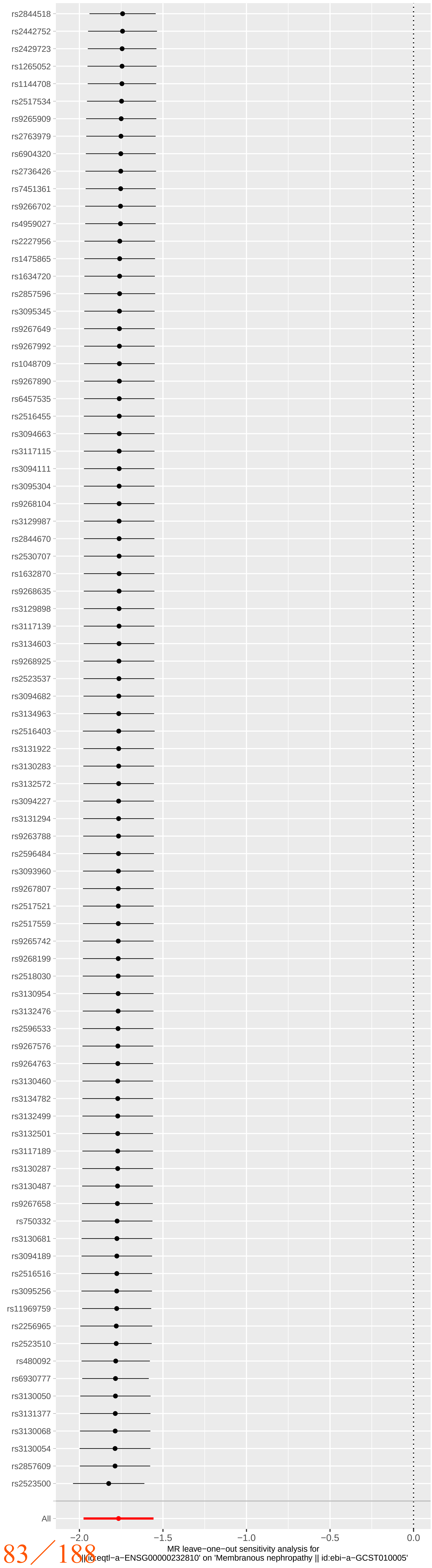

Supplementary Figure 4：VIM

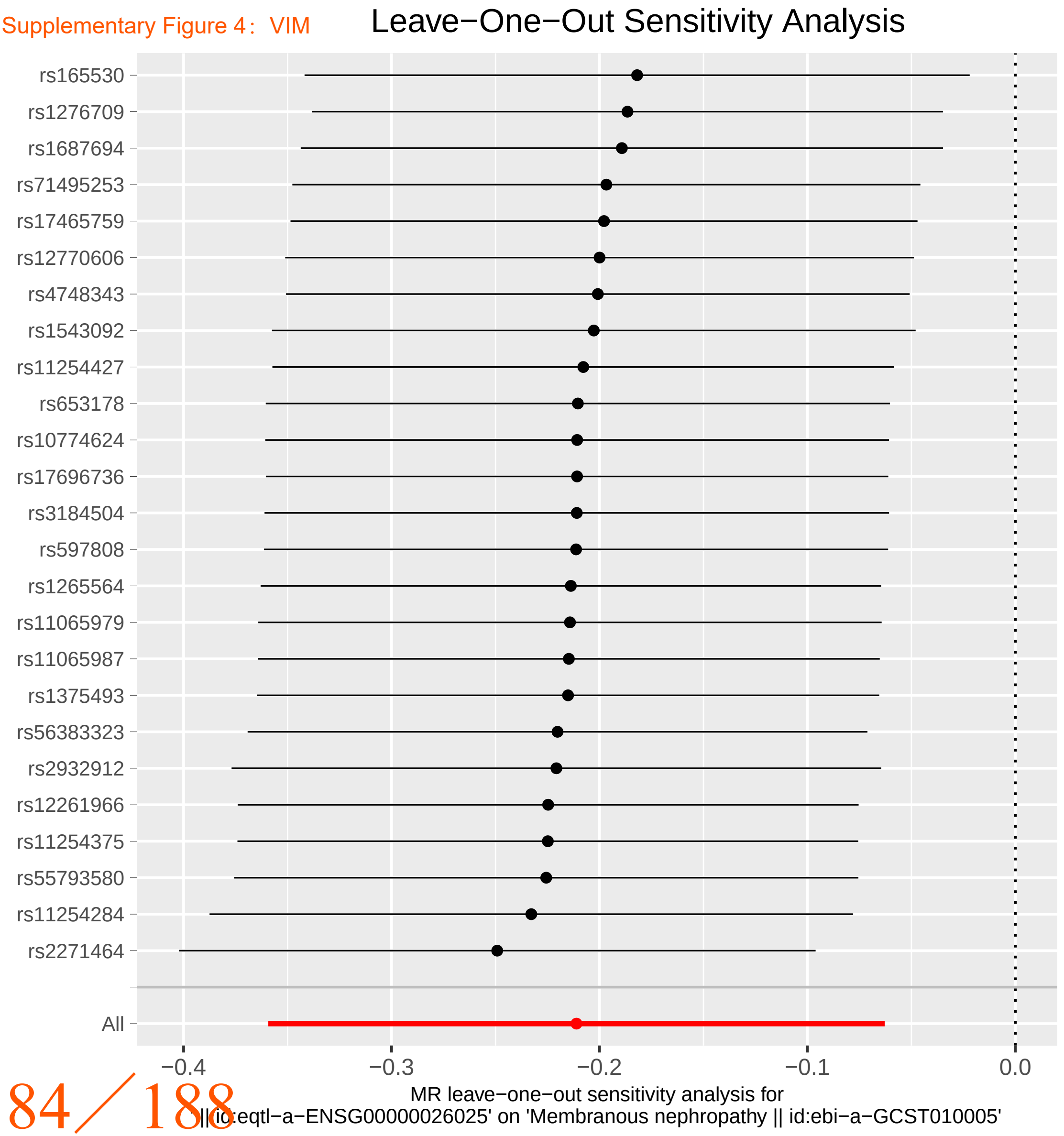

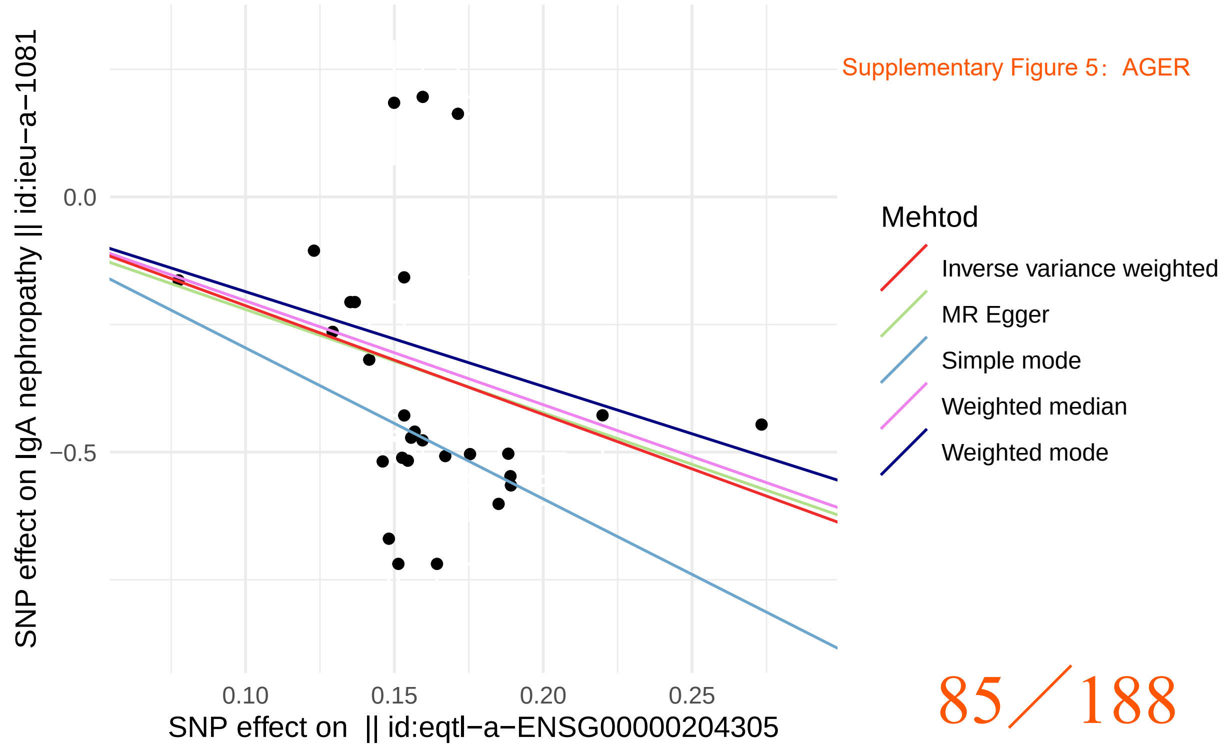

Supplementary Figure 5： AGER
Mehtod
Inverse variance weighted
MR Egger
Simple mode
Weighted median
Weighted mode
0.0
−0.5
0.10
0.15
0.20
0.25
SNP effect on || id:eqtl−a−ENSG00000204305
SNP effect on IgA nephropathy || id:ieu−a−1081

Supplementary Figure 5: ATF6

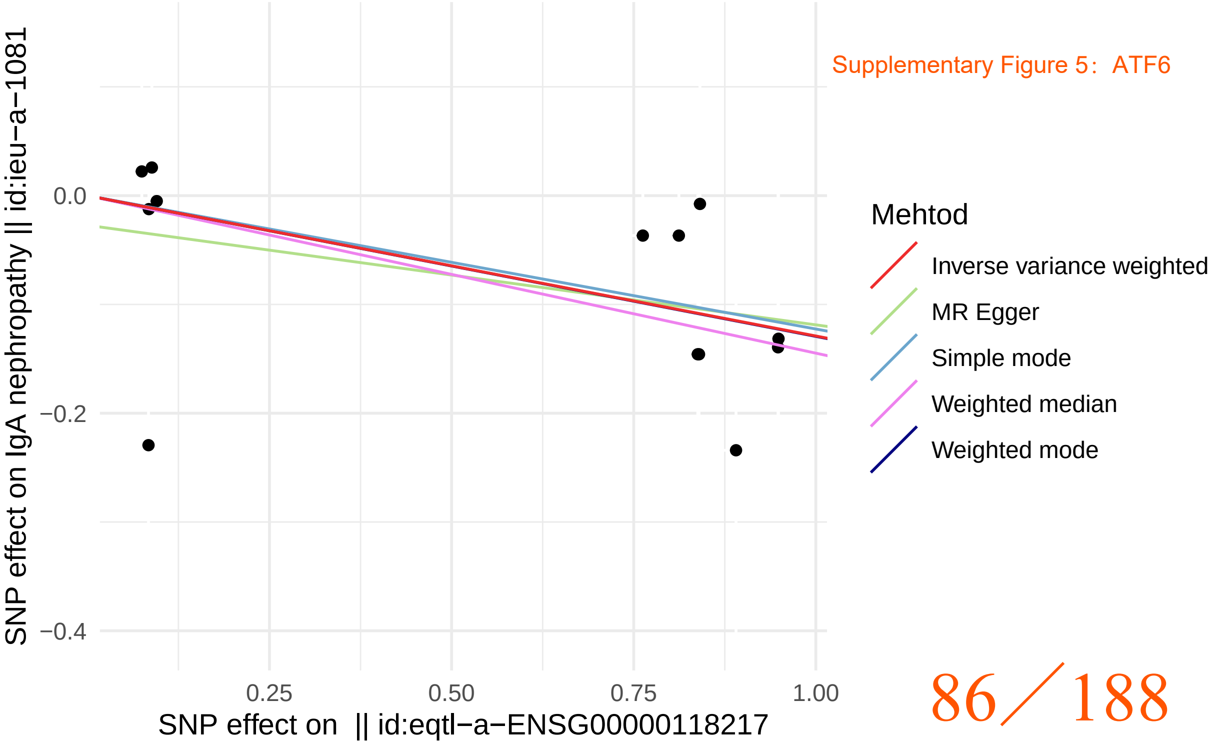

86／188

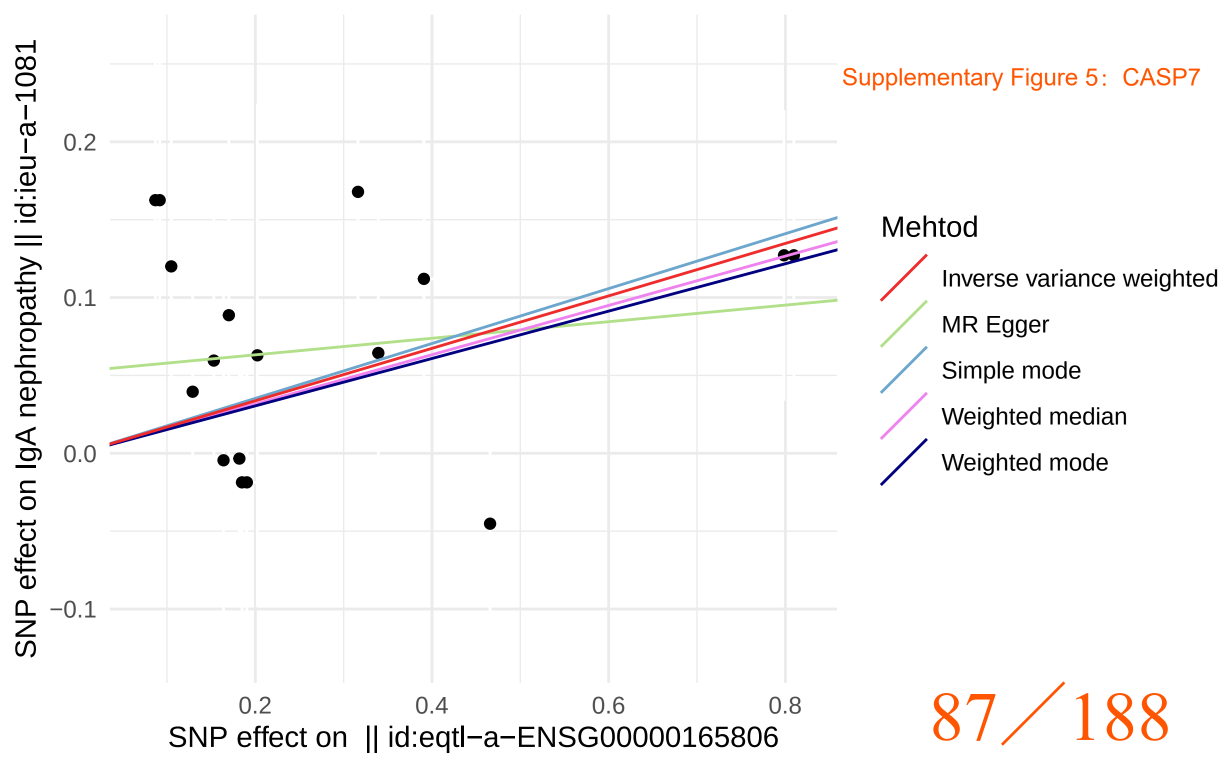
Supplementary Figure 5：CASP7
Mehtod
Inverse variance weighted
MR Egger
Simple mode
Weighted median
Weighted mode
SNP effect on IgA nephropathy || id:ieu−a−1081
0.2
0.1
0.0
−0.1
0.2
0.4
0.6
0.8
SNP effect on || id:eqtl−a−ENSG00000165806

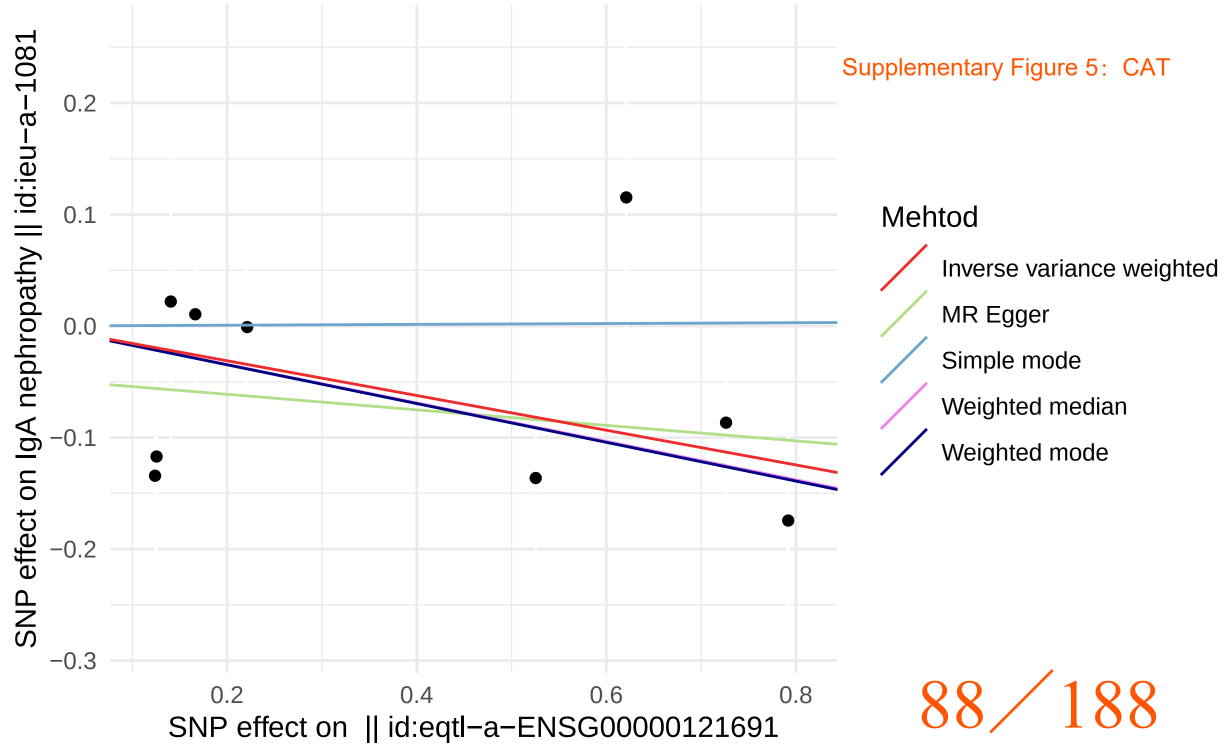
Supplementary Figure 5：CAT
SNP effect on IgA nephropathy || id:ieu−a−1081
0.2
0.1
0.0
−0.1
−0.2
−0.3
0.2
0.4
0.6
0.8
SNP effect on || id:eqtl−a−ENSG00000121691
Mehtod
Inverse variance weighted
MR Egger
Simple mode
Weighted median
Weighted mode

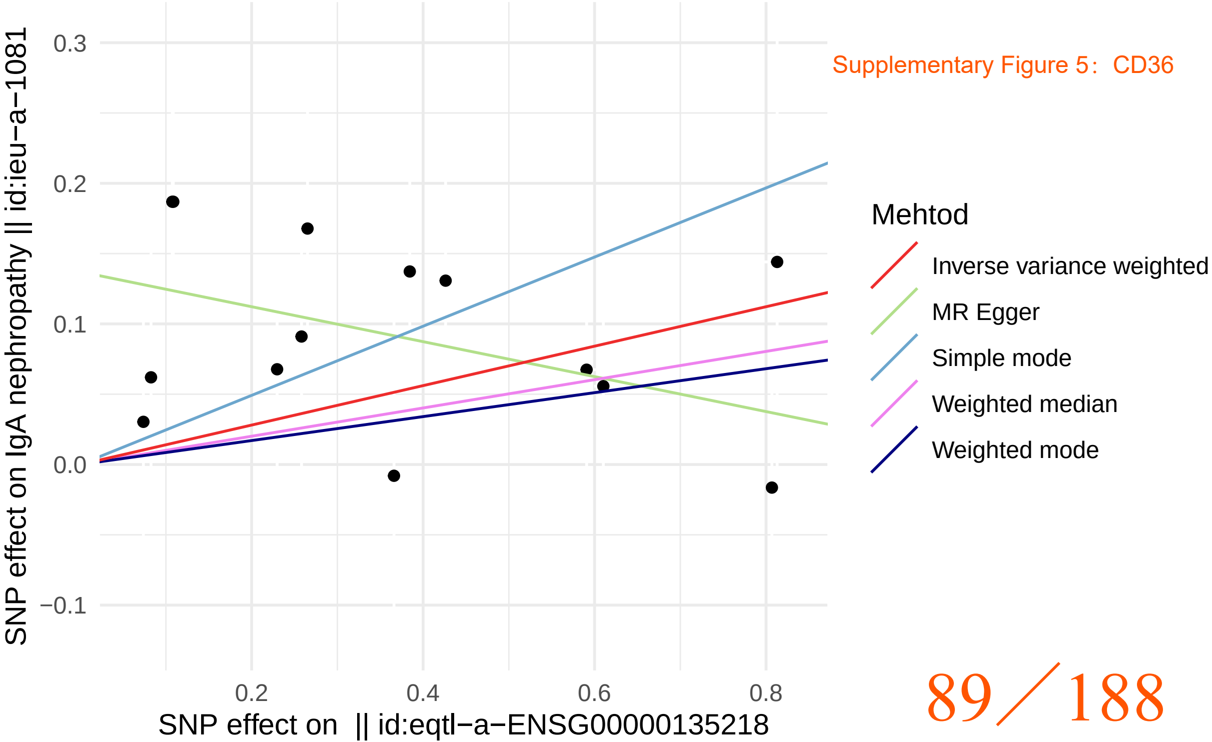

Supplementary Figure 5：CD36

Supplementary Figure 5： ELF2

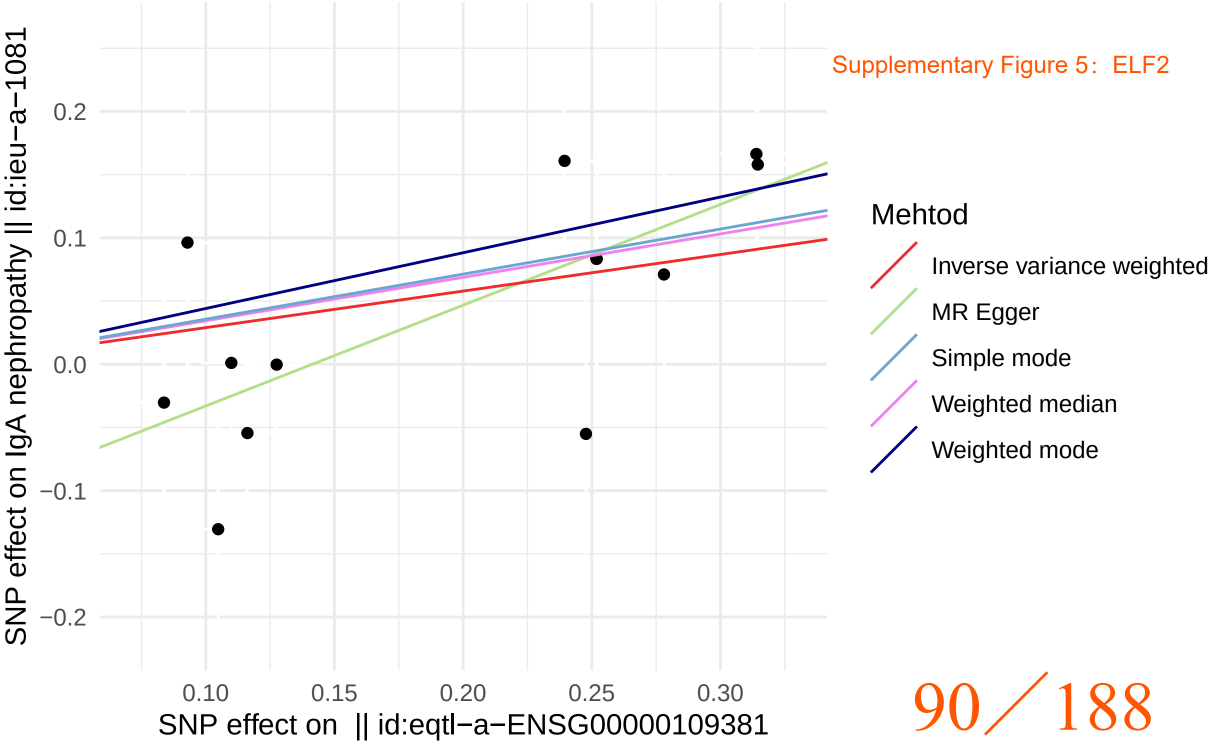

Supplementary Figure 5： JAK2

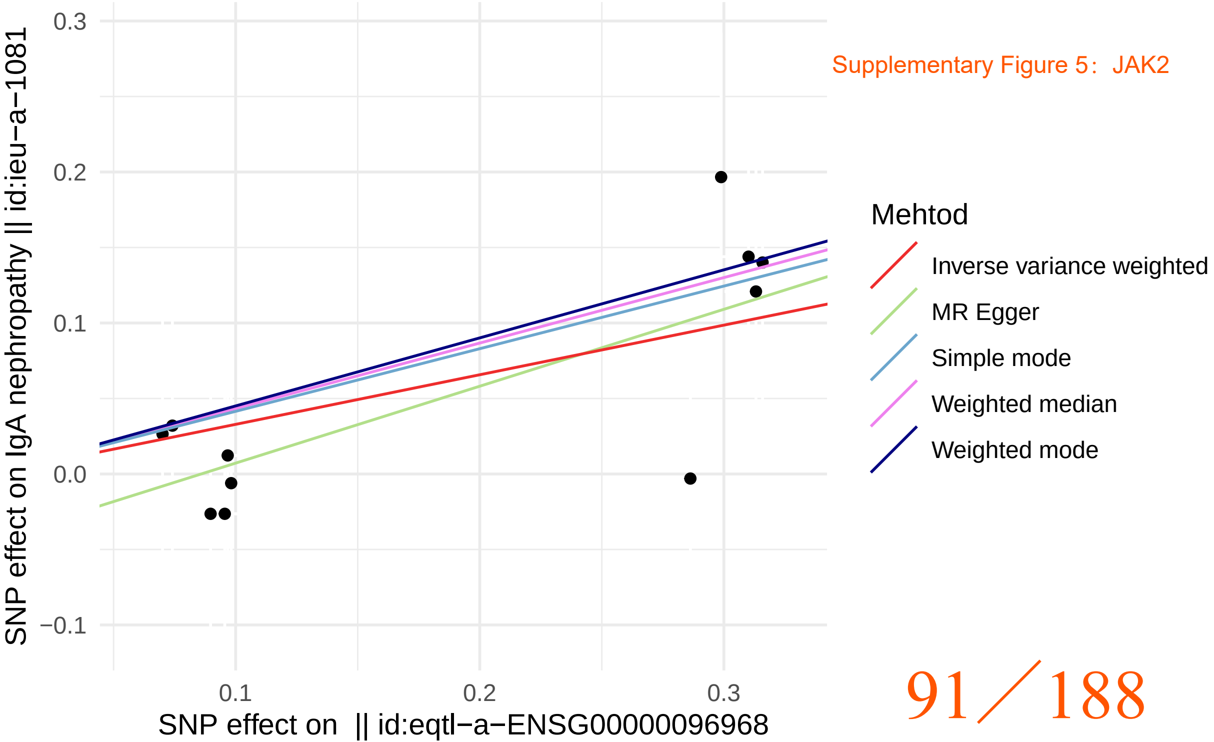

Supplementary Figure 5：LCN2

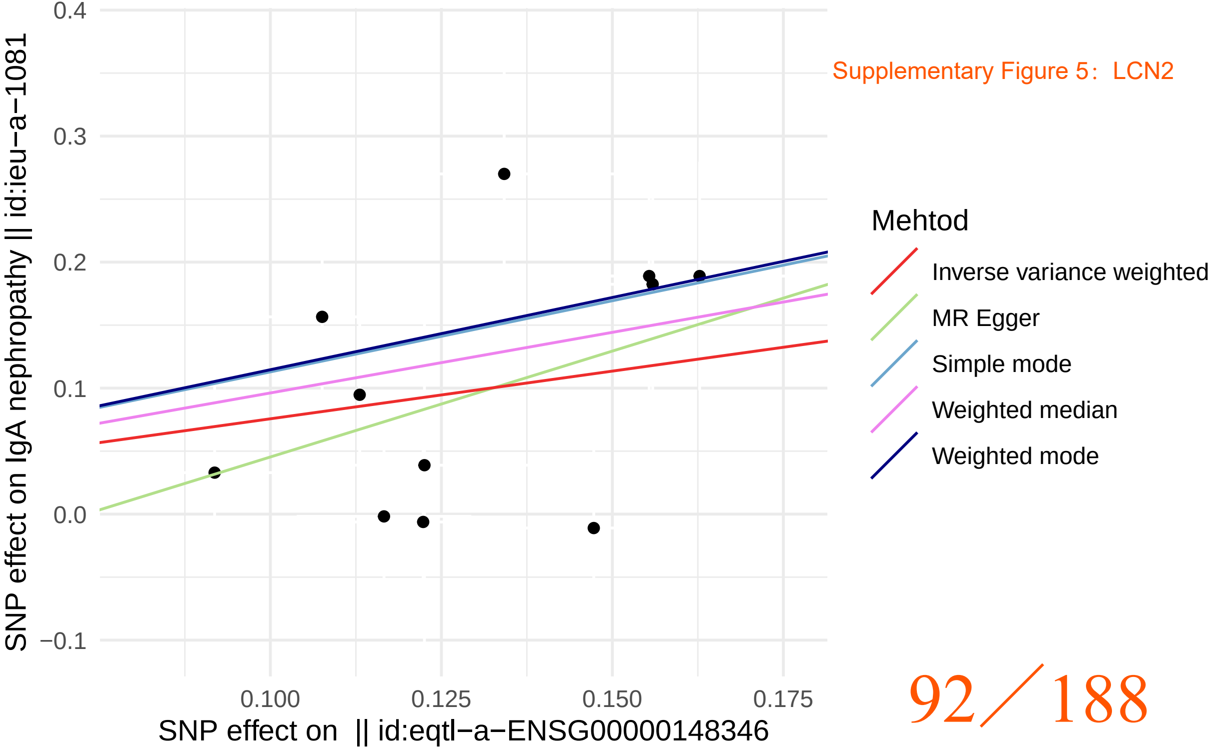

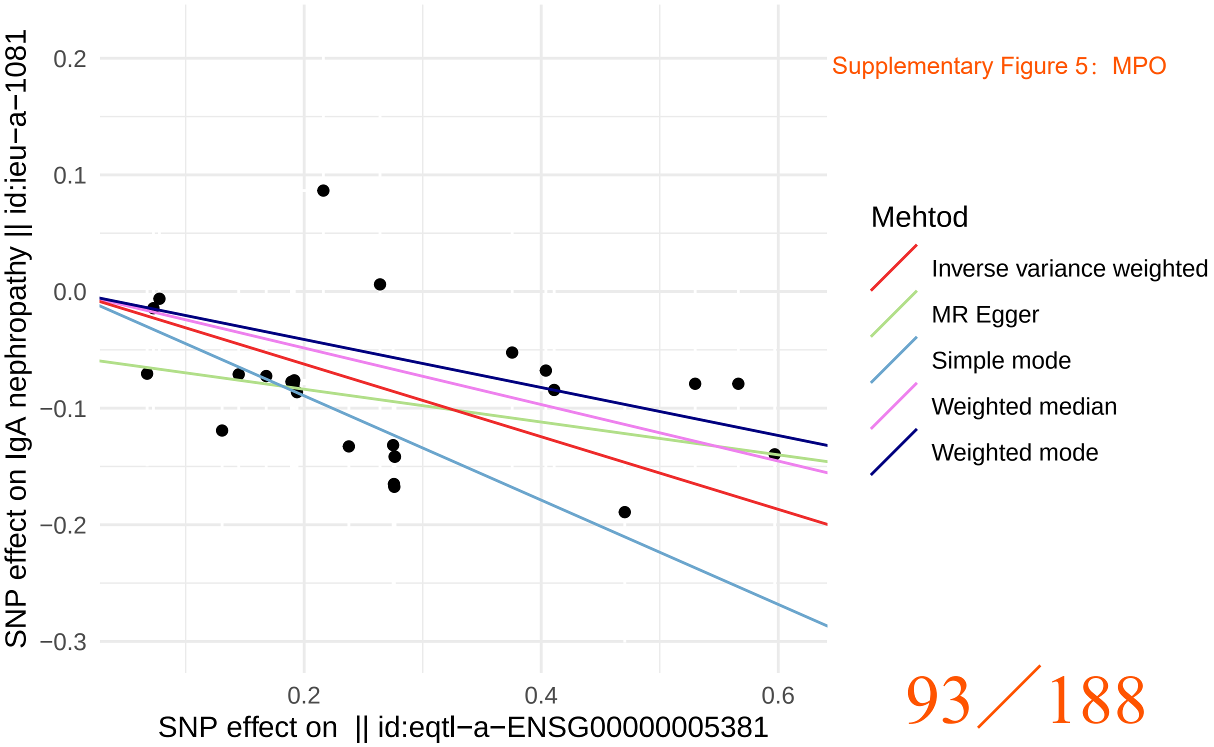

Supplementary Figure 5： MPO

Supplementary Figure 5：SREBF1

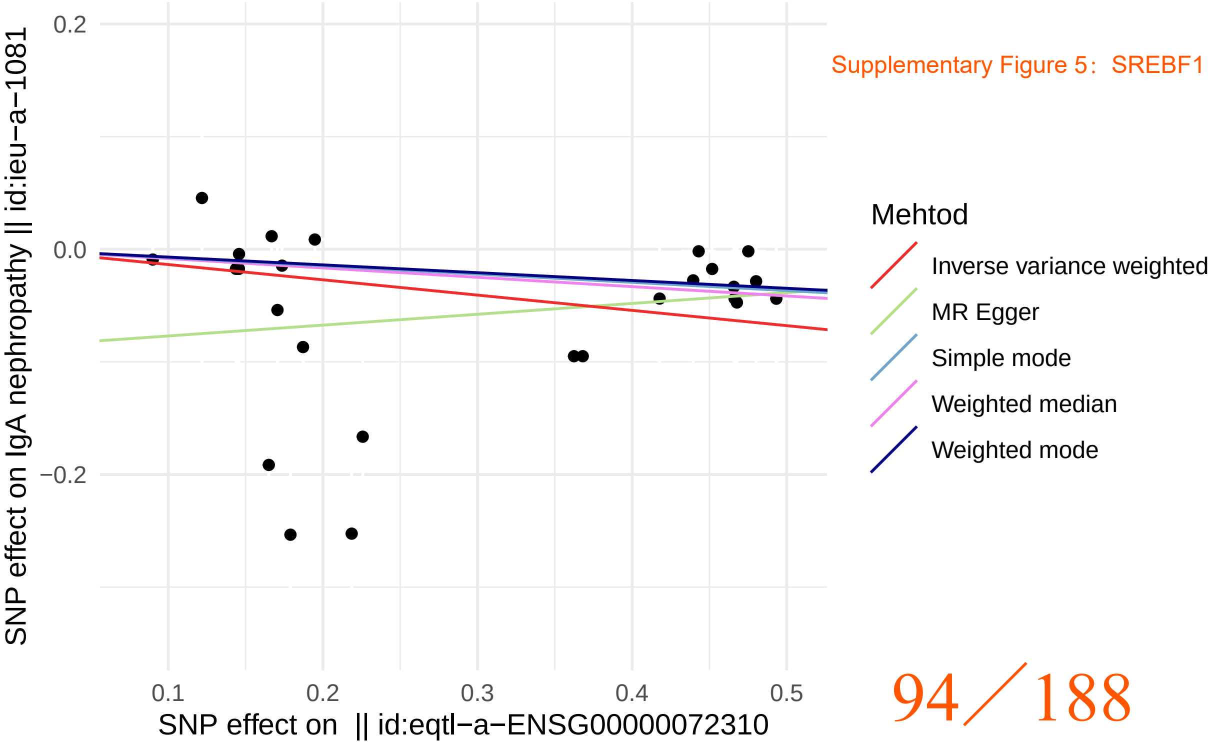

Supplementary Figure 5：TNF

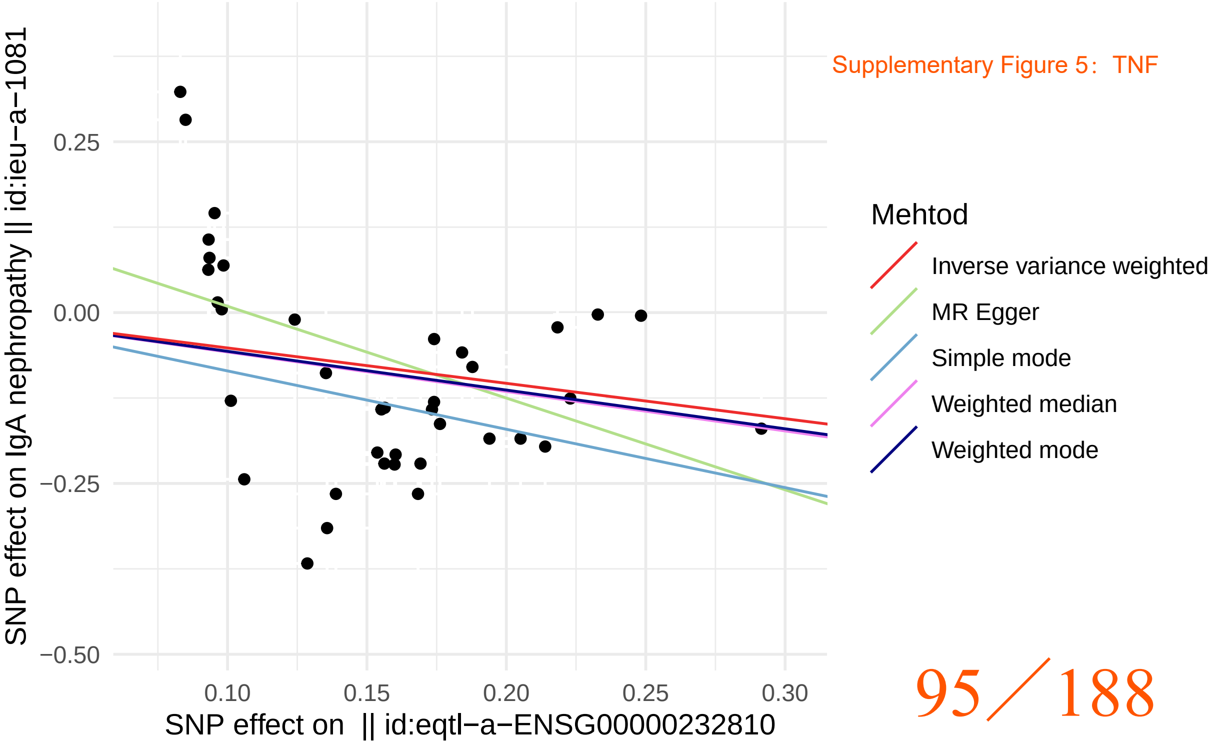

Supplementary Figure 6： AGER

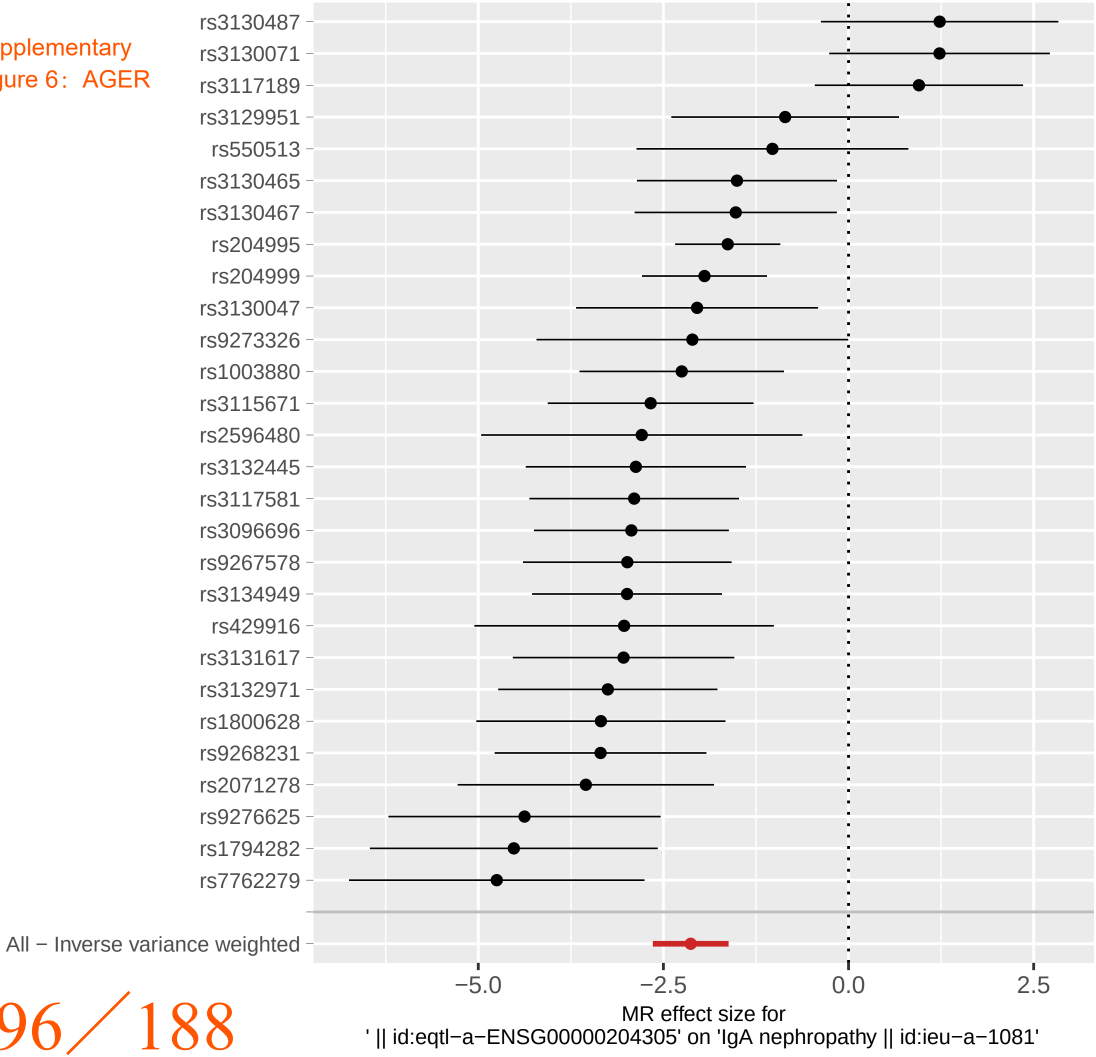

Supplementary Figure 6：ATF6

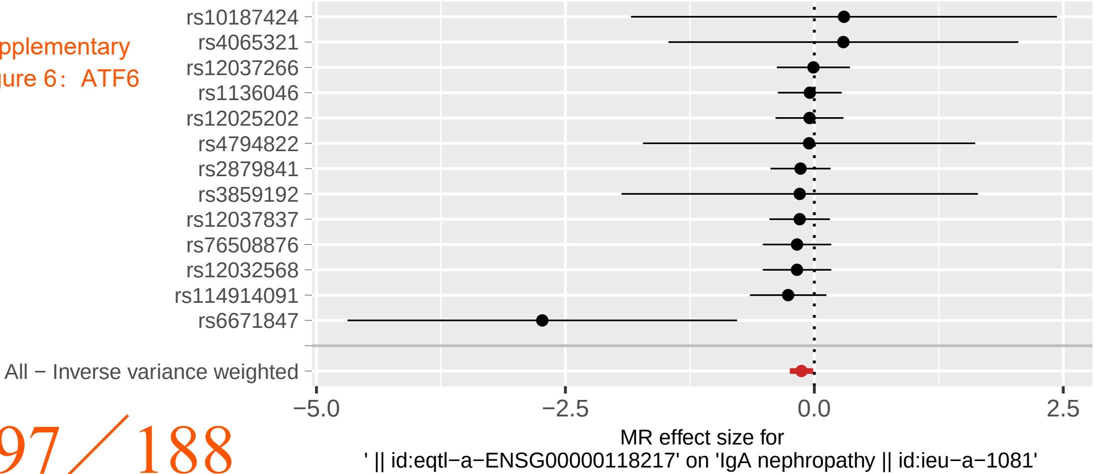

97／188

Supplementary Figure 6： CASP7

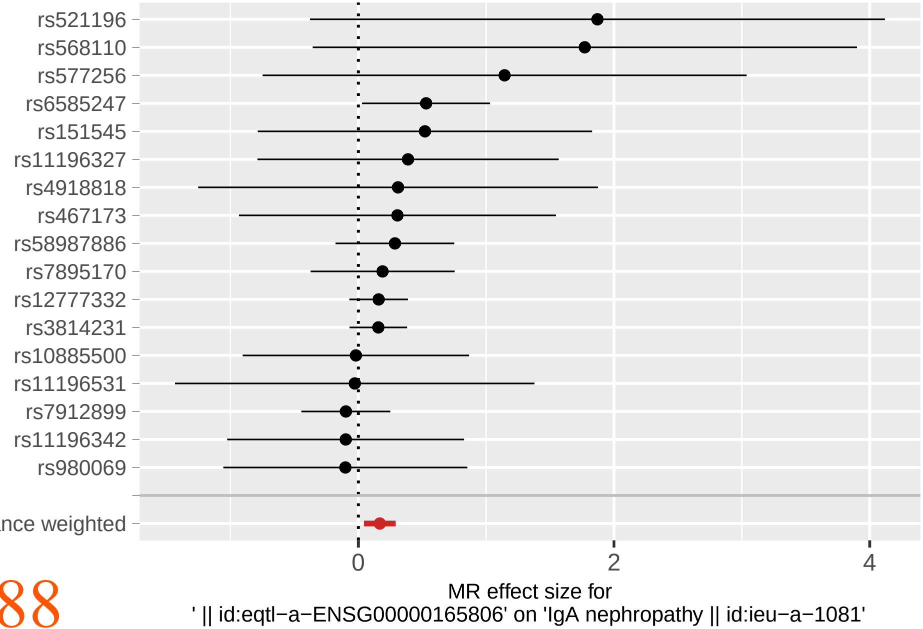

Supplementary Figure 6：CAT

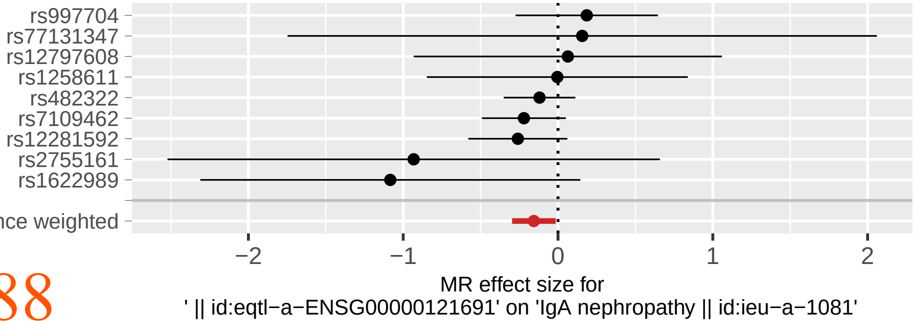

Supplementary Figure 6：CD36

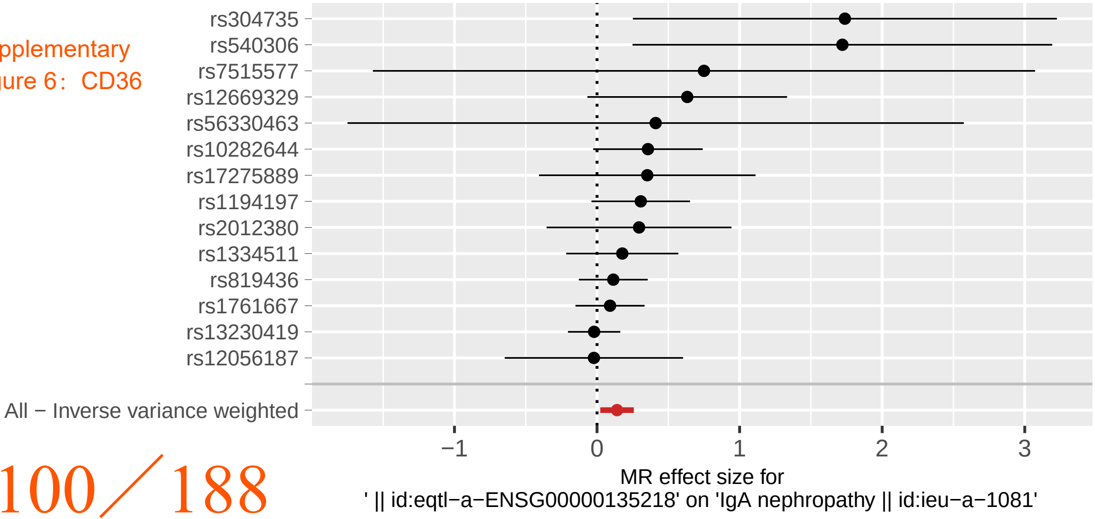

Supplementary Figure 6： ELF2

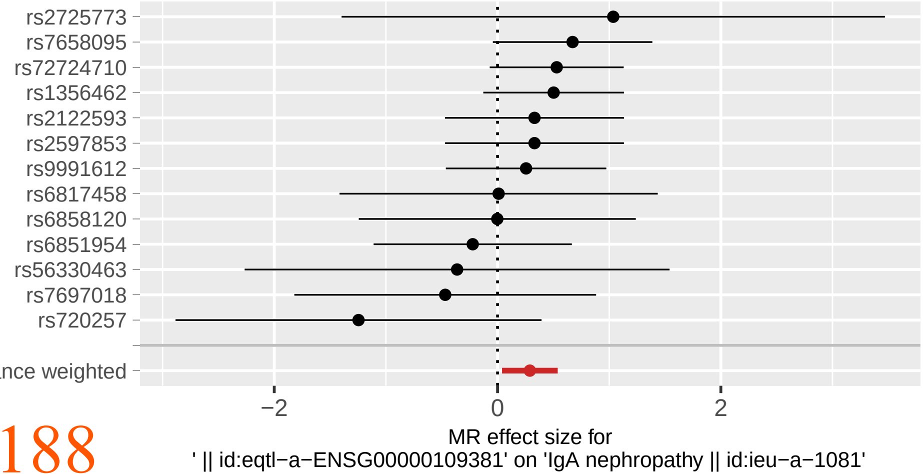

Supplementary Figure 6：JAK2

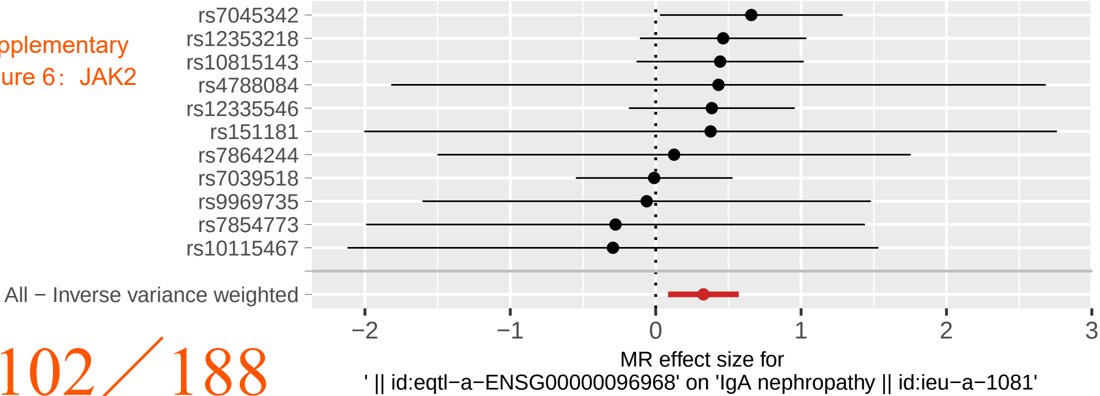

Supplementary Figure 6：LCN2

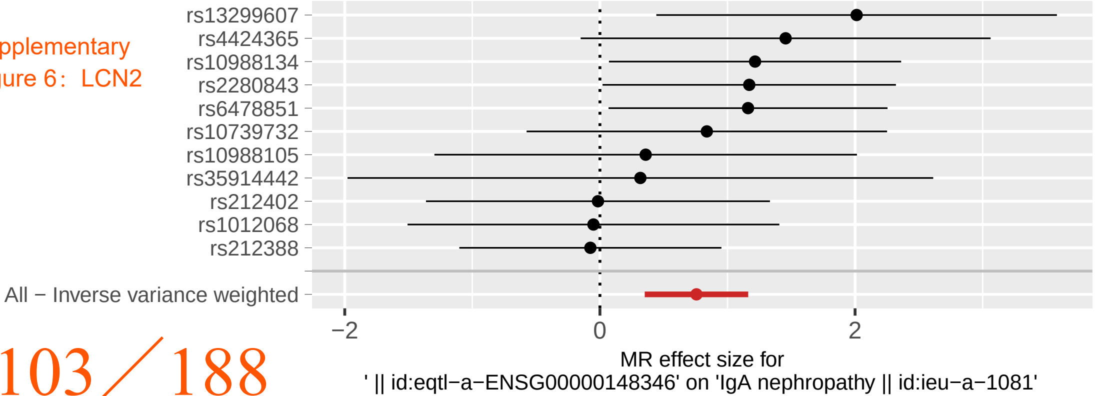

Supplementary Figure 6：MPO

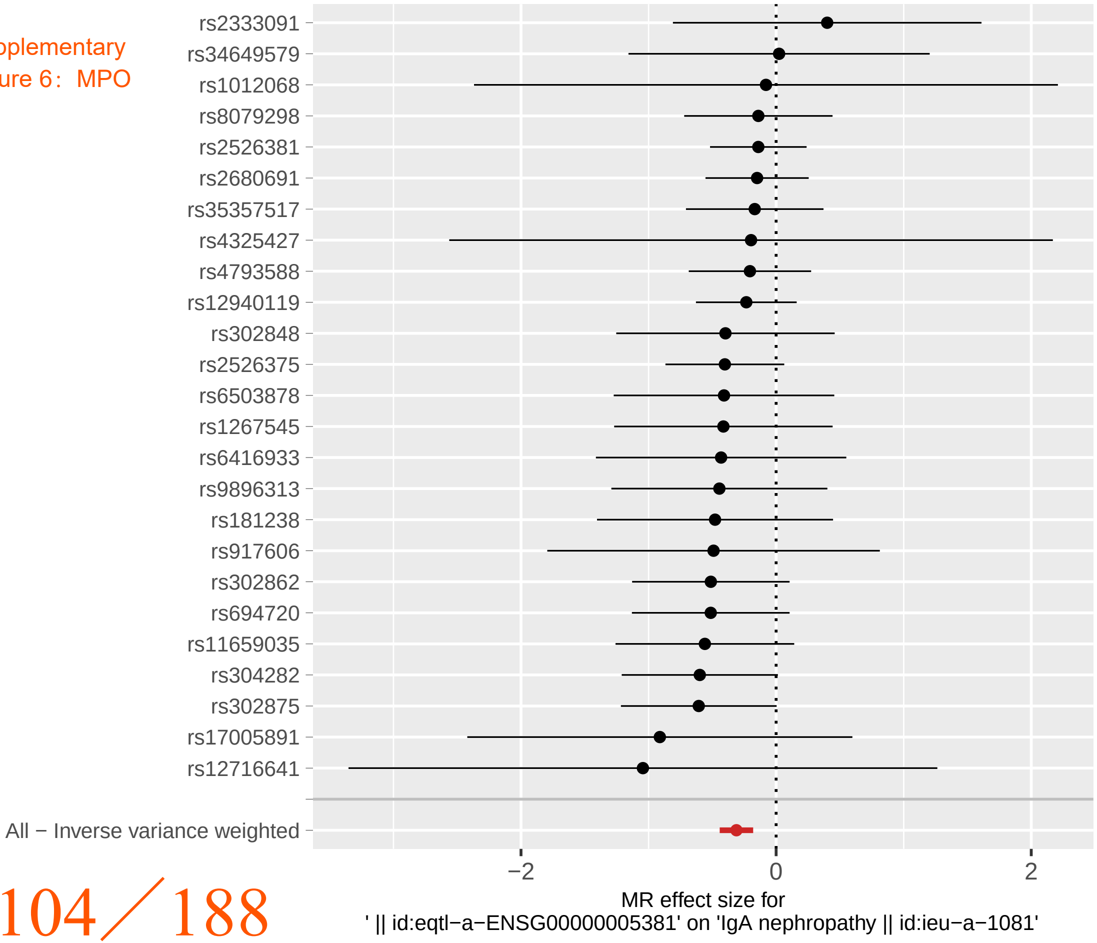

Supplementary Figure 6： SREBF1

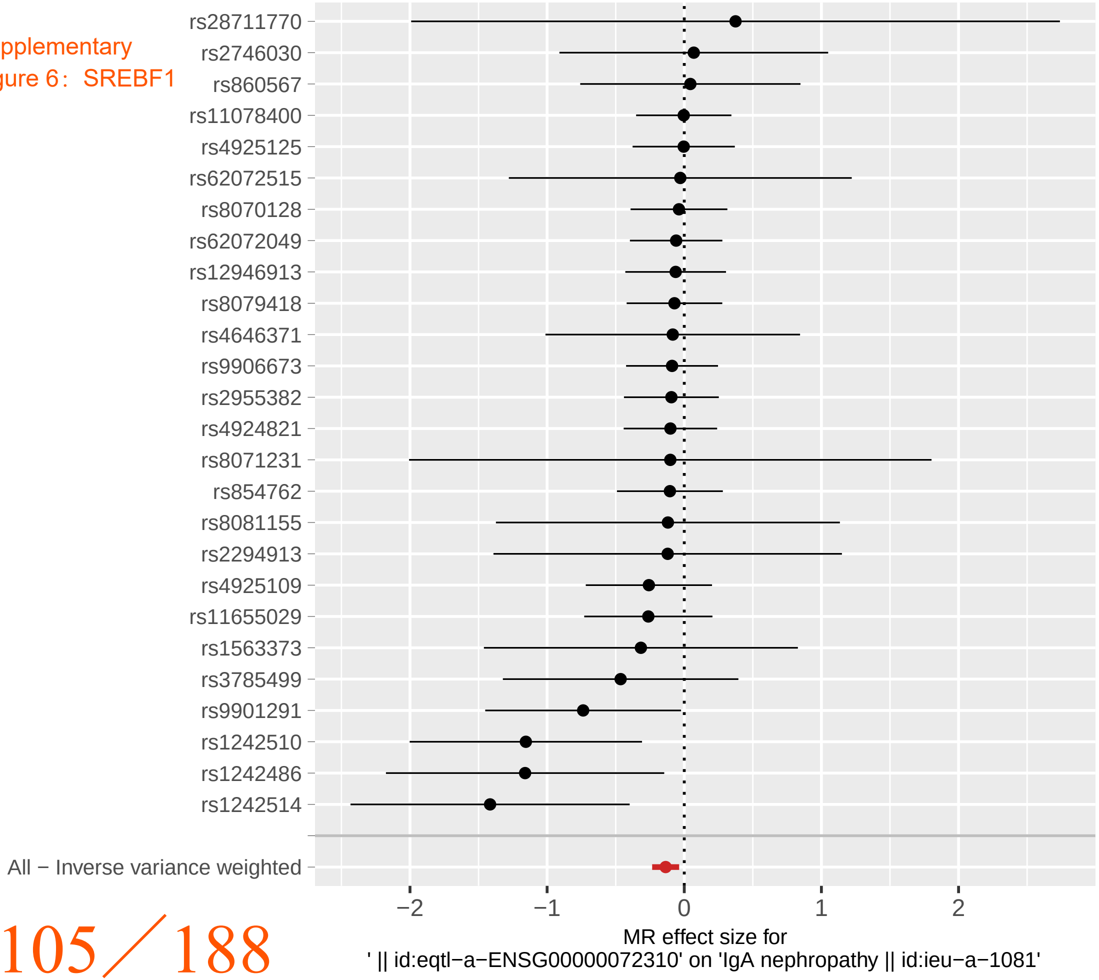

Supplementary Figure 6：TNF

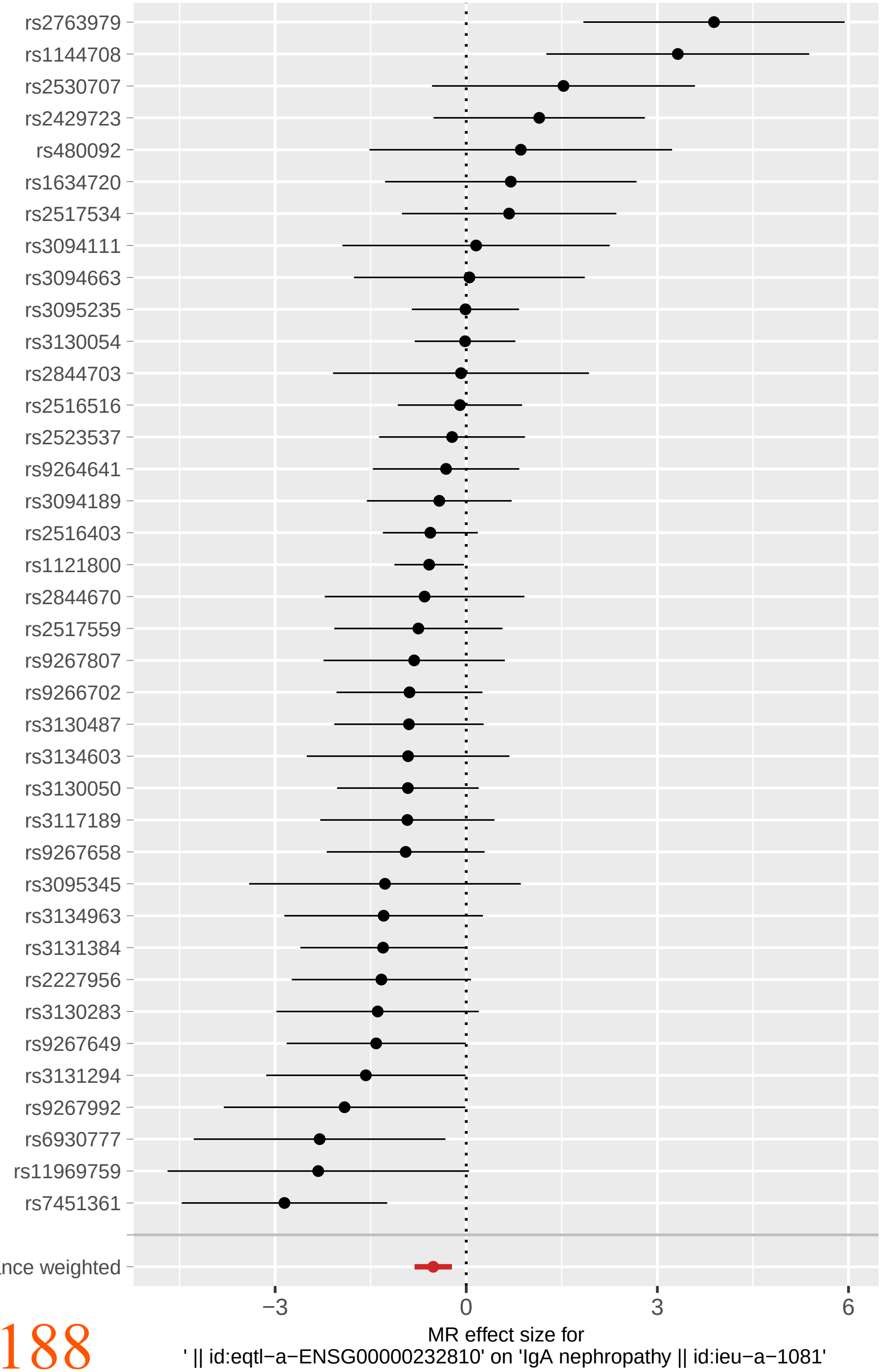

Supplementary
Figure 7： AGER

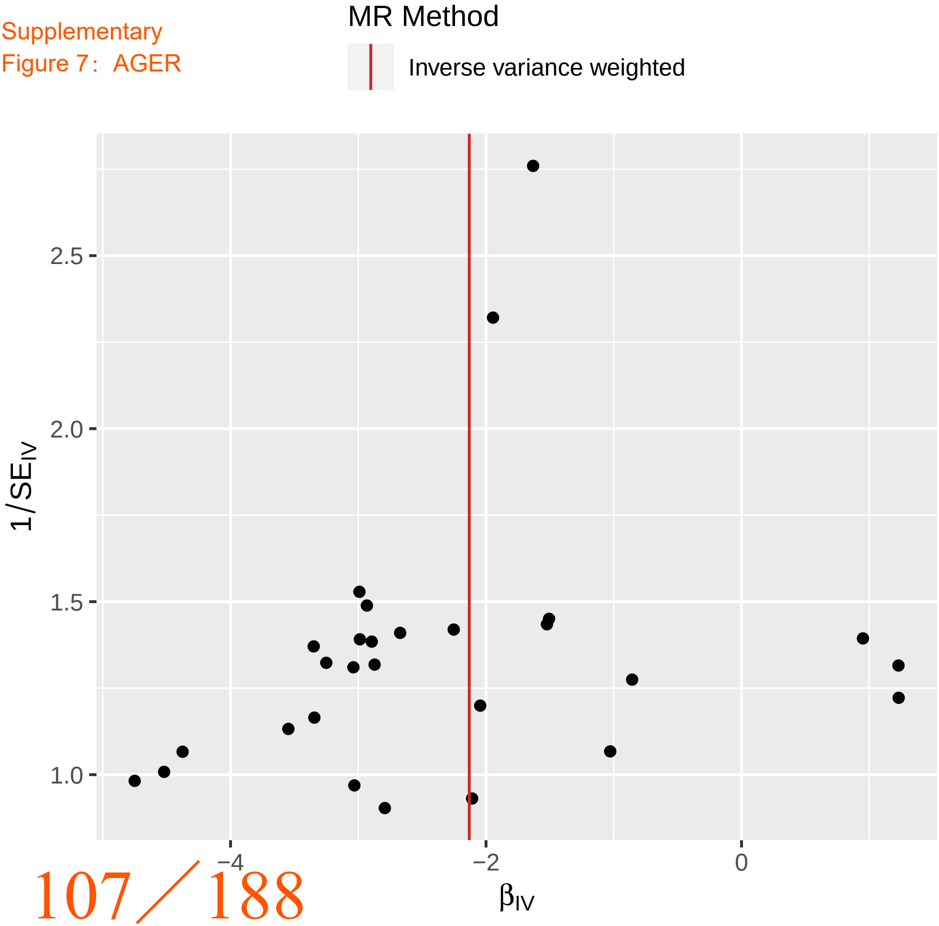

Supplementary Figure 7： ATF6

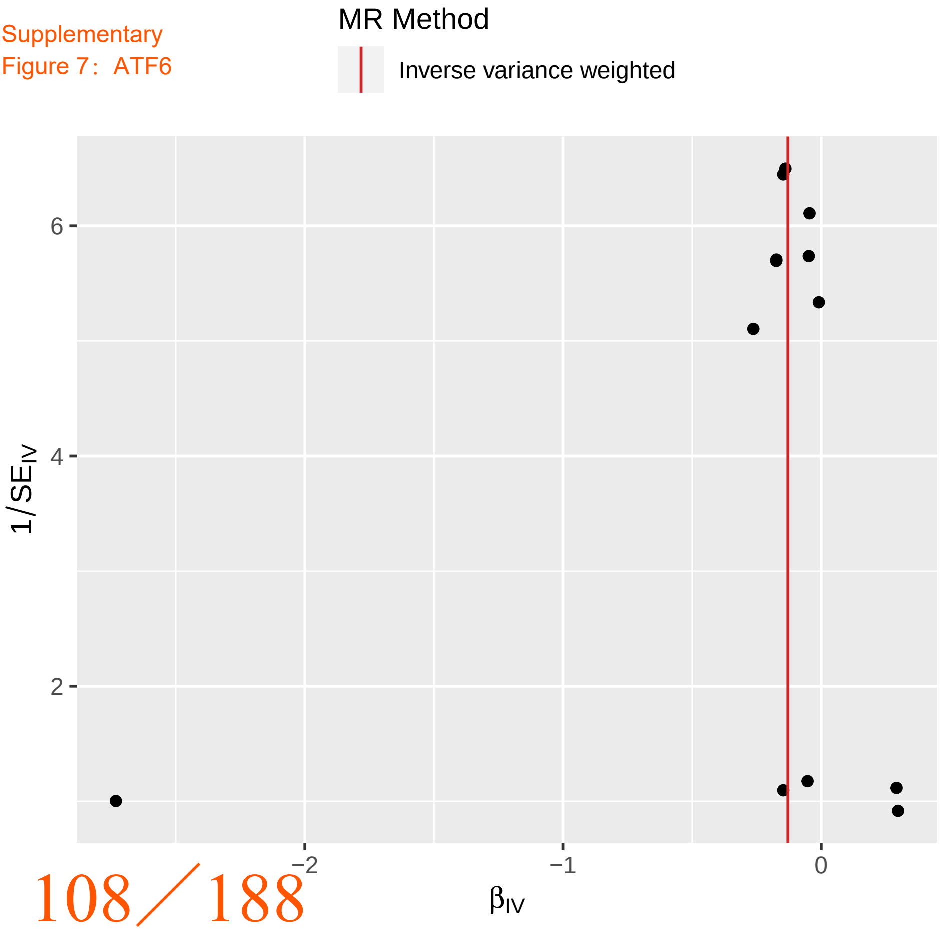

Supplementary
Figure 7：CASP7

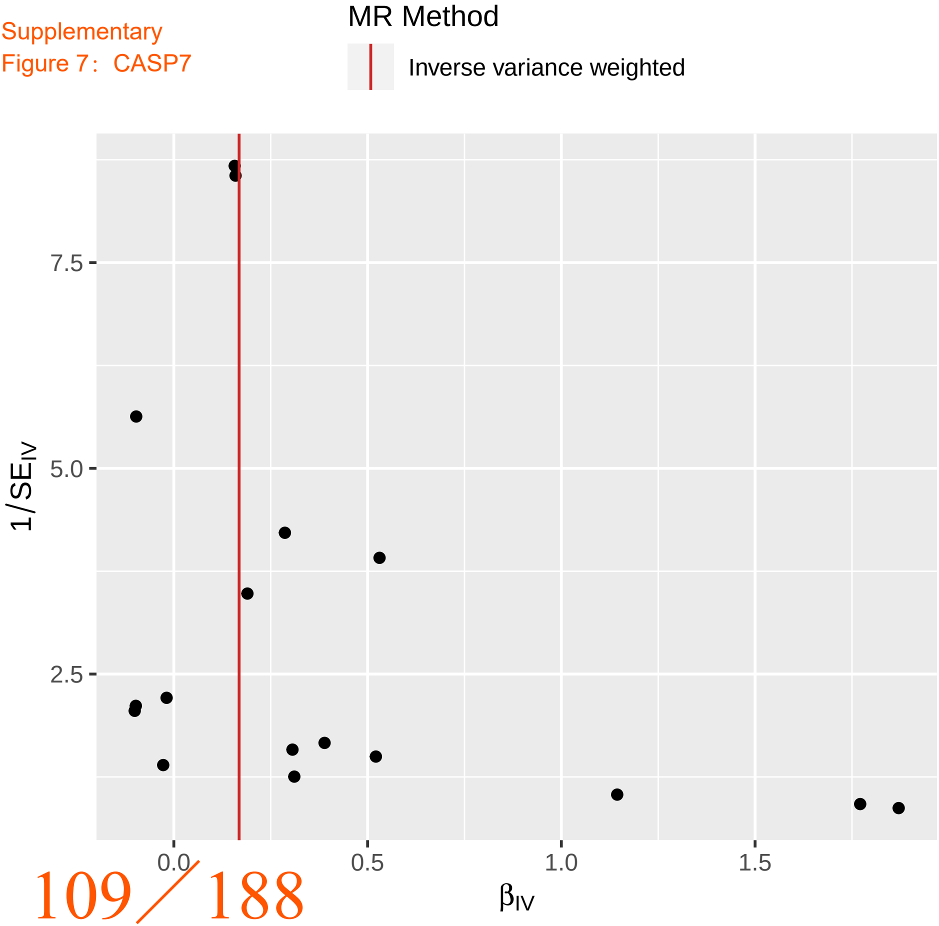

Supplementary Figure 7：CAT

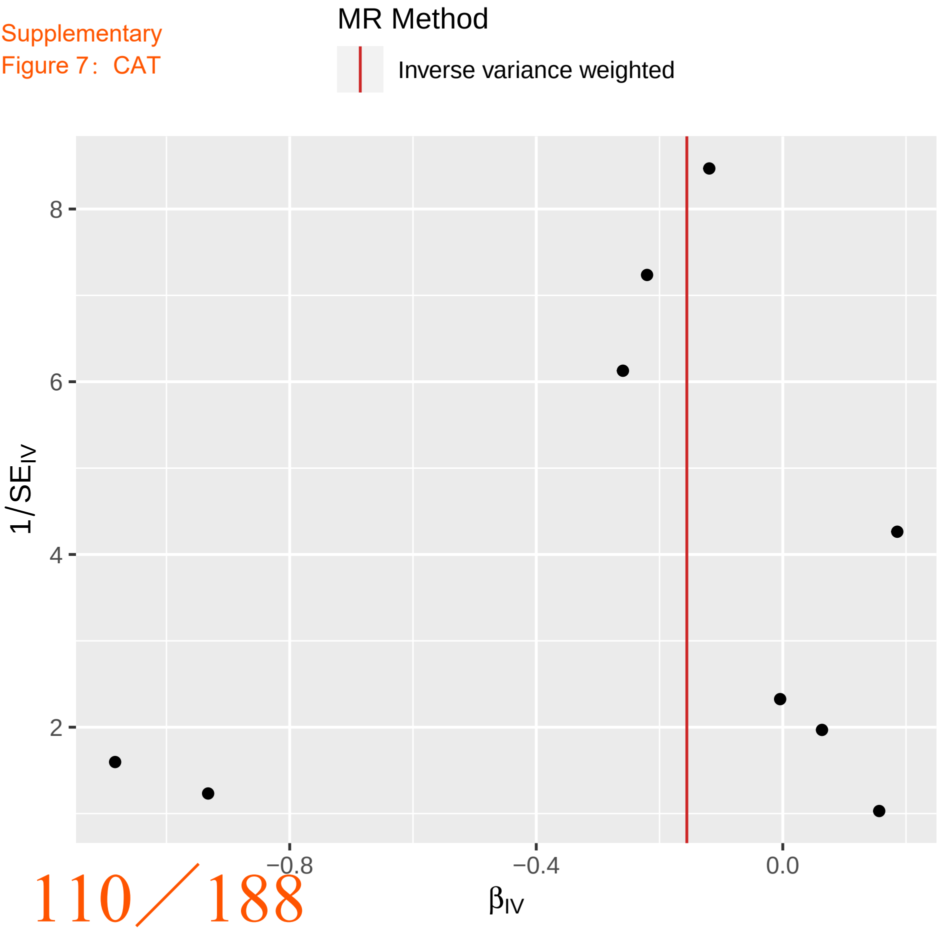

Supplementary Figure 7： CD36

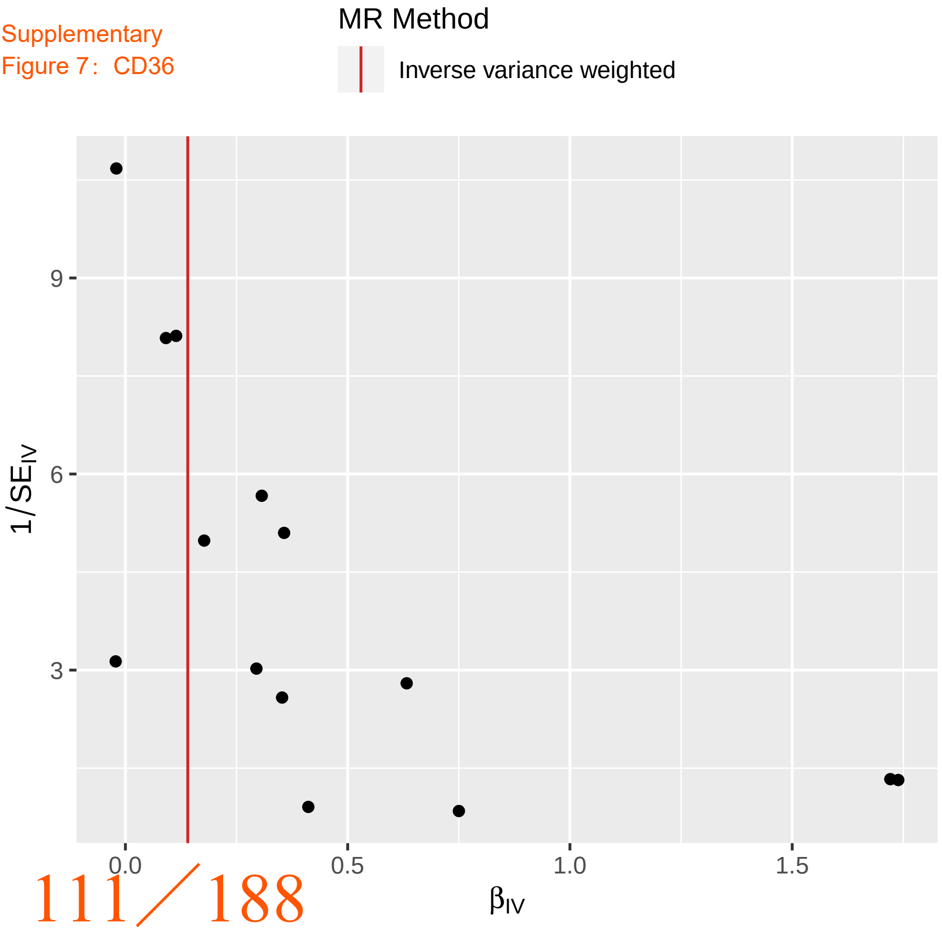

Supplementary Figure 7： ELF2

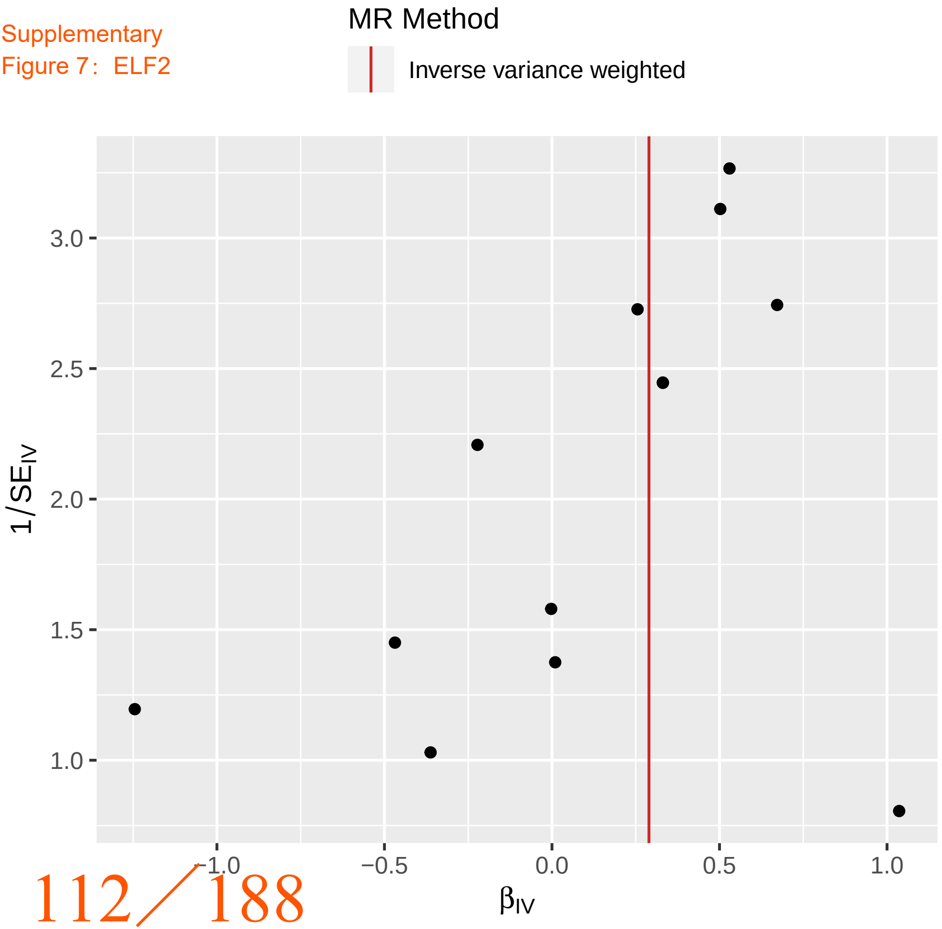

Supplementary
Figure 7：JAK2

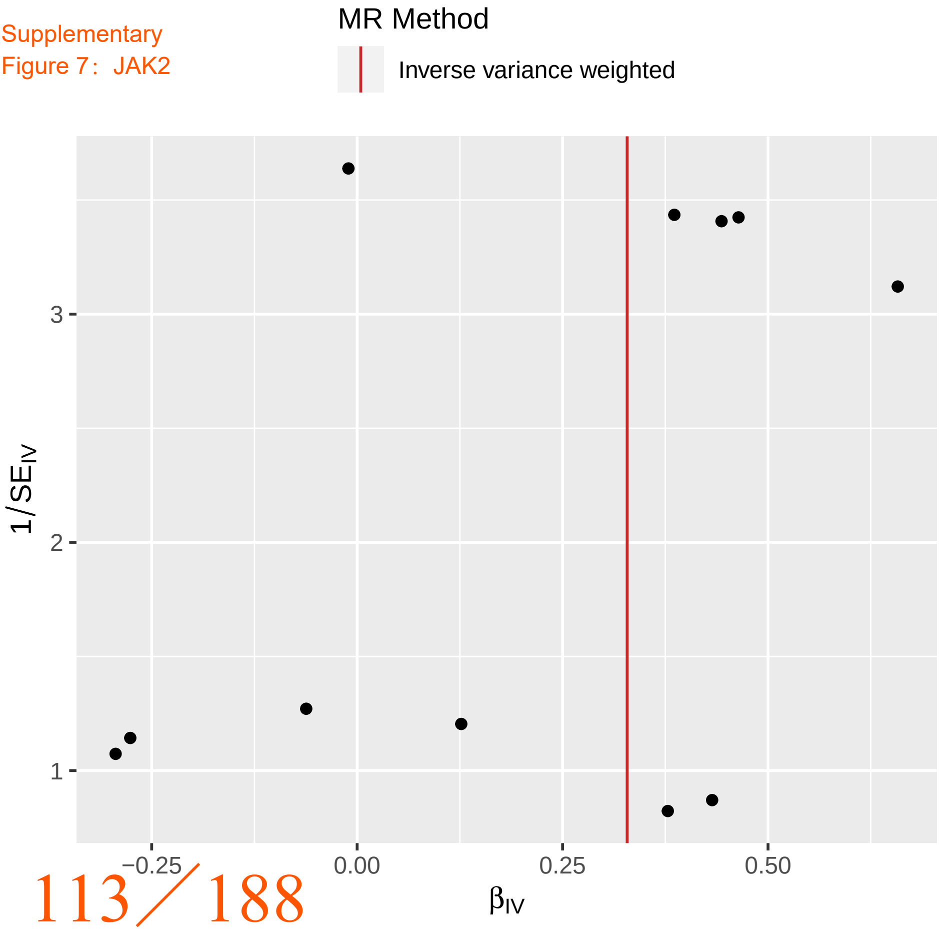

Supplementary
Figure 7： LCN2

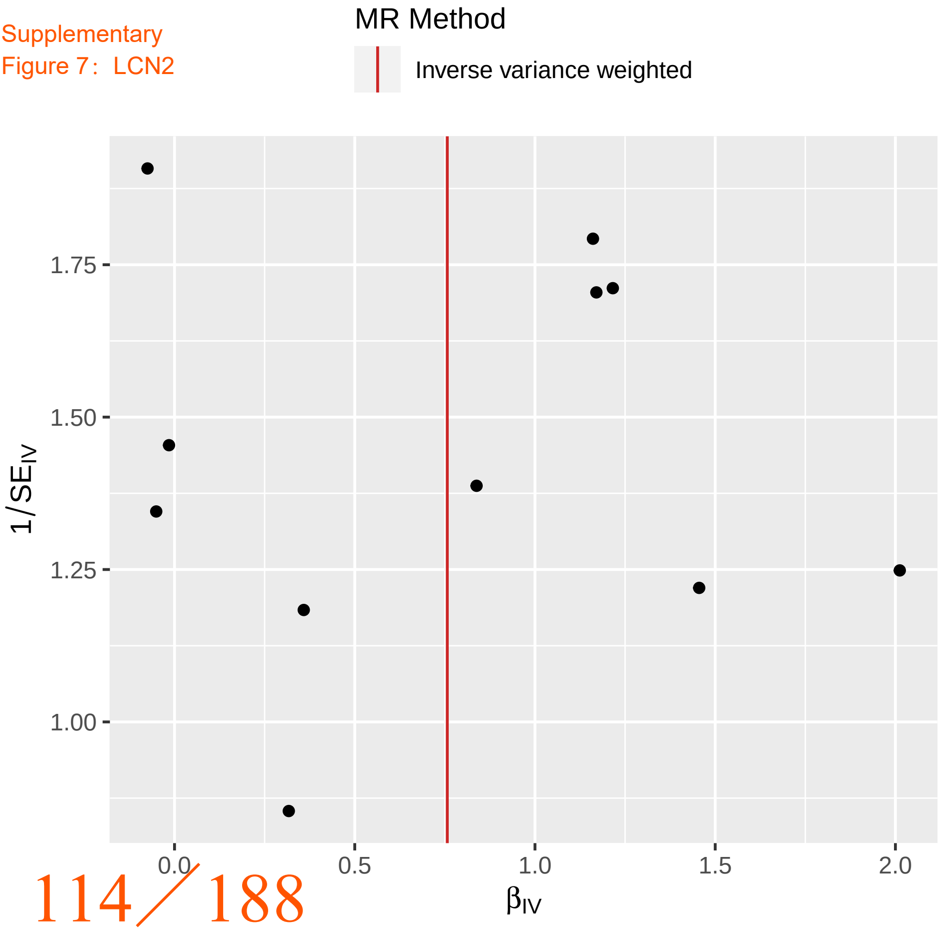

Supplementary
Figure 7： MPO

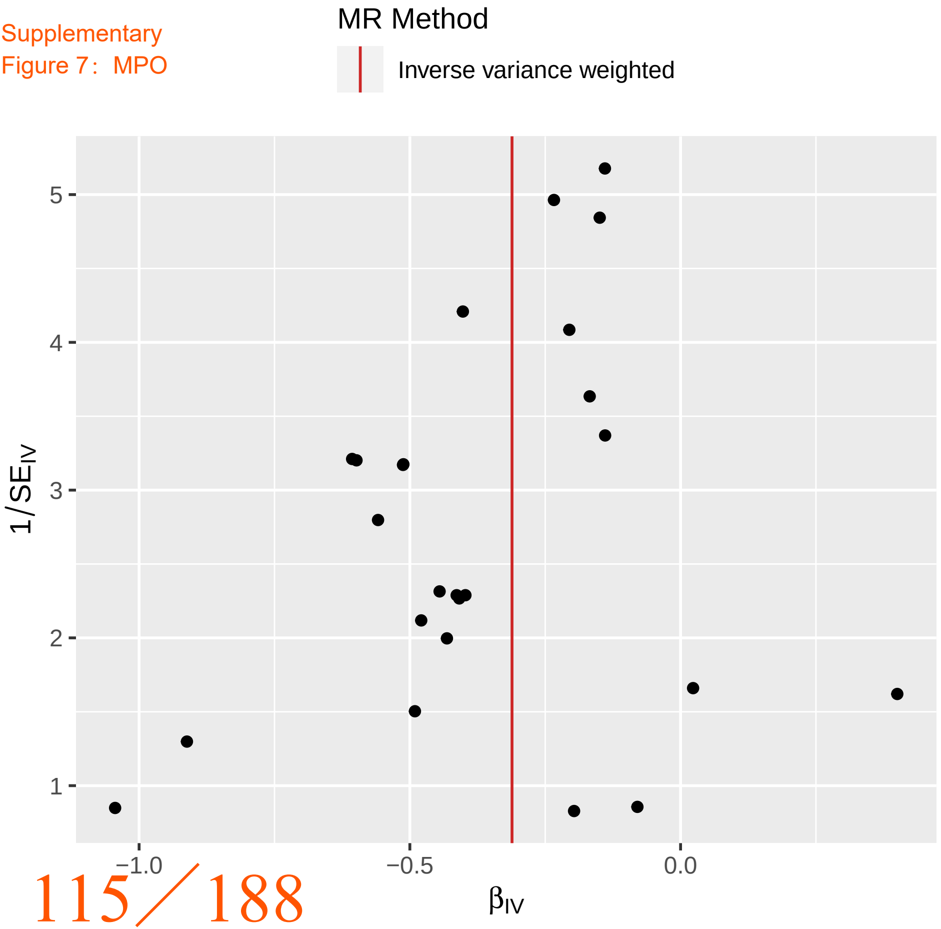

Supplementary Figure 7： SREBF1

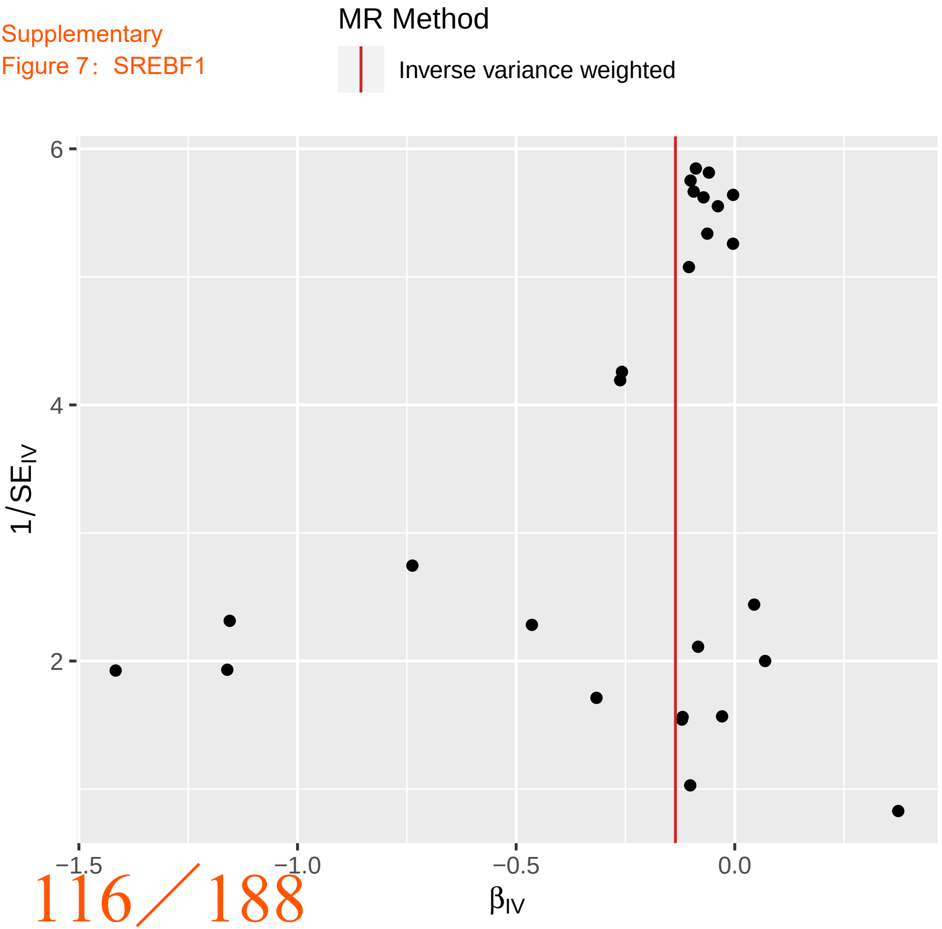

Supplementary
Figure 7：TNF

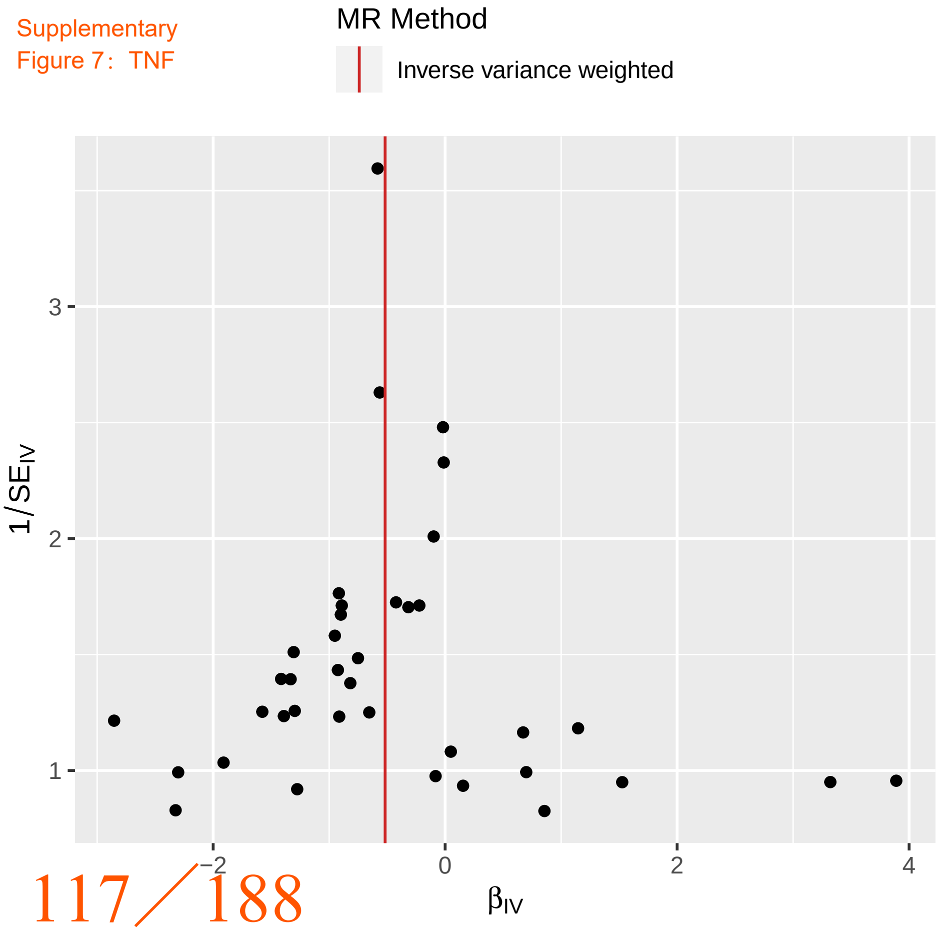

Supplementary Figure 8：AGER

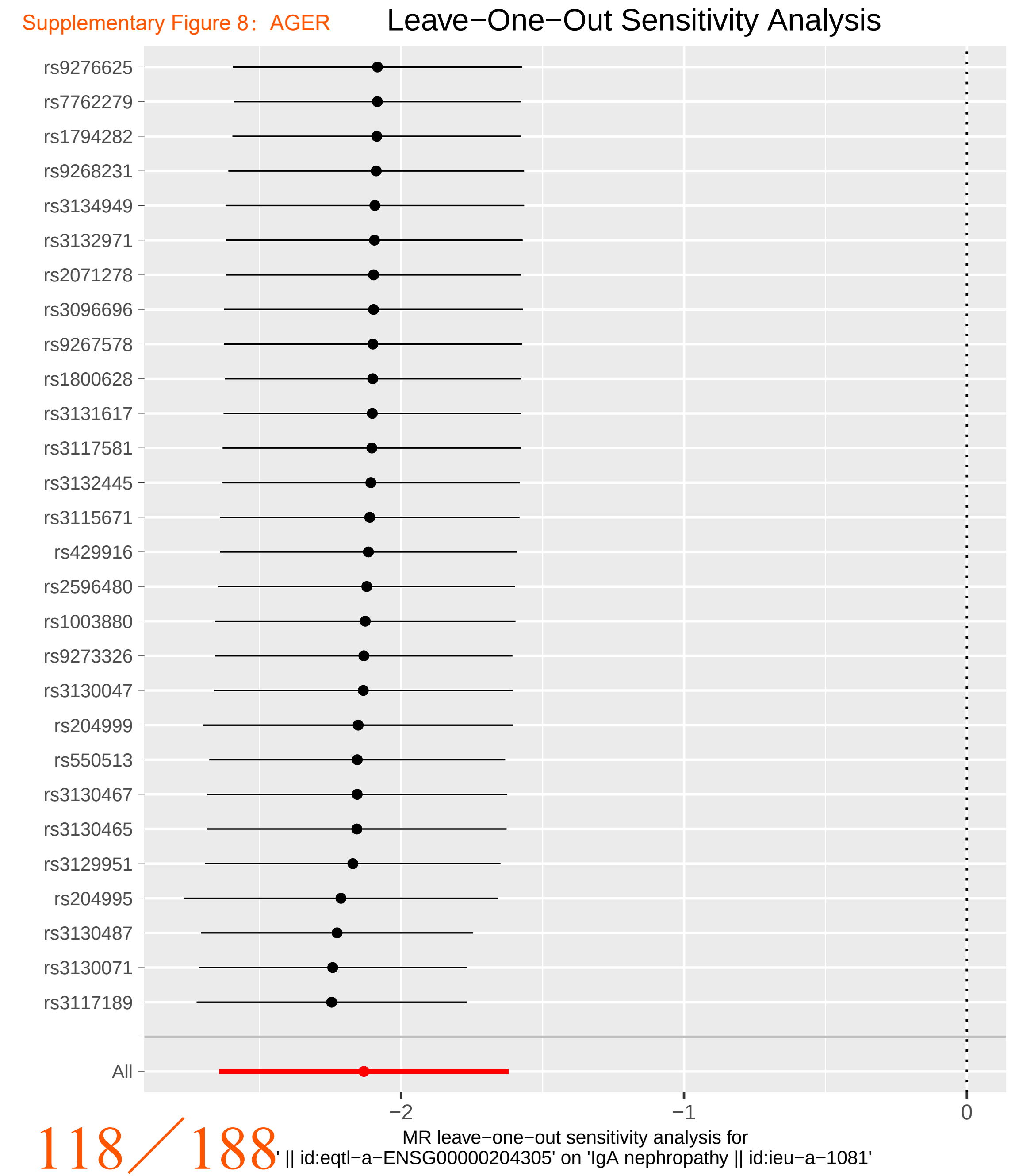

Supplementary Figure 8: ATF6

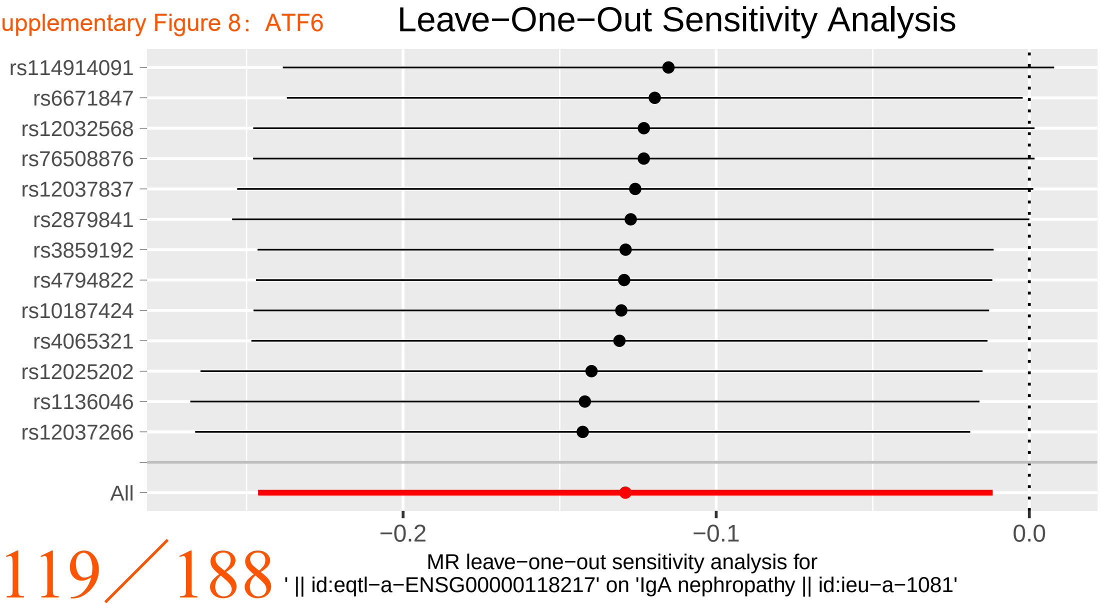

Supplementary Figure 8：CASP7

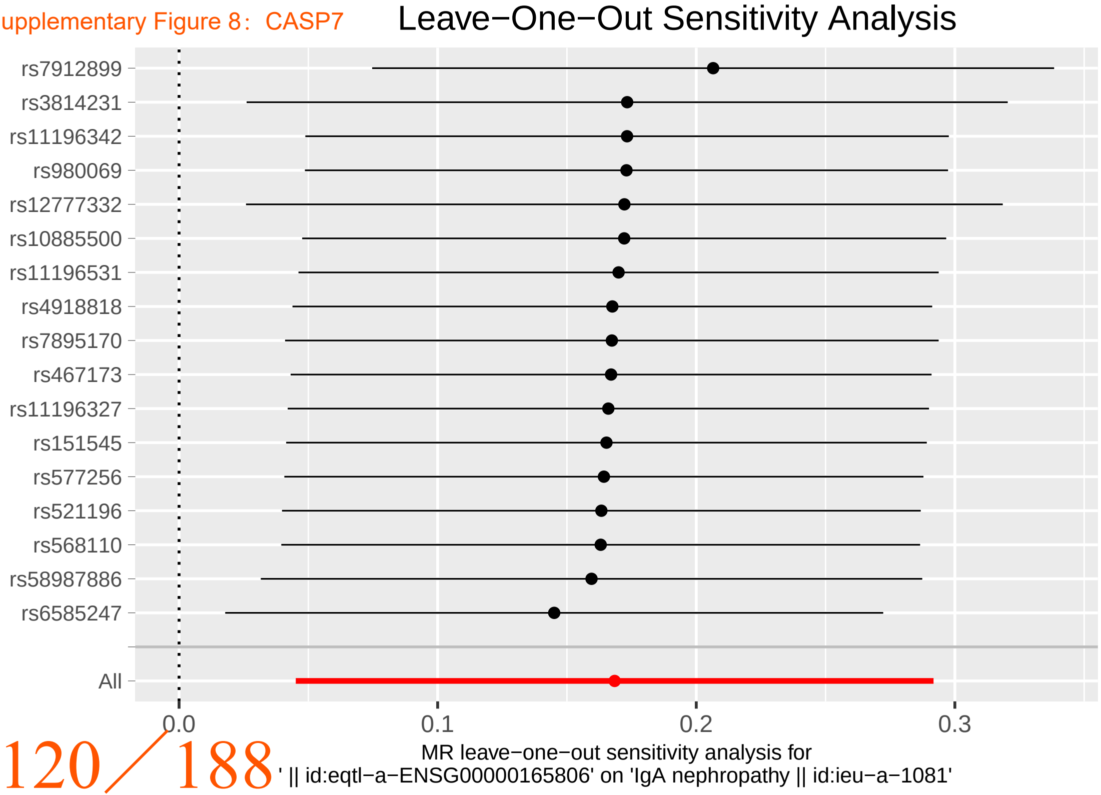

Supplementary Figure 8：CAT

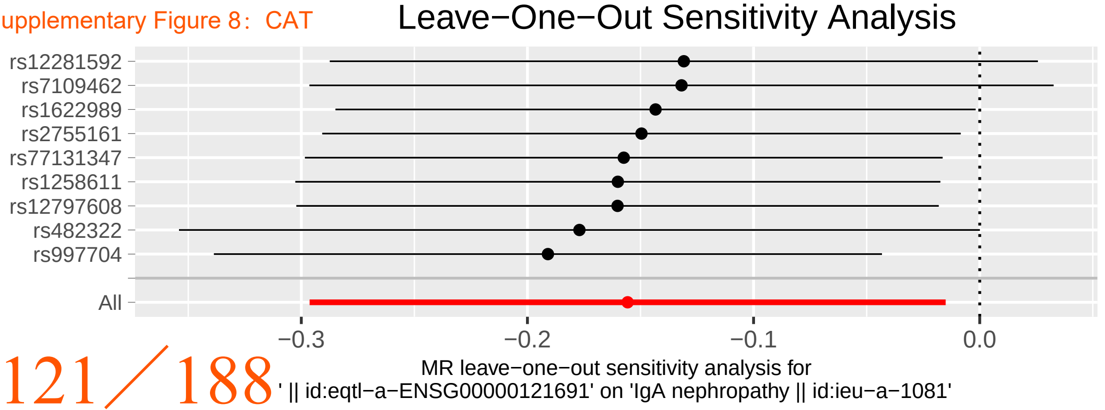

Supplementary Figure 8：CD36

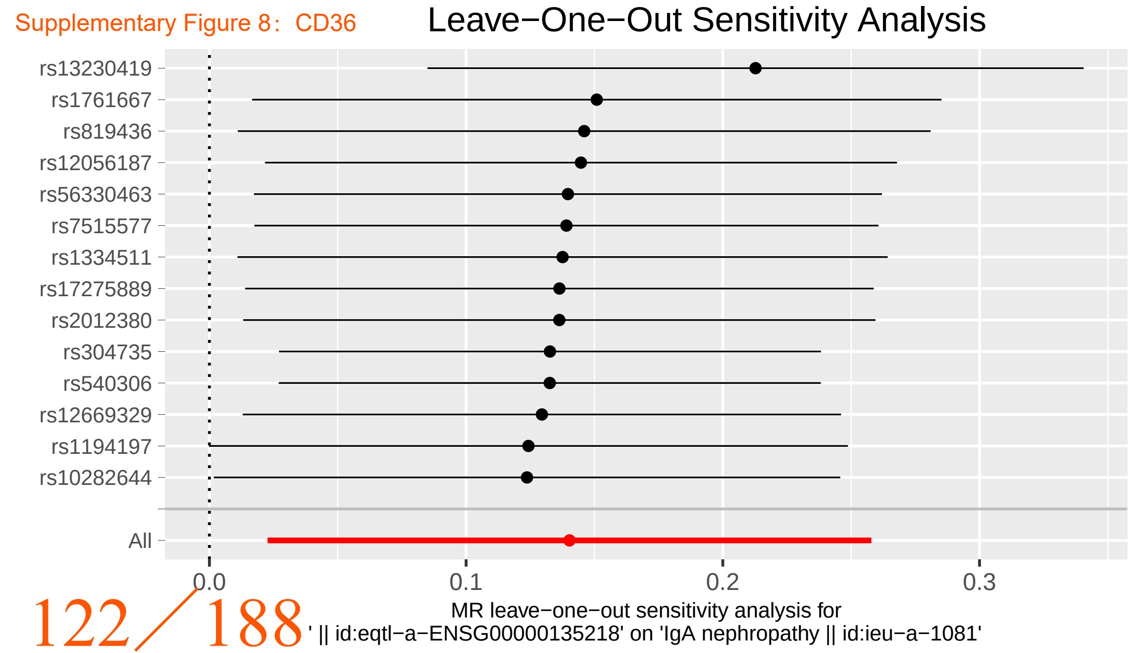

Supplementary Figure 8： ELF2

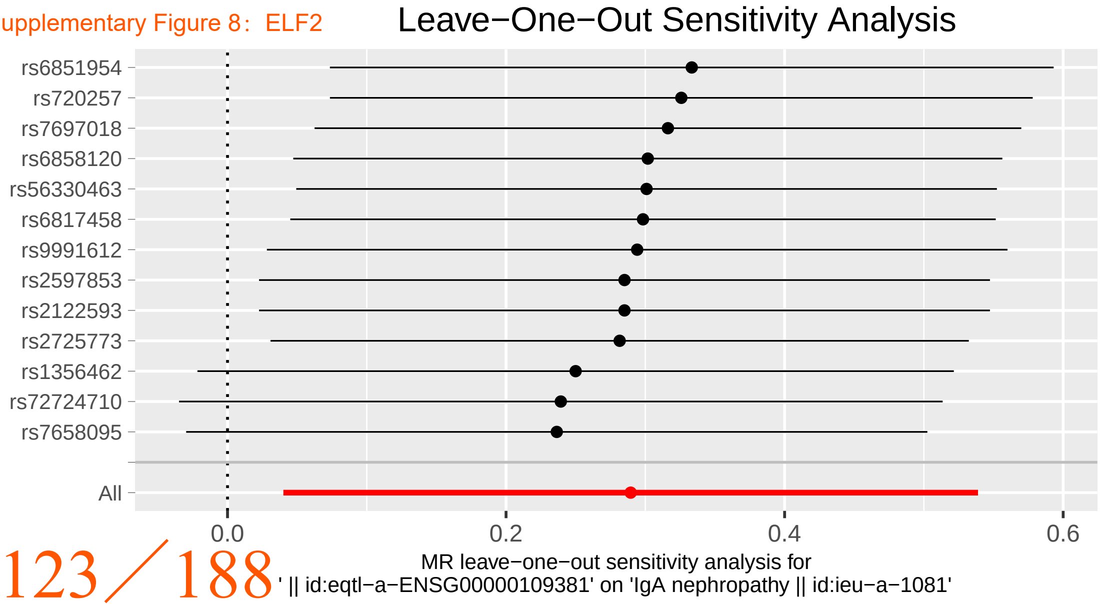

Supplementary Figure 8： JAK2

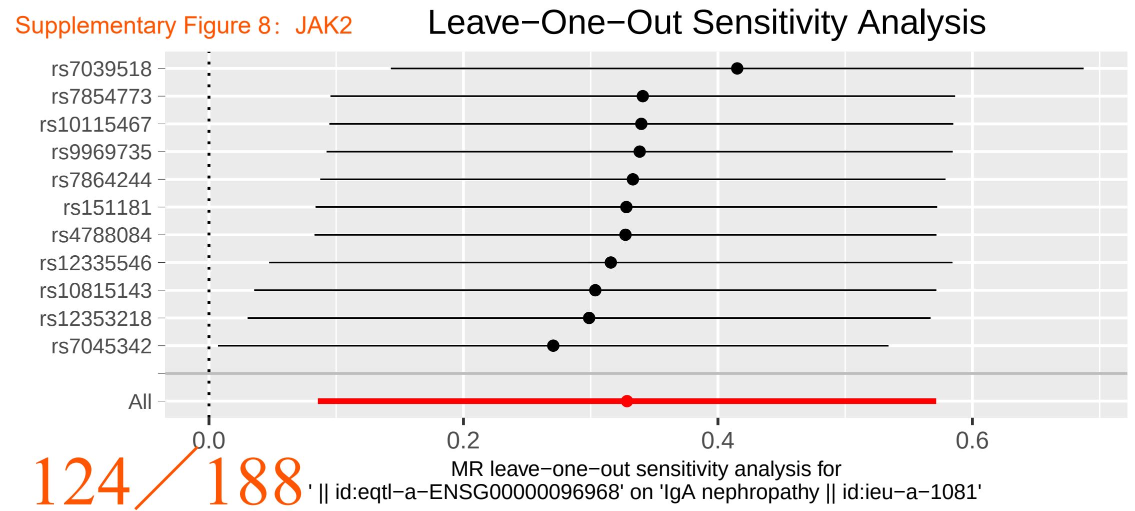

Supplementary Figure 8： LCN2

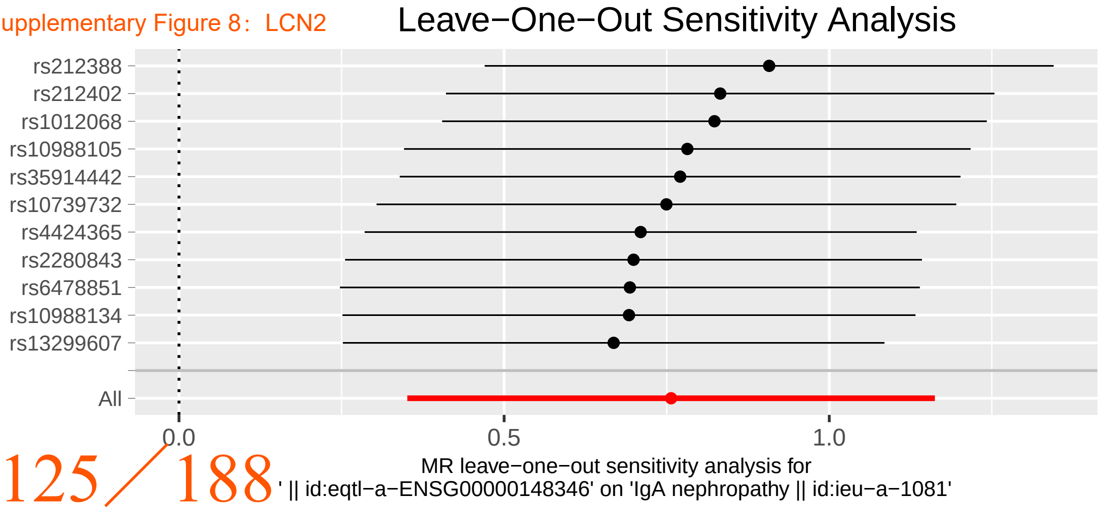

125／188

Supplementary Figure 8：MPO

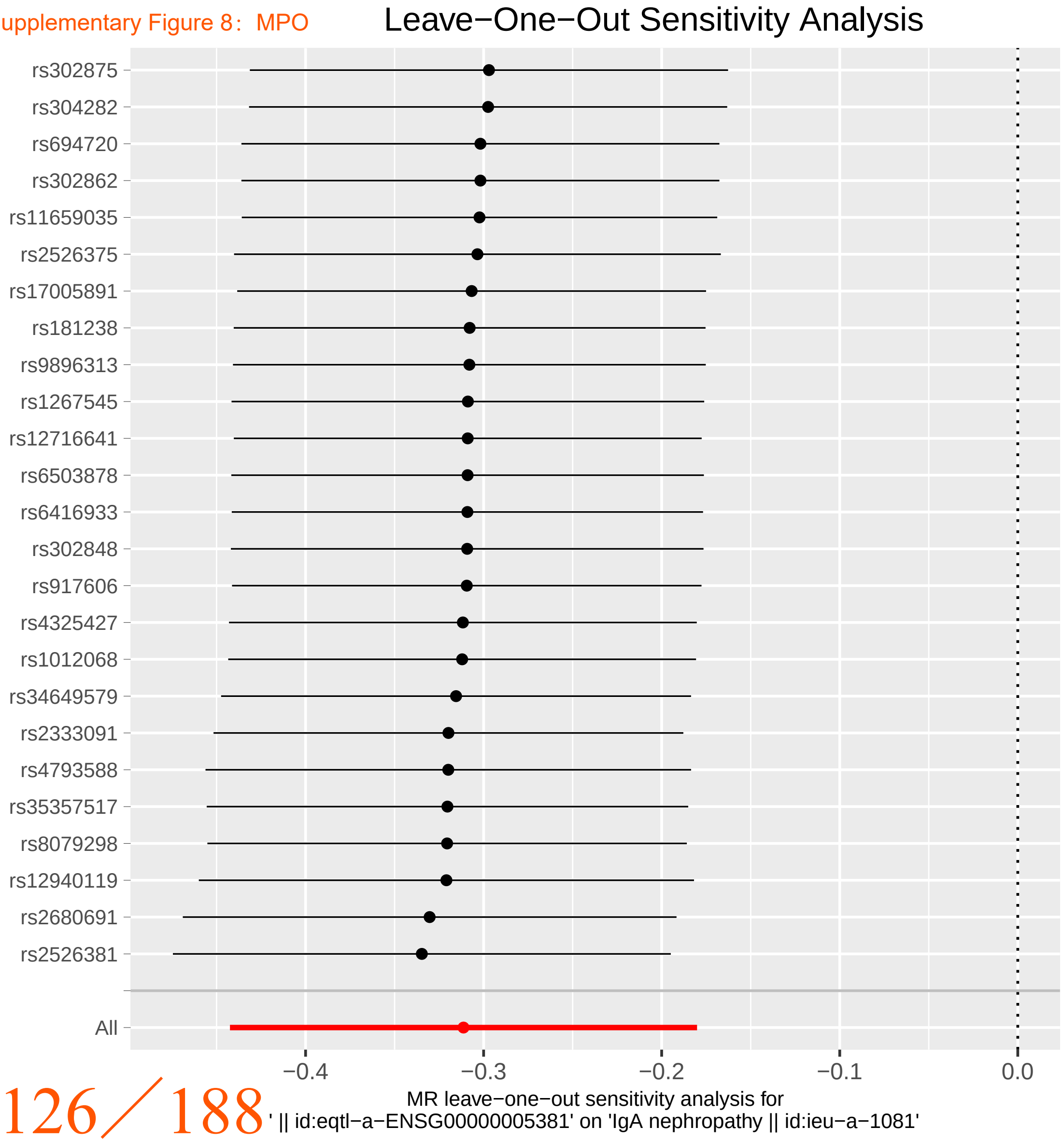

Supplementary Figure 8：SREBF1

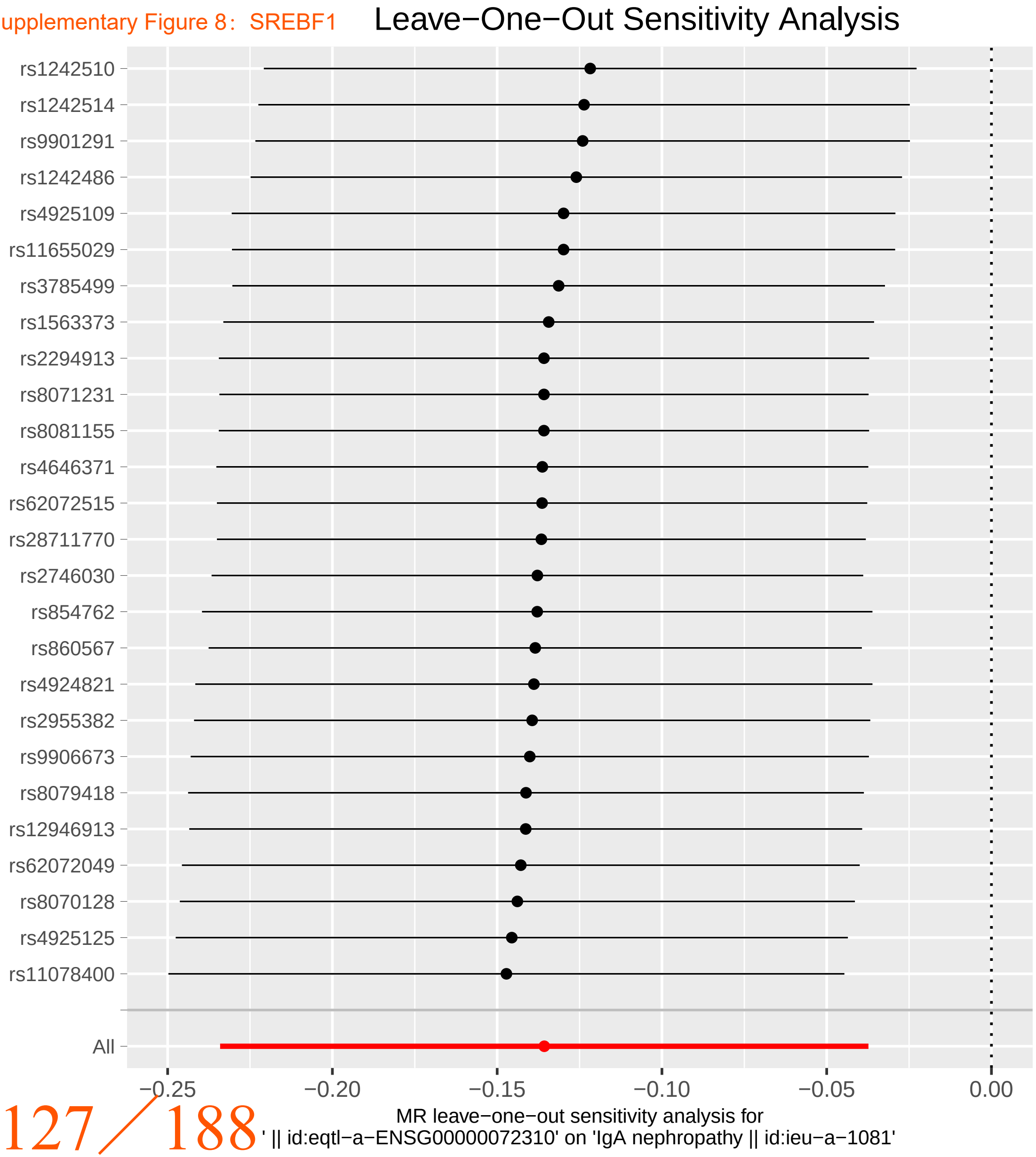

Supplementary Figure 8：TNF

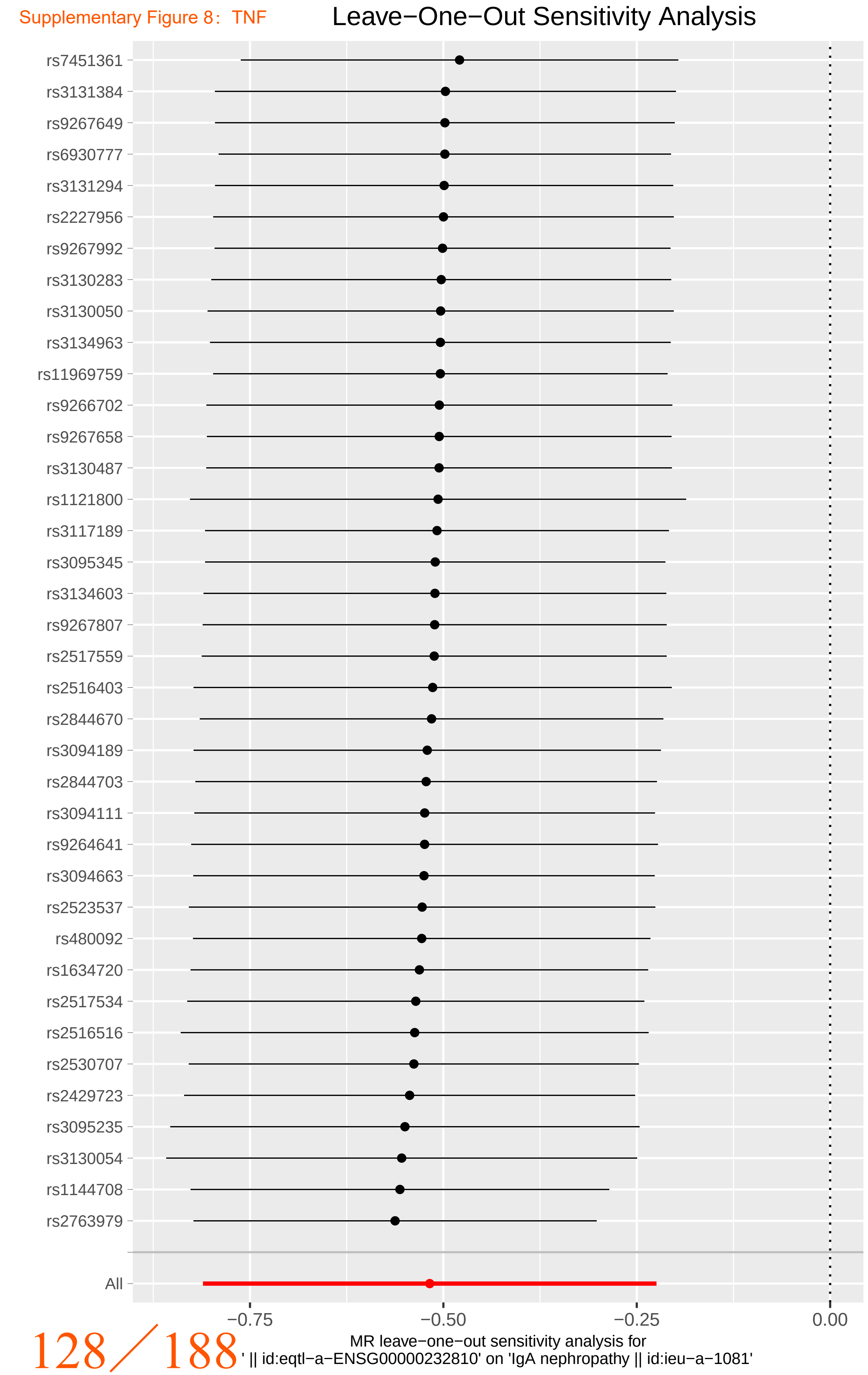

Supplementary Figure 9:
Dementia with Lewy bodies

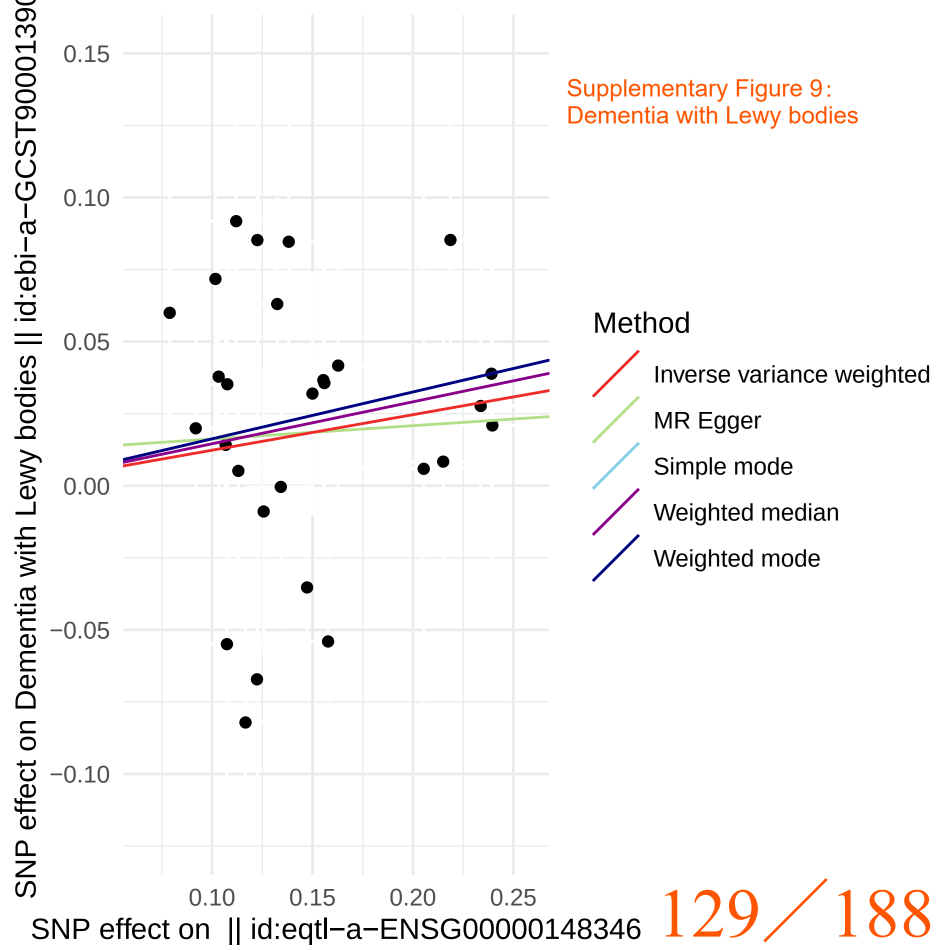

Supplementary Figure 9: Hypertension

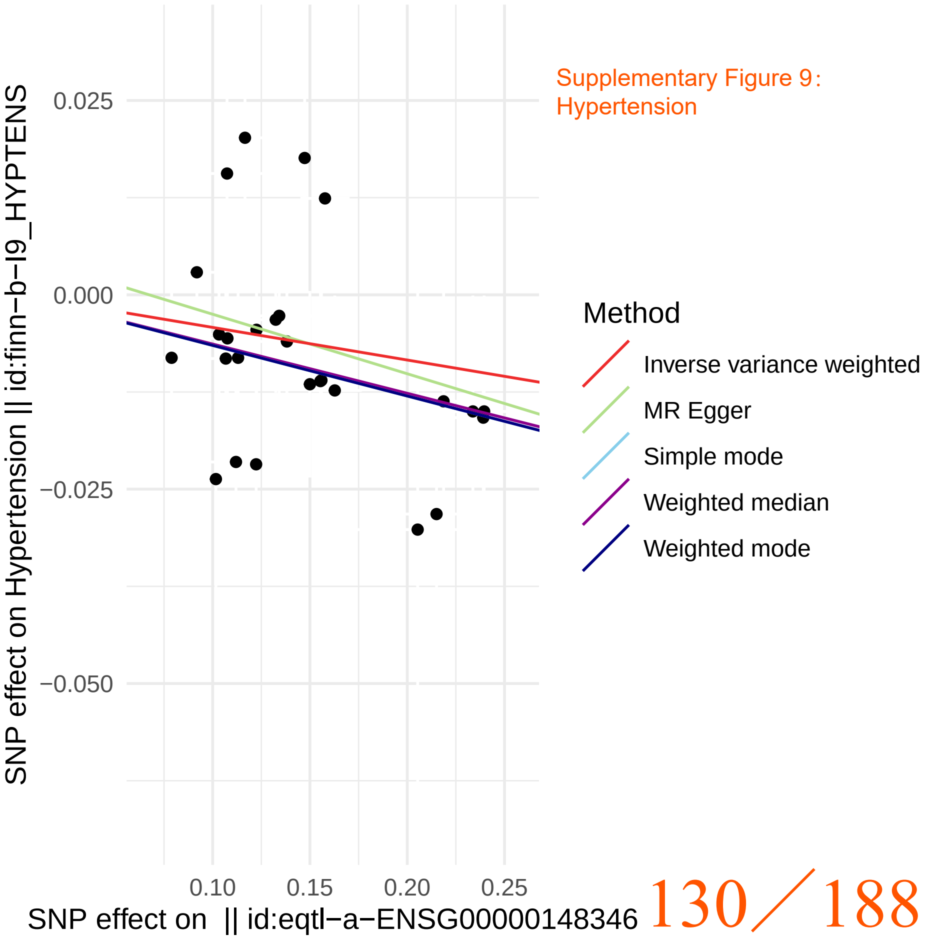

Supplementary Figure 9:
Osteoarthritis (hospital diagnosed)

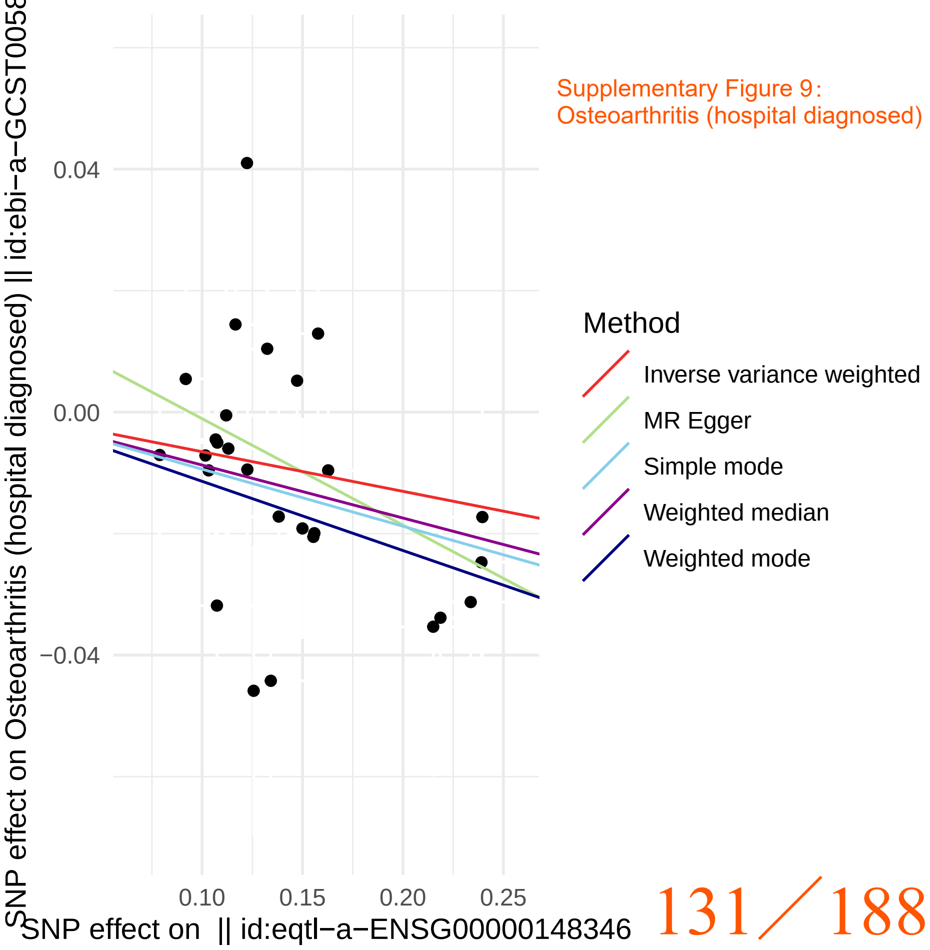

Supplementary Figure 9: Pancreatic cancer

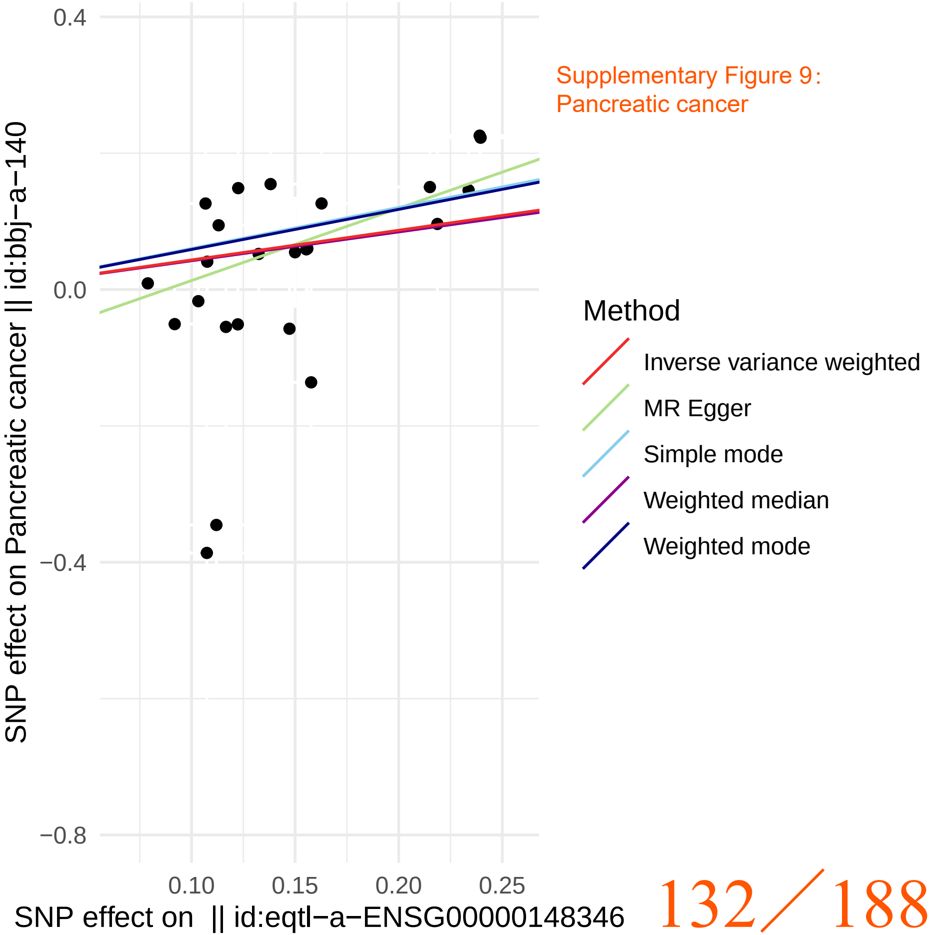

Supplementary Figure 9:
Rheumatoid arthritis (M13_RHEUMA)

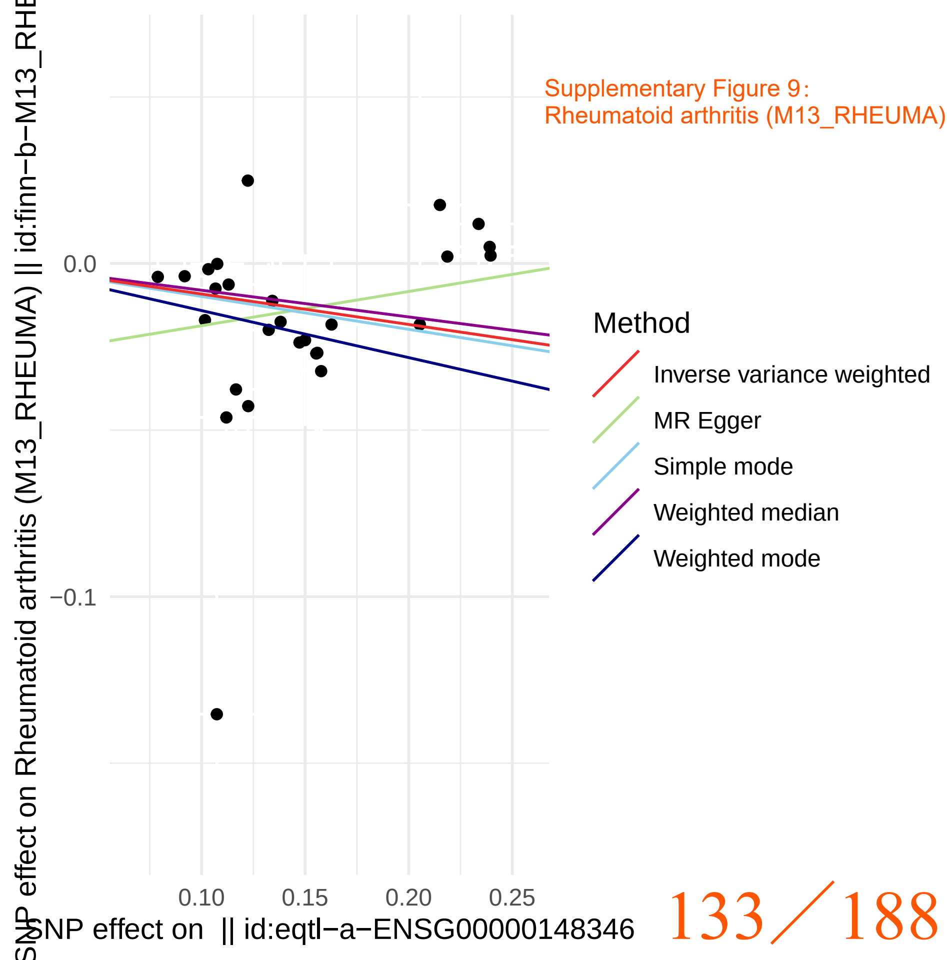

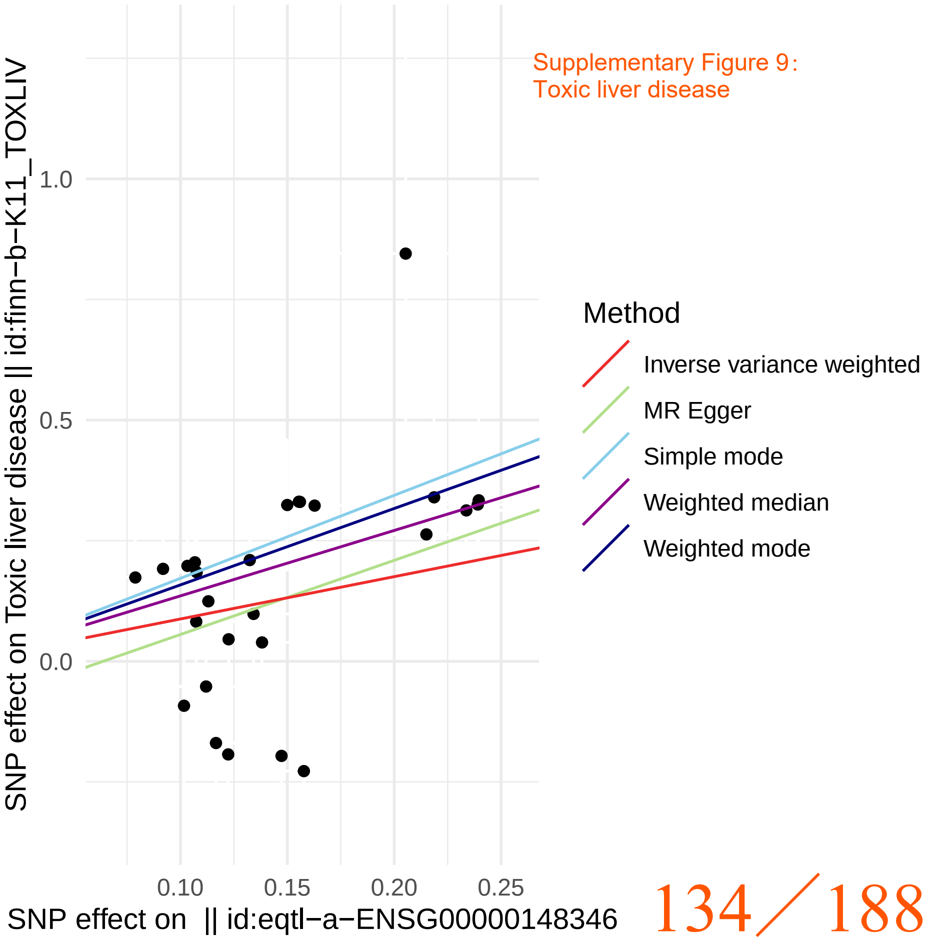
SNP effect on Toxic liver disease || id:finn-b-K11_TOXLIV
1.0
0.5
0.0
0.10
0.15
0.20
0.25
SNP effect on || id:eqtl-a-ENSG00000148346
Method
Inverse variance weighted
MR Egger
Simple mode
Weighted median
Weighted mode

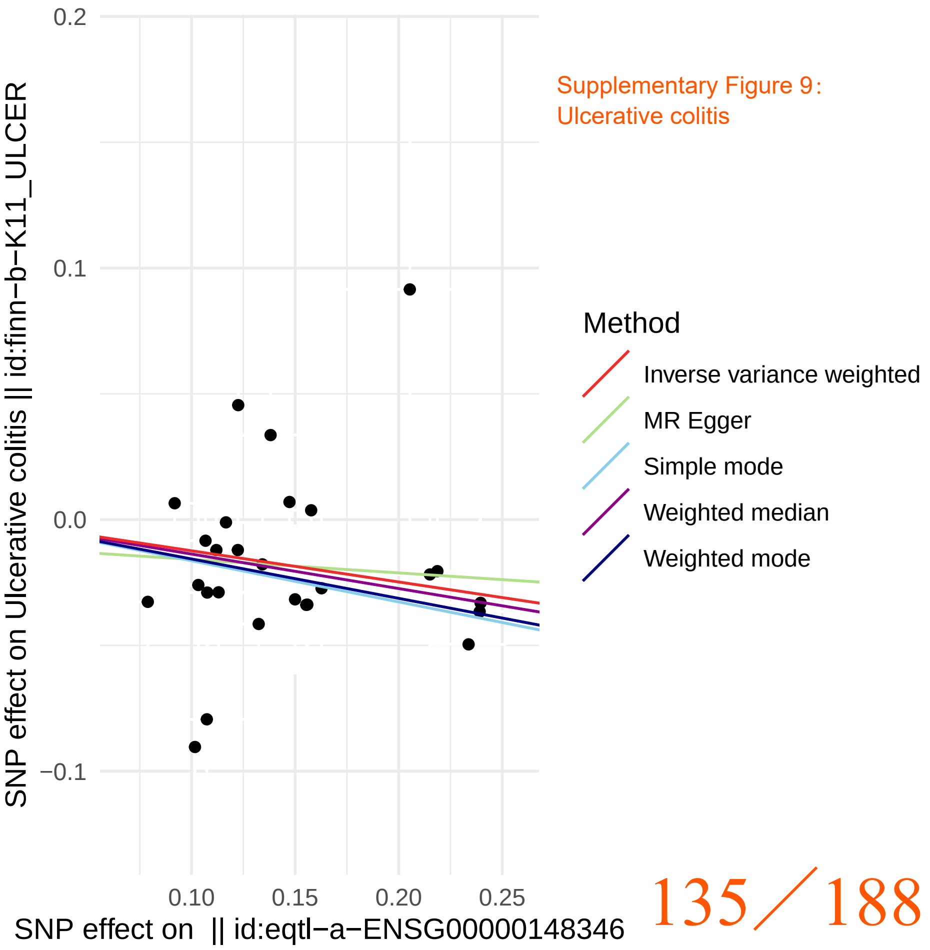
Supplementary Figure 9:
Ulcerative colitis
SNP effect on Ulcerative colitis || id:finn−b−K11_ULCER
SNP effect on || id:eqtl−a−ENSG00000148346
0.2
0.1
0.0
−0.1
0.10
0.15
0.20
0.25
Method
Inverse variance weighted
MR Egger
Simple mode
Weighted median
Weighted mode

Supplementary Figure 10: Dementia with Lewy bodies

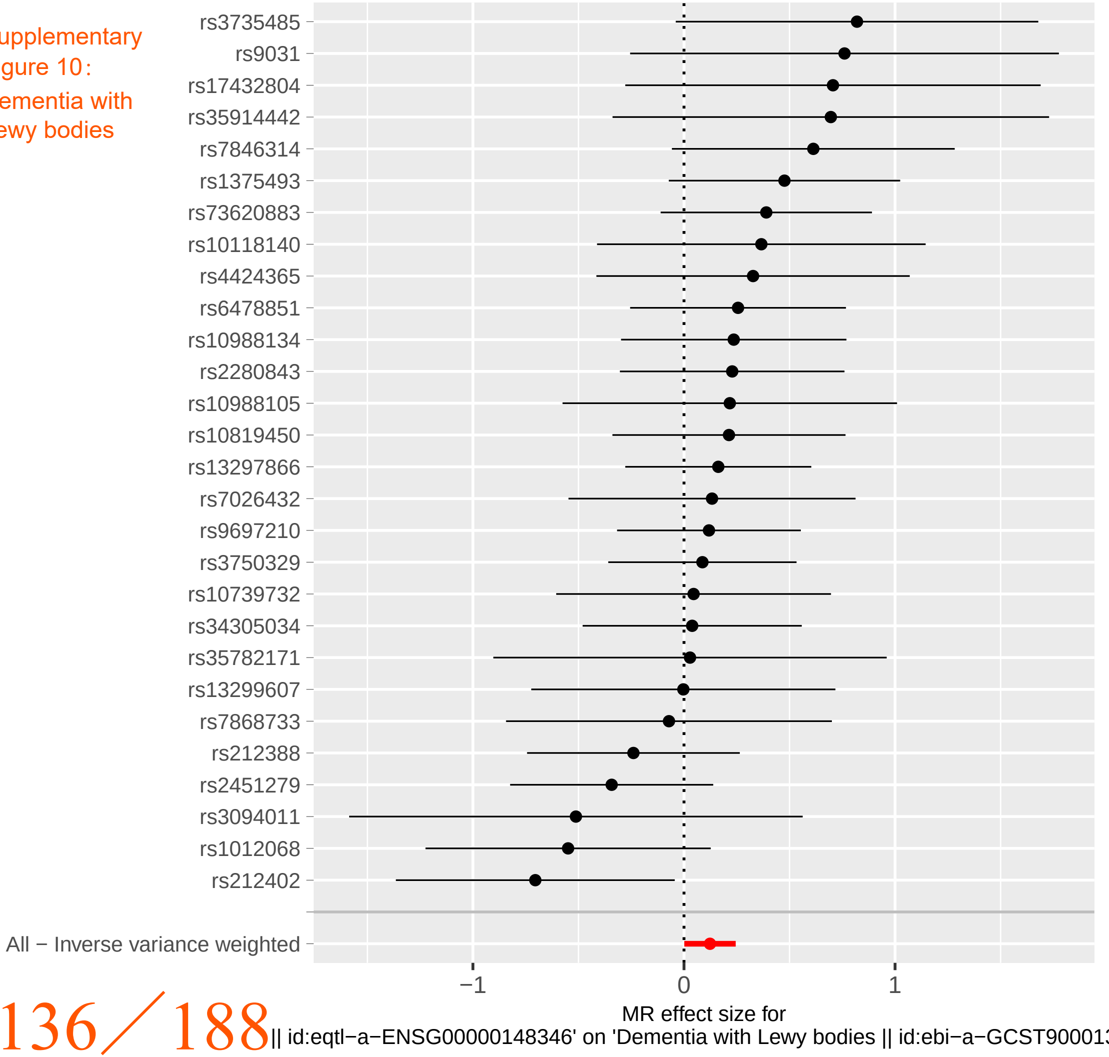

Supplementary Figure 10：Hypertension

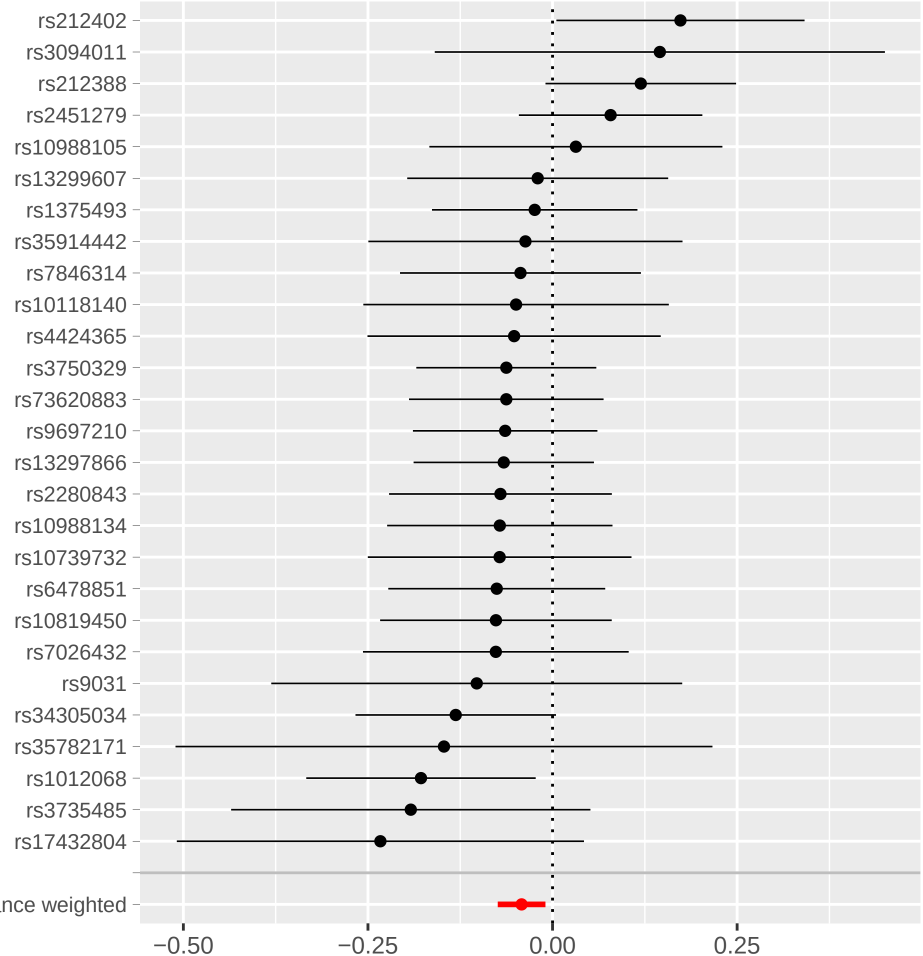

Supplementary Figure 10：Osteoarthritis (hospital diagnosed)

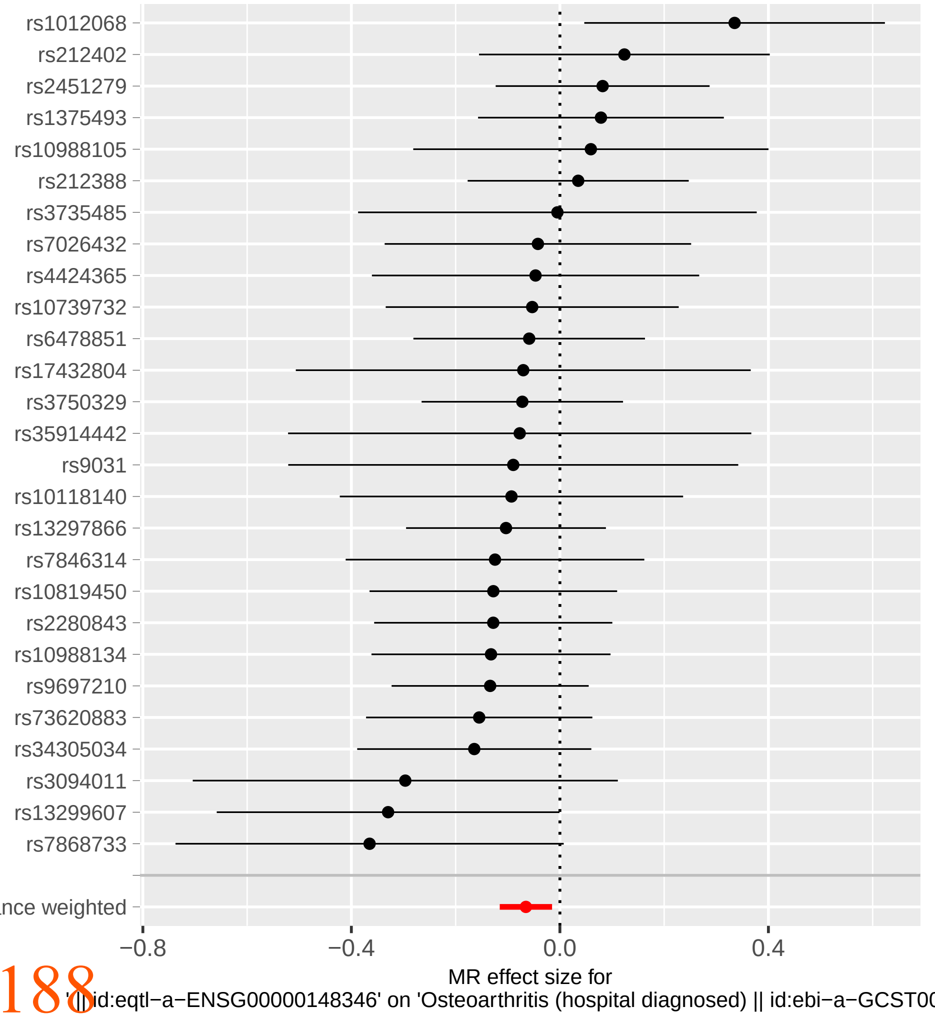

Supplementary Figure 10: Pancreatic cancer

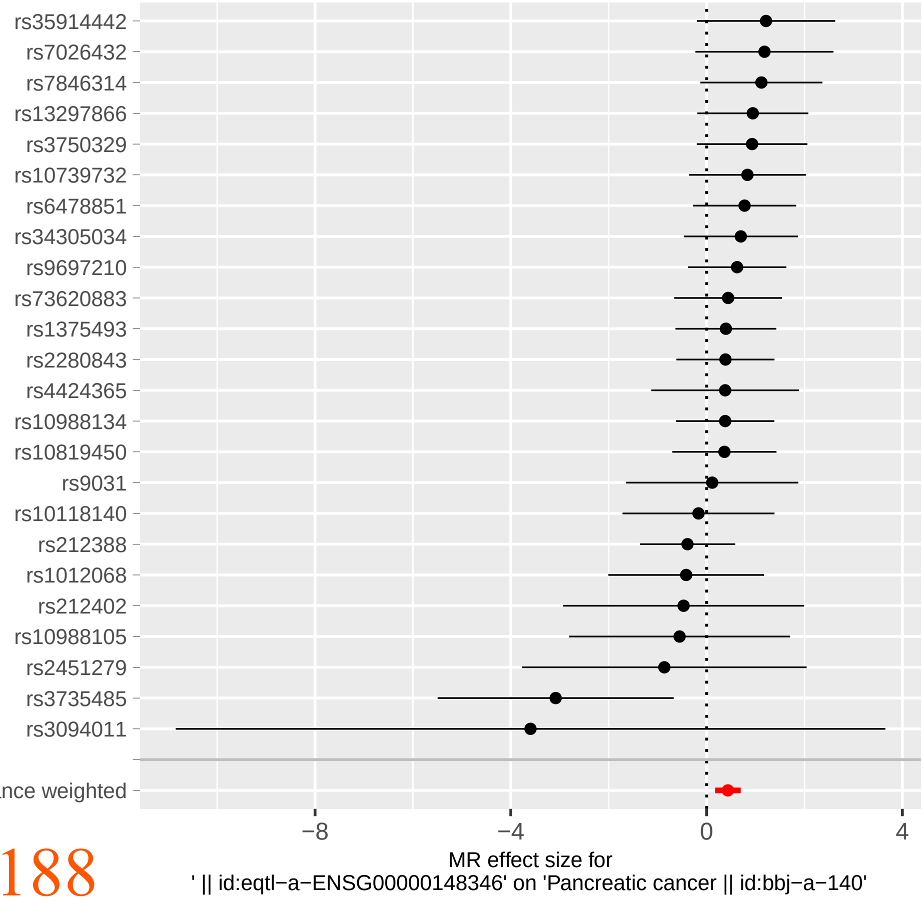

Supplementary Figure 10：Rheumatoid arthritis (M13_RHEUMA)

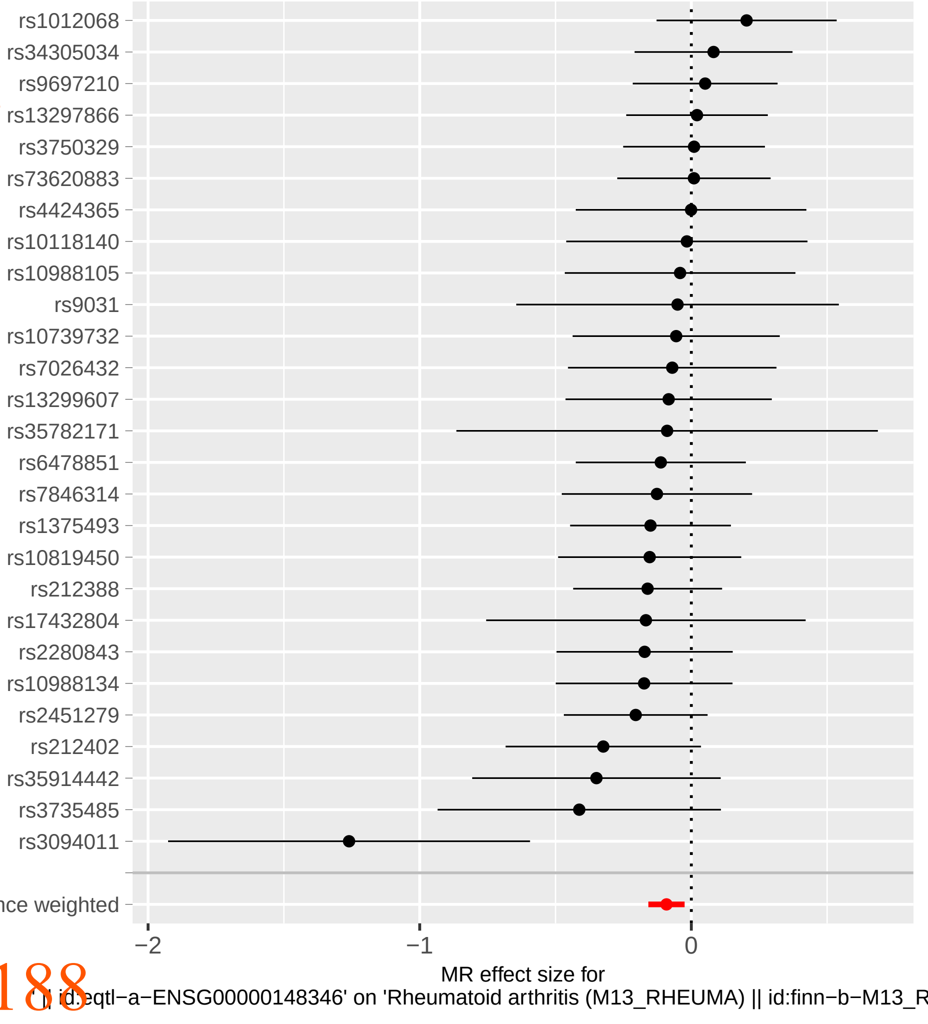

Supplementary Figure 10：
Toxic liver disease

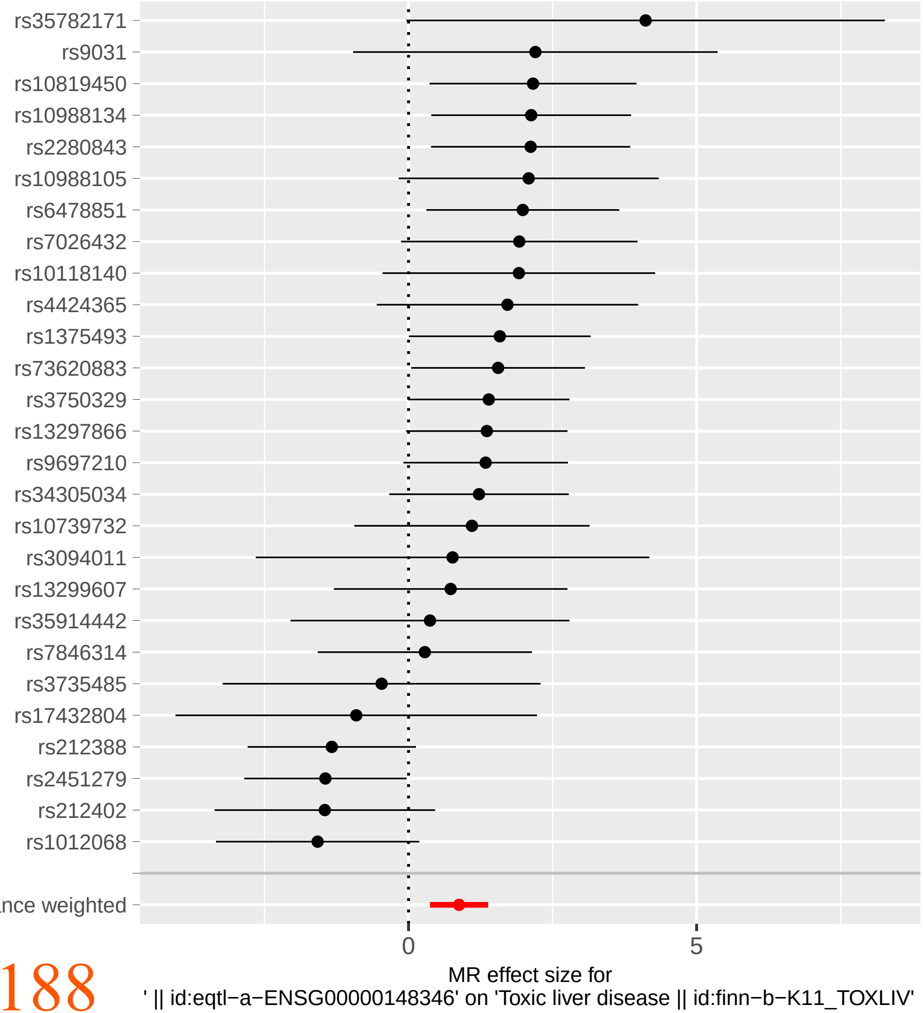

Supplementary Figure 10: Ulcerative colitis

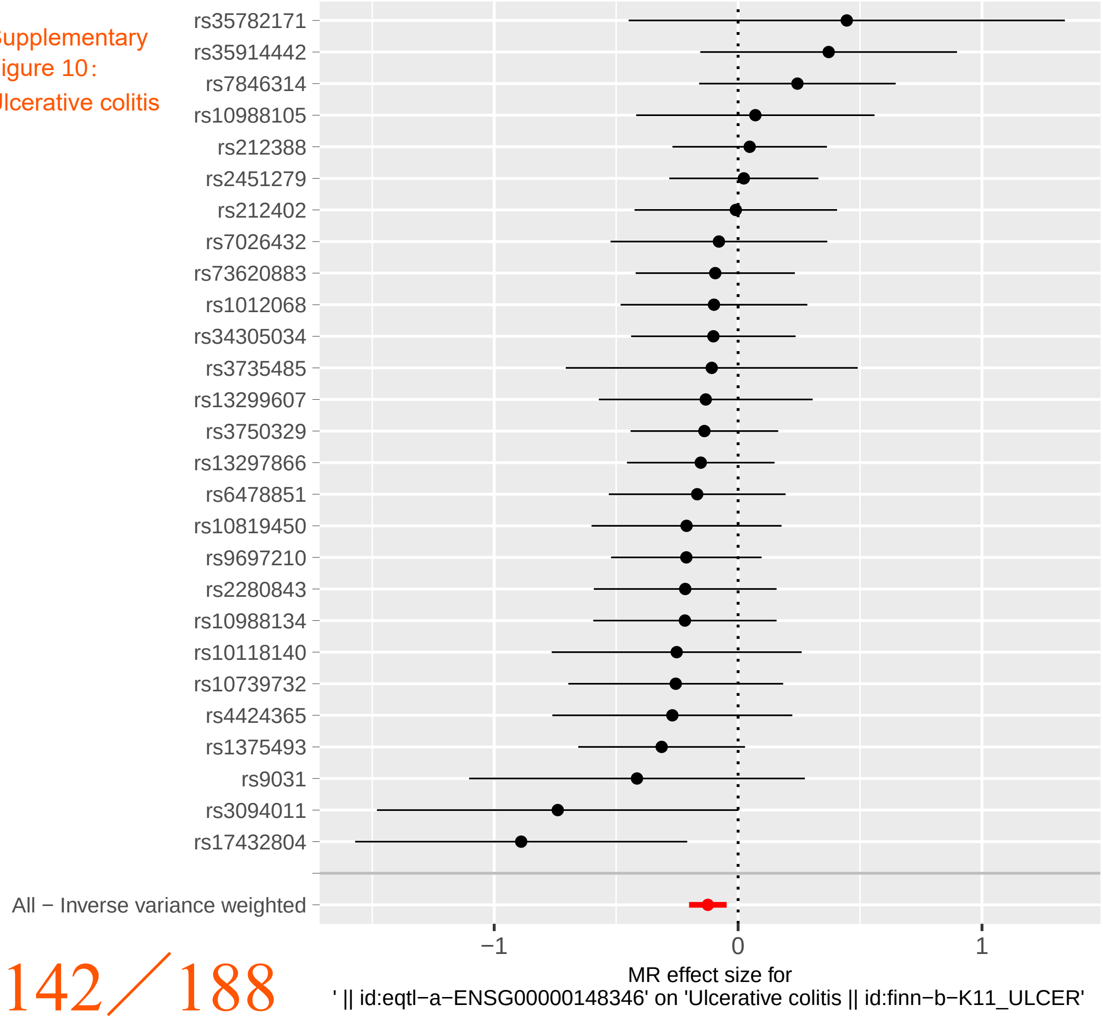

Supplementary Figure 11:
Dementia with Lewy bodies

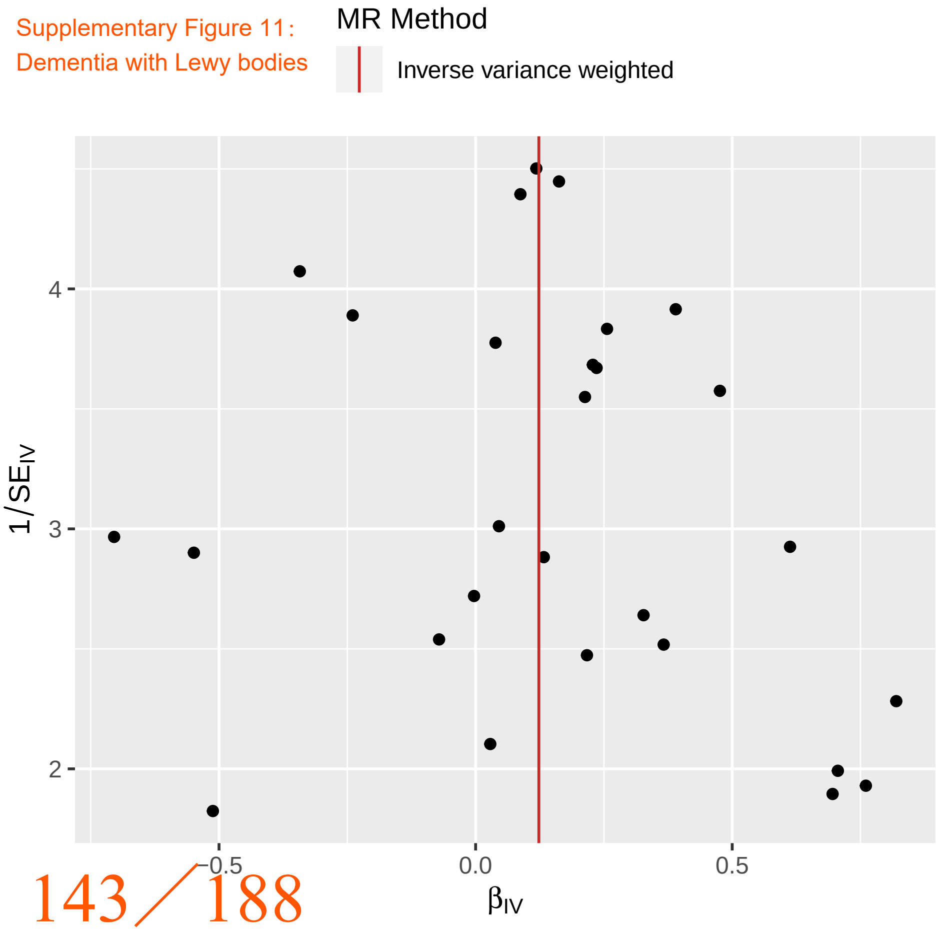

Supplementary Figure 11:
Hypertension

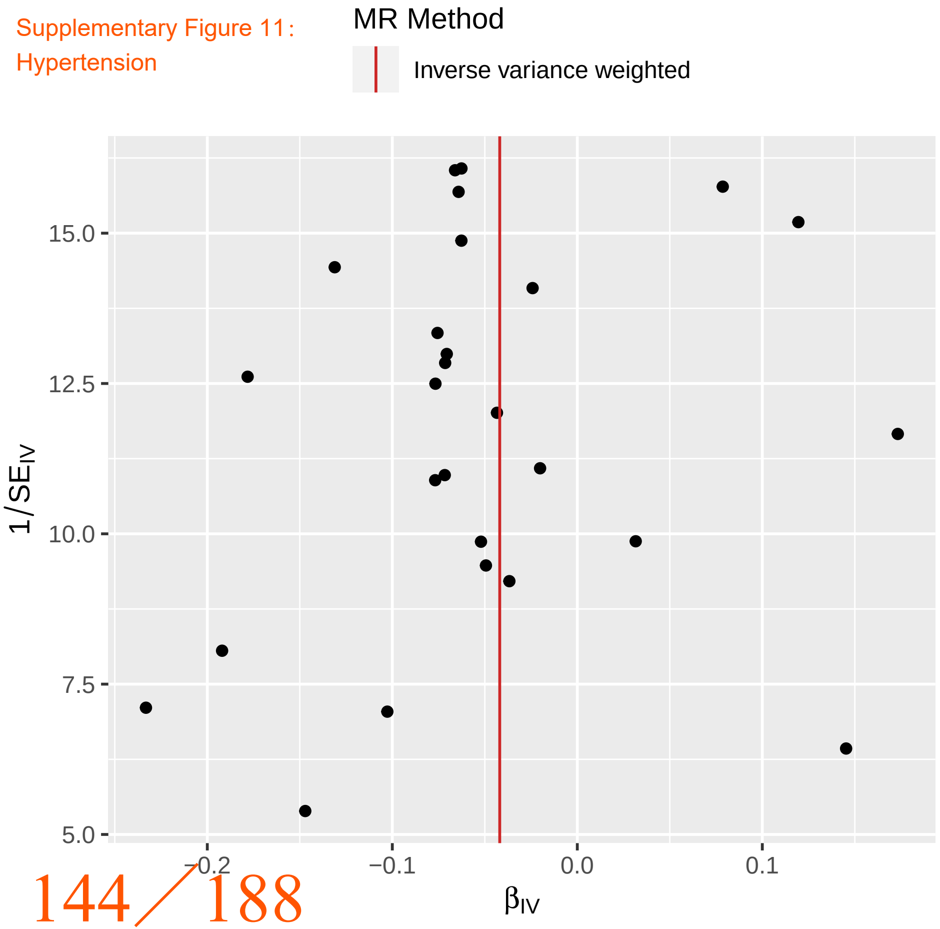

Supplementary Figure 11: Osteoarthritis (hospital diagnosed)

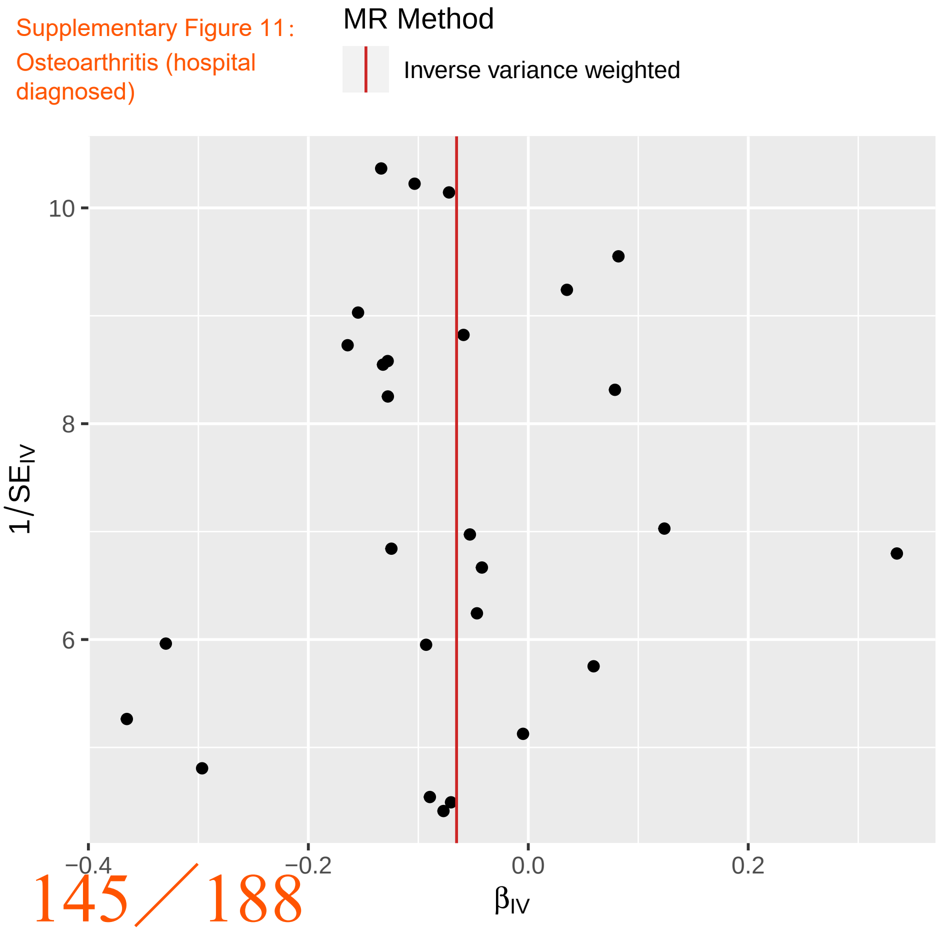

Supplementary Figure 11:
Pancreatic cancer

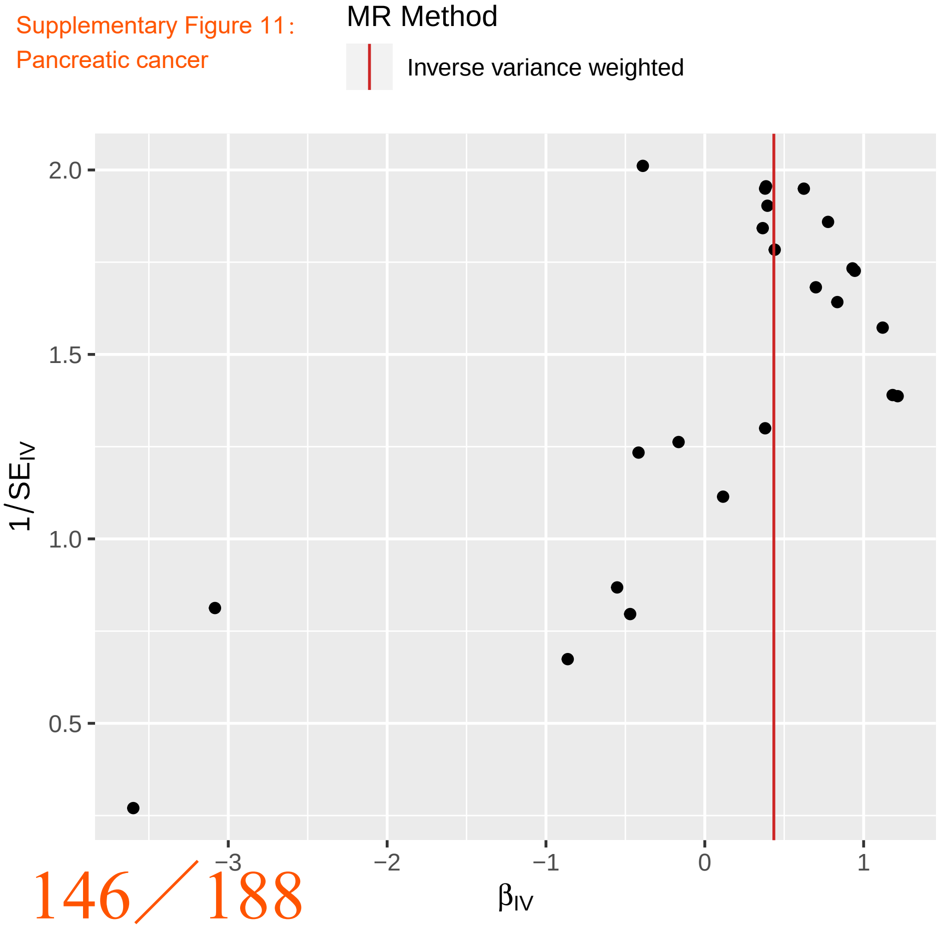

Supplementary Figure 11:
Rheumatoid arthritis
(M13_RHEUMA)

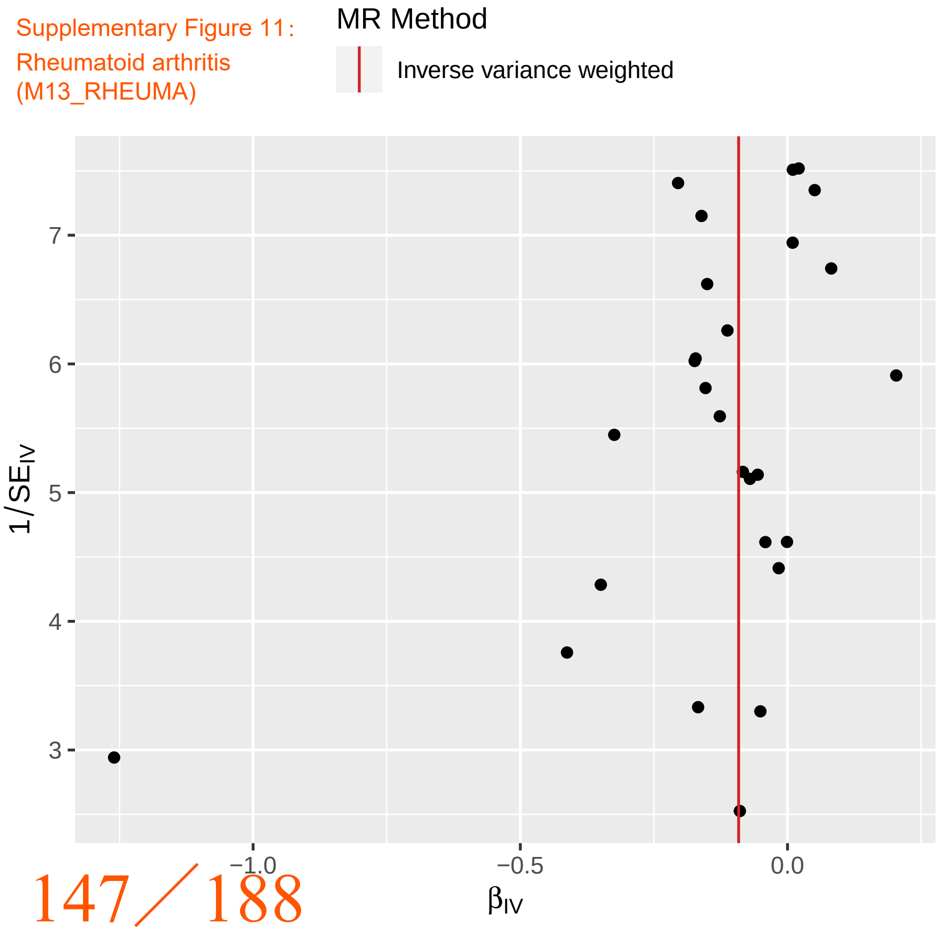

Supplementary Figure 11 :
Toxic liver disease

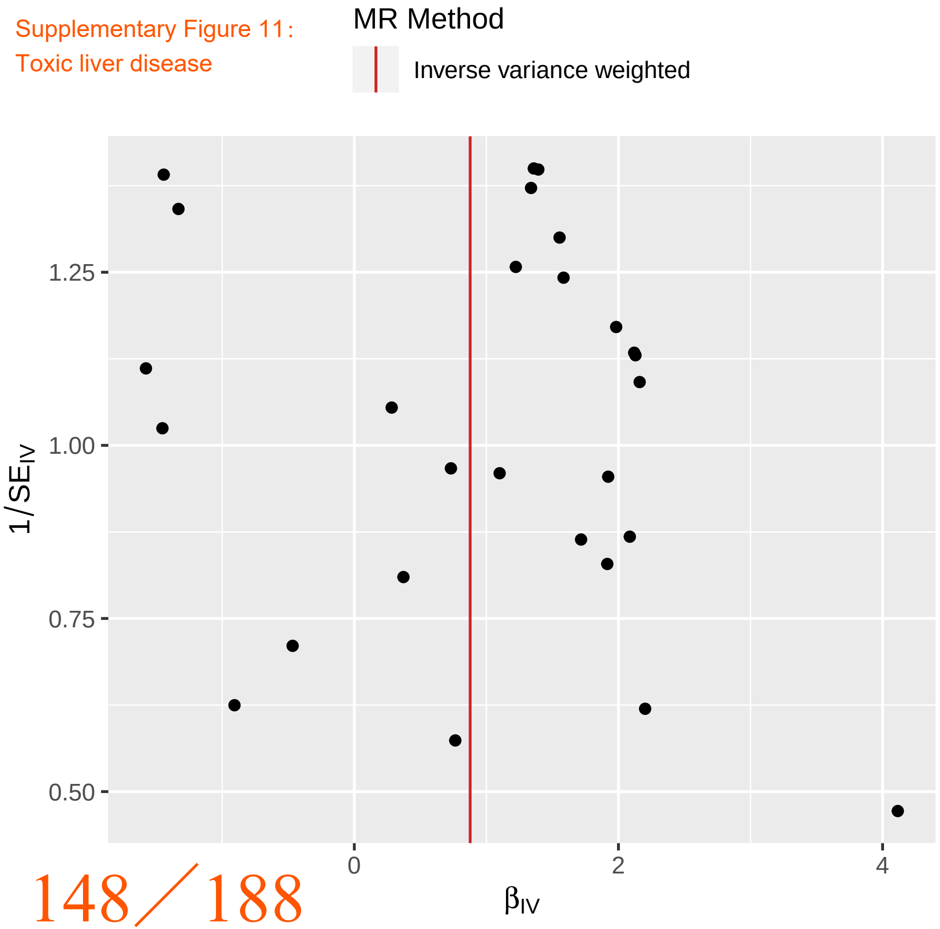

Supplementary Figure 11:
Ulcerative colitis

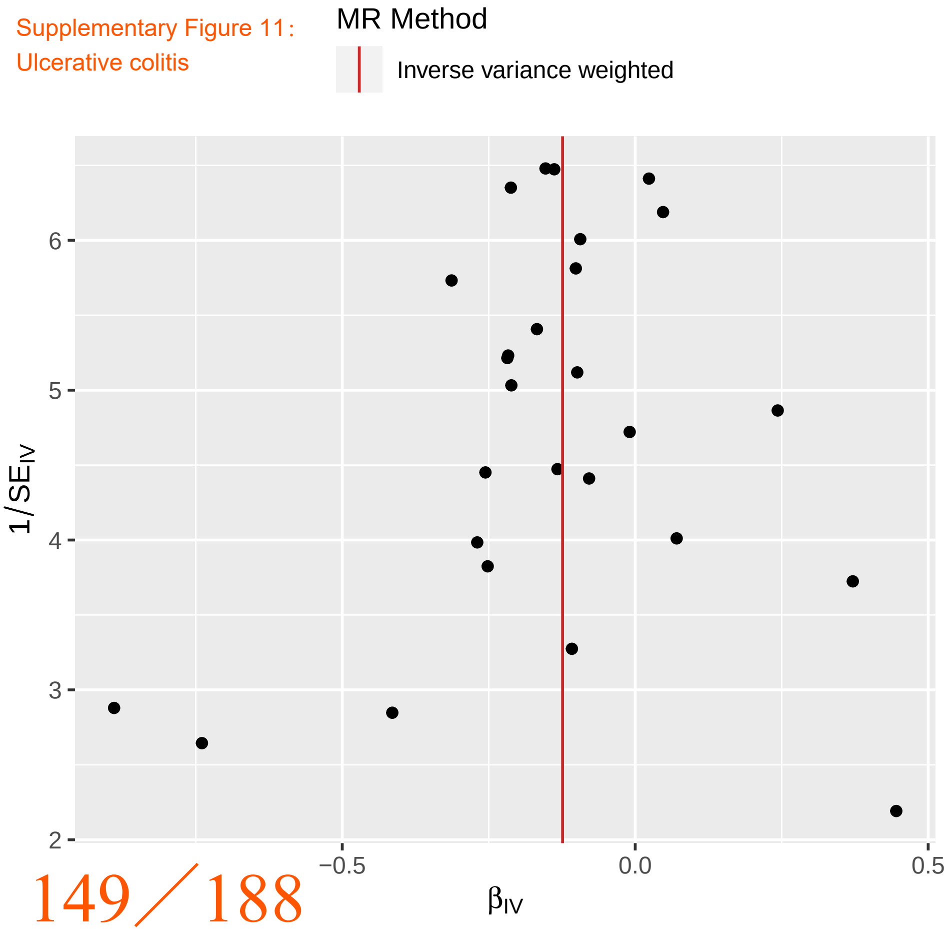

Supplementary Figure 12：
Dementia with Lewy bodies

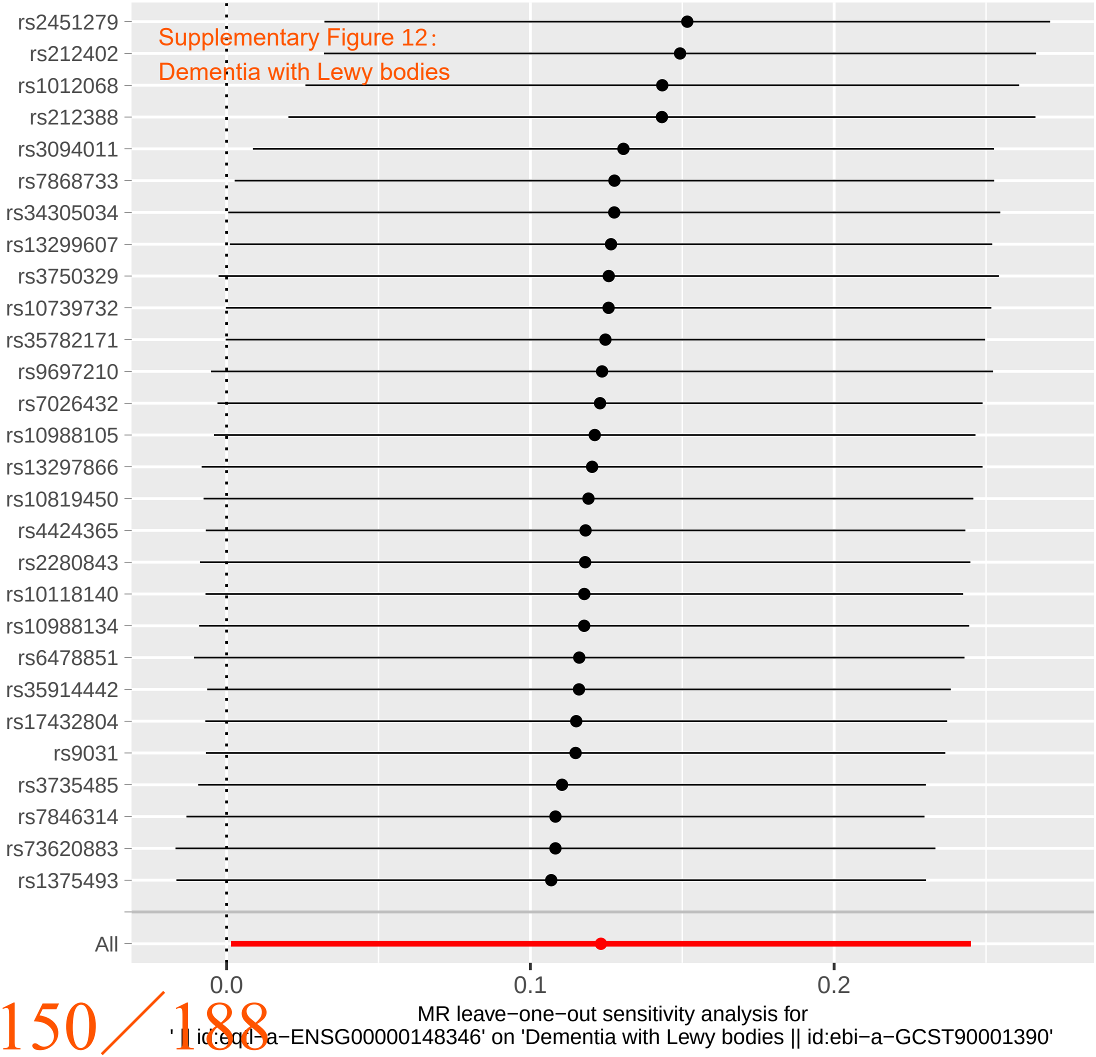

Supplementary Figure 12：Hypertension

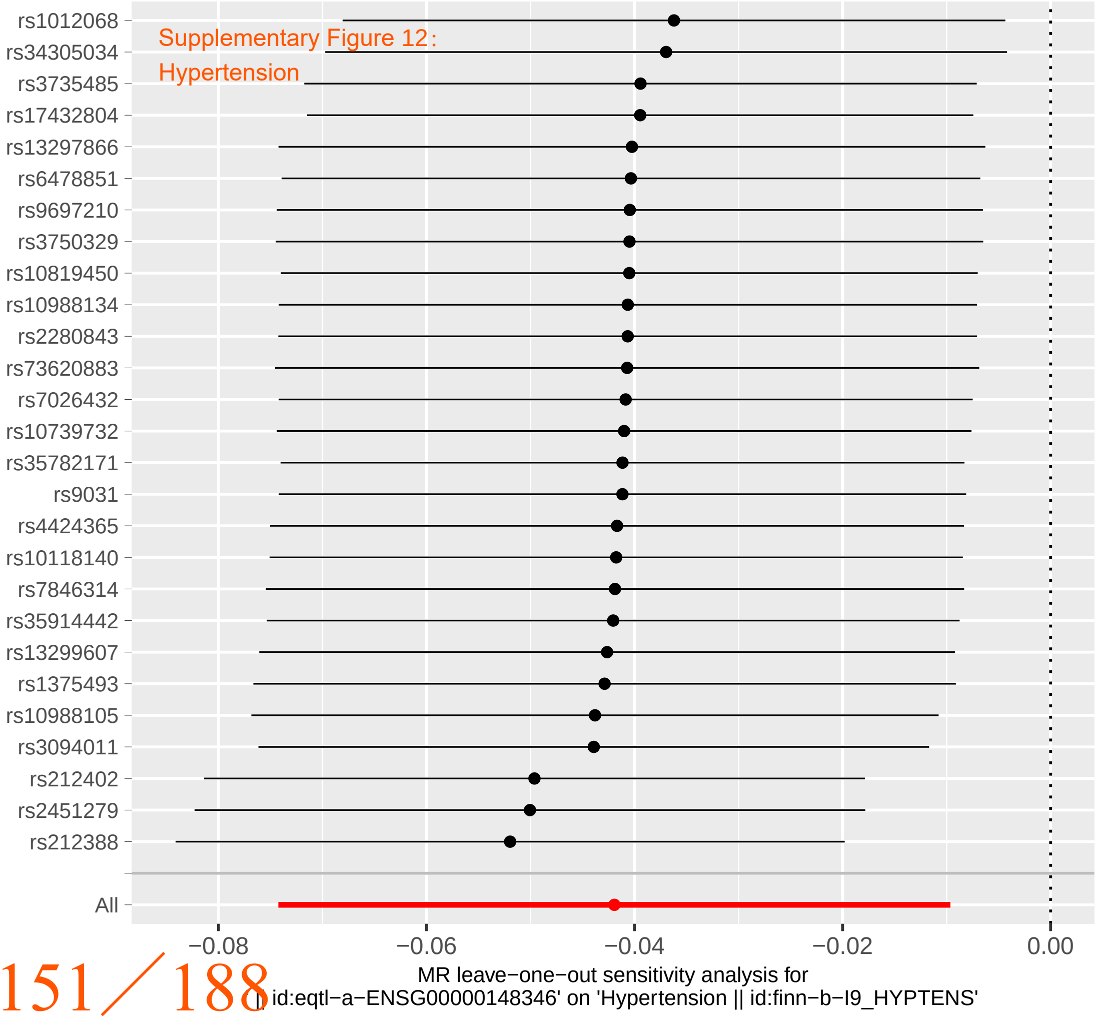

Supplementary Figure 12：Osteoarthritis (hospital diagnosed)

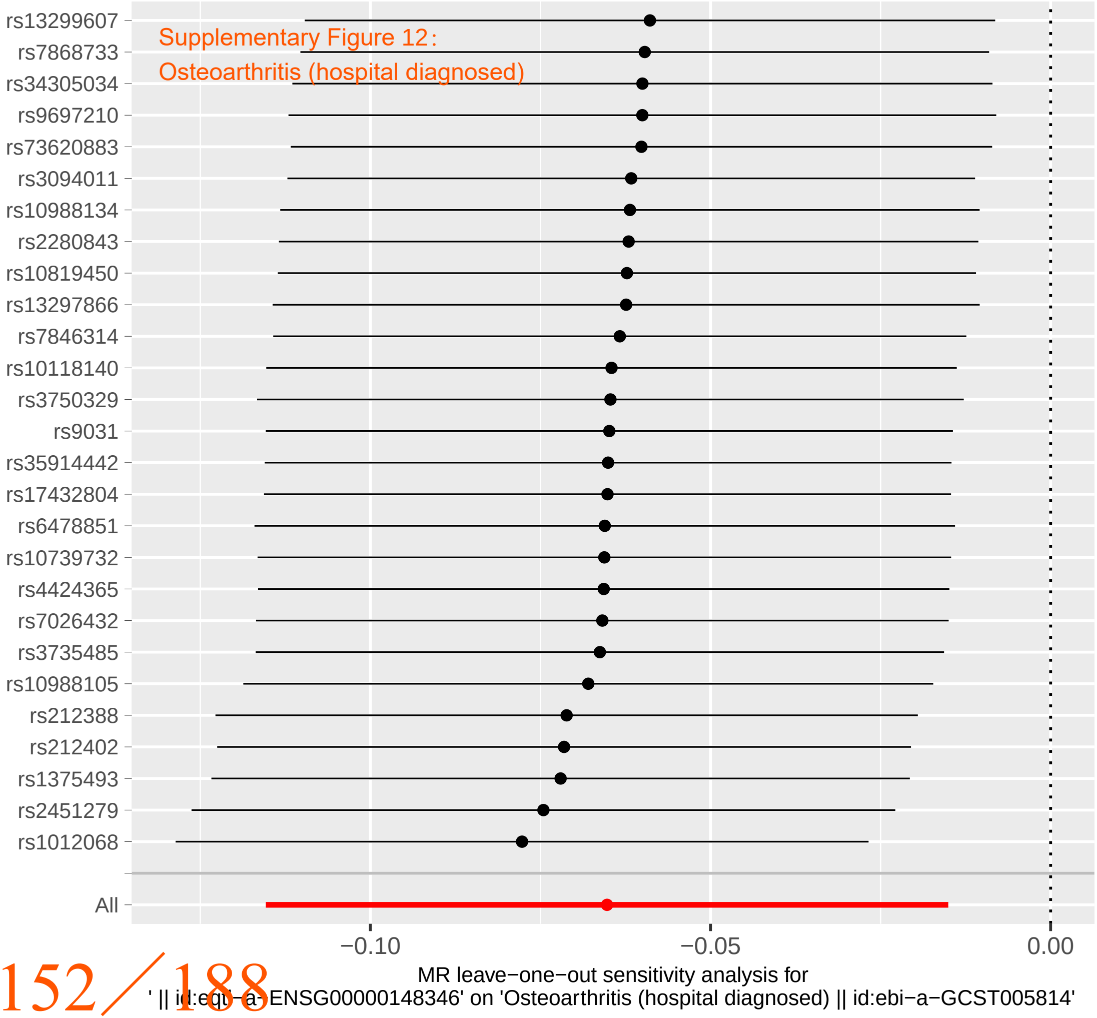

Supplementary Figure 12: Pancreatic cancer

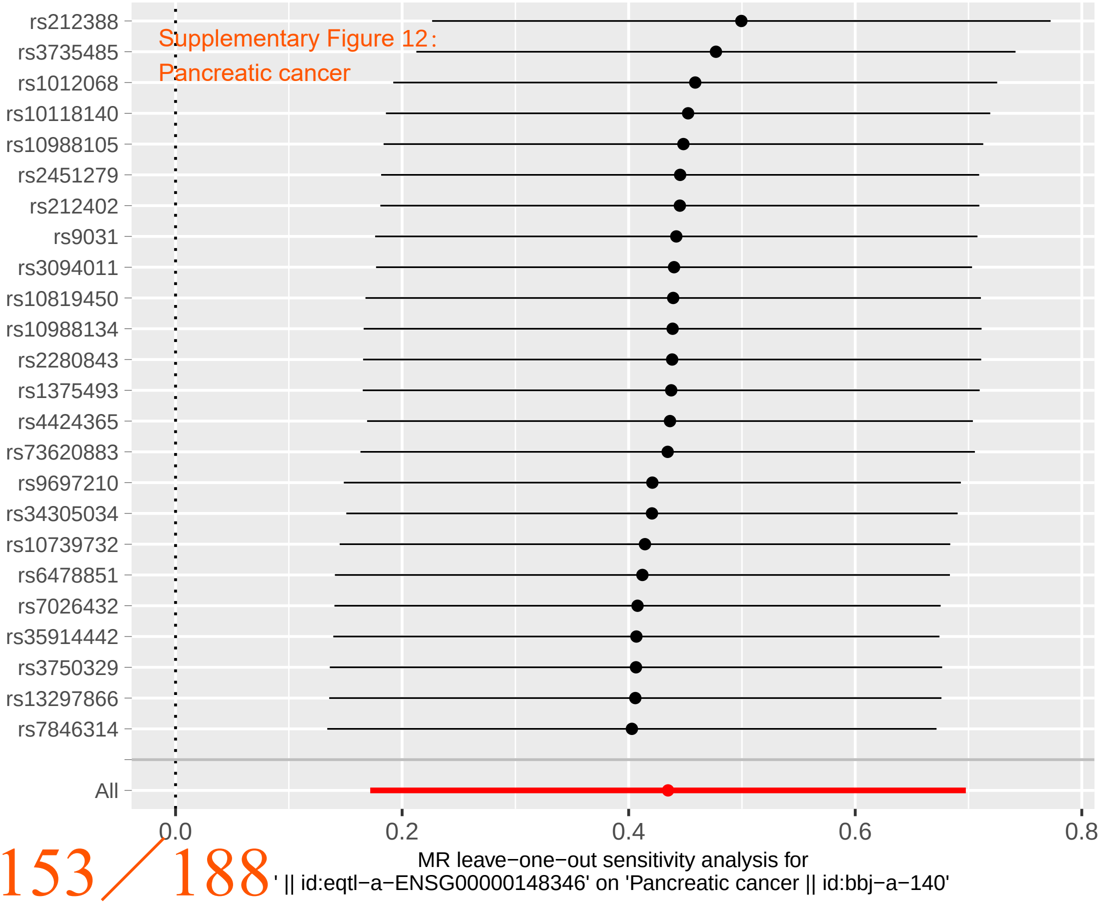

Supplementary Figure 12：

Rheumatoid arthritis (M13_RHEUMA)

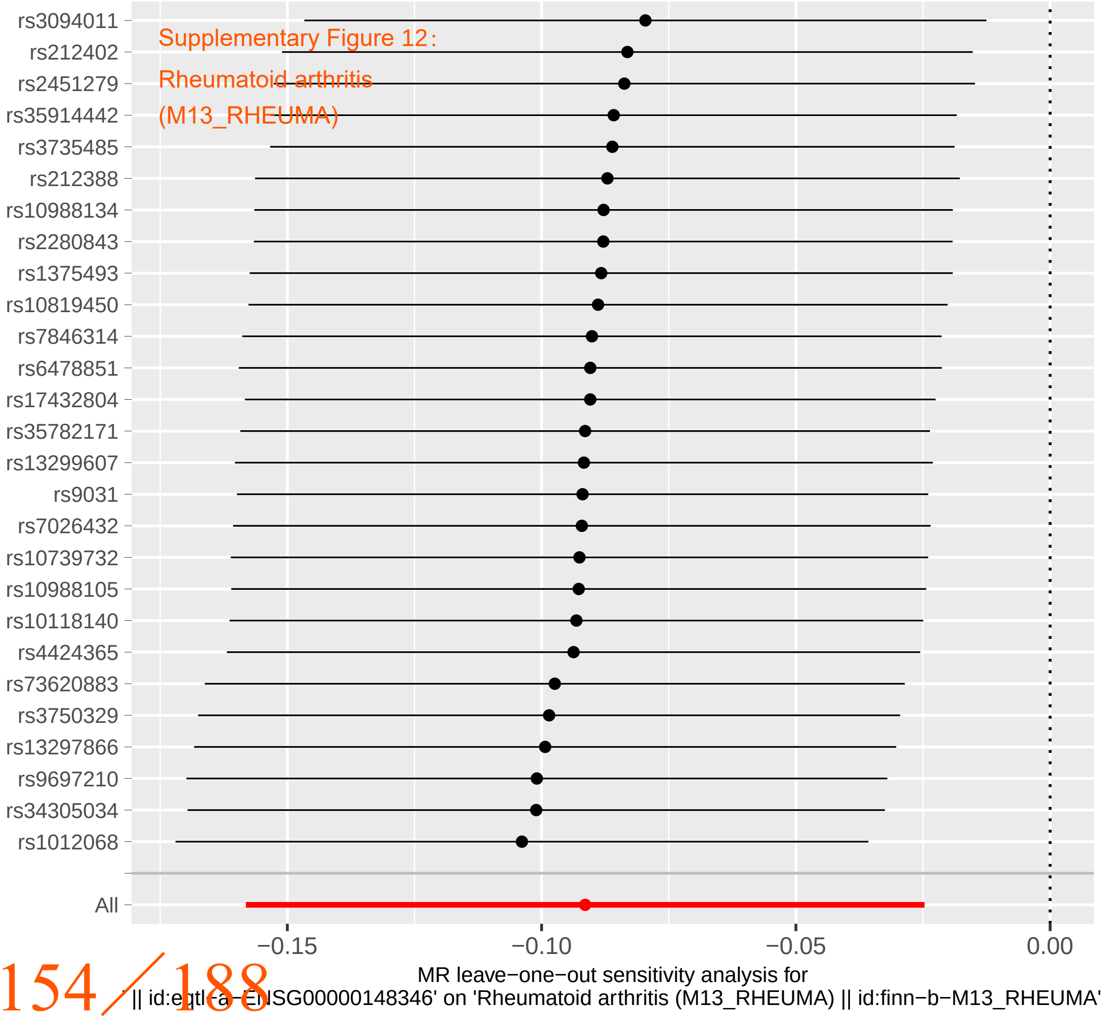

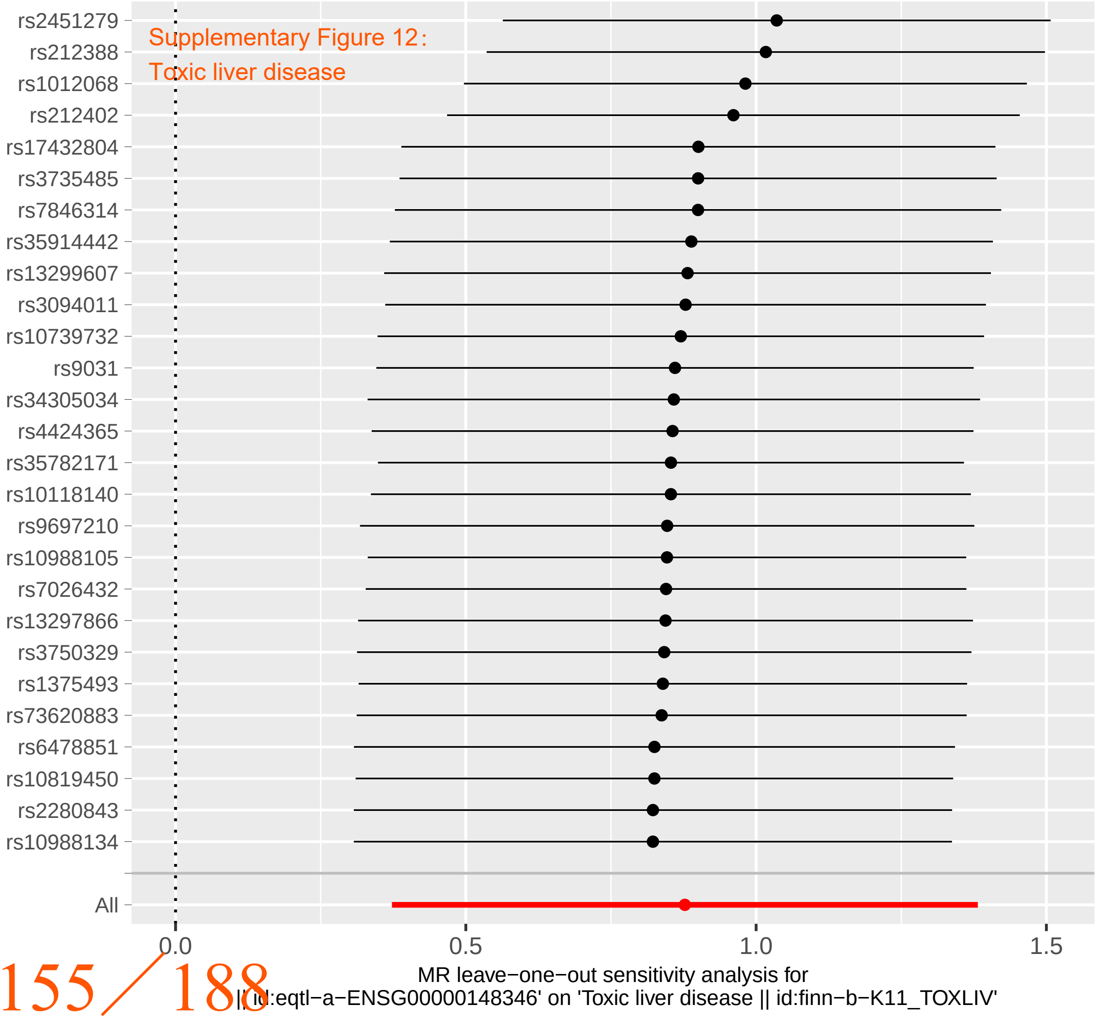
Supplementary Figure 12:
Toxic liver disease
rs2451279
rs212388
rs1012068
rs212402
rs17432804
rs3735485
rs7846314
rs35914442
rs13299607
rs3094011
rs10739732
rs9031
rs34305034
rs4424365
rs35782171
rs10118140
rs9697210
rs10988105
rs7026432
rs13297866
rs3750329
rs1375493
rs73620883
rs6478851
rs10819450
rs2280843
rs10988134
All
0.0
0.5
1.0
1.5
MR leave−one−out sensitivity analysis for
'id:eqtl−a−ENSG00000148346' on 'Toxic liver disease || id:finn−b−K11_TOXLIV'

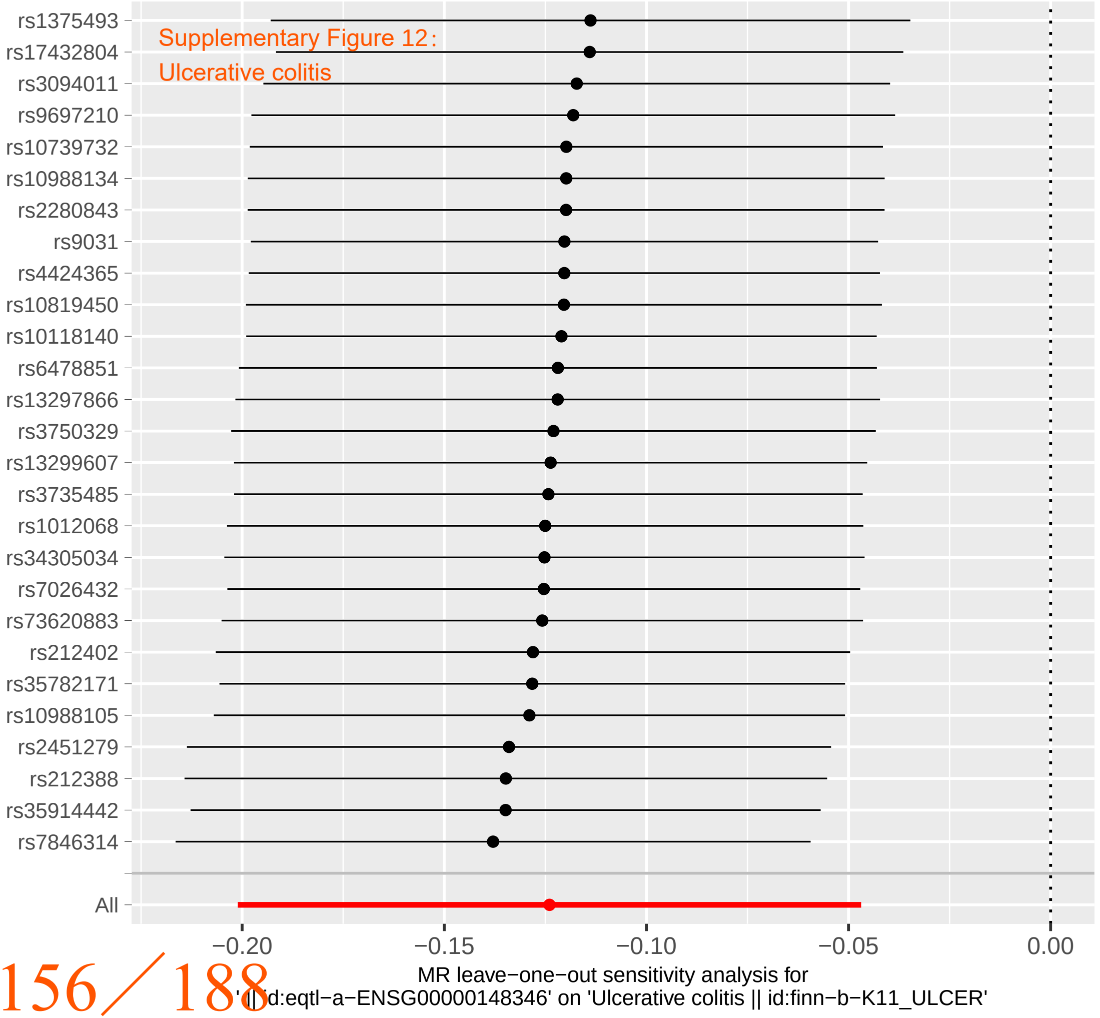
Supplementary Figure 12：
Ulcerative colitis
rs1375493
rs17432804
rs3094011
rs9697210
rs10739732
rs10988134
rs2280843
rs9031
rs4424365
rs10819450
rs10118140
rs6478851
rs13297866
rs3750329
rs13299607
rs3735485
rs1012068
rs34305034
rs7026432
rs73620883
rs212402
rs35782171
rs10988105
rs2451279
rs212388
rs35914442
rs7846314
All
−0.20
−0.15
−0.10
−0.05
0.00
MR leave−one−out sensitivity analysis for
'|| id:eqtl−a−ENSG00000148346' on 'Ulcerative colitis || id:finn−b−K11_ULCER'

Supplementary Figure 13:
Asthma

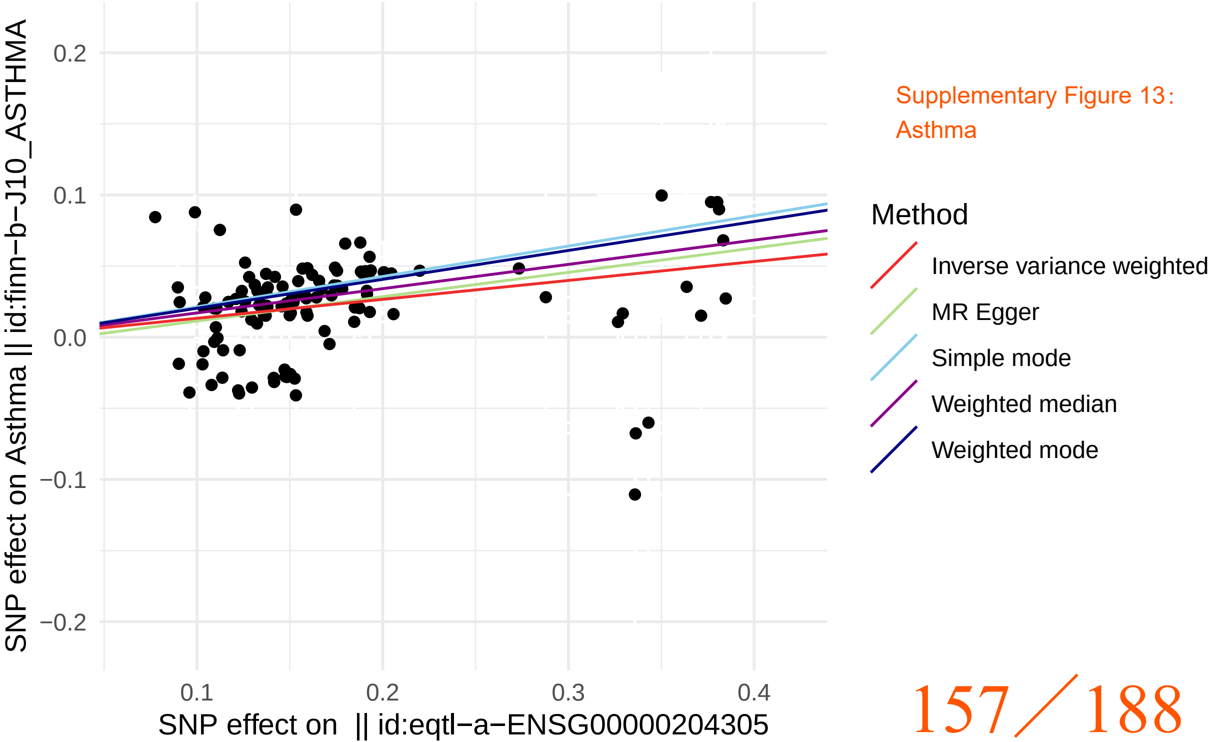

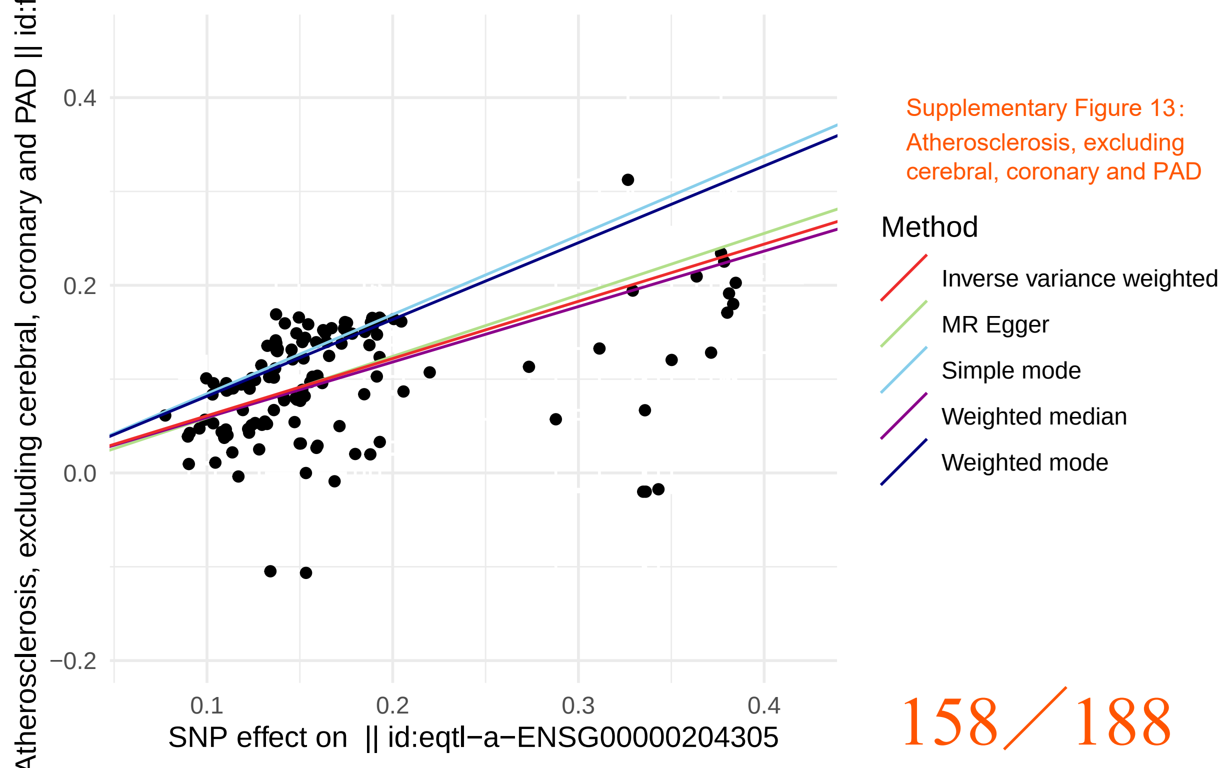

Supplementary Figure 13: Atherosclerosis, excluding cerebral, coronary and PAD

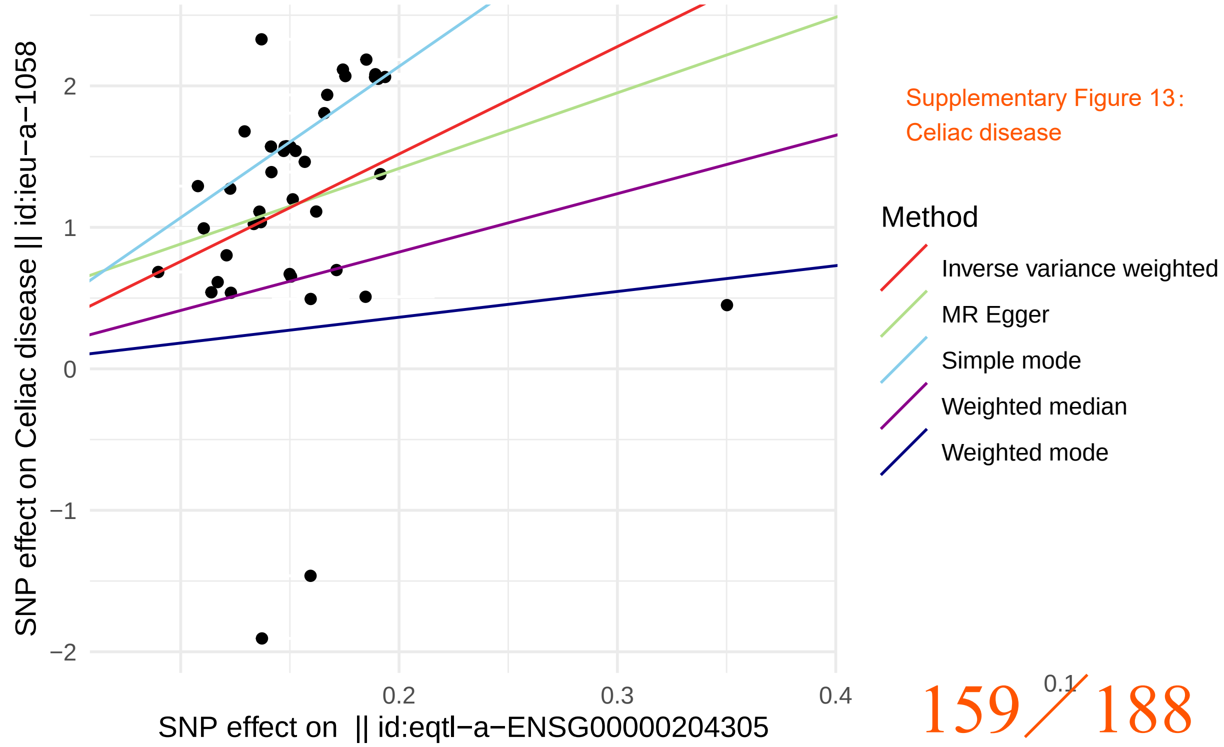

Supplementary Figure 13:
Celiac disease

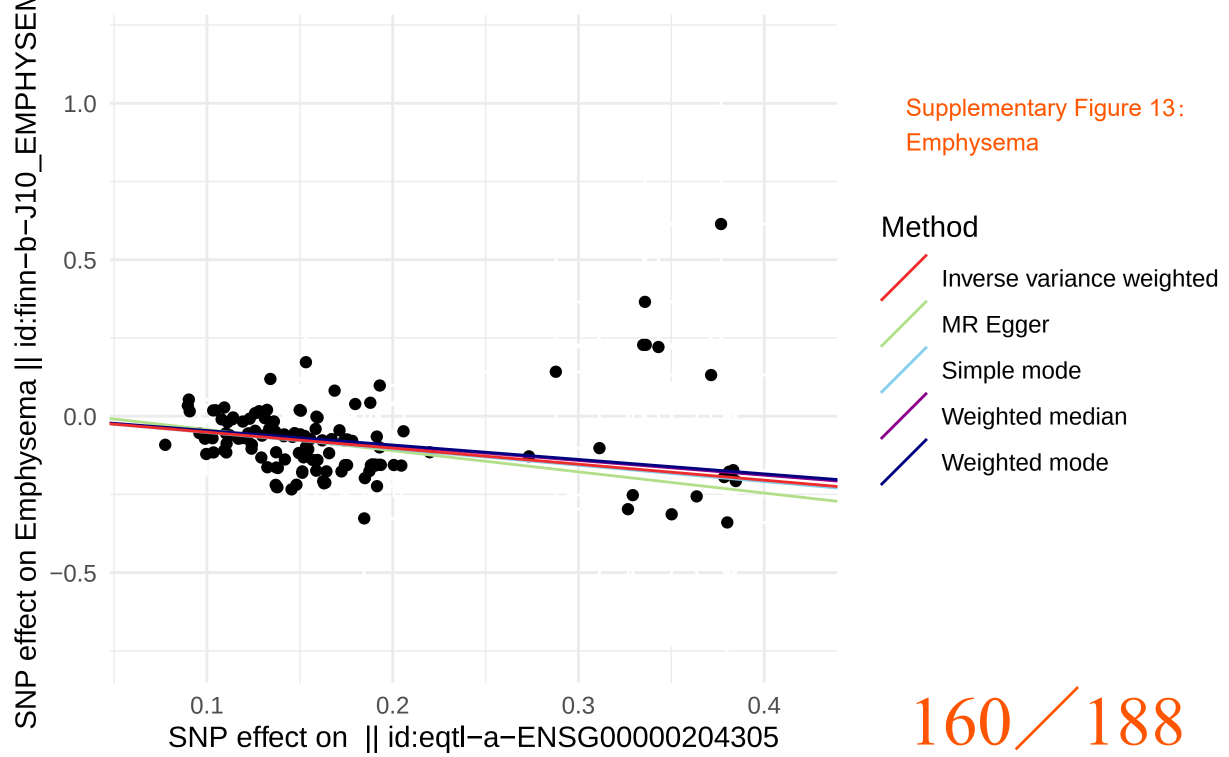

Supplementary Figure 13: Emphysema

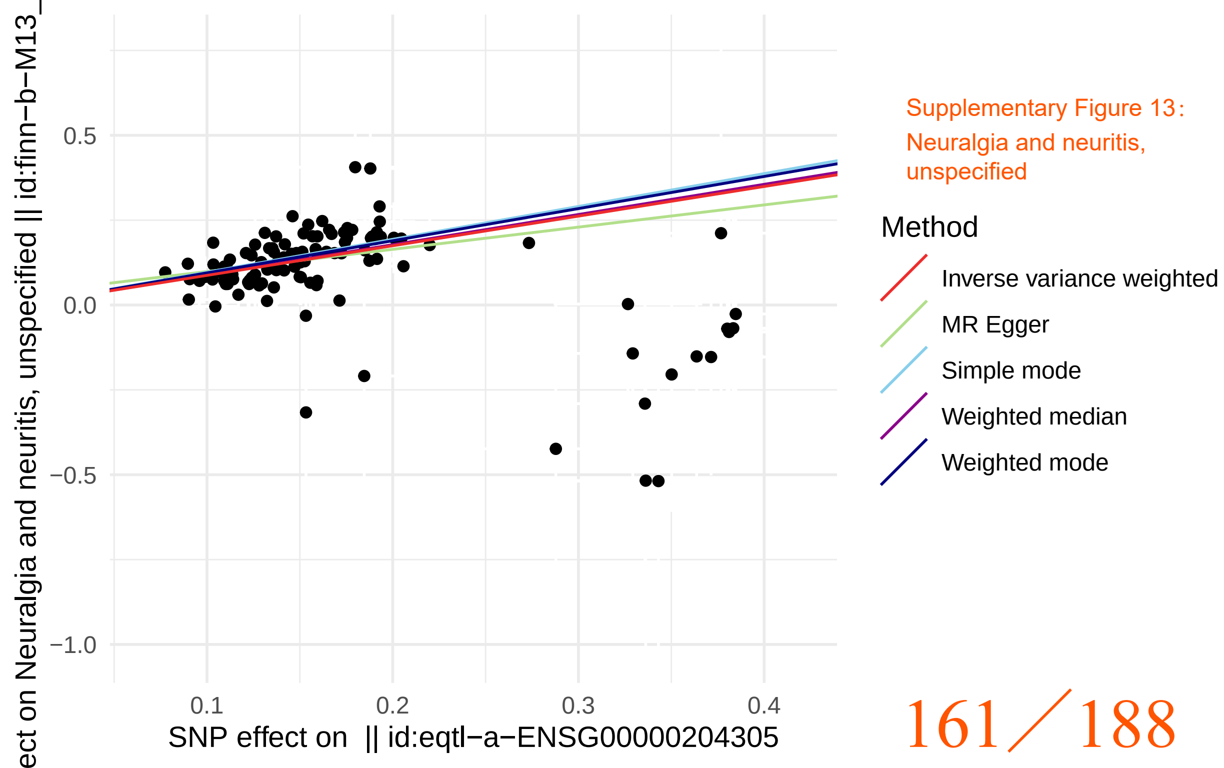

Supplementary Figure 13: Neuralgia and neuritis, unspecified

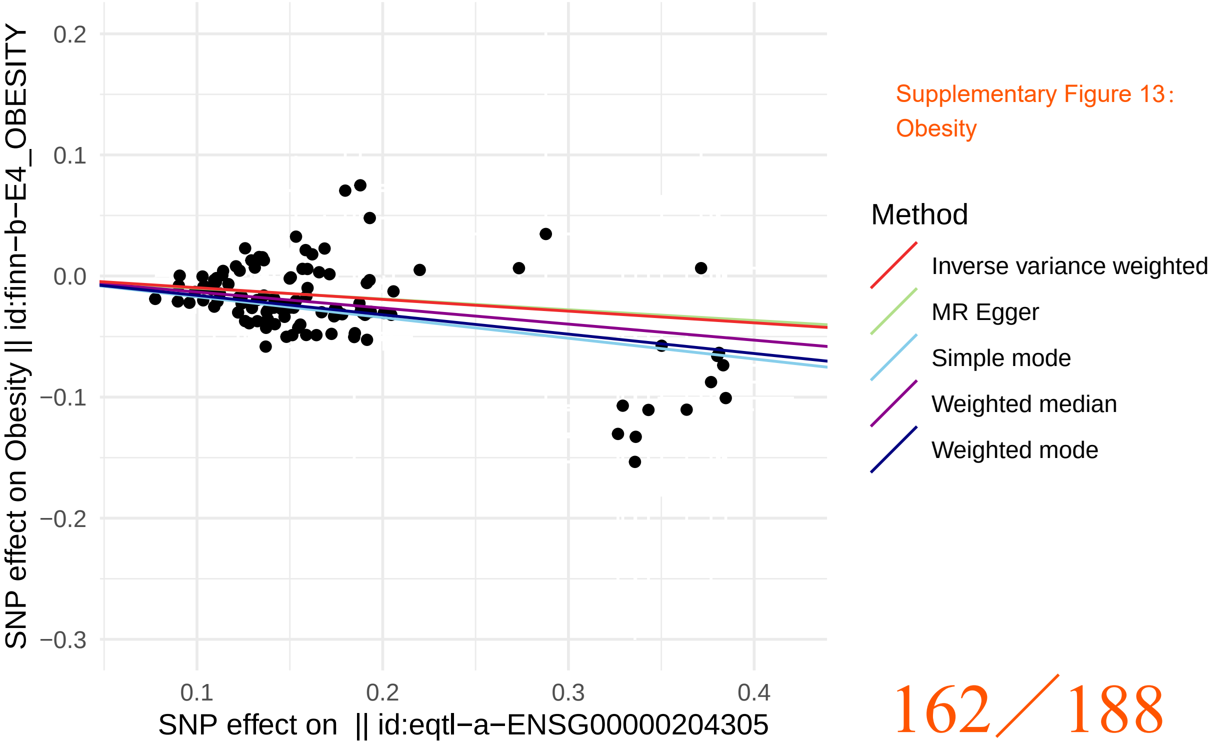

Supplementary Figure 13: Obesity

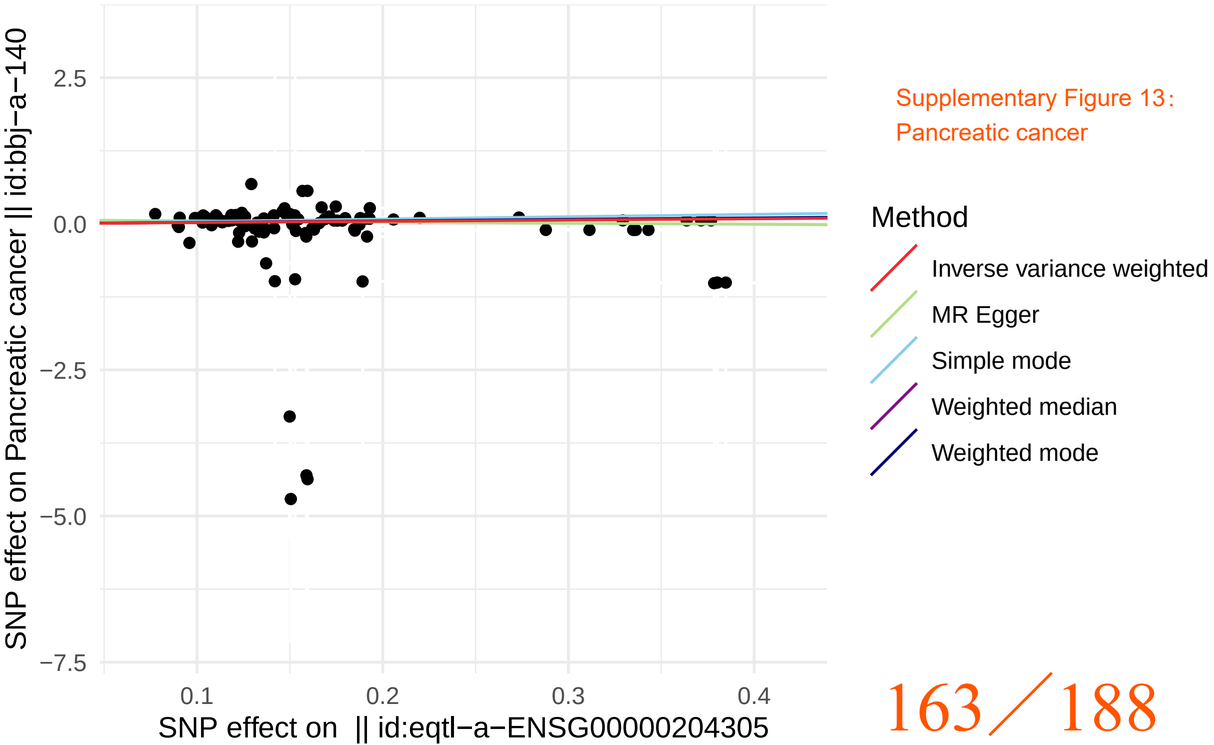

Supplementary Figure 13:
Pancreatic cancer

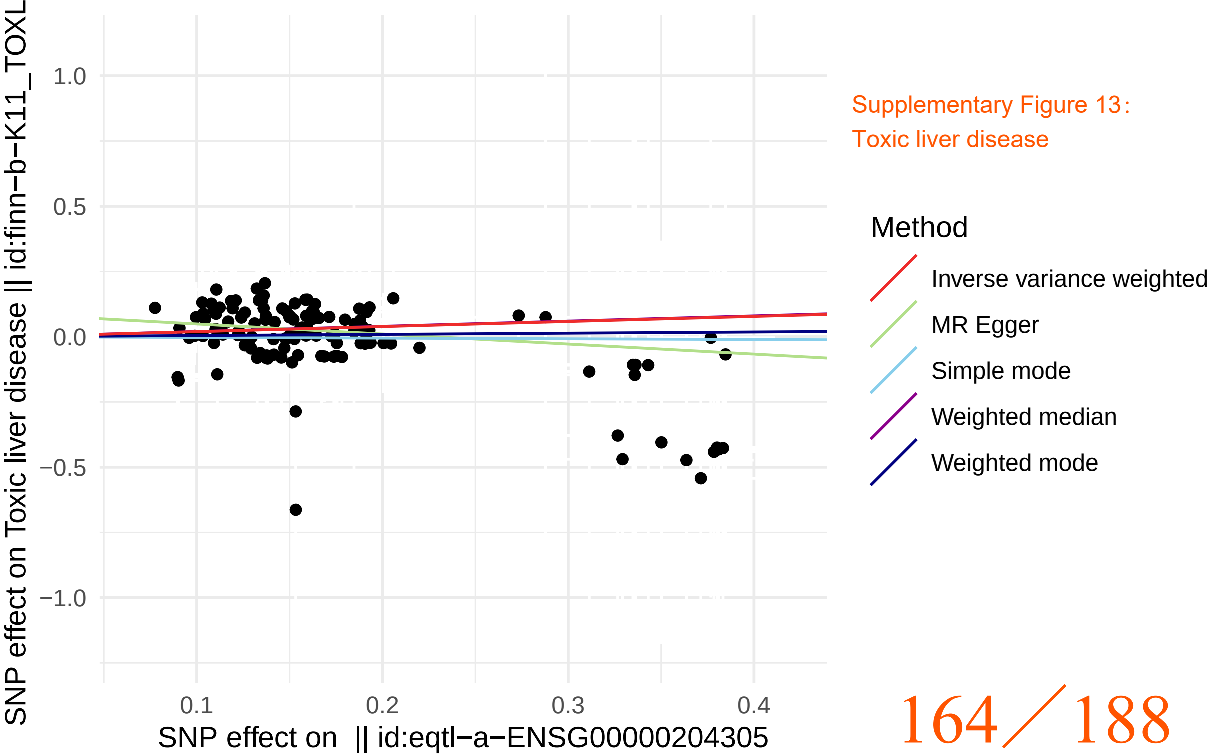

Supplementary Figure 13:
Toxic liver disease

Supplementary Figure 14: Asthma

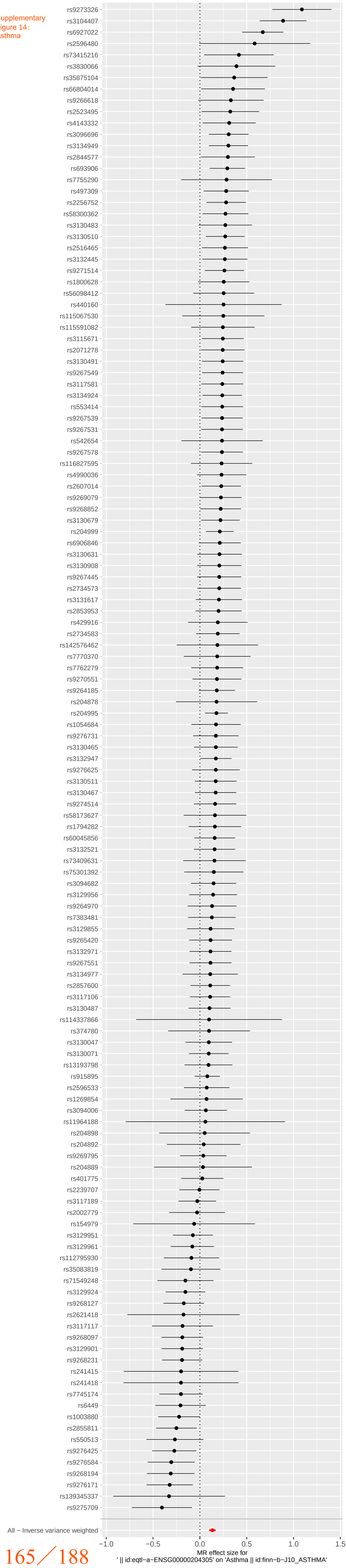

Supplementary Figure 14: Atherosclerosis, excluding cerebral, coronary and PAD

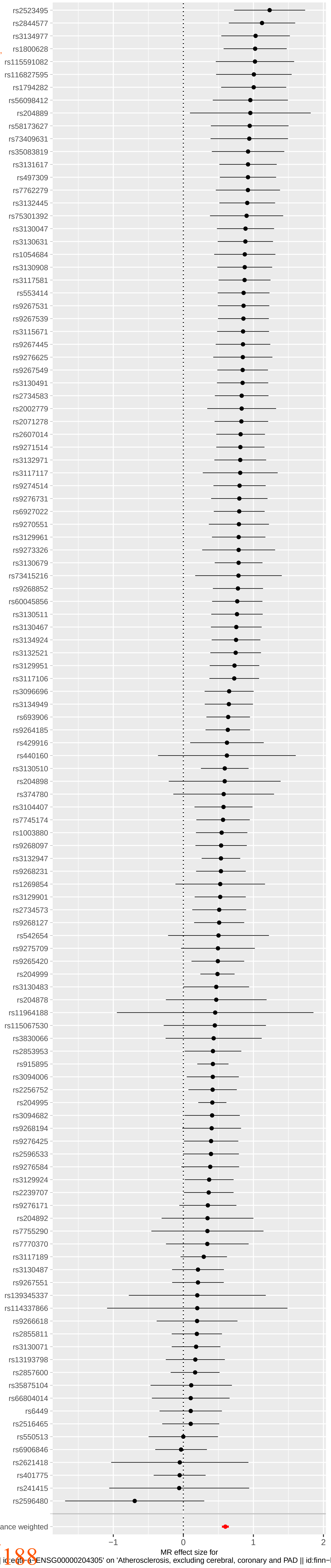

Supplementary Figure 14: Celiac disease

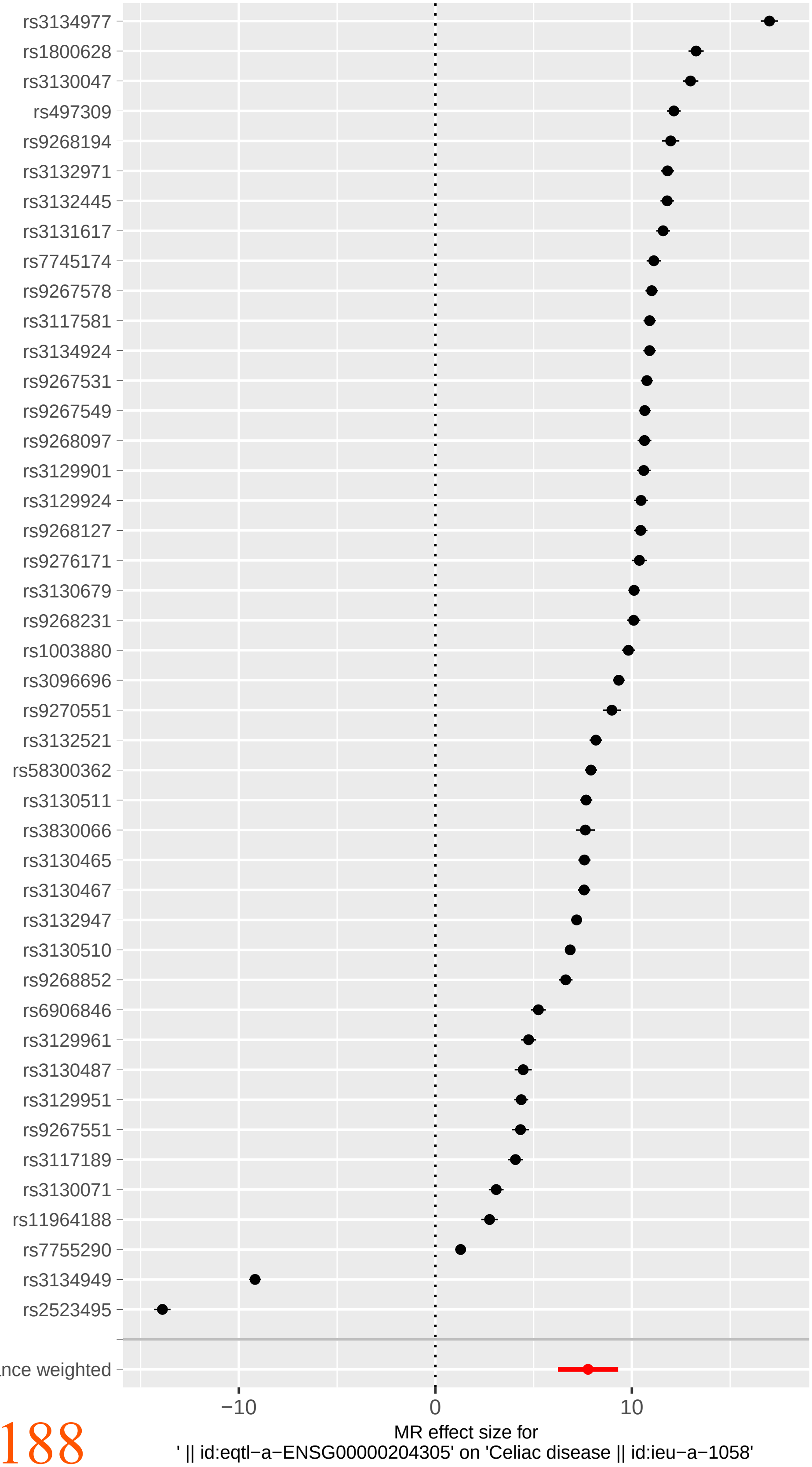

Supplementary Figure 14: Emphysema

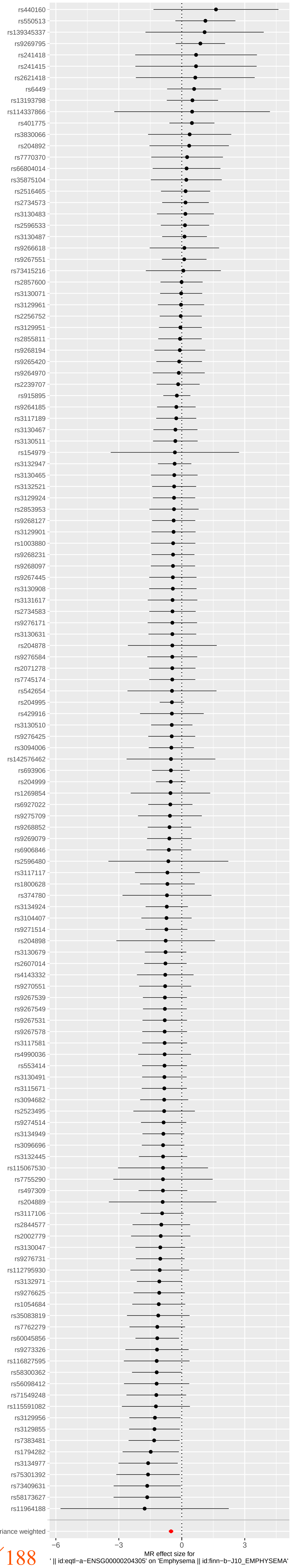

Supplementary Figure 14: Neuralgia and neuritis, unspecified

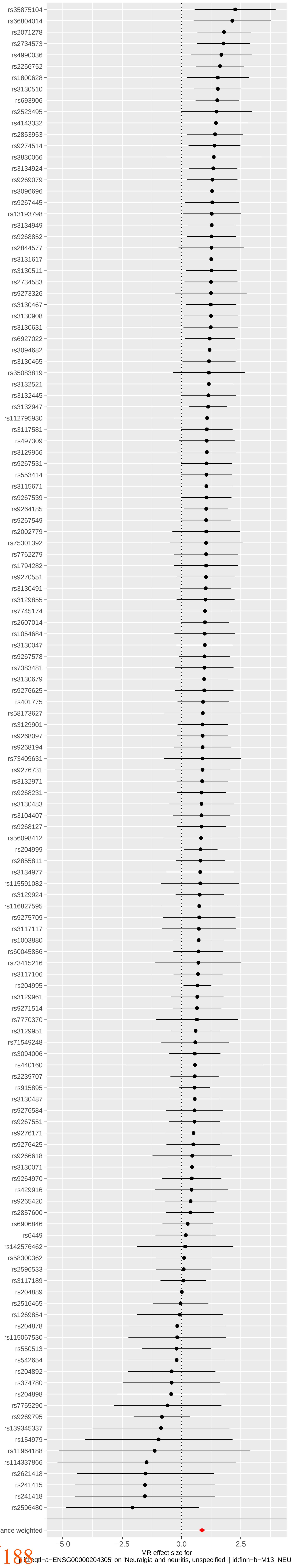

Supplementary Figure 14: Obesity

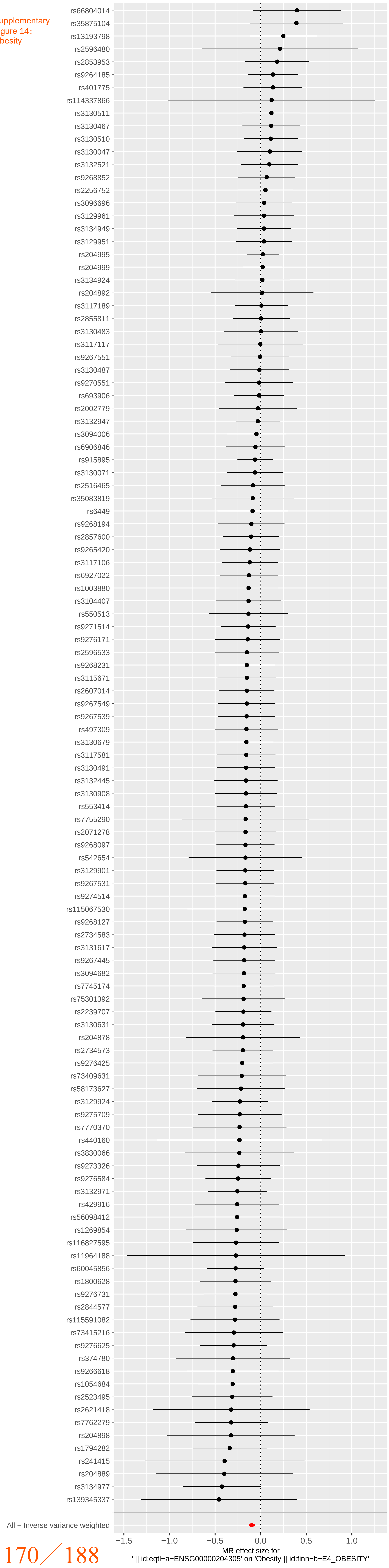

Supplementary Figure 14 :
Pancreatic cancer

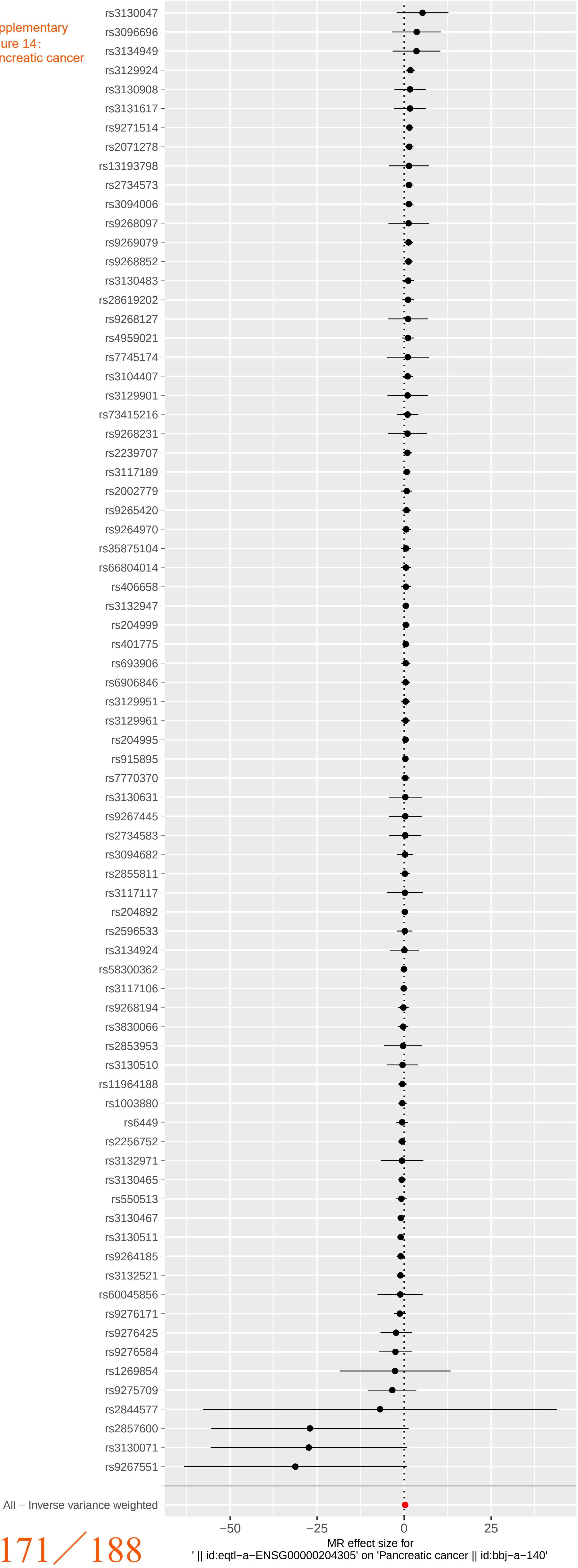

Supplementary Figure 14: Toxic liver disease

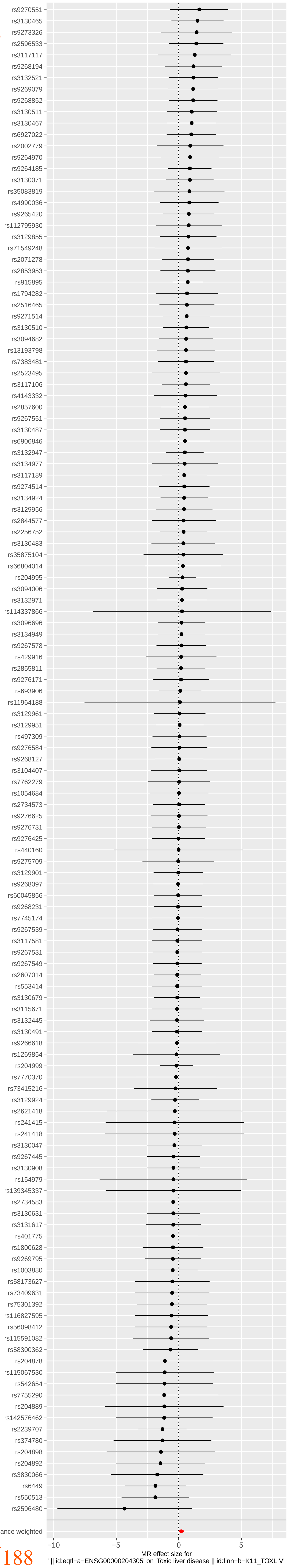

Supplementary Figure 15:
Asthma

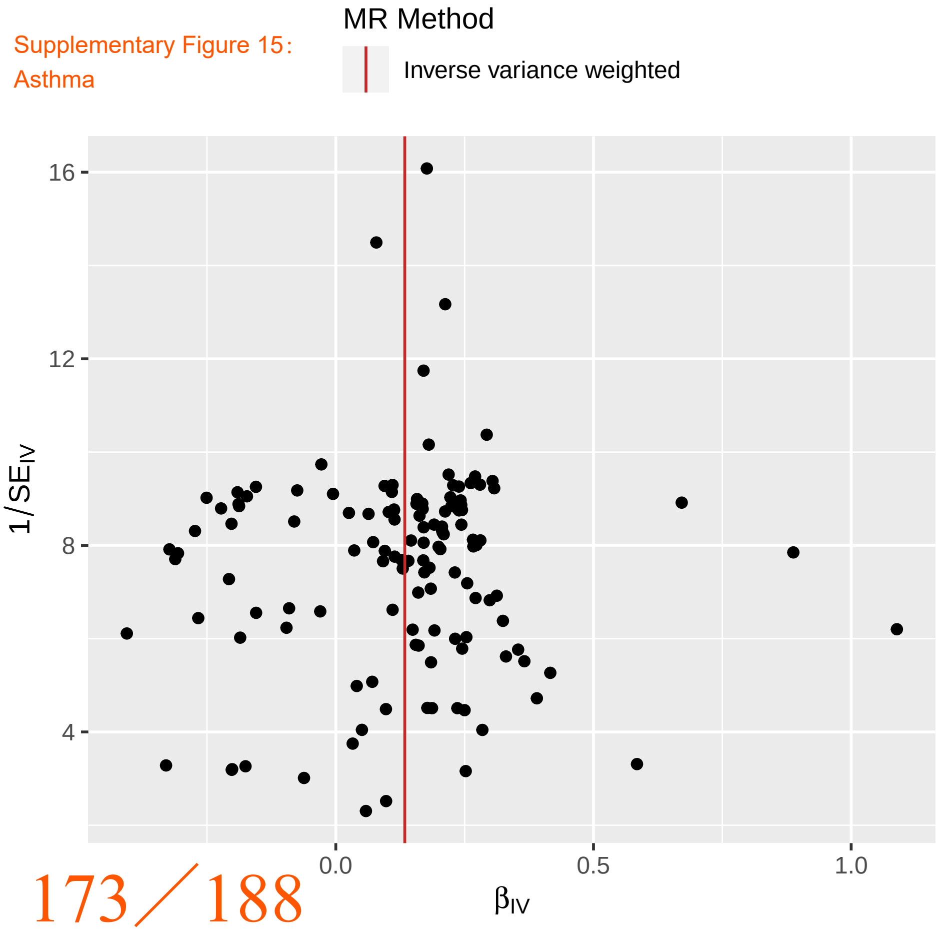

Supplementary Figure 15:
Atherosclerosis, excluding
cerebral, coronary and PAD

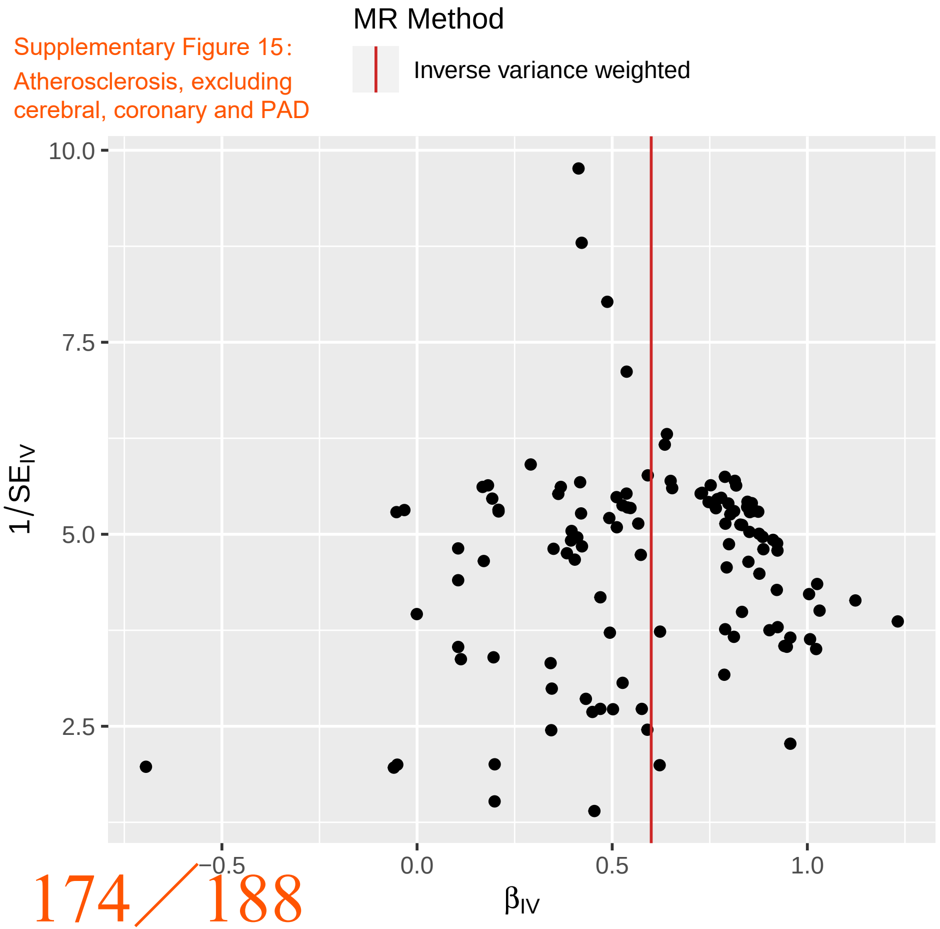

Supplementary Figure 15:
Celiac disease

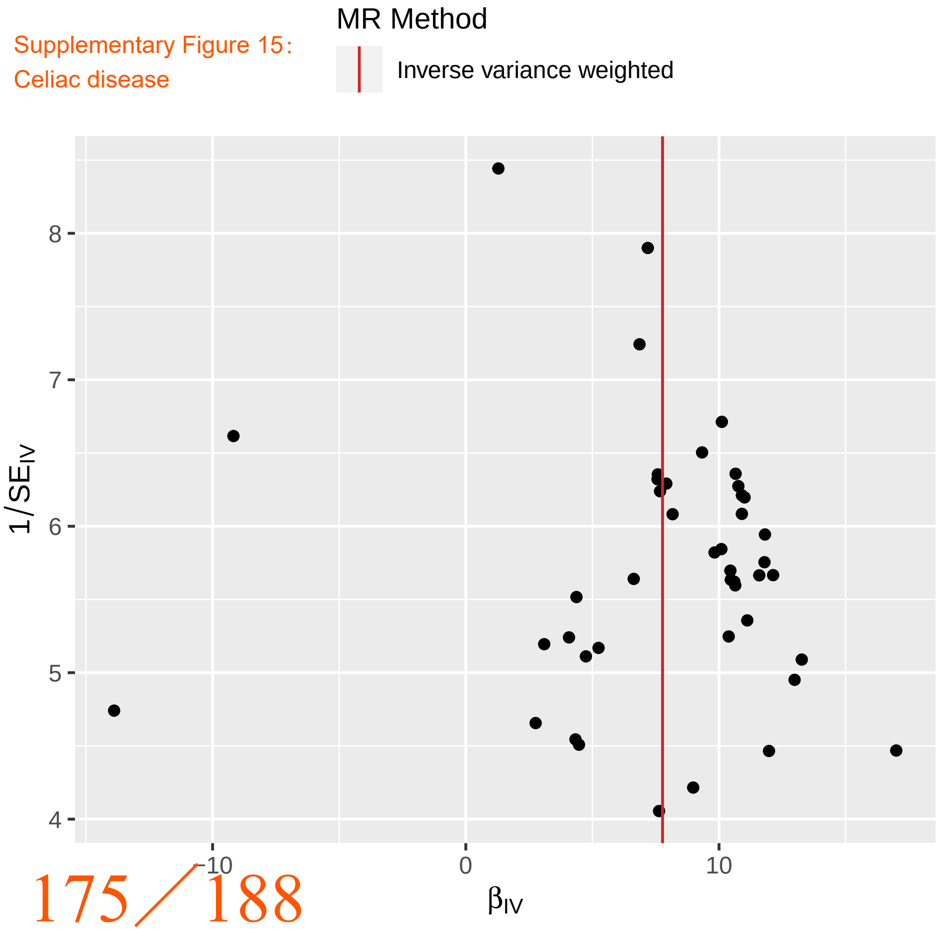

Supplementary Figure 15:
Emphysema

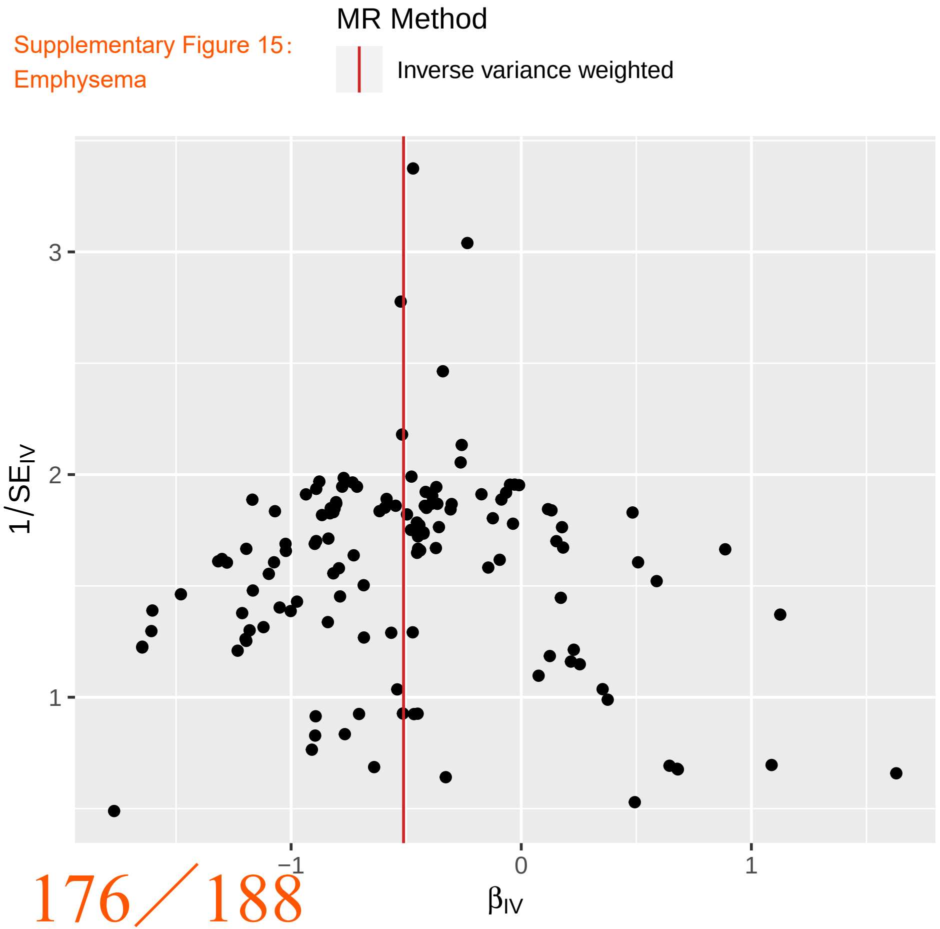

Supplementary Figure 15:
Neuralgia and neuritis, unspecified

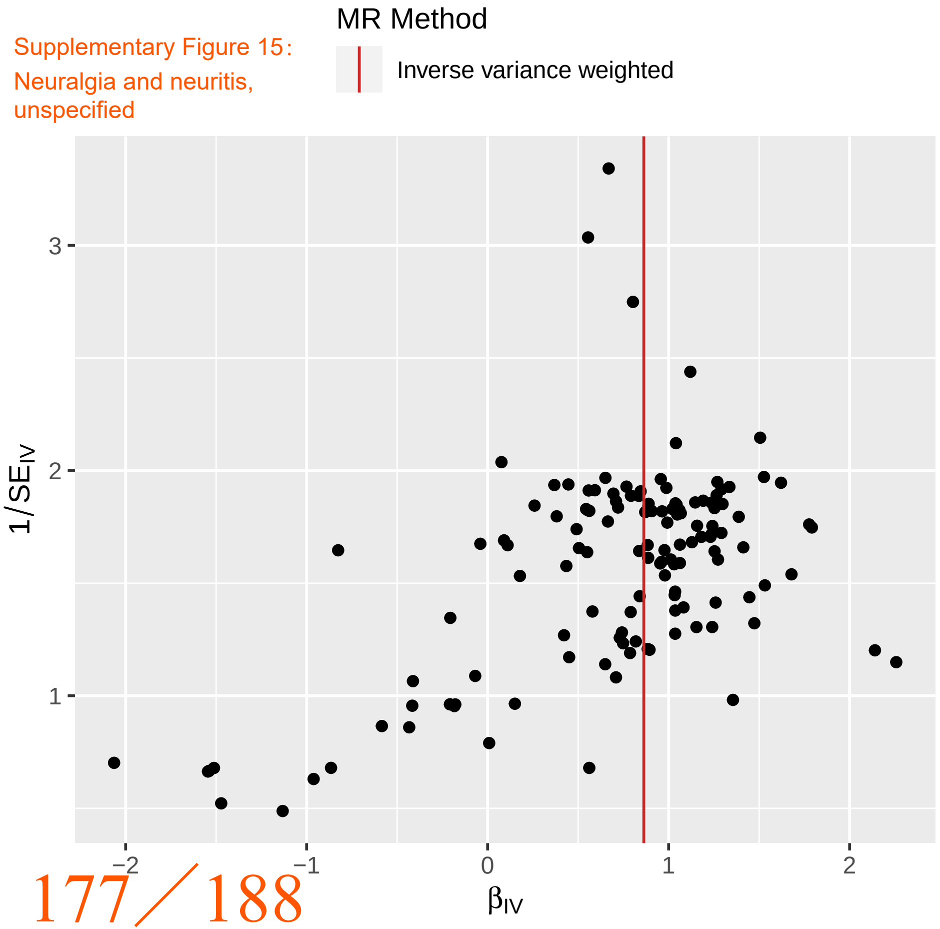

Supplementary Figure 15:
Obesity

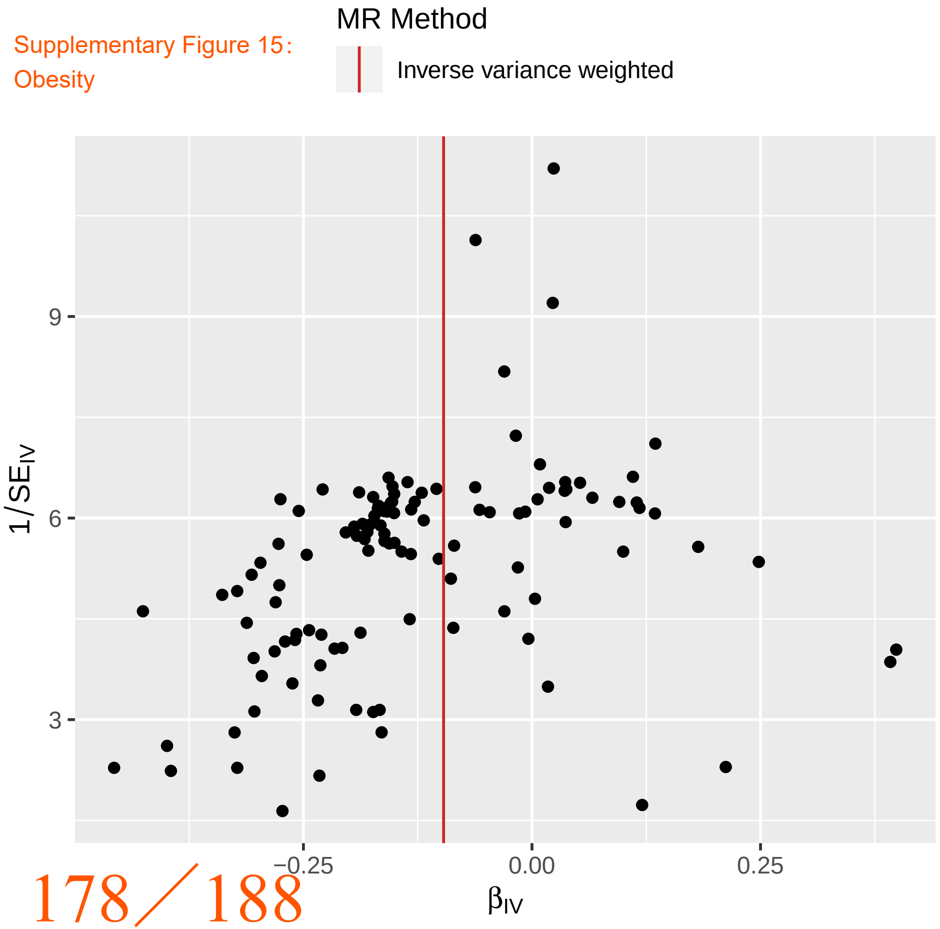

Supplementary Figure 15:
Pancreatic cancer

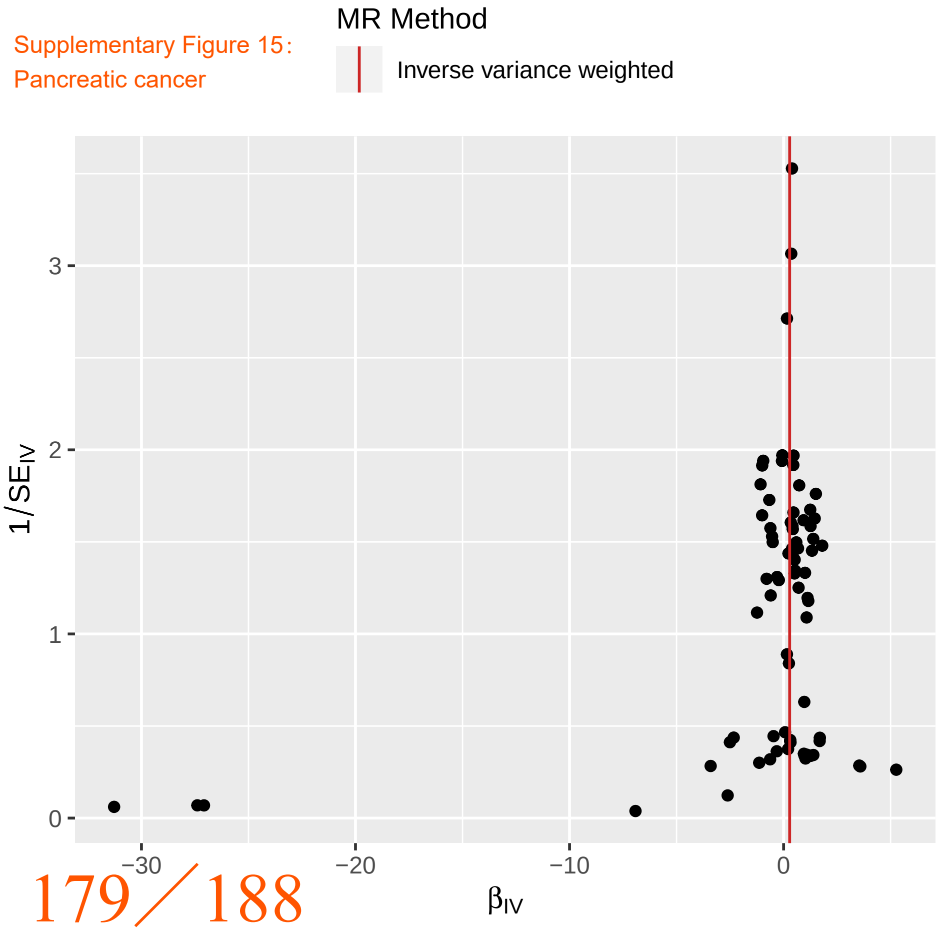

Supplementary Figure 15:
Toxic liver disease

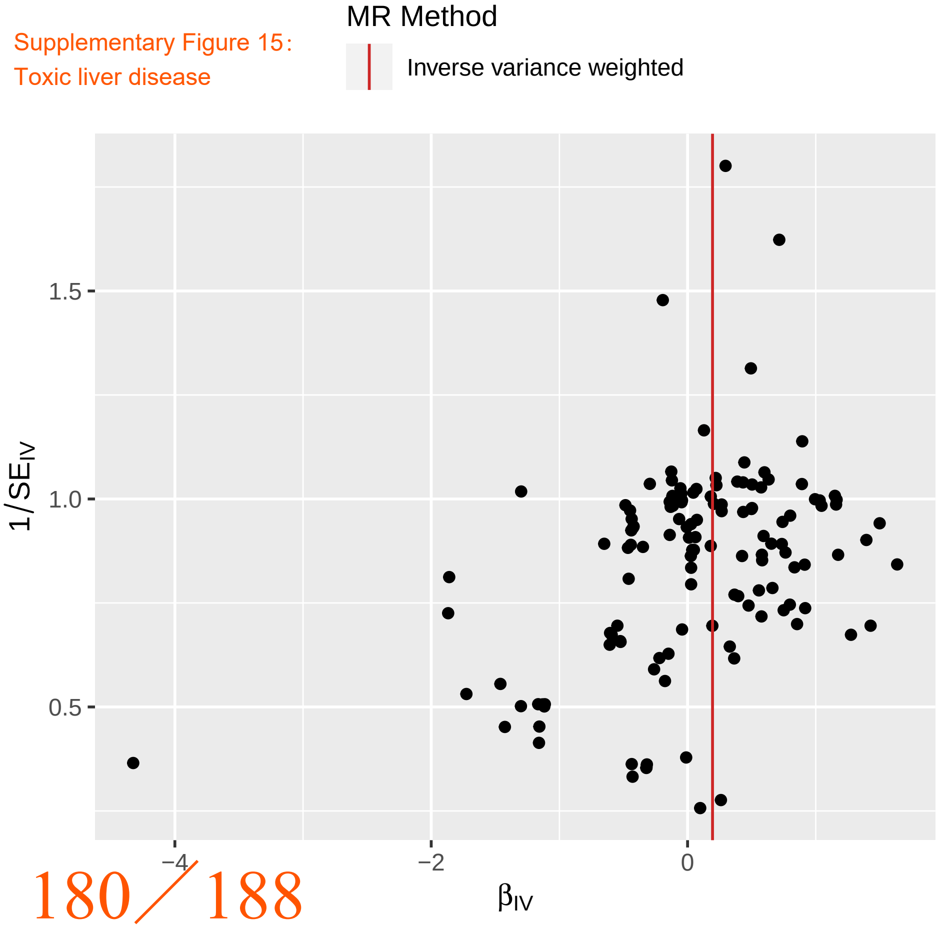

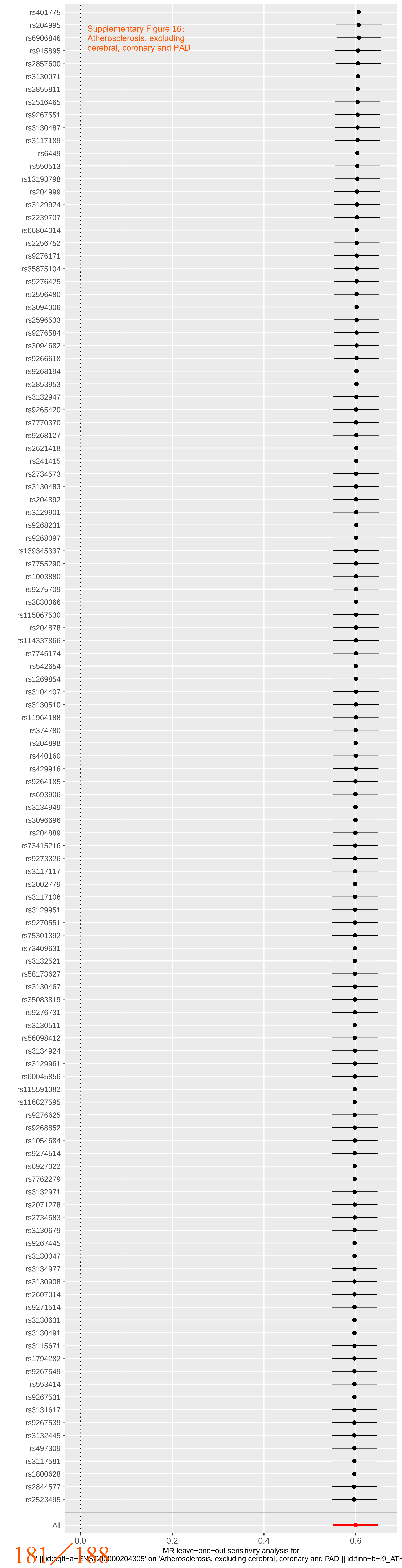
Supplementary Figure 16:
Atherosclerosis, excluding
cerebral, coronary and PAD
rs401775
rs204995
rs6906846
rs915895
rs2857600
rs3130071
rs2855811
rs2516465
rs9267551
rs3130487
rs3117189
rs6449
rs550513
rs13193798
rs204999
rs3129924
rs2239707
rs66804014
rs2256752
rs9276171
rs35875104
rs9276425
rs2596480
rs3094006
rs2596533
rs9276584
rs3094682
rs9266618
rs9268194
rs2853953
rs3132947
rs9265420
rs7770370
rs9268127
rs2621418
rs241415
rs2734573
rs3130483
rs204892
rs3129901
rs9268231
rs9268097
rs139345337
rs7755290
rs1003880
rs9275709
rs3830066
rs115067530
rs204878
rs114337866
rs7745174
rs542654
rs1269854
rs3104407
rs3130510
rs11964188
rs374780
rs204898
rs440160
rs429916
rs9264185
rs693906
rs3134949
rs3096696
rs204889
rs73415216
rs9273326
rs3117117
rs2002779
rs3117106
rs3129951
rs9270551
rs75301392
rs73409631
rs3132521
rs58173627
rs3130467
rs35083819
rs9276731
rs3130511
rs56098412
rs3134924
rs3129961
rs60045856
rs115591082
rs116827595
rs9276625
rs9268852
rs1054684
rs9274514
rs6927022
rs7762279
rs3132971
rs2071278
rs2734583
rs3130679
rs9267445
rs3130047
rs3134977
rs3130908
rs2607014
rs9271514
rs3130631
rs3130491
rs3115671
rs1794282
rs9267549
rs553414
rs9267531
rs3131617
rs9267539
rs3132445
rs497309
rs3117581
rs1800628
rs2844577
rs2523495
All
0.0
0.2
0.4
0.6
MR leave−one−out sensitivity analysis for
' || id:eqtl−a−ENSG00000204305' on 'Atherosclerosis, excluding cerebral, coronary and PAD || id:finn−b−I9_ATH

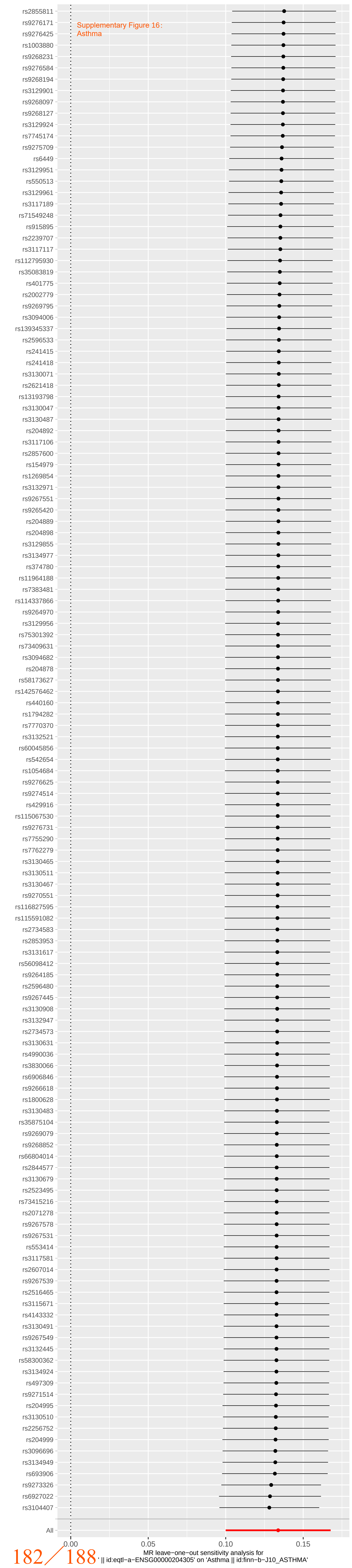
Supplementary Figure 16:
Asthma
rs2855811
rs9276171
rs9276425
rs1003880
rs9268231
rs9276584
rs9268194
rs3129901
rs9268097
rs9268127
rs3129924
rs7745174
rs9275709
rs6449
rs3129951
rs550513
rs3129961
rs3117189
rs71549248
rs915895
rs2239707
rs3117117
rs112795930
rs35083819
rs401775
rs2002779
rs9269795
rs3094006
rs139345337
rs2596533
rs241415
rs241418
rs3130071
rs2621418
rs13193798
rs3130047
rs3130487
rs204892
rs3117106
rs2857600
rs154979
rs1269854
rs3132971
rs9267551
rs9265420
rs204889
rs204898
rs3129855
rs3134977
rs374780
rs11964188
rs7383481
rs114337866
rs9264970
rs3129956
rs75301392
rs73409631
rs3094682
rs204878
rs58173627
rs142576462
rs440160
rs1794282
rs7770370
rs3132521
rs60045856
rs542654
rs1054684
rs9276625
rs9274514
rs429916
rs115067530
rs9276731
rs7755290
rs7762279
rs3130465
rs3130511
rs3130467
rs9270551
rs116827595
rs115591082
rs2734583
rs2853953
rs3131617
rs56098412
rs9264185
rs2596480
rs9267445
rs3130908
rs3132947
rs2734573
rs3130631
rs4990036
rs3830066
rs6906846
rs9266618
rs1800628
rs3130483
rs35875104
rs9269079
rs9268852
rs66804014
rs2844577
rs3130679
rs2523495
rs73415216
rs2071278
rs9267578
rs9267531
rs553414
rs3117581
rs2607014
rs9267539
rs2516465
rs3115671
rs4143332
rs3130491
rs9267549
rs3132445
rs58300362
rs3134924
rs497309
rs9271514
rs204995
rs3130510
rs2256752
rs204999
rs3096696
rs3134949
rs693906
rs9273326
rs6927022
rs3104407
All
0.00
0.05
0.10
0.15
MR leave−one−out sensitivity analysis for
' || id:eqtl−a−ENSG00000204305' on 'Asthma || id:finn−b−J10_ASTHMA'

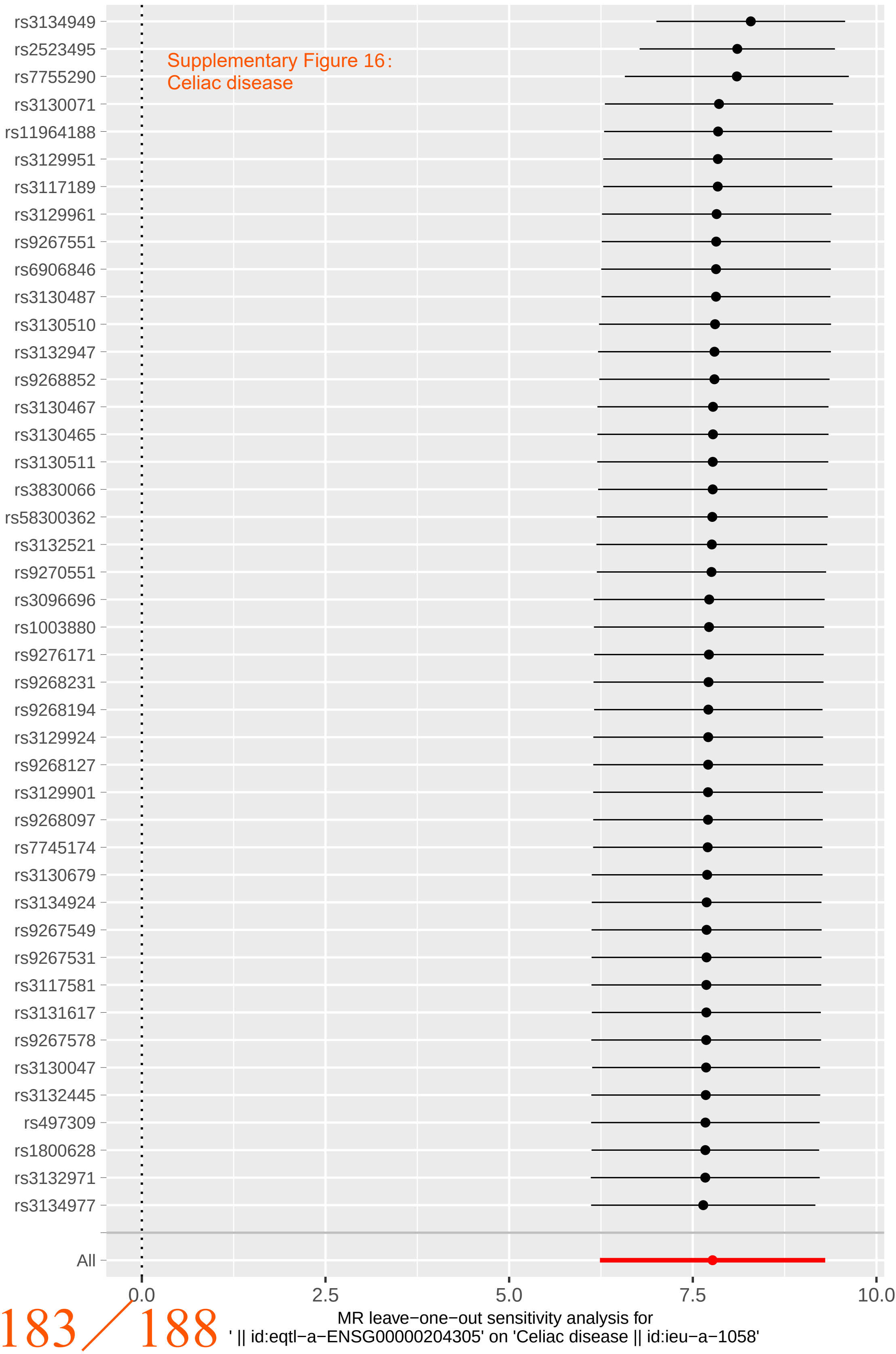

Supplementary Figure 16:
Celiac disease
rs3134949
rs2523495
rs7755290
rs3130071
rs11964188
rs3129951
rs3117189
rs3129961
rs9267551
rs6906846
rs3130487
rs3130510
rs3132947
rs9268852
rs3130467
rs3130465
rs3130511
rs3830066
rs58300362
rs3132521
rs9270551
rs3096696
rs1003880
rs9276171
rs9268231
rs9268194
rs3129924
rs9268127
rs3129901
rs9268097
rs7745174
rs3130679
rs3134924
rs9267549
rs9267531
rs3117581
rs3131617
rs9267578
rs3130047
rs3132445
rs497309
rs1800628
rs3132971
rs3134977
All
0.0
2.5
5.0
7.5
10.0
MR leave−one−out sensitivity analysis for
' || id:eqtl−a−ENSG00000204305' on 'Celiac disease || id:ieu−a−1058'

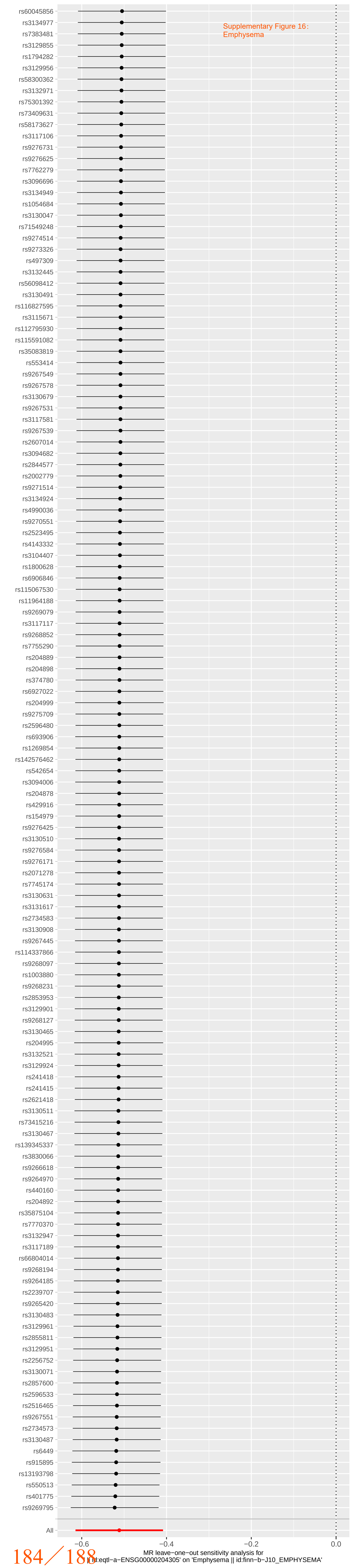
Supplementary Figure 16:
Emphysema
rs60045856
rs3134977
rs7383481
rs3129855
rs1794282
rs3129956
rs58300362
rs3132971
rs75301392
rs73409631
rs58173627
rs3117106
rs9276731
rs9276625
rs7762279
rs3096696
rs3134949
rs1054684
rs3130047
rs71549248
rs9274514
rs9273326
rs497309
rs3132445
rs56098412
rs3130491
rs116827595
rs3115671
rs112795930
rs115591082
rs35083819
rs553414
rs9267549
rs9267578
rs3130679
rs9267531
rs3117581
rs9267539
rs2607014
rs3094682
rs2844577
rs2002779
rs9271514
rs3134924
rs4990036
rs9270551
rs2523495
rs4143332
rs3104407
rs1800628
rs6906846
rs115067530
rs11964188
rs9269079
rs3117117
rs9268852
rs7755290
rs204889
rs204898
rs374780
rs6927022
rs204999
rs9275709
rs2596480
rs693906
rs1269854
rs142576462
rs542654
rs3094006
rs204878
rs429916
rs154979
rs9276425
rs3130510
rs9276584
rs9276171
rs2071278
rs7745174
rs3130631
rs3131617
rs2734583
rs3130908
rs9267445
rs114337866
rs9268097
rs1003880
rs9268231
rs2853953
rs3129901
rs9268127
rs3130465
rs204995
rs3132521
rs3129924
rs241418
rs241415
rs2621418
rs3130511
rs73415216
rs3130467
rs139345337
rs3830066
rs9266618
rs9264970
rs440160
rs204892
rs35875104
rs7770370
rs3132947
rs3117189
rs66804014
rs9268194
rs9264185
rs2239707
rs9265420
rs3130483
rs3129961
rs2855811
rs3129951
rs2256752
rs3130071
rs2857600
rs2596533
rs2516465
rs9267551
rs2734573
rs3130487
rs6449
rs915895
rs13193798
rs550513
rs401775
rs9269795
All
−0.6
−0.4
−0.2
0.0
MR leave−one−out sensitivity analysis for
'id:eqtl−a−ENSG00000204305' on 'Emphysema || id:finn−b−J10_EMPHYSEMA'

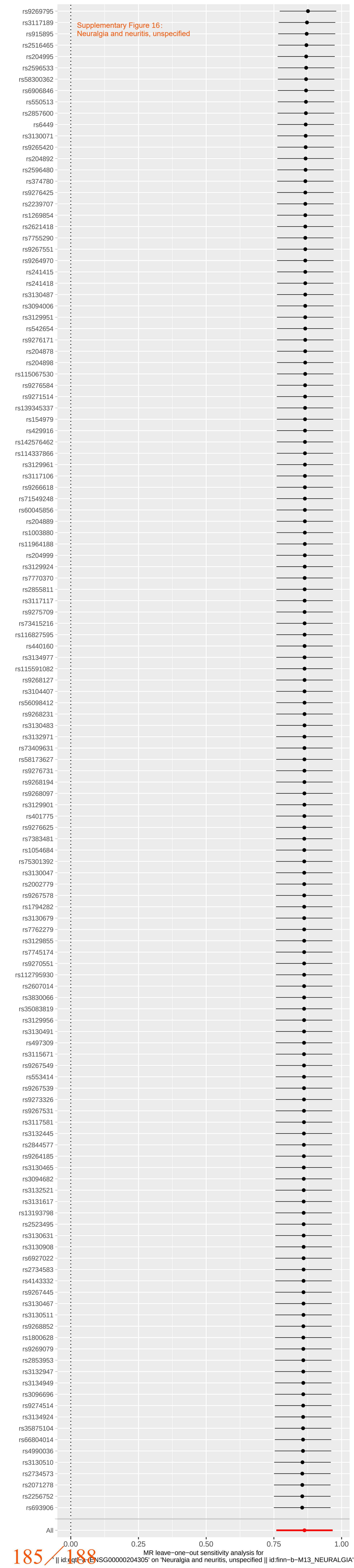

Supplementary Figure 16:
Neuralgia and neuritis, unspecified

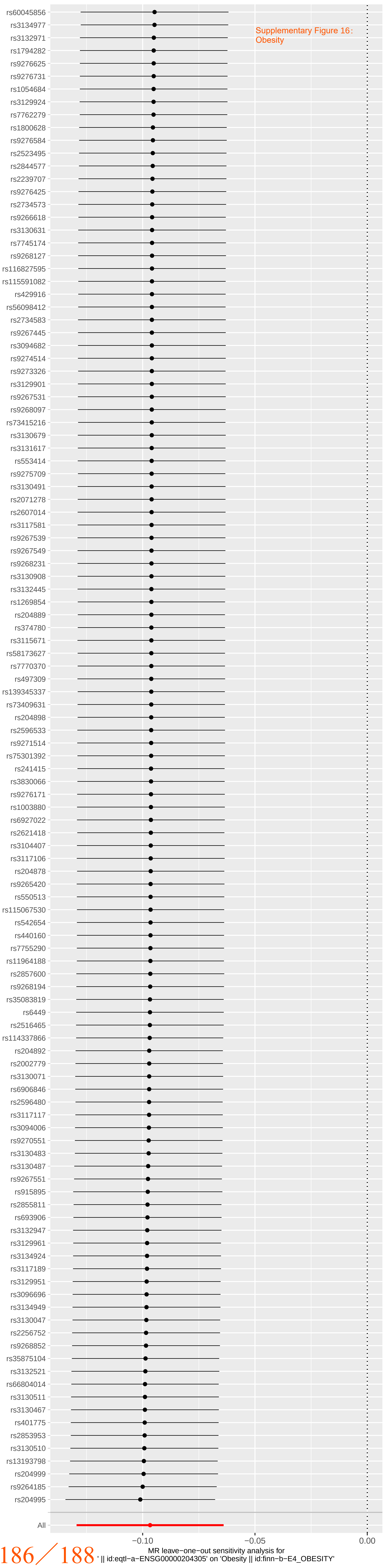

Supplementary Figure 16:
Obesity
rs60045856
rs3134977
rs3132971
rs1794282
rs9276625
rs9276731
rs1054684
rs3129924
rs7762279
rs1800628
rs9276584
rs2523495
rs2844577
rs2239707
rs9276425
rs2734573
rs9266618
rs3130631
rs7745174
rs9268127
rs116827595
rs115591082
rs429916
rs56098412
rs2734583
rs9267445
rs3094682
rs9274514
rs9273326
rs3129901
rs9267531
rs9268097
rs73415216
rs3130679
rs3131617
rs553414
rs9275709
rs3130491
rs2071278
rs2607014
rs3117581
rs9267539
rs9267549
rs9268231
rs3130908
rs3132445
rs1269854
rs204889
rs374780
rs3115671
rs58173627
rs7770370
rs497309
rs139345337
rs73409631
rs204898
rs2596533
rs9271514
rs75301392
rs241415
rs3830066
rs9276171
rs1003880
rs6927022
rs2621418
rs3104407
rs3117106
rs204878
rs9265420
rs550513
rs115067530
rs542654
rs440160
rs7755290
rs11964188
rs2857600
rs9268194
rs35083819
rs6449
rs2516465
rs114337866
rs204892
rs2002779
rs3130071
rs6906846
rs2596480
rs3117117
rs3094006
rs9270551
rs3130483
rs3130487
rs9267551
rs915895
rs2855811
rs693906
rs3132947
rs3129961
rs3134924
rs3117189
rs3129951
rs3096696
rs3134949
rs3130047
rs2256752
rs9268852
rs35875104
rs3132521
rs66804014
rs3130511
rs3130467
rs401775
rs2853953
rs3130510
rs13193798
rs204999
rs9264185
rs204995
All
−0.10
−0.05
0.00
MR leave−one−out sensitivity analysis for
' || id:eqtl−a−ENSG00000204305' on 'Obesity || id:finn−b−E4_OBESITY'

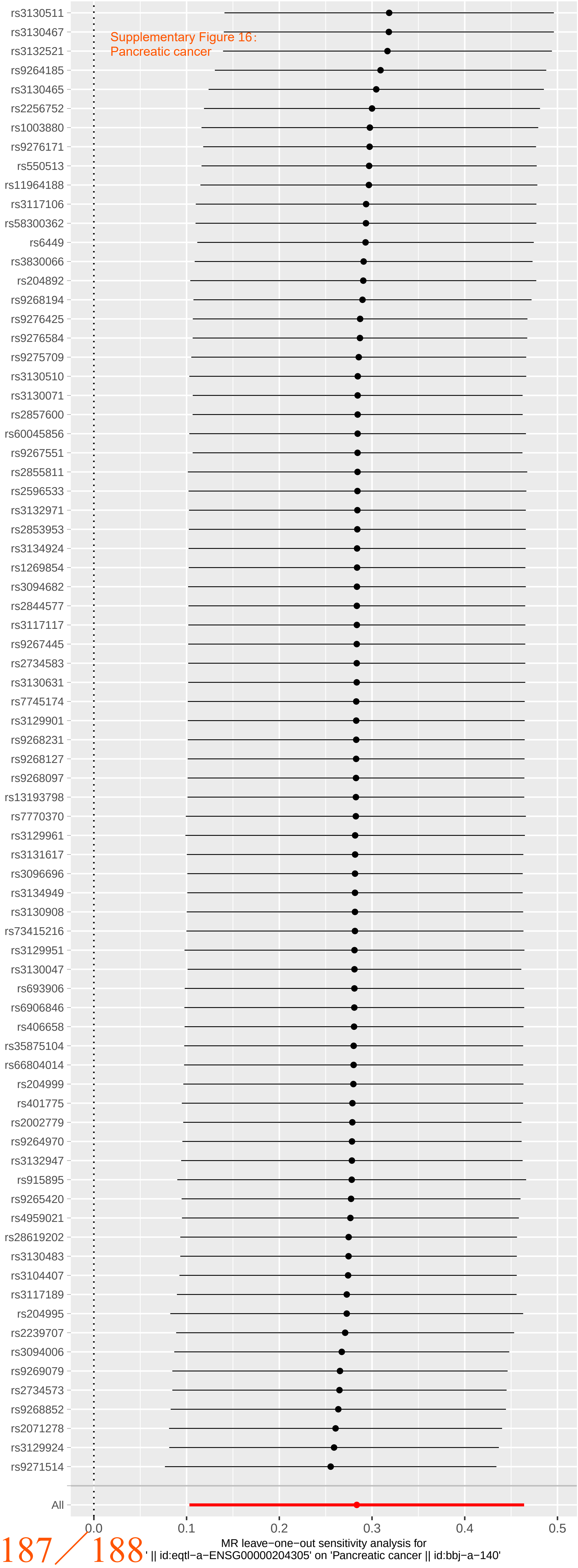

Supplementary Figure 16:
Pancreatic cancer

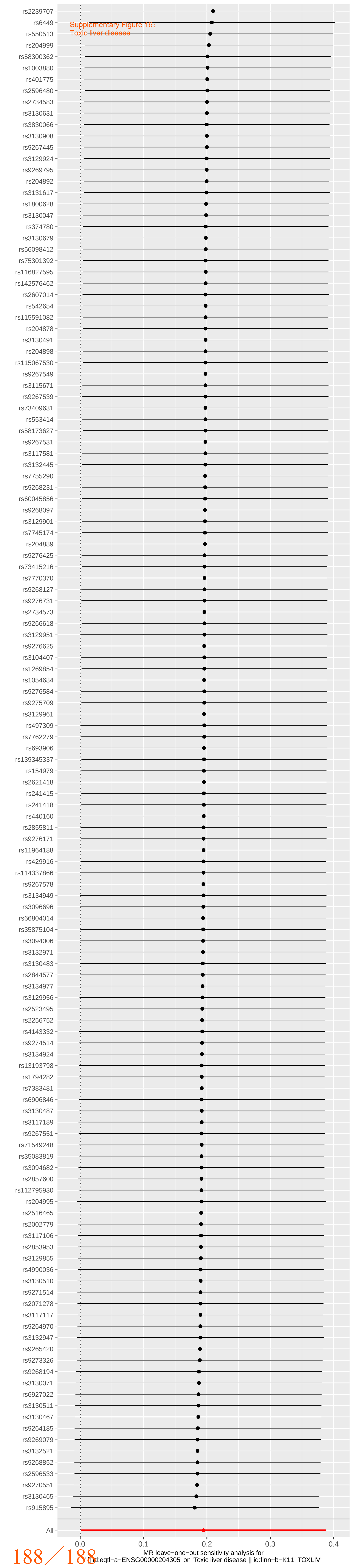
Supplementary Figure 16:
Toxic liver disease
rs2239707
rs6449
rs550513
rs204999
rs58300362
rs1003880
rs401775
rs2596480
rs2734583
rs3130631
rs3830066
rs3130908
rs9267445
rs3129924
rs9269795
rs204892
rs3131617
rs1800628
rs3130047
rs374780
rs3130679
rs56098412
rs75301392
rs116827595
rs142576462
rs2607014
rs542654
rs115591082
rs204878
rs3130491
rs204898
rs115067530
rs9267549
rs3115671
rs9267539
rs73409631
rs553414
rs58173627
rs9267531
rs3117581
rs3132445
rs7755290
rs9268231
rs60045856
rs9268097
rs3129901
rs7745174
rs204889
rs9276425
rs73415216
rs7770370
rs9268127
rs9276731
rs2734573
rs9266618
rs3129951
rs9276625
rs3104407
rs1269854
rs1054684
rs9276584
rs9275709
rs3129961
rs497309
rs7762279
rs693906
rs139345337
rs154979
rs2621418
rs241415
rs241418
rs440160
rs2855811
rs9276171
rs11964188
rs429916
rs114337866
rs9267578
rs3134949
rs3096696
rs66804014
rs35875104
rs3094006
rs3132971
rs3130483
rs2844577
rs3134977
rs3129956
rs2523495
rs2256752
rs4143332
rs9274514
rs3134924
rs13193798
rs1794282
rs7383481
rs6906846
rs3130487
rs3117189
rs9267551
rs71549248
rs35083819
rs3094682
rs2857600
rs112795930
rs204995
rs2516465
rs2002779
rs3117106
rs2853953
rs3129855
rs4990036
rs3130510
rs9271514
rs2071278
rs3117117
rs9264970
rs3132947
rs9265420
rs9273326
rs9268194
rs3130071
rs6927022
rs3130511
rs3130467
rs9264185
rs9269079
rs3132521
rs9268852
rs2596533
rs9270551
rs3130465
rs915895
All
0.0
0.1
0.2
0.3
0.4
MR leave−one−out sensitivity analysis for
'id:eqtl−a−ENSG00000204305' on 'Toxic liver disease || id:finn−b−K11_TOXLIV'
